# Amide and Thioester Synthesis Via Oxidative Coupling of Alcohols with Amines or Thiols Using Alcohol Dehydrogenases

**DOI:** 10.1002/anie.202515469

**Published:** 2025-09-30

**Authors:** Matteo Damian, Vasilis Tseliou, Patrick Peters, Tanja Knaus, Francesco G. Mutti

**Affiliations:** ^1^ Van ’t Hoff Institute for Molecular Sciences HIMS‐Biocat University of Amsterdam Science Park 904 Amsterdam 1098 XH The Netherlands

**Keywords:** Alcohol dehydrogenases, Amide synthesis, Biocatalysis, Enzyme promiscuity, Thioester synthesis

## Abstract

Amide and thioester moieties are prevalent in pharmaceuticals, natural products, and functional materials, but their chemical synthesis suffers from poor atom economy and ungreen conditions, while biocatalytic methods require ATP‐dependent enzymes, activated intermediates, or show limited scope and activity. Here, we report the oxidative coupling of alcohols with ammonia or amines catalyzed by alcohol dehydrogenases (ADHs) via hemiaminal intermediates to form primary and secondary amides at pH 9.5–10.5. Pf‐ADH preferably converted linear aliphatic or arylaliphatic alcohols (up to 90% conversion), while Pp‐ADH and Aa‐ADH preferably converted branched or aromatic alcohols (up to 99% conversion). Preparative‐scale synthesis of an N‐methyl amide gave >99% conversion and 87% isolated yield. The method was extended to thioacid and thioester formation via hemithioacetal intermediates using hydrogen sulfide or thiols at pH 7. Pf‐ADH favored linear aliphatic alcohols (up to 93% conversion), Pp‐ADH branched alcohols (up to 82% conversion), and Aa‐ADH aromatic alcohols (up to 98% conversion). A KPi/MTBE biphasic system enabled the reaction with poorly soluble long‐chain thiols. Structure‐guided engineering of Aa‐ADH led to the Y151A and L186A variants with expanded activity toward longer‐chain amines or thiols. This work highlights how enzyme promiscuity with protein engineering can enable new‐to‐nature synthetic pathways for the production of valuable compounds.

## Graphical abstract

Alcohol dehydrogenases (ADHs) are best known for reducing ketones to chiral alcohols, but their oxidative potential is rarely exploited. Here, we show that selected ADHs catalyze newly discovered promiscuous transformations: the oxidative coupling of primary alcohols with amines or thiols, enabling the direct and efficient synthesis of a broad range of amides and thioesters under mild reaction conditions.

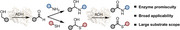

## Introduction

The formation of amide bonds represents a fundamental transformation in organic synthesis, given the prevalence of amides in both natural and synthetic compounds. Notably, amide functionalities are found in approximately half of all compounds disclosed in medicinal chemistry patents,^[^ ^1^ ^]^ and at least one amide group is present in 122 of the Top 200 Small Molecule Drugs by retail sales in 2024.^[^ ^2^ ^]^ The available methods for amide synthesis can generally be classified into four groups: condensation from activated derivatives, thermal amidation, catalytic amidation utilizing metals or boron derivatives, and light or thermal‐driven rearrangement.^[^ ^3^, ^4^, ^5^, ^6^, ^7^, ^8^, ^9^, ^10^, ^11^, ^12^, ^13^, ^14^, ^15^, ^16^, ^17^ ^]^ Regrettably, most of these methods suffer from poor atom efficiency and generation of copious amounts of waste.^[^ ^18^, ^19^ ^]^ Thioesters have also attracted significant interest owing to their broad utility in medicinal chemistry, materials science, and as intermediates in the synthesis of pharmaceuticals and natural products.^[^ ^20^, ^21^ ^]^ Conventional chemical approaches to thioester synthesis typically rely on activated acyl derivatives^[^ ^22^, ^23^ ^]^ or transition metal catalysis.^[^ ^24^, ^25^, ^26^, ^27^, ^28^, ^29^ ^]^ However, these methods suffer from limitations: the preparation of activated intermediates often lacks atom economy and selectivity, while metal‐catalyzed protocols frequently require toxic CO gas and exhibit restricted substrate scope, all factors that hinder their scalability and practical application.^[^ ^28^, ^29^, ^30^ ^]^

**Figure 1:**
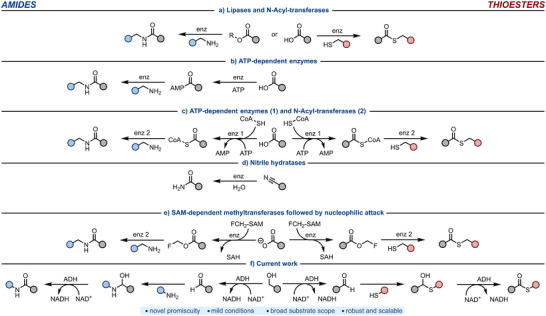
Amide or thioester synthesis catalyzed by: a) lipases or *N*‐acyl‐transferases from carboxylic acids or activated esters usually in neat organic solvents or aqueous buffer; b) ATP‐dependent synthetases from carboxylic acids; c) ATP‐dependent synthetases and *N*‐acyl‐transferases from CoA‐thioester intermediates; d) nitrile hydratases from nitriles. e) SAM‐dependent methyltransferase, performing fluoromethylation of carboxylates followed by nucleophilic attack of amines or thiols; f) This work: oxidative coupling of alcohols and amines catalyzed by alcohol dehydrogenases.

Biocatalytic amide synthesis is possible by using diverse enzymes.^[^ ^31^, ^32^ ^]^ Lipases and *N*‐acyl‐transferases synthesize amides from esters or carboxylic acids, both in neat organic solvents and in aqueous buffer (Figure 1a).^[^ ^33^, ^34^, ^35^, ^36^, ^37^, ^38^, ^39^, ^40^, ^41^, ^42^, ^43^, ^44^ ^]^ In aqueous buffer, the rate of amide synthesis can be enhanced by enzyme engineering, while at the same time undesired hydrolytic activity is suppressed.^[^ ^40^, ^45^, ^46^ ^]^ Some ATP‐dependent synthetases catalyze the formation of amides starting from carboxylic acids, ATP and amines (Figure 1b).^[^ ^47^, ^48^, ^49^, ^50^, ^51^, ^52^, ^53^, ^54^, ^55^ ^]^ By combining ATP with CoASH, *N*‐acyl‐transferases can also couple the activated thioester with an amine to yield amides (Figure 1c).^[^ ^56^, ^57^ ^]^ Amides can also be synthesized by hydrolysis of nitrile using nitrile hydratases (Figure 1d).^[^ ^31^, ^58^, ^59^, ^60^ ^]^ Last year, an alternative approach was reported in which SAM‐dependent methyltransferases were used to fluoromethylate carboxylates, generating fluoromethyl esters that subsequently reacted with amine nucleophiles to yield the corresponding amides. (Figure 1e).^[^ ^61^ ^]^

As for amide formation, biocatalytic thioester synthesis can also be accomplished using promiscuous hydrolase activity, starting from thiols and vinyl esters or carboxylic acids in neat organic solvents or—commonly after enzyme engineering—in aqueous buffer (Figure 1a)^[^ ^62^, ^63^, ^64^, ^65^, ^66^ ^]^ However, most biocatalytic approaches involve the generation of coenzyme A thioesters using ATP and coenzyme A‐dependent synthetases (Figure 1c).^[^ ^67^, ^68^ ^]^ These coenzyme A thioesters can then be used as substrates for CoA‐dependent transferases to generate various products,^[^ ^69^, ^70^, ^71^ ^]^ or for oxalyl‐CoA decarboxylase (OXC)/2‐hydroxyacyl‐CoA lyase (HACL), in combination with aromatic aldehydes, to obtain α‐hydroxy carboxylic acids.^[^ ^72^ ^]^ S‐N‐acetylcysteamine (SNAC), a thioester derivative of *N*‐acetylcysteamine, can also replace coenzyme A in the synthesis of thioesters from short‐chain fatty acids catalyzed by promiscuous adenylate‐forming enzymes.^[^ ^54^ ^]^ Finally, fluoromethylation of carboxylates using SAM‐dependent methyltransferases, followed by nucleophilic attack by a thiol, is used to produce thioesters, similarly to what was described for amides (Figure 1e).^[^ ^61^ ^]^

Alcohol dehydrogenases (ADHs), also called keto‐ or aldo‐reductases, naturally catalyze the conversion of primary alcohols to aldehydes and secondary alcohols to ketones while concomitantly reducing NAD^+^ to NADH or the opposite reduction reactions.^[^ ^73^, ^74^, ^75^, ^76^, ^77^ ^]^ In this work, we hypothesized that some alcohol dehydrogenases (ADHs) could exhibit promiscuous catalytic activity on hemiaminal intermediates. During the catalytic cycle of an ADH, a hemiaminal could form in situ by the intermolecular attack of an amine onto an aldehyde, the latter of which can also be generated through the natural oxidation of primary alcohols catalyzed by ADHs (Figure 1f). The only evidence in the literature about the ability of an ADH to oxidize a hemiaminal concerns an intramolecular cyclization to give a lactam product.^[^ ^78^ ^]^ Therefore, we envisioned that the potentially broad promiscuous activity of certain ADHs on hemiaminals could enable enzymatic amide synthesis through the direct coupling of alcohols and amines. This method offers several advantages over the other biocatalytic strategies discussed: it does not require neat organic solvents, as in the case of hydrolases, which can reduce enzyme activity, stability, and substrate scope; it avoids the need for ATP as a cofactor, unlike ATP‐dependent amide bond‐forming enzymes (the required NAD⁺ is both cheaper and readily recyclable using atmospheric O_2_); and it does not rely on activated acyl donors, as required by *N*‐acyltransferases, which also suffer from limited substrate scope. It also avoids the limitations of nitrile hydratases, which are restricted to the formation of primary amides and are more complex to express due to their heterodimeric nature. Notably, in this work, we demonstrated that this type of promiscuous ADH activity can also be extended to other nucleophiles, such as thiols, to generate thioesters as final products.

**Figure 2:**
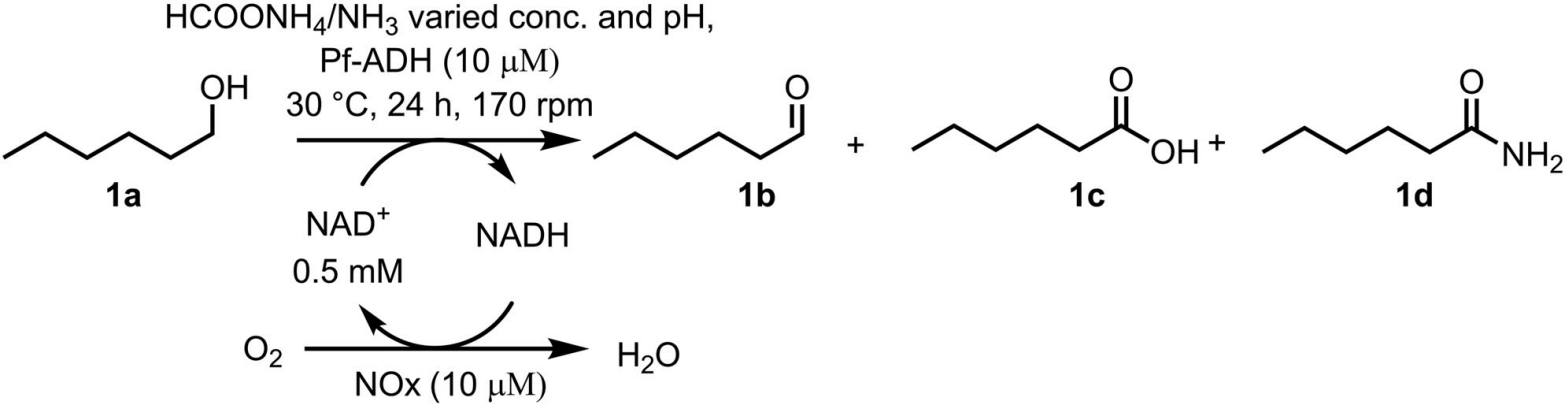
Oxidative coupling of an alcohol with an amine to yield an amide catalyzed by Pf‐ADH in ammonium formate buffer. Reaction conditions: ADH (10 µM), NOx (10 µM), and NAD^+^ (0.05 mM) at 30 °C, 170 rpm.

The ADH from *Pichia finlandica* (Pf‐ADH), which was studied in our lab due to its antiPrelog stereoselectivity in the reduction of ketones,^[^ ^79^ ^]^ was initially tested for the oxidation of *n*‐hexanol (**1a**) to yield the corresponding amide (**1d**). A catalytic amount of NAD^+^ was added and internally recycled using a nicotinamide adenine dinucleotide oxidase (NOx) and dioxygen from air as a sacrificial cosubstrate (Figure 2).^[^ ^80^ ^]^ Both enzymes were prepared by overexpression in *E. coli* BL21(DE3) cells and used in purified form (see Supporting Information Section 6). We conducted the first tests in ammonium formate buffer (100 mM, pH 9) with **1a** (10 mM), (Figure 2). Traces of the desired product (**1d**), along with aldehyde (**1b**) and the corresponding carboxylic acid (**1c**), showed the feasibility of the approach. Therefore, various sets of experiments were performed to optimize the reaction conditions in terms of buffer concentration (Supporting Information, Table S2), pH (Supporting Information, Table S4 and Figure S3), and the concentrations of the enzymes ADH and NOx (SI, Table S3) for amide formation from alcohol **1a**. Using Pf‐ADH, optimal reaction conditions were identified at pH 10, with 1 to 5 M NH_3_/NH_4_⁺ and 10 µM of both ADH and NOx, resulting in 65%, 85%, and 96% conversion to amide **1d**. (Table S5 and Figure S4). Notably, the amount of carboxylic acid by‐product **1c** was reduced to as little as 4%. Subsequently, we investigated whether other ADHs besides Pf‐ADH, which has antiPrelog selectivity and NAD‐dependency, could have similar promiscuous activity. The following ADHs in our lab possessing Prelog or antiPrelog selectivity and NAD or NADP‐dependency were tested: *Candida maris* (Cm‐ADH),^[^ ^79^ ^]^ *Bacillus subtilis* (Bs‐BDHA),^[^ ^81^, ^82^, ^83^ ^]^ *Aromatoleum aromaticum* (Aa‐ADH),^[^ ^84^, ^85^ ^]^ *Paracoccus pantotrophus* (Pp‐ADH),^[^ ^86^ ^]^ *Thermoanaerobacter ethanolicus* variant W110A (Te‐ADH^W110A^),^[^ ^87^ ^]^ and *Bacillus stearothermophilus* (Ht‐ADH)^[^ ^85^, ^88^ ^]^ (see Table S1 for details). Since the inherent stability of these ADHs at high ammonium buffer concentration was unknown, we tested the enzymes for conversion of **1a** at 1 M NH_3_/NH_4_^+^ species concentration in the pH range from 8.5 to 13. Except for Cm‐ADH and Bs‐BDHA, the other ADHs exhibited the same catalytic promiscuity as Pf‐ADH. Aa‐ADH worked the best at pH 10.5, Pp‐ADH at pH 10 while Te‐ADH^W110A^ and Ht‐ADH gave the best results at pH 9.5 (Tables S7, S10, S13 and S16). Next, the ADHs were tested at different buffer concentrations. Similar to Pf‐ADH, Pp‐ADH yielded higher amide **1d** concentrations at higher buffer concentrations (81%–95% amide in the range from 2 to 5 M; Table S11). In contrast, the conversion to **1d** worsened at high buffer concentrations with Aa‐ADH, Te‐ADH^W110A^, and Ht‐ADH (Tables S8, S14, and S17), probably due to lower stability under these conditions. Therefore, the best compromise between conversion to **1d** and enzyme stability was generally set around 1 M NH_3_/NH_4_^+^ buffer. These conditions led to 46% of **1d** with Aa‐ADH, 16% for Te‐ADH^W110A^, and 10% for Ht‐ADH. Finally, we investigated the influence of temperature on the reaction between 20 and 35 °C (Tables S6, S9, S12, S15, and S18). While Te‐ADH^W110A^, Pp‐ADH and Aa‐ADH showed their best performance at 30 °C, Pf‐ADH and Ht‐ADH yielded higher conversions to the amide at 25 °C.

**Figure 3:**
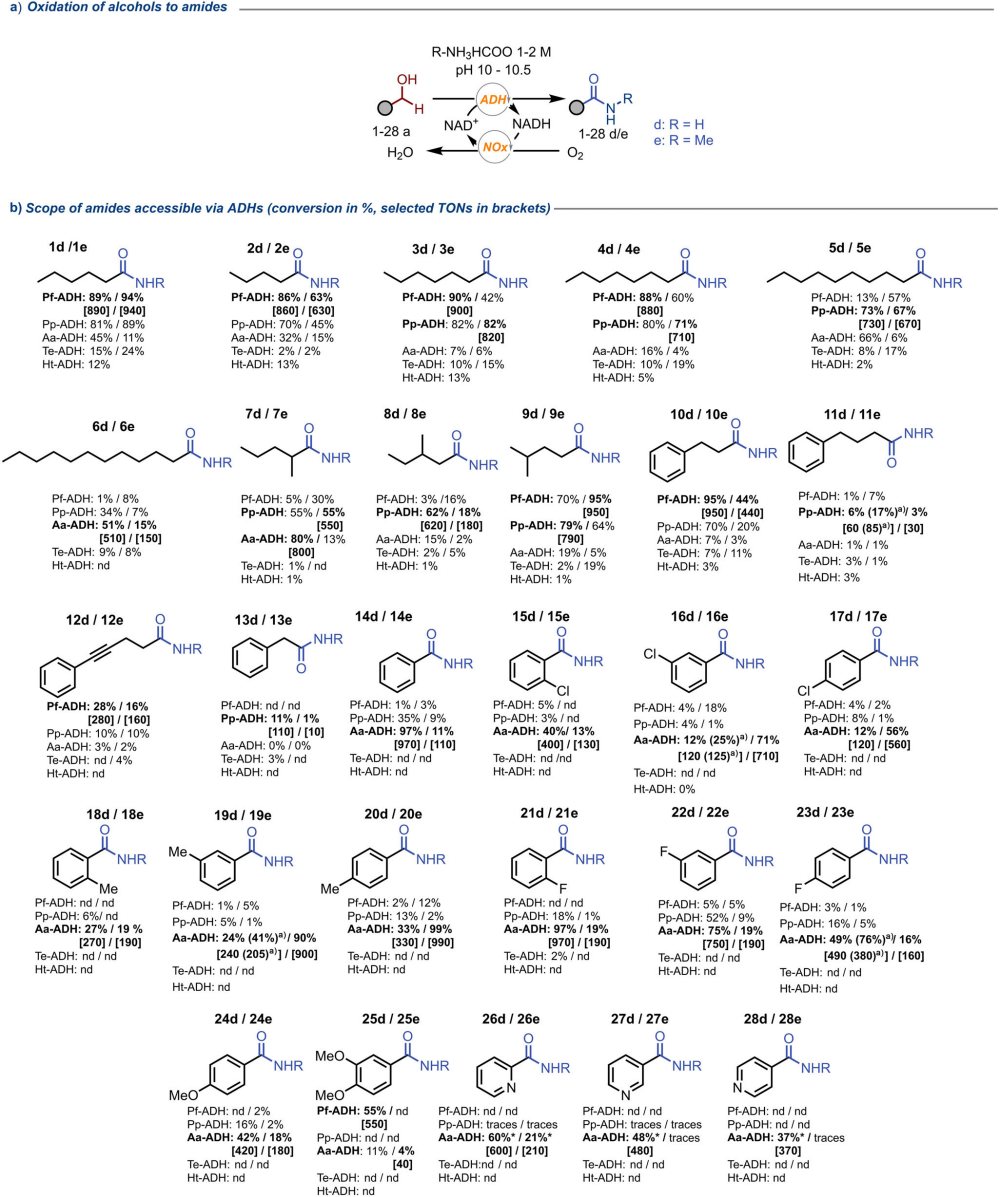
The substrate scope for the oxidative coupling of alcohols (**1–28a**) with ammonia to give primary amides (R = H, **1–28d**) or with methylamine to give secondary amides (R = CH_3_, **1–28e**) catalyzed by ADHs. The conversion reported is the average obtained by three independent measurements. [*] For substrates **26a**, **27a**, and **28a**, the reported values are isolated yields for reactions at 100 mg‐scale (for details see Supporting Information Section 7). nd; not detected under the assay conditions. Reaction performed in amine buffer pH 10 or 10.5 for Aa‐ADH, 1 M or 2 M for Pf‐ and Pp‐ADH, 0.1 mol% ADH, 0.1 mol% NOx, substrate 10 mM, NAD^+^ 0.05 mM, unless otherwise stated. Reactions were performed at 30 °C at 800 rpm in 1 mL volume. ^a)^ Reactions with 0.2 mol% of ADH. The analysis was performed with GC‐FID with an Agilent J&W DB‐1701 column (30 m) using toluene as internal standard (10 mM).

To investigate the synthetic applicability of our method, we tested the reaction under optimized conditions on a broad panel of substrates (Figure 3). Initially, we screened linear short‐chain aliphatic alcohols. Pf‐ADH outperformed the other ADHs, yielding conversions between 86% and 90% starting from *n*‐pentanol (**2a**), *n*‐hexanol (**1a**), *n*‐heptanol (**3a**), and *n*‐octanol (**4a**). Conversely, with increasing chain length using *n*‐decanol (**5a**) and *n*‐dodecanol (**6a**), Pp‐ADH and Aa‐ADH gave the best results with the highest amide conversion of 73% for **5a** and 51% for **6a**. Next, three branched hexanols were tested (**7–9a**) yielding highest conversions between 62% and 80%. Interestingly, the ADHs behaved differently depending on the position of the methyl group with respect to the hydroxyl. Aa‐ADH preferentially accepted substrates with the methyl group closer to the alcohol moiety (**7a**). In contrast, Pf‐ADH was more sensitive to steric hindrance close to the hydroxyl group, thus better accepting alcohol **9a**. Pp‐ADH activity was less affected by the position of the methyl substituent. Subsequently, we tested alkyl aryl alcohols (**10–13a**) and benzyl alcohols (**14–25a**), yielding the respective amide product with moderate to excellent conversion. For alkyl aryl alcohols, Pf‐ADH and Pp‐ADH afforded the highest conversions from 6% to 95%. In contrast, Aa‐ADH consistently afforded the highest conversions with benzyl alcohols (from 12% to 97%) with the only exception being the more sterically demanding substrate **25a**, which was better accepted by Pf‐ADH (55% conversion). The fluoro‐substituted alcohols gave the highest conversions among all substituted benzyl alcohols, suggesting that the steric effect of the substituent has a greater influence than its electronic.

To complete the scope, hydroxymethyl pyridines were tested giving relevant conversions only with Aa‐ADH. The capability of Aa‐ADH to convert benzylic alcohols and hydroxymethyl pyridines correlates with the high activity of this enzyme for the reduction of acetophenone and derivatives thereof, as described in the literature.^[^ ^84^ ^]^ We point out that the substrate scope was investigated mainly using 10 mM alcohol substrate and 10 µM ADH, corresponding to an enzyme loading of 0.1 mol%. Even in cases where the conversion was low (ca. 10%) or moderate (<50%), the calculated turnover numbers (TONs) still ranged from approximately 100 to 500, generally underscoring the immediate synthetic applicability of these biotransformations.

Next, we decided to test if our enzymatic methodology could be extended to the synthesis of N‐substituted amides using **1a** as the test alcohol substrate and formate buffer salts derived from different amine donors (see Supporting Information Section 7.6 for details). Only the reaction conducted using methylamine as the donor led to the corresponding N‐methyl substituted amide (**1e**) with Pf‐ADH, Pp‐ADH, Aa‐ADH, and Te‐ADH^W110A^. In contrast, Ht‐ADH could not synthesize the amide using methylamine. Thus, the oxidative coupling with all the alcohols from this study (**1–28a**) was performed with MeNH_2_/MeNH_3_^+^ buffer (Figure 3), giving the same trend as observed with NH_3_ as the amino donor with some exceptions for aliphatic and one benzylic substrate (**3–4a**, **6–7a**, **9a**, **11a**, **13a**, and **25a**).

A preparative scale synthesis was performed with *n*‐hexanol (**1a**, 500 mg, 4.89 mmol, and 20 mM) using Pf‐ADH (cell‐free extract equivalent to ca. 20 µM of pure enzyme; for details see Supporting Information Table S28, S29) in MeNH_2_/MeNH_3_^+^ buffer (2 M, pH 10). The reaction was incubated for 5 h, at 170 rpm and 25 °C. Pf‐ADH yielded >99% conversion of **1a** into *N*‐methylhexanamide (**1e**) and, after work‐up, **1e** was isolated in 87% yield. Based on these data and considering the isolated yield of 87%, the productivity metrics of the scale‐up were a chemical turnover number (TON) of 871, a turnover frequency (TOF) of 174 h^−1^, and a space‐time yield (STY) of 433 mg·L^−1^·h^−1^.

Notably, in this 500 mg‐scale biotransformation, we observed an influence of the ADH preparation on reaction stability. When Pf‐ADH was used as a crude lysate, the conversion to the product was >99% at both the 500 mg scale and the analytical scale (1 mL). In contrast, when Pf‐ADH was used in purified form, the conversion was 70% and 94% at the 500 mg scale and analytical scale, respectively. This behavior has already been observed in other applications of enzymes for organic synthesis as other proteins and/or biomolecules present in a crude lysate can have a protective action on the overexpressed biocatalyst.

**Figure 4:**
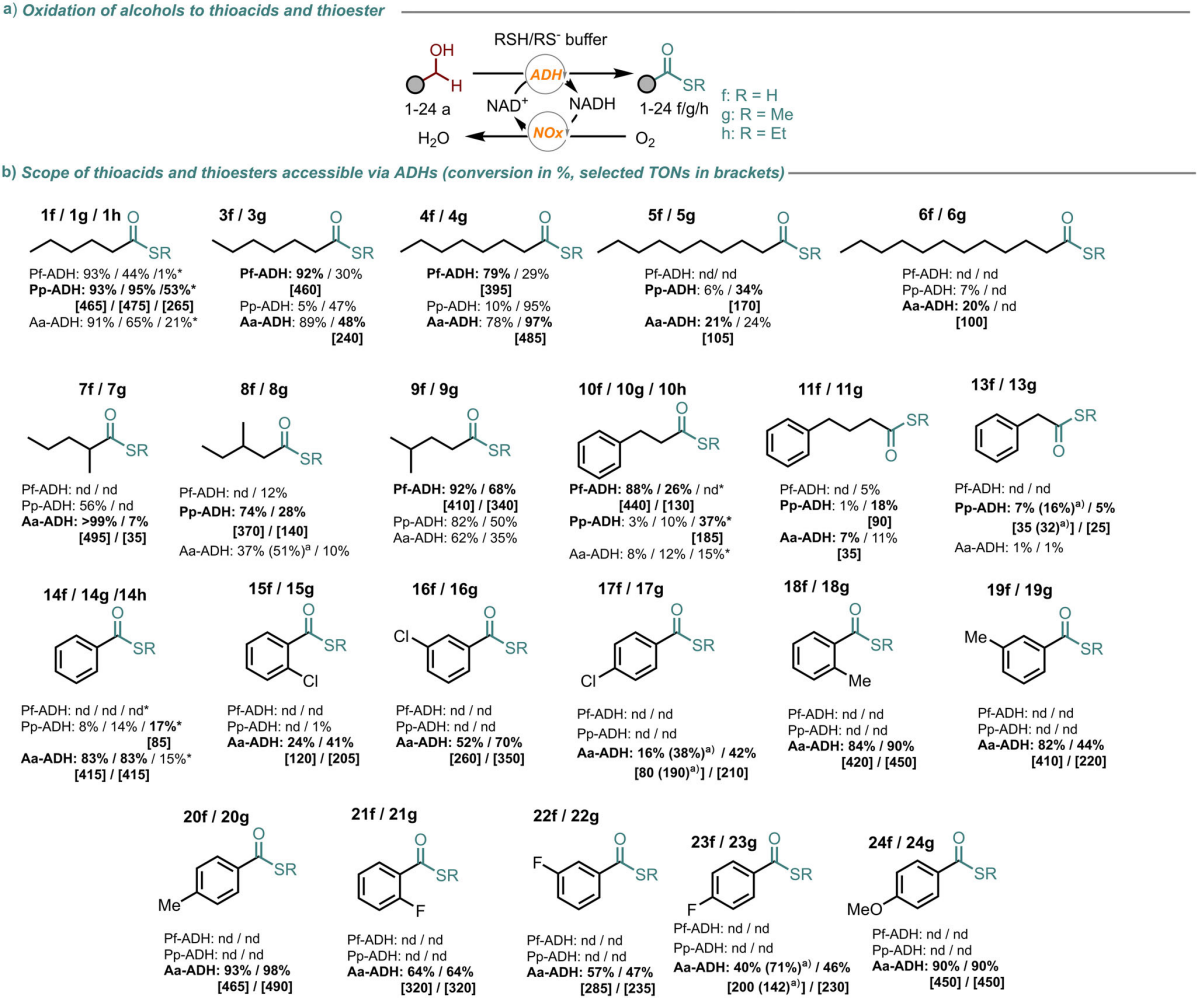
Substrate scope for the oxidative coupling of alcohols (**1–24a**) with hydrogen sulfide (H_2_S/HS^−^) to afford thioacids (R = H, **1–24f**), and with alkyl thiols to afford thioesters (R = CH_3_, **1–24 g** and R = CH_2_CH_3_, **1h**, **10h,** and **14h**), catalyzed by ADHs. The reported conversions are the averages of three independent measurements. Reaction performed in the thiol buffer pH 7, substrate 10 mM, NAD^+^ 0.5 mM, ADH, and NOx 0.2 mol% unless otherwise stated. The concentration of HS^−^ was 0.3 M in all cases. The concentration of MeS^−^ was 1 M for the reactions with Pf‐ADH and Pp‐ADH and 0.3 M for the reactions with Aa‐ADH. *In these cases, the reactions were performed in a biphasic system consisting of KPi (500 mM, pH 7) and heptane (1:1 v/v), with EtSH 0.5 M. Reactions were performed at 30 °C at 800 rpm in 1 mL volume.^a)^ Reaction performed with 0.5 mol% of ADH. The analysis was performed with GC‐FID with an Agilent J&W HP‐5 column (30 m) using toluene as internal standard (10 mM).

With robust conditions for amide formation established, we next investigated whether the same biocatalytic strategy could be applied to thioester synthesis using thiols as nucleophiles. In principle, ADHs can oxidize primary alcohols to aldehydes in the presence of thiols, forming thiohemiacetals in situ, which can then be oxidized to thioesters. To validate this hypothesis, we tested the ADHs that were found more active for amide formation (i.e., Pf‐ADH, Pp‐ADH, and Aa‐ADH) for the oxidation of 1‐hexanol (**1a**) in a H_2_S/HS^−^ buffer to yield the corresponding thioacid (Figure 4, products **1–24f**). The reaction used **1a** (10 mM) in the H_2_S/HS^−^ buffer (50 mM pH 10) with a catalytic NAD^+^ (0.5 mM), ADH (20 µM), and NOx (20 µM). Under these conditions, Pf‐ADH, Pp‐ADH, and Aa‐ADH gave only trace amounts of thioacid, while other ADHs stopped at the aldehyde (data not shown). Increasing the buffer concentration to 300 mM resulted in complete conversion of **1a**, yielding a mixture of carboxylic acid (**1c**) and thioacid (**1f**), with the former predominating (Tables S30, S34, and S38).

To improve selectivity, we screened pH values from 6.5 to 12.0. The highest thioacid selectivity with complete substrate conversion was observed at pH 7.0 (Tables S33, S37, and S41), likely due to the lower pK_a_ of H_2_S compared to water. At pH 7, the higher HS^−^/OH^−^ ratio enhances nucleophilic attack by HS^−^ and favors thioacid formation. At lower pH values (6.0–6.5), reduced enzyme activity leads to lower conversion, confirming pH 7 as the best compromise between enzyme activity and nucleophile availability. Building on the successful thioacid synthesis from **1a**, we evaluated the scope to a range of primary alcohols, including long‐chain aliphatic, branched, aryl, and benzyl ones. Pf‐ADH and Pp‐ADH performed best with aliphatic substrates, whereas Aa‐ADH showed superior activity with benzyl alcohol derivatives (Figure 4, products **1f‐24f**).

Next, we tested these ADHs for thioester synthesis via oxidative coupling of alcohols with methylthiol (MeSH) in MeSH‐containing buffer at pH 7. Pf‐ADH and Pp‐ADH required 1 M MeSH concentration, while Aa‐ADH tolerated only 0.3 M MeSH. Remarkably, all three enzymes successfully catalyzed methyl thioester formation (Figure 4, products **1–24 g**).

Unfortunately, longer thiols were not accepted due to poor solubility in water. To address this, reactions with ethanethiol (EtSH) were conducted in a biphasic KPi buffer/heptane system. Lower ionic strength of the aqueous phase favored carboxylic acid formation, whereas higher ionic strength shifted the selectivity toward the desired thioester formation. Longer thiols gave no conversion, as also observed in the analogous amide‐forming reactions. The only exceptions were reactions with substrates **1a**, **10a** and **14a**, which yielded products **1h**, **10h,** and **14h** using EtSH as nucleophile (Figure 4).

Therefore, to broaden the substrate scope and improve reactivity in amide and thioester formation, we engineered Aa‐ADH, selected due to the availability of its X‐ray crystal structure (PDB: 2EWM), thereby enabling reliable docking studies (see Supporting Information Section 13).

**Figure 5:**
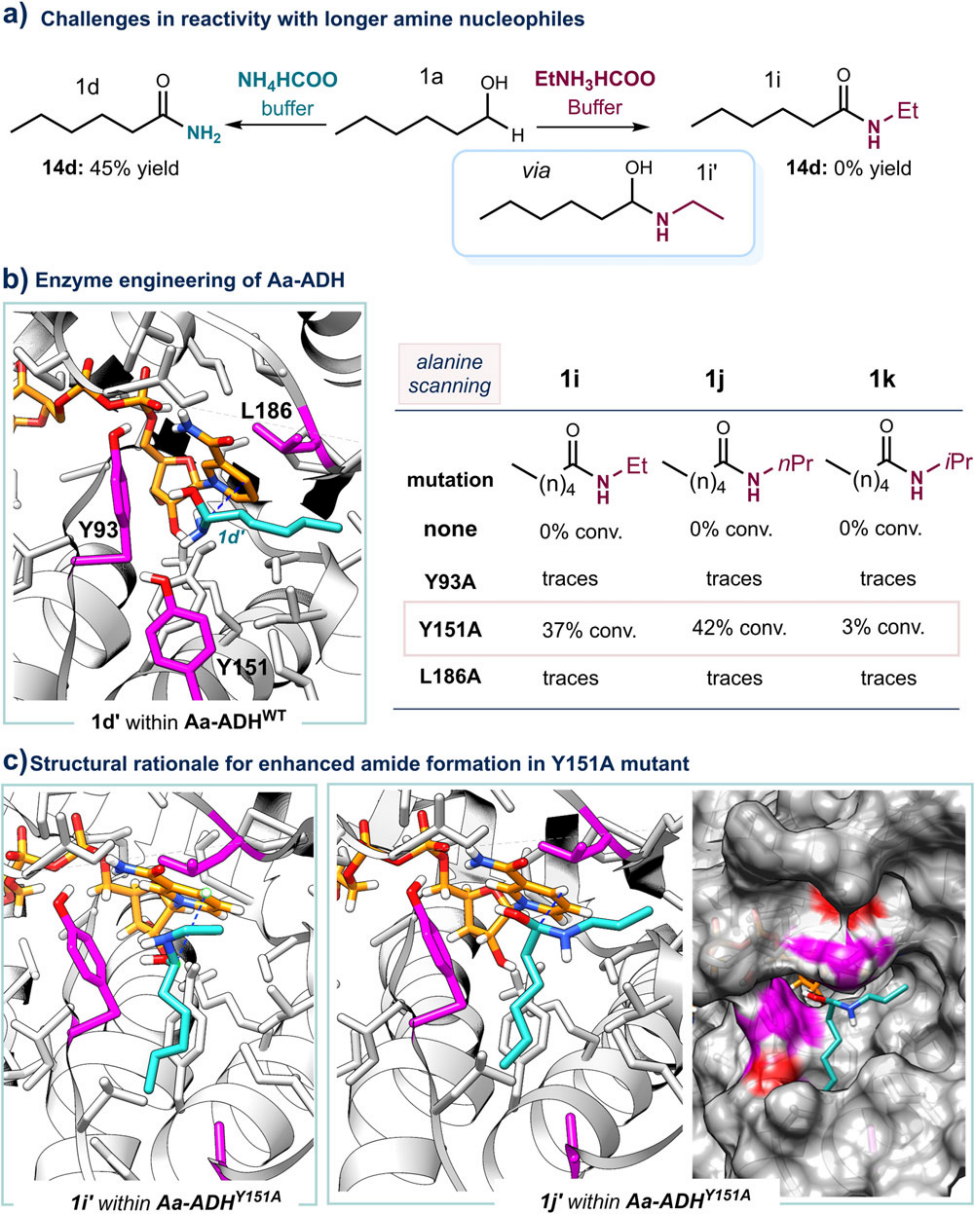
a) Challenges in amide synthesis with longer‐chain amine nucleophiles. b) Enzyme engineering strategy to expand the reactivity profile of Aa‐ADH. c) Molecular docking studies to investigate the increased reactivity profile of Aa‐ADH^Y151A^. Dockings performed with YASARA structure; UCSF Chimera software was used for visualization.

As a model substrate for the docking studies, we selected alcohol **1a** (Figure 5a) to better explore the potential for expanding reactivity toward more challenging substrates. Additionally, the flexibility of the alkyl chain in **1a** allowed us to identify alternative binding poses that could inform the design of more effective enzyme variants. Notably, **1a** also proved to be one of the most reactive substrates for the thioacid formation. Docking of the reactive hemiaminal intermediate **1d’** within the active site of Aa‐ADH (Figure 5b) revealed that residues Y93, Y151, and L186 are positioned around **1d’** and may influence substrate orientation and reactivity. To evaluate their role, we performed an alanine‐scanning mutagenesis, targeting each of these positions individually. While the wild‐type enzyme showed negligible conversion with longer‐chain amine nucleophiles such as ethylamine or *n*‐propylamine, the Aa‐ADH^Y151A^ mutant retained activity across all tested amines, achieving a 42% conversion with the more sterically demanding *n*‐propylamine. This suggests that Y151 plays a critical role in modulating the spatial constraints of the binding pocket, likely relieving steric hindrance that limits productive binding of longer‐chain amines. In contrast, Aa‐ADH^Y93A^ and Aa‐ADH^L186A^ showed only minimal activity, indicating that although these residues are proximal to the substrate, their mutation does not significantly improve access or stabilize the transition state for these nucleophiles. Overall, these results highlight Y151 as a promising engineering hotspot for expanding the substrate scope of Aa‐ADH, particularly toward longer‐chain amines. Unfortunately, the aromatic alcohol **14a** remained unreactive in the oxidative coupling with these bulkier amines (see Supporting Information, Section 7.12), including *n*‐propylamine and isopropylamine, and afforded only 2% conversion with ethylamine. A rational explanation for the low reactivity of substrate **14a** with ethylamine is provided by molecular docking studies (see Supporting Information, Section 13.2 and Figure S6), which revealed a nonproductive binding pose with an unfavorable hydride transfer distance in the Aa‐ADH^Y151A^ mutant. Interestingly, substrate **10a**, which contains a less sterically hindered aromatic moiety, showed slightly improved reactivity, yielding 6% and 3% of the corresponding amides with ethylamine and *n*‐propylamine, respectively (Supporting Information, Section 7.12).

To understand the improved activity of the Aa‐ADH^Y151A^ mutant, we performed docking studies using the hemiaminal intermediate formed from **1a** with ethylamine (**1i’**) or *n*‐propylamine (**1j’**, Figure 5c). The results showed that the Y151A mutation generates additional space in the active site, allowing accommodation of the longer alkyl chains. Interestingly, the ethyl or propyl substituent now occupies the region previously occupied by the alkyl tail of **1a** in the wild‐type enzyme (compare dockings in Figure 5b,c). This shift in binding orientation highlights the role of Y151 in restricting access for bulkier nucleophiles and demonstrates how its mutation into alanine creates a cavity that permits alternative substrate poses, facilitating efficient catalysis with longer‐chain amines.

**Figure 6:**
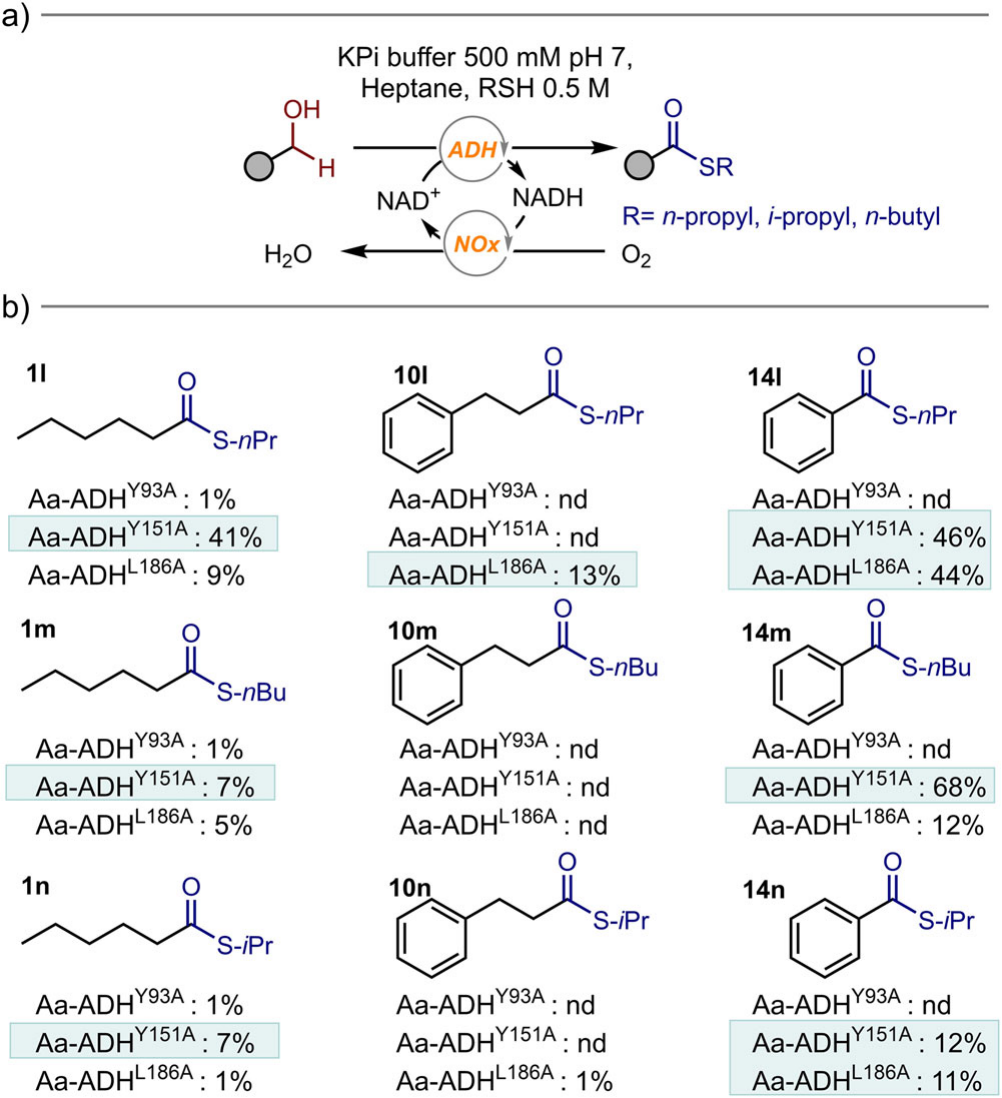
a) General scheme of the biocatalytic reaction for thioester formation. b) Activity profile of Aa‐ADH variants in thioester formation. The reported conversions are the averages of three independent measurements. Reaction performed in a biphasic system consisting of KPi (500 mM, pH 7) and heptane (1:1 v/v), with RSH 0.5 M. Reactions were performed at 30 °C at 800 rpm in 1 mL volume. The analysis was performed with GC‐FID with an Agilent J&W HP‐5 column (30 m) using toluene as internal standard (10 mM).

Encouraged by the performance of the Aa‐ADH^Y151A^ variant with challenging amines, we next explored its potential in reactions involving thiol nucleophiles to assess whether similar enhancements could be observed in thioester formation. The wild‐type enzyme and the Y93A variant showed minimal to no activity across the panel of thiols tested. In contrast, the Aa‐ADH^Y151A^ and Aa‐ADH^L186A^ mutants displayed improved reactivity, particularly with linear alkyl thiols (Figure 6). For instance, with *n*‐propylthiol, the Aa‐ADH^Y151A^ mutant afforded **1l** with 41% conversion starting from **1a** as substrate and 46% conversion for **14l** using **14a**. The same variant also showed high activity with the longer *n*‐butylthiol, reaching 68% conversion for **14m** starting from **14a**, that showed no activity in the amidation reaction. Finally, for the more sterically demanding *i*‐propylthiol, the Aa‐ADH^Y151A^ showed measurable levels of activity with both **1a** and 1**4a** (7% for **1n** and 12% for **14n**). Interestingly, while the bulky substrate **10a** remained unreactive using the Aa‐ADH^Y151A^, it showed modest conversion with Aa‐ADH^L186A^ variant using *n*‐propylthiol as reaction partner (13% for **10l**). This variant also performed well with substrate **14a**, yielding the corresponding thioesters **14l** and **14n** with 44% and 11% conversion using *n*‐propyl‐ and *i*‐propylthiol, respectively. These results highlight L186A as a complementary and valuable mutation for expanding the enzyme's substrate scope, especially with sterically hindered alcohols such as **10a** that are poorly accepted by other variants.

Finally, we conducted a series of experiments to elucidate the reaction mechanism (see Supporting Information Section 7.8 for details), using the amidation as model reaction. These data confirmed that the amide product is formed via the ADH‐catalyzed oxidation of the hemiaminal intermediates. Removing ADH from the reaction mixture led to no amide formation. Notably, both alcohol and aldehyde could serve as initial substrates for the reaction. We also demonstrated that in ammonium free buffer, the presence of the ADH is necessary for the formation of the carboxylic acid via oxidation of the hydrate of the aldehyde. We believe that these mechanistic findings are extendable to other accepted nucleophiles such sulfur donors like hydrogen sulfide and thiols. It is also interesting to note that a similar mechanism for amide synthesis, via coupling of alcohols and amines through a hemiaminal intermediate, has been reported in homogeneous catalysis using Ru(II) catalysts.^[^ ^89^ ^]^ Our group also discovered a catalytic promiscuous activity in a variant of galactose oxidase from *Fusarium* sp. (GOx M3‐5), which was capable of converting alcohols into nitriles using ammonia and atmospheric oxygen, also via a hemiaminal intermediate.^[^ ^90^ ^]^

## Conclusion

In summary, by harnessing the promiscuous activity of certain ADHs, we established a new approach for the sustainable synthesis of primary and secondary amides, thioacids, and thioesters. Our strategy enables achieving good to excellent conversions across a wide range of structurally diverse alcohol substrates. Notably, we demonstrated that the method's applicability can be extended to longer‐chain or bulkier nucleophiles through structure‐guided enzyme engineering. Furthermore, by fine‐tuning the reaction conditions, we could favor thioester formation over competing oxidation pathways. Moreover, active‐site engineering, particularly by introducing the Y151A and L186A mutations in Aa‐ADH, enabled efficient coupling with more sterically demanding alkyl thiols and longer‐chain amines, illustrating how targeted mutagenesis can broaden substrate compatibility. Finally, scalability was exemplified through the oxidative coupling of 500 mg *n*‐hexanol with methylamine, achieving quantitative conversion and highlighting the practical utility of the method in organic synthesis.

Collectively, our findings deliver a versatile biocatalytic platform for the sustainable synthesis of both amides and thioesters. This work exemplifies the powerful synergy between enzyme promiscuity, reaction condition control, and protein engineering and, once again, highlights the importance of exploring enzyme promiscuity to uncover new catalytic activities of value to humanity.

## Supporting Information

Supporting Information includes: materials and methods (Section 3); lists of substrates and enzymes (Sections 4 and 5); procedures for enzyme preparation (Section 6); studies on amide formation with all ADHs, screening of amine donors, time‐course analysis, control experiments for reaction mechanisms, and preparative‐scale synthesis (Section 7); studies on thioacid and thioester formation and screening of different thiol donors (Sections 8–11); details of substrate scope (Section 12); computational docking studies (Section 13); site‐directed mutagenesis (Section 14); and analytical methods, including NMR spectra and chromatograms (Section 15). The authors have cited additional references within the Supporting Information.^[^ ^12^, ^13^, ^14^, ^15^, ^16^, ^17^, ^18^, ^19^ ^]^

## Conflict of Interests

The authors declare no conflict of interest.

## Supporting information



Supporting Information

## Data Availability

The data that support the findings of this study are available in the Supporting Information of this article.
