# Supplementary material for: Amide and Thioester Synthesis Via Oxidative Coupling of Alcohols with Amines or Thiols Using Alcohol Dehydrogenases
Source: Angew Chem Int Ed Engl. 2025 Sep 30;65(1):e202515469. doi: 10.1002/anie.202515469 (PMC12759251; doi:10.1002/anie.202515469)
Supplement: Supplementary file 1 — Supporting Information [file ANIE-65-e202515469-s001.pdf]

Supporting information for:

Amide and Thioester Synthesis via Oxidative Coupling of Alcohols with Amines or Thiols Using Alcohol Dehydrogenases

Matteo Damian, Vasilis Tseliou, Patrick Peters, Tanja Knaus, Francesco G. Mutti *

Van ’t Hoff Institute for Molecular Sciences, HIMS-Biocat, University of Amsterdam, Science Park 904, 1098 XH Amsterdam, The Netherlands

* Corresponding author: f.mutti@uva.nl

1. Contents

2. List of abbreviations 4
3. Material and methods 4
4. List of substrates 4
5. List of enzymes 5
6. General procedure for enzymes expression and purification 5
7. Amidation study 6
7.1. Reaction optimization with *Pichia finlandica* ADH 6
7.1.1. Study at varied buffer concentration at pH 9 6
7.1.2. Study at varied enzyme concentration 7
7.1.3. Study at varied pH 7
7.1.4. Study at varied buffer concentration at pH 10 8
7.1.5. Study at varied temperature 9
7.2. Reaction optimization with Aa-ADH 10
7.2.1. Study at varied pH 10
7.2.2. Study at varied buffer concentration at pH 10.5 10
7.2.3. Study at varied temperature 11
7.3. Reaction optimization with Pp-ADH 12
7.3.1. Study at varied pH 12
7.3.2. Study at varied buffer concentration at pH 10 12
7.3.3. Study at varied temperature 13
7.4. Reaction optimization with Te-ADH W110A 14
7.4.1. Study at varied pH 14
7.4.2. Study at varied buffer concentration at pH 9.5 14
7.4.3. Study at varied temperature 15
7.5. Reaction optimization with Ht-ADH 16
7.5.1. Study at varied pH 16
7.5.2. Study at varied buffer concentration at pH 9.5 16
7.5.3. Study at varied temperature 17

7.6. Different amine buffers ... 18
7.7. Time study ... 19
7.7.1. Pf-ADH and NH_3 ... 19
7.7.2. Pf-ADH and $MeNH_2$ ... 19
7.7.3. Pp-ADH and NH_3 ... 20
7.7.4. Pp-ADH and $MeNH_2$ ... 21
7.7.5. AA-ADH and NH_3 ... 21
7.7.6. AA-ADH and $MeNH_2$ ... 22
7.7.7. AA-ADH and NH_3 with substrate 14a ... 23
7.7.8. AA-ADH and $MeNH_2$ with substrate 20a ... 23
7.8. Control experiments to verify catalytic promiscuity and mechanism ... 25
7.9. Preparative scale synthesis of N-methylhexanamide ... 26
7.10. Reaction with substrate 26-28a ... 28
7.11. Quantitative determination of carboxylic acid ... 28
7.12. Longer chain amines screening ... 28
8. Thioacids synthesis ... 29
8.1. Reaction optimization with *Pichia finlandica* ADH ... 29
8.1.1. Buffer concentration pH 10 ... 29
8.1.2. pH study 1 M buffer ... 30
8.1.3. Concentration buffer pH 7 ... 30
8.1.4. pH study 0.3 M buffer ... 31
8.2. Reaction optimization with *Paracoccus pantotrophus ADH* ... 31
8.2.1. Buffer concentration pH 10 ... 31
8.2.2. pH study 1 M buffer ... 32
8.2.3. Concentration buffer pH 7 ... 33
8.2.4. pH study 0.3 M buffer ... 33
8.3. Reaction optimization with Aromatoleum aromaticum ADH ... 34
8.3.1. Buffer concentration pH 10 ... 34
8.3.2. pH study 1 M buffer ... 34
8.3.3. Concentration buffer pH 7 ... 35
8.3.4. pH study 0.3 M buffer ... 36
9. Methylthioester synthesis ... 37
9.1. Pf-ADH ... 37
9.1.1. Buffer concentration pH 7 ... 37
9.2. Pp-ADH ... 37
9.2.1. Buffer concentration pH 7 ... 37
9.3. Aa-ADH ... 38
9.3.1. Buffer concentration ... 38
10. Thioesters (EtSH) ... 39
10.1. Pf-ADH ... 39

10.2. Pp-ADH 39
10.3. Aa-ADH 40
11. Longer chain thiols screening 41
12. Substrate scope 42
12.1. Amides 42
12.2. Thioacids and thioesters 56
13. Computational studies 68
13.1. General procedure of docking 68
13.2. Docking experiment to rationalize the lack of reactivity 68
14. Site-directed mutagenesis procedure 70
15. Analytical methods and GC chromatograms 71
16. References 121

2. List of abbreviations

ADH	Alcohol dehydrogenase
NOx	Nicotinamide adenine dinucleotide oxidase
$NAD(P)^+$	Nicotinamide adenine dinucleotide phosphate (oxidized form)
GC-FID	Gas chromatography with flame ionization detector
GC-MS	Gas chromatography coupled with mass spectrometry
ATP	Adenosine triphosphate
CoA	Coenzyme A
GOx	Galactose oxidase
E. coli	*Escherichia coli*

3. Material and methods

Nicotinamide adenine dinucleotide cofactor (NAD^+) was purchased from Melford Biolaboratories (Chelsworth, Ipswich, UK).
The conversions for the reduction of the ketones were determined by GC using a 7890A GC system (Agilent Technologies), equipped with an FID detector using H_2 as carrier gas and a DB-1701 column from Agilent (30 m, 250 µm, 0.25 µm).
^{1}H (400 MHz) spectra were recorded at ambient temperature using a Bruker AV400. ^{1}H NMR spectra are reported in parts per million (ppm) downfield relative to $CDCl_3$ (7.26 ppm). NMR data were processed using the MestReNova 14.1.0 software package.

4. List of substrates

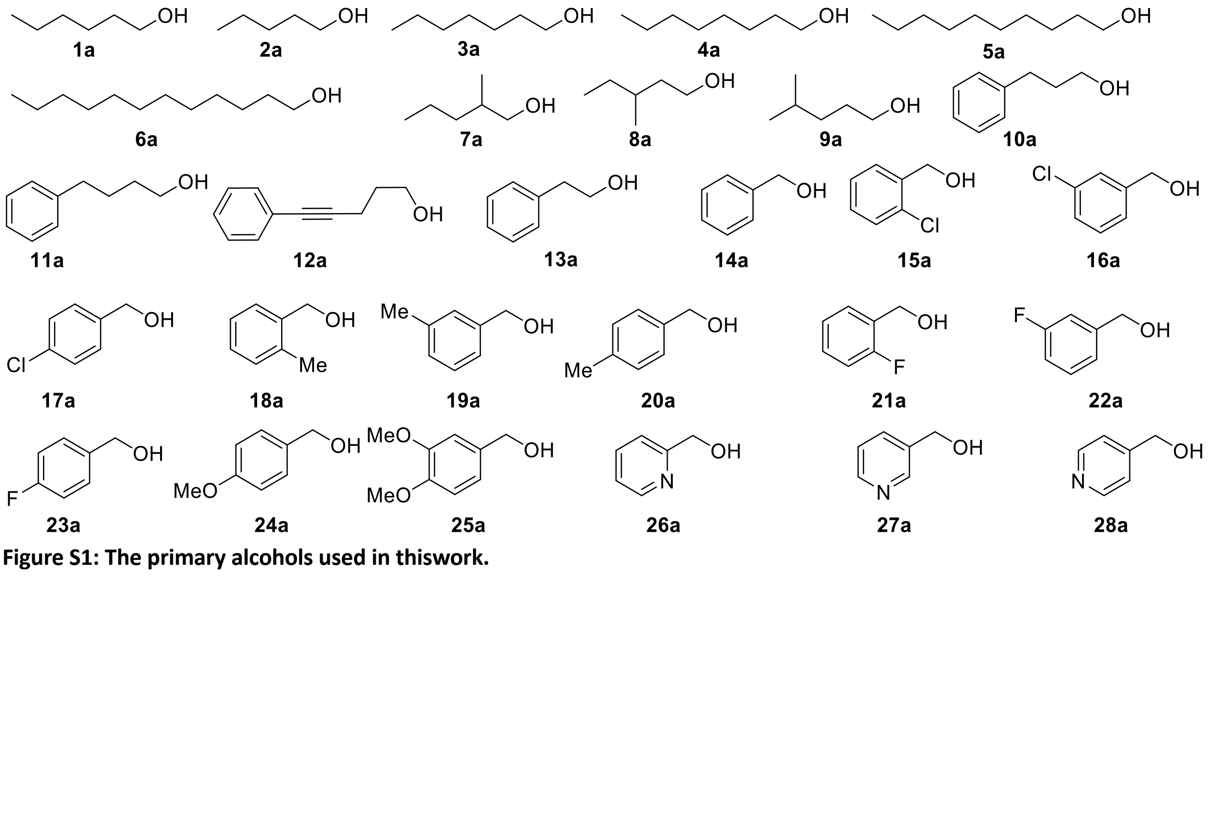

Figure S1: The primary alcohols used in thiswork.

5. List of enzymes

Table S1: Enzymes used in this work.

Abbreviation	Source	Selectivity	Cofactor	Used form	Ref
Pf-ADH	ADH from *Pichia finlandica*	Anti-Prelog	NAD	Purified and crude lysate	[1]
Aa-ADH	ADH from *Aromatoleum aromaticum*	Prelog	NAD	Purified and crude lysate	[2, 3]
Pp-ADH	ADH from *Paracoccus pantotrophus* DSM 11072	Prelog	NAD	Purified	[4]
Ht-ADH	ADH from *Bacillus stearothermophilus*	Primary alcohols	NAD	Purified	[3, 5]
Te-ADH W110A	ADH from *Thermoanaerobacter ethanolicus* variant W110A	Prelog	NADP	Purified	[6]
Cm-ADH	ADH from *Candida maris*	Anti-Prelog	NAD	Purified	[1]
Bs-BDHA	ADH from *Bacillus subtilis*	Anti-Prelog	NAD	Purified	[7]
NOx	NOx from *Streptococcus mutans*	n. a.	n. a.	Purified	[8]

6. General procedure for enzymes expression and purification

Enzyme expression

For recombinant expression, 800 mL of LB medium supplemented with the appropriate antibiotic (100 µg mL^{-1} ampicillin or 50 µg mL^{-1} kanamycin) were inoculated with 15 mL of an overnight culture harboring the desired vector with genes for the expression of the enzyme. *E. coli* BL21(DE3) cells were used as expression host organism in this study. Cells were grown at 37 °C until an OD_{600} in the range from 0.6 to 1 was reached and expression of protein was induced by the addition of IPTG. Protein expression was carried out overnight and after harvesting of the cells (4 °C, 4500 rpm, 10 min), the remaining cell pellets were washed with buffer. For the preparation of lyophilized *E. coli* whole cells, we used: 50 mM KPi buffer at pH 8.0 for ADHs and NOx. For the preparation of the cell lysate for further enzyme purification by affinity chromatography, we used lysis buffer as reported in the next paragraph.

Purification by Nickel ion affinity chromatography

His_6-tagged enzymes were resuspended in lysis buffer (50 mM KH_2PO_4, 300 mM NaCl, 10 mM imidazole, pH 8.0, 5 mL per 1 g of wet cells) prior to cell disruption via sonication (10 mins, pulse 10 s ON, pulse 10 s OFF, 45% amp). In the case of NOx, before the disruption of cells containing overexpressed enzyme, a tiny amount of FAD was externally added. Protein purification was performed by Ni-NTA affinity chromatography using pre-packed Ni-NTA HisTrap HP columns (GE Healthcare), previously equilibrated with lysis buffer. After loading of the filtered lysate, the column was washed with sufficient amounts of wash buffer (50 mM KH_2PO_4, 300 mM NaCl, 25 mM imidazole, pH 8.0), and bound protein was recovered with elution buffer (50 mM KH_2PO_4, 300 mM NaCl, 200 mM imidazole, pH 8.0). Enzymes purity was analyzed by SDS-PAGE and fractions containing the enzyme were combined and dialyzed overnight against KPi buffer (4 L, pH 8.0, 50 mM). The enzyme solutions were concentrated, and their concentration was determined spectrophotometrically based on their extinction coefficient at the wavelength of 280 nm. The concentration of NOx enzyme was determined based on the extinction coefficient of FAD at the wavelength of 440 nm.

SDS-Page

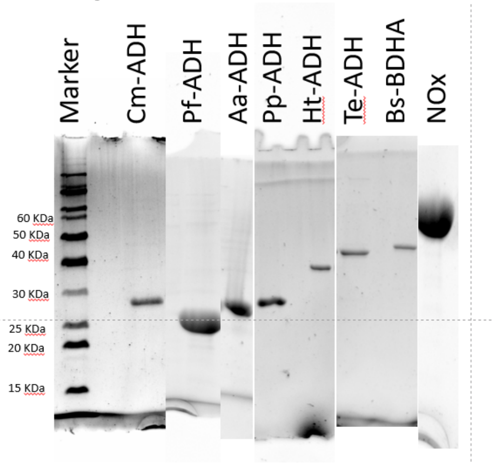

Figure S2: SDS-PAGE of purified enzymes. Marker: PageRuler™ Unstained Protein Ladder (ThermoFisher Scientific).

7. Amidation study

7.1. Reaction optimization with *Pichia finlandica* ADH

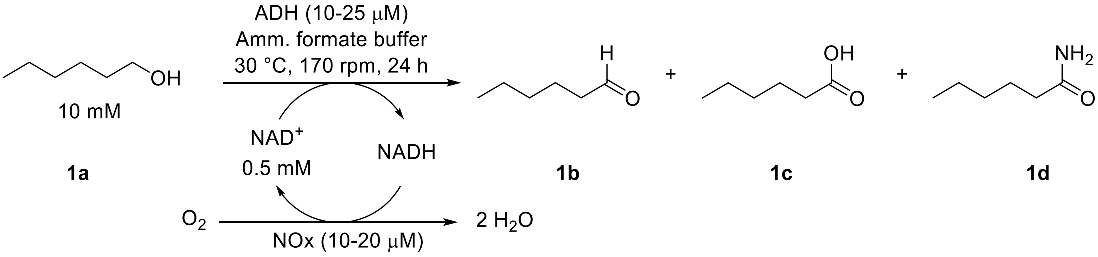

Scheme S1: First tests for the conversion of substrate 1a into amide 1d.

General procedure

In an Eppendorf tube (2 mL), NAD^+ (0.5 mM), NOx (10–20 μM) and Pf-ADH (10–25 μM) were added in ammonium formate buffer (varied concentration and pH, final volume of 1 mL). The substrate was added from a DMSO stock solution 1 M as last having, generally, a final concentration in the solution of 10 mM. The reaction was incubated at 30 °C, 170 for 24 h on an orbital shaker. Then, the aqueous phase was acidified to pH 2 with HCl 2 M and was extracted with ethyl acetate (500 µL x 2). The organic layer was dried over $MgSO_4$ and analyzed by GC-FID on DB-1701 30 m column. During the study, each point of any graph was at least the average value obtained from three independent tests.

7.1.1. Study at varied buffer concentration at pH 9

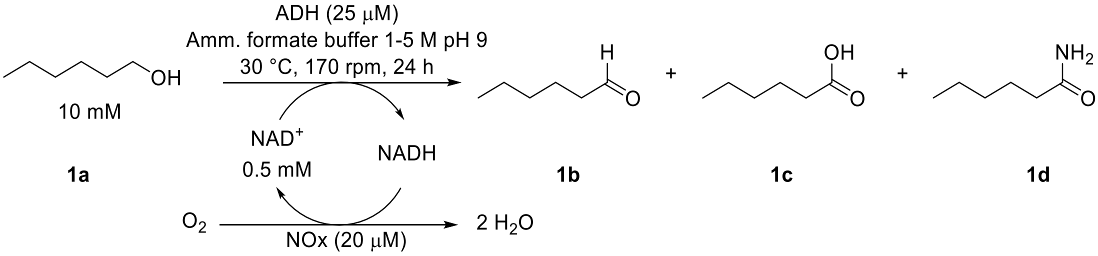

Scheme S2: Tests on substrate 1a at varied concentrated of ammonium formate buffer.

Using the general procedure, the reaction was tested on substrate **1a** at different concentrations of ammonium formate buffer (1 – 2 – 3 – 4 – 5 M). Then, the solution was extracted with HCl 2 M (i.e., pH 3-4). Each point of the table is the average obtained from three independent tests.

Table S2: Screening at varied ammonium formate buffer concentration on substrate 1a.

Buffer concentration [M]	Alcohol [%]	Aldehyde [%]	Acid [%]	Amide [%]
1	n.d.	n.d.	68	32
2	n.d.	n.d.	49	51
3	n.d.	n.d.	35	65
4	n.d.	n.d.	23	77
5	n.d.	n.d.	17	83

n.d.: not detected as it was below the detection limit.

7.1.2. Study at varied enzyme concentration

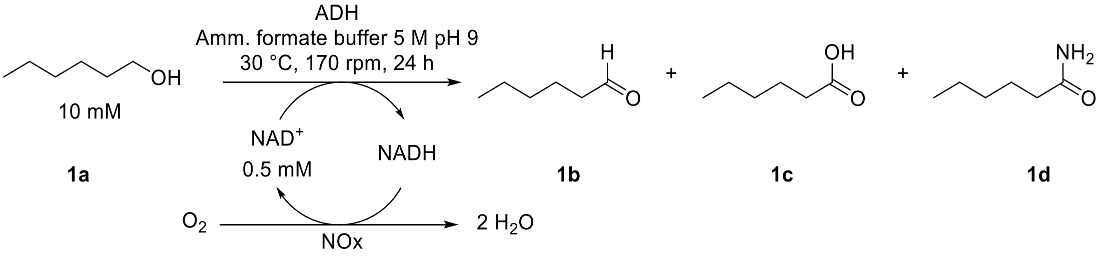

Scheme S3: Tests performed on substrate 1a at lower enzyme concentration.

Using the general procedure, the reaction was tested on substrate **1a** at different enzymes concentrations (both ADH and NOx) in ammonium formate buffer 5 M at pH 9. Each point of the table is the average obtained from three independent tests.

Table S3: Test on substrate 1a to reduce the enzyme loading.

Enzyme concentrations [µM]	Alcohol [%]	Aldehyde [%]	Acid [%]	Amide [%]
ADH 25 µM – NOx 20 µM	n.d.	n.d.	18	82
ADH 20 µM – NOx 10 µM	n.d.	n.d.	18	82
ADH 10 µM – NOx 10 µM	n.d.	n.d.	18	82
ADH 5 µM – NOx 5 µM	46	6	5	43

n.d.: not detected as it was below the detection limit.

The reaction reached complete conversion with ADH and NOx both at 10 µM; therefore, these concentrations were chosen for the next reactions.

7.1.3. Study at varied pH

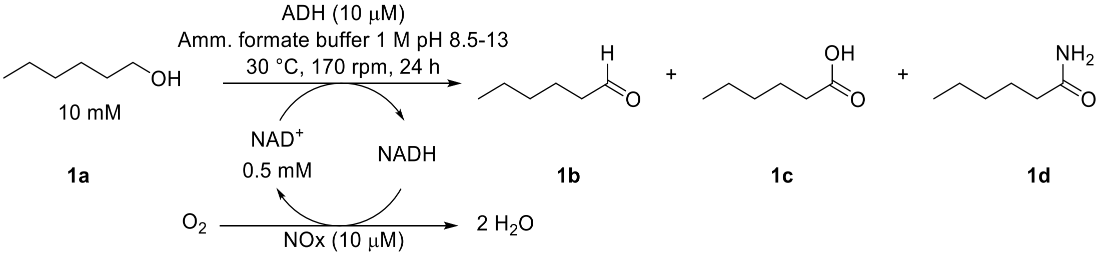

Scheme S4: Tests performed on substrate 1a with Pf-ADH at pH ranging from 8.5 to 13.

Using the general procedure, the reaction was tested on substrate **1a** at different pH values (8.5 – 9 – 9.5 – 10 – 10.5 – 11 – 12 – 13) in ammonium formate buffer 1 M. Each point of the table is the average obtained from three independent tests.

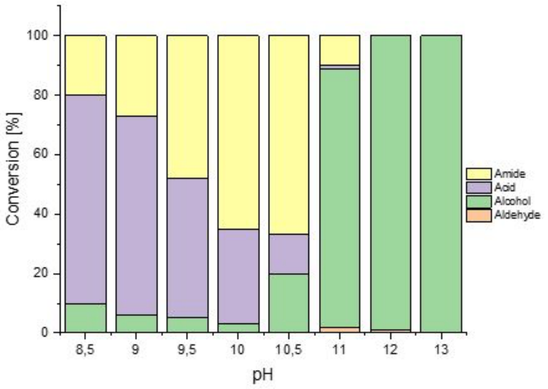

Figure S3: pH screening

Table S4: Screening at varied pH performed on 1a with Pf-ADH.

pH	Alcohol [%]	Aldehyde [%]	Acid [%]	Amide [%]
8.5	10	n.d.	70	20
9.0	6	n.d.	67	27
9.5	5	n.d.	47	48
10.0	3	n.d.	32	65
10.5	20	n.d.	13	67
11.0	87	2	1	10
12.0	99	1	n.d.	n.d.
13.0	100	n.d.	n.d.	n.d.

n.d.: not detected as it was below the detection limit.

The best alcohol conversion and amide formation was observed at pH 10.

7.1.4. Study at varied buffer concentration at pH 10

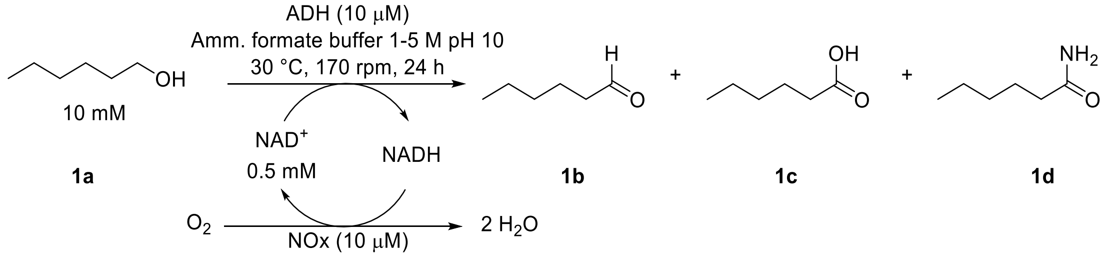

Scheme S5: Tests on substrate 1a using Pf-ADH at pH 10 and at varied ammonium formate buffer concentration.

Using the general procedure, the reaction was tested on substrate **1a** at different ammonium formate buffer concentrations (1 – 2 – 3 – 4 – 5 M) at pH 10. Each point of the table is the average obtained from three independent tests.

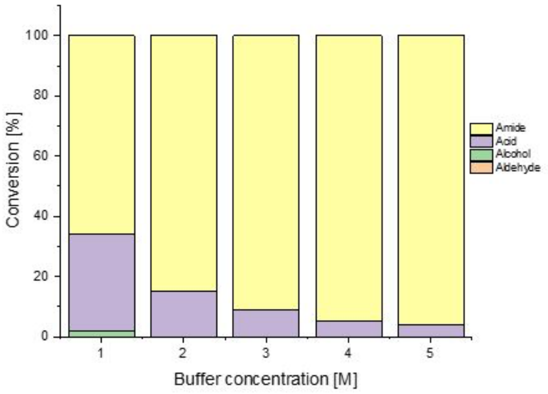

Figure S4: Concentration screening to tune the selectivity

Table S5: Screening at varied ammonium formate buffer concentration on substrate 1a with Pf-ADH and with optimized pH.

Buffer concentration [M]	Alcohol [%]	Aldehyde [%]	Acid [%]	Amide [%]
1	2	n.d.	32	66
2	n.d.	n.d.	15	85
3	n.d.	n.d.	9	91
4	n.d.	n.d.	5	95
5	n.d.	n.d.	4	96

n.d.: not detected as it was below the detection limit.

7.1.5. Study at varied temperature

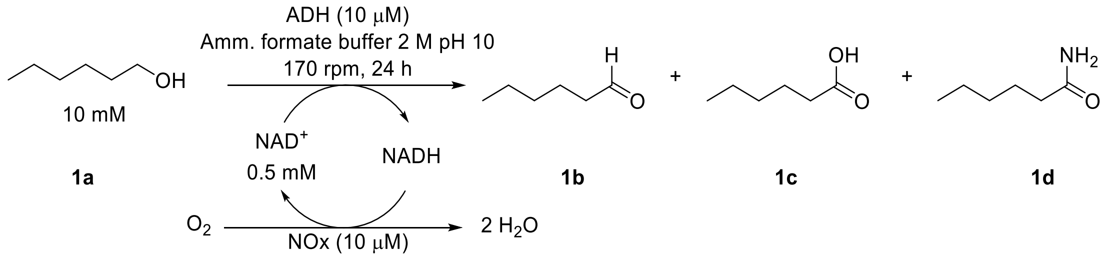

Scheme S6: Temperature study performed on substrate 1a with Pf-ADH.

Using the general procedure, the reaction was tested at different temperatures (20 – 25 – 30 – 40 °C) in ammonium formate buffer 2 M pH 10. Each point of the table is the average obtained from two independent tests.

Table S6: Reaction tested at different temperatures using 1a as substrate.

Temperature [°C]	Alcohol [%]	Aldehyde [%]	Acid [%]	Amide [%]
20	n.d.	1	12	87
25	n.d.	n.d.	12	88
30	n.d.	n.d.	15	85
40	n.d.	n.d.	18	82

n.d.: not detected as it was below the detection limit.

7.2. Reaction optimization with Aa-ADH

7.2.1. Study at varied pH

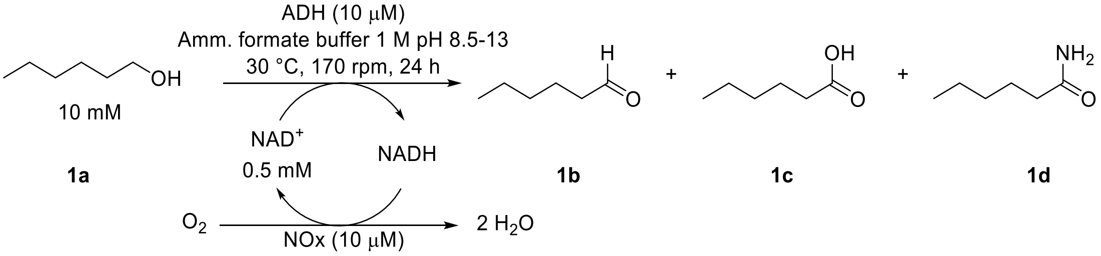

Scheme S7: Tests performed on substrate 1a with Aa-ADH at pH ranging from 8.5 to 13.
Starting from the optimized condition for the reaction catalyzed by Pf-ADH (NAD^+ 0.5 mM, ADH 10 μM, NOx 10 μM), the reaction was tested on substrate **1a** at different pH values (8.5 – 9 – 9.5 – 10 – 10.5 – 11 – 12 – 13) in ammonium formate buffer 1 M. Each point of the table is the average obtained from three independent tests.

Table S7: Screening at varied pH performed on 1a with Aa-ADH.

pH	Alcohol [%]	Aldehyde [%]	Acid [%]	Amide [%]
8.5	68	2	13	17
9.0	62	2	13	23
9.5	60	1	6	33
10.0	53	1	4	42
10.5	50	1	3	46
11.0	89	n.d.	n.d.	10
12.0	98	n.d.	n.d.	n.d.
13.0	100	n.d.	n.d.	n.d.

n.d.: not detected as it was below the detection limit.

The best alcohol conversion and amide formation was obtained at pH 10.5.

7.2.2. Study at varied buffer concentration at pH 10.5

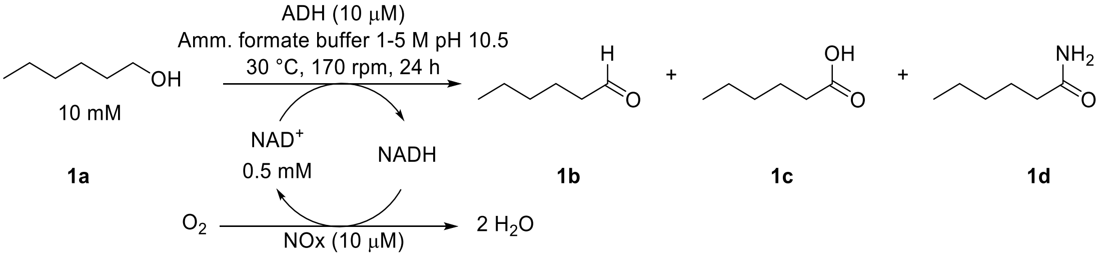

Scheme S8: Tests on substrate 1a using Aa-ADH at pH 10.5 and at varied ammonium formate buffer concentration.
Using the general procedure, the reaction was tested on substrate **1a** with ammonium buffer at pH 10.5 and at different concentrations (0.5 – 1 – 2 – 3 – 4 –5 M). Each point of the table is the average obtained from three independent tests.

Table S8: Screening at varied ammonium formate buffer concentration on substrate 1a with Aa-ADH and with optimized pH.

Buffer concentration [M]	Alcohol [%]	Aldehyde [%]	Acid [%]	Amide [%]
0.5	56	1	5	38
1	50	1	3	46
2	58	n.d.	1	40
3	63	n.d.	1	36
4	68	n.d.	1	31
5	71	n.d.	n.d.	29

n.d.: not detected as it was below the detection limit.

7.2.3. Study at varied temperature

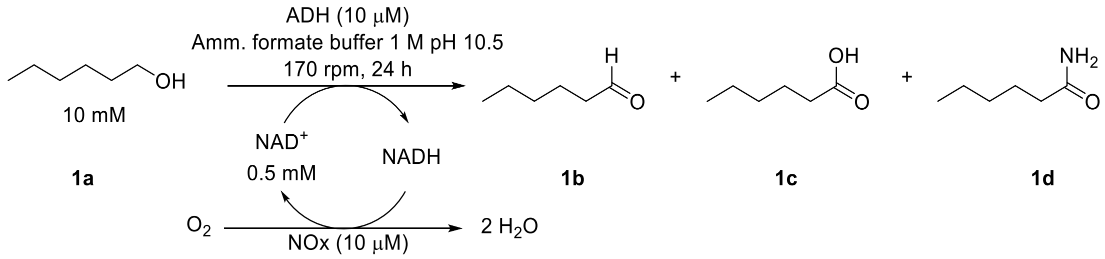

Scheme S9: Temperature study performed on substrate 1a with Aa-ADH.

Using the general procedure, the reaction was tested on substrate **1a** at different temperatures (20 – 25 – 30 – 40 °C) in ammonium formate buffer 1 M pH 10.5. Each point of the graph is the average obtained from two independent tests.

Table S9: Reaction tested at different temperatures with 1a with Aa-ADH.

Temperature [°C]	Alcohol [%]	Aldehyde [%]	Acid [%]	Amide [%]
20	78	n.d.	1	21
25	73	n.d.	2	25
30	50	1	3	46
40	90	n.d.	n.d.	10

n.d.: not detected as it was below the detection limit.

7.3. Reaction optimization with Pp-ADH

7.3.1. Study at varied pH

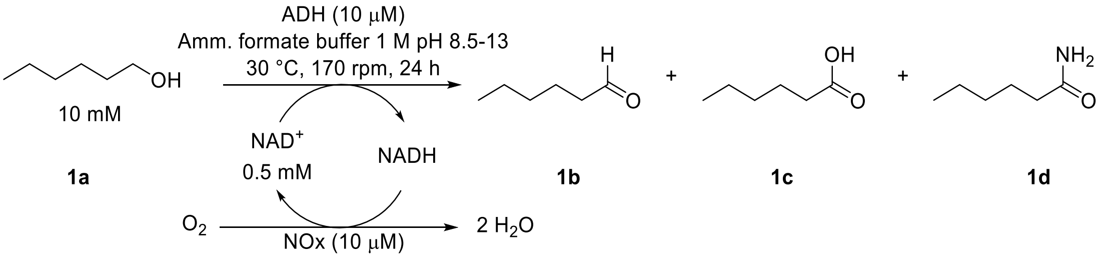

Scheme S10: Tests performed on substrate 1a with Pp-ADH at pH ranging from 8.5 to 13.

Starting from the optimized condition for the reaction catalyzed by Pf-ADH (NAD^+ 0.5 mM, ADH 10 µM, NOx 10 µM), the reaction was tested on substrate **1a** at different pH values (8.5 – 9 – 9.5 – 10 – 10.5 – 11 – 12 – 13) in ammonium formate buffer 1 M. Each point of the table is the average obtained from three independent tests.

Table S10: Screening at varied pH performed on 1a with Pp-ADH.

pH	Alcohol [%]	Aldehyde [%]	Acid [%]	Amide [%]
8.5	n.d.	n.d.	86	14
9.0	3	1	64	32
9.5	n.d.	n.d.	58	42
10.0	n.d.	n.d.	39	61
10.5	21	1	24	52
11.0	90	4	2	4
12.0	100	n.d.	n.d.	n.d.
13.0	100	n.d.	n.d.	n.d.

n.d.: not detected as it was below the detection limit.

The best alcohol conversion and amide formation is observed at pH 10.

7.3.2. Study at varied buffer concentration at pH 10

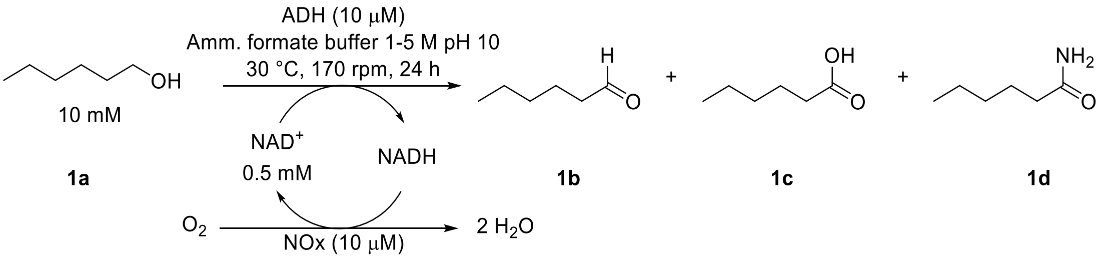

Scheme S11: Tests on substrate 1a using Pp-ADH at pH 10 and at varied ammonium formate buffer concentration.

Using the general procedure, the reaction was tested on substrate **1a** with ammonium buffer pH 10 at different concentrations (0.5 – 1 – 2 – 3 – 4 –5 M). Each point of the table is the average obtained from three independent tests.

Table S11: Screening at varied ammonium formate buffer concentration on substrate 1a with Pp-ADH and with optimized pH.

Buffer concentration [M]	Alcohol [%]	Aldehyde [%]	Acid [%]	Amide [%]
0.5	n.d.	n.d.	59	41
1	n.d.	n.d.	39	61
2	n.d.	n.d.	19	81
3	n.d.	n.d.	13	87
4	n.d.	n.d.	9	91
5	n.d.	n.d.	5	95

n.d.: not detected as it was below the detection limit.

7.3.3. Study at varied temperature

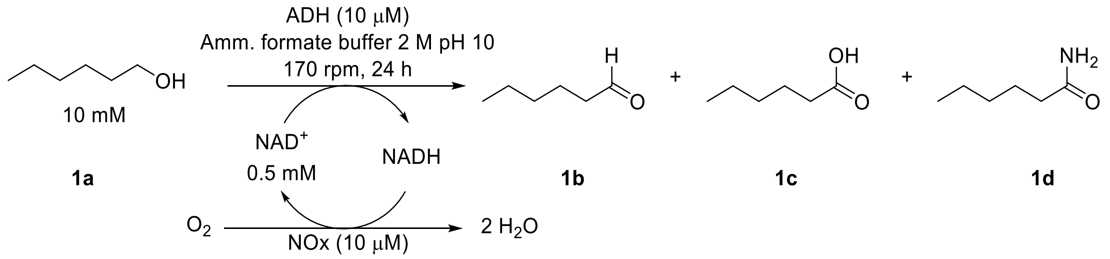

Scheme S12: Temperature study performed on substrate 1a with Pp-ADH.

Using the general procedure, the reaction was tested on substrate **1a** at different temperatures (25 – 30 – 35 °C) in ammonium formate buffer 1 M pH 10. Each point of the table is the average obtained from two independent tests.

Table S12: Reaction tested at different temperatures with 1a with Pp-ADH.

Temperature [°C]	Alcohol [%]	Aldehyde [%]	Acid [%]	Amide [%]
25	n.d.	n.d.	21	78
30	n.d.	n.d.	19	81
35	n.d.	n.d.	19	81

n.d.: not detected as it was below the detection limit.

7.4. Reaction optimization with Te-ADH W110A

7.4.1. Study at varied pH

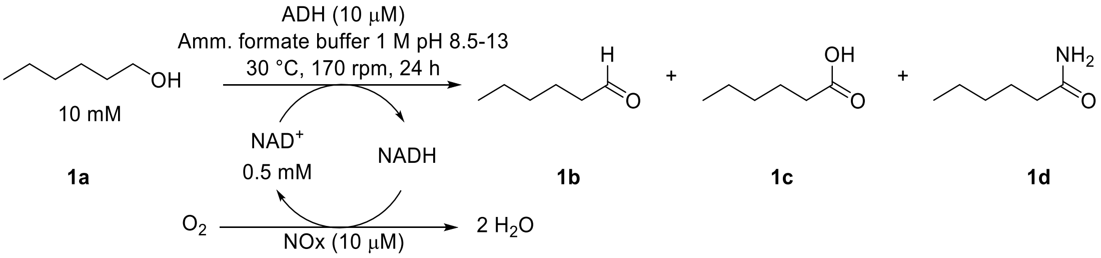

Scheme S13: Tests performed on substrate 1a with Te-ADH W110A at pH ranging from 8.5 to 13.
Starting from the optimized condition for the reaction catalyzed by Pf-ADH ($NADP^+$ 0.5 mM, ADH 10 μM, NOx 10 μM), the reaction was tested on substrate **1a** with different pH values (8.5 – 9 – 9.5 – 10 – 10.5 – 11 – 12 – 13) in ammonium formate buffer 1 M. Each point of the table is the average obtained from three independent tests.

Table S13: Screening at varied pH performed on 1a with Te-ADH W110A.

pH	Alcohol [%]	Aldehyde [%]	Acid [%]	Amide [%]
8.5	69	1	27	4
9.0	65	1	23	12
9.5	64	1	20	16
10.0	84	2	3	12
10.5	97	1	n.d.	2
11.0	99	1	n.d.	n.d.
12.0	100	n.d.	n.d.	n.d.
13.0	100	n.d.	n.d.	n.d.

n.d.: not detected as it was below the detection limit.

The best alcohol conversion and amide formation was observed at pH 9.5.

7.4.2. Study at varied buffer concentration at pH 9.5

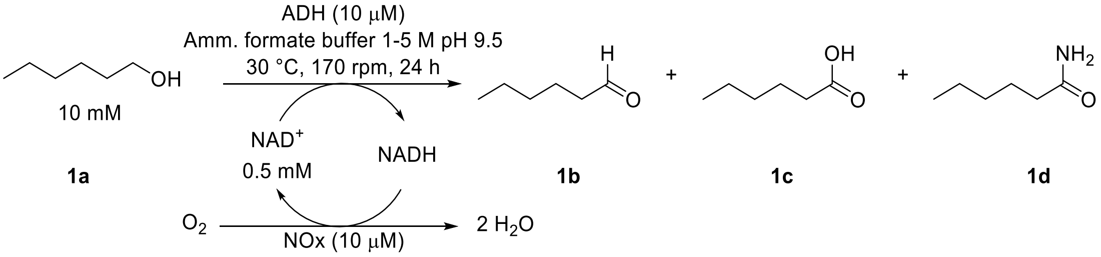

Scheme S14: Tests on substrate 1a using Te-ADH W110A at pH 9.5 and at varied ammonium formate buffer concentration.
Using the general procedure, the reaction was tested on substrate **1a** with ammonium buffer at pH 9.5 and at different concentrations (0.5 – 1 – 2 – 3 – 4 –5 M). Each point of the table is the average obtained from three independent tests.

Table S14: Screening at varied ammonium formate buffer concentration on substrate 1a with Te-ADH W110A and with optimized pH.

Buffer concentration [M]	Alcohol [%]	Aldehyde [%]	Acid [%]	Amide [%]
0.5	43	1	46	10
1	64	1	20	16
2	86	1	5	9
3	89	2	2	7
4	95	1	2	2
5	96	1	2	1

7.4.3. Study at varied temperature

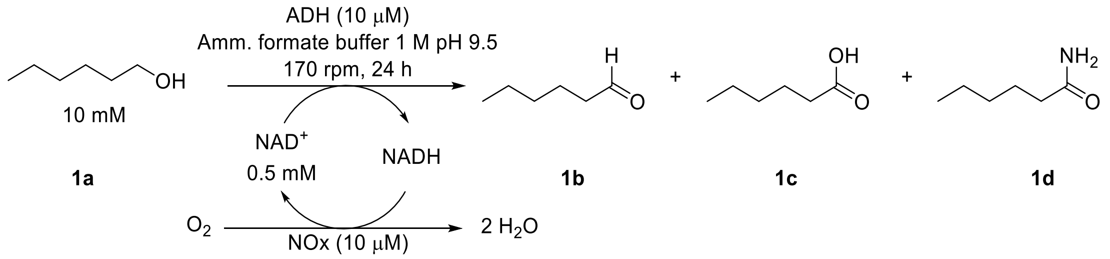

Scheme S15: Temperature study performed on substrate 1a with Te-ADH W110A.

Using the general procedure, the reaction was tested on substrate **1a** at different temperatures (25 – 30 – 40 °C) in ammonium formate buffer 1 M pH 9.5. Each point of the table is the average obtained from two independent tests.

Table S15: Reaction tested at different temperatures with 1a with Te-ADH 110A.

Temperature [°C]	Alcohol [%]	Aldehyde [%]	Acid [%]	Amide [%]
25	64	1	24	11
30	64	1	20	16
35	49	1	37	12

7.5. Reaction optimization with Ht-ADH

7.5.1. Study at varied pH

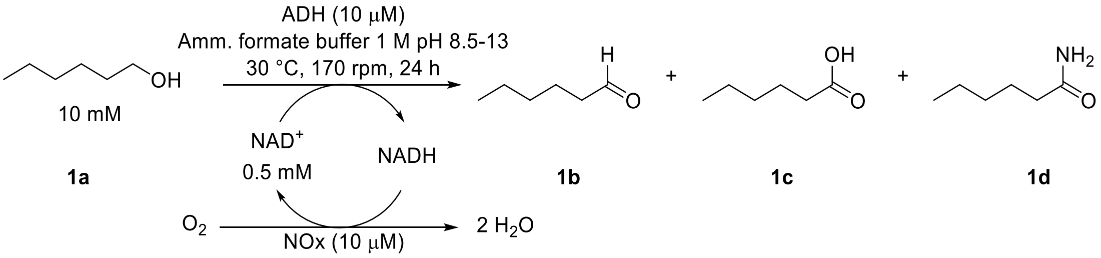

1a 1b 1c 1d

Scheme S16: Tests performed on substrate 1a with Ht-ADH at pH ranging from 8.5 to 13.
Starting from the optimized condition for the reaction catalyzed by Pf-ADH (NAD^+ 0.5 mM, ADH 10 μM, NOx 10 μM), the reaction was tested with different pH values (8.5 – 9 – 9.5 – 10 – 10.5 – 11 – 12 – 13) in ammonium formate buffer 1 M. Each point of the table is the average obtained from three independent tests.

Table S16: Screening at varied pH performed on 1a with Ht-ADH.

pH	Alcohol [%]	Aldehyde [%]	Acid [%]	Amide [%]
8.5	69	1	27	4
9.0	69	1	23	7
9.5	67	1	22	10
10.0	88	2	2	8
10.5	97	1	n.d.	2
11.0	99	1	n.d.	n.d.
12.0	100	n.d.	n.d.	n.d.
13.0	100	n.d.	n.d.	n.d.

n.d.: not detected as it was below the detection limit.

The best alcohol conversion and amide formation was observed at pH 9.5.

7.5.2. Study at varied buffer concentration at pH 9.5

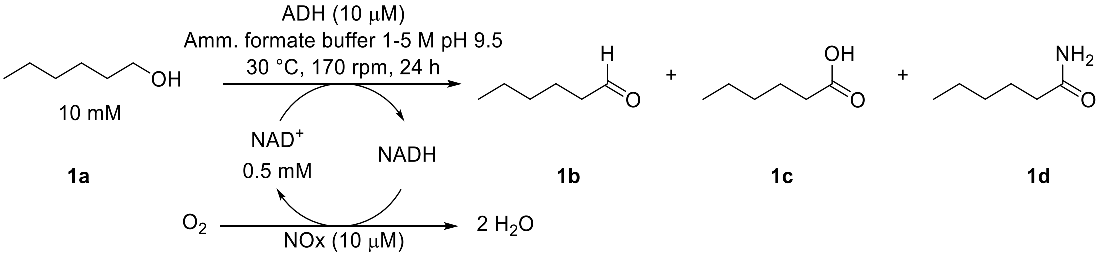

1a 1b 1c 1d

Scheme S17: Tests on substrate 1a using Ht-ADH at pH 9.5 and at varied ammonium formate buffer concentration.
Using the general procedure, the reaction was tested with ammonium buffer pH 9.5 at different concentrations (0.5 – 1 – 2 – 3 – 4 –5 M). Each point of the table is the average obtained from three independent tests.

Table S17: Screening at varied ammonium formate buffer concentration on substrate 1a with Ht-ADH and with optimized pH.

Buffer concentration [M]	Alcohol [%]	Aldehyde [%]	Acid [%]	Amide [%]
0.5	6	12	78	4
1	67	1	22	10
2	21	60	25	4
3	13	71	13	3
4	40	54	4	2
5	46	48	5	1

7.5.3. Study at varied temperature

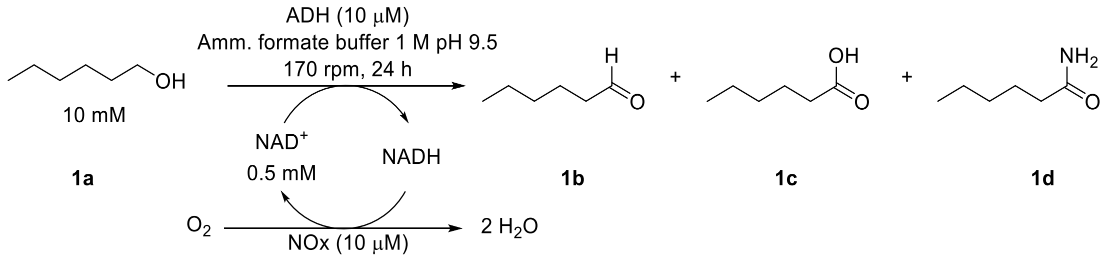

Scheme S18: Temperature study performed on substrate 1a with Ht-ADH.

Using the general procedure, the reaction was tested at different temperatures (20 – 25 – 30 – 35 °C) in ammonium formate buffer 1 M pH 9.5. Each point of the table is the average obtained from two independent tests.

Table S18: Reaction tested at different temperatures with 1a with Ht-ADH.

Temperature [°C]	Alcohol [%]	Aldehyde [%]	Acid [%]	Amide [%]
20	n.d.	14	78	8
25	n.d.	13	73	12
30	27	51	22	10
35	34	44	18	4

n.d.: not detected as it was below the detection limit.

7.6. Different amine buffers

The reaction with the five ADHs were tested with $MeNH_2$, $EtNH_2$, $nPrNH_2$, $nBuNH_2$, $allylNH_2$, $BnNH_2$, Me_2NH and Et_2NH amine buffers, which were prepared by adding the desired concentration of amine in water and adjusting the pH with formic acid. The reactions were performed on the model substrate **1a**, using the optimized conditions obtained in ammonium formate buffer for each enzyme.

The reaction on $EtNH_2$, $nPrNH_2$, $nBuNH_2$, $allylNH_2$, $BnNH_2$, Me_2NH and Et_2NH formate buffers did not lead to any product formation. The alcohol was neither converted to the corresponding amide nor to the aldehyde. In contrast, good to excellent product formation was obtained in $MeNH_2$ buffer. The results obtained with $MeNH_2/(HCOO)^{-}MeNH_3^{+}$ buffer are reported in Table S19.

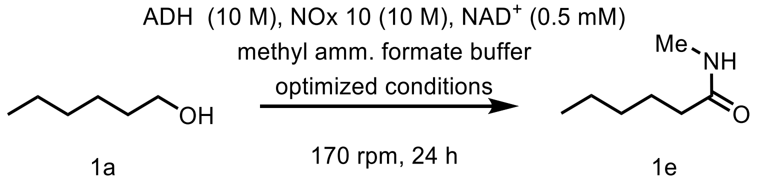

Scheme S19: Test on substrate 1a with methylamine/methylammonium formate buffer.

Table S19: Methylamine/methylammonium formate buffer tested with each enzyme on substrate 1a.

Enzyme	Aldehyde [%]	Alcohol [%]	Acid [%]	Amide [%]
Pf-ADH	1	4	1	94
Pp-ADH	2	8	1	89
AA-ADH	1	89	n.d.	11
Te-ADH	2	56	18	24
Ht-ADH	100	n.d.	n.d.	n.d.

n.d.: not detected as it was below the detection limit.

7.7. Time study

The time study of the reaction on the model substrate **1a** was performed using the enzymes that led to the best results, namely Pf-ADH, AA-ADH and Pp-ADH. The study was performed either with ammonia or methylamine as the amine donors. The reactions were monitored after 0.5, 1, 2, 4, 6 and 24 hours.

With AA-ADH, we also performed a time study on substrate **14a** with ammonia and on substrate **20a** with methylamine.

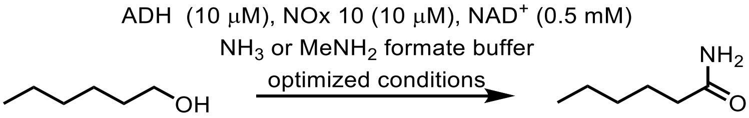

Scheme S20: Example of time study performed on substrate 1a with Pf-ADH, Pp-AADH and Aa-ADH.

7.7.1. Pf-ADH and NH_3

Table S20: Time study on substrate 1a with Pf-ADH in ammonium formate buffer under optimized conditions.

Time [hours]	Alcohol [%]	Aldehyde [%]	Acid [%]	Amide [%]
0	100	n.d.	n.d.	n.d.
0.5	85	6	1	7
1	66	6	4	24
2	31	4	8	57
4	n.d.	1	11	88
6	n.d.	n.d.	12	88
24	n.d.	n.d.	12	88

n.d.: not detected as it was below the detection limit.

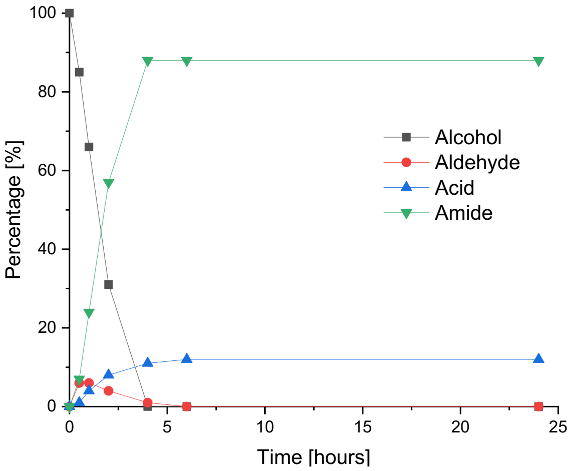

Scheme S21: Time study performed on substrate 1a in ammonium formate buffer with Pf-ADH.

7.7.2. Pf-ADH and $MeNH_2$

Table S21: Time study on substrate 1a with Pf-ADH and methylammonium formate buffer under optimized conditions.

Time [h]	Alcohol [%]	Aldehyde [%]	Acid [%]	Methylamide [%]
n.d.	100	n.d.	n.d.	n.d.
0.5	83	1	n.d.	16
1	58	1	n.d.	41
2	8	1	1	90
4	4	1	1	94
6	4	1	1	94
24	4	1	1	94

n.d.: not detected as it was below the detection limit.

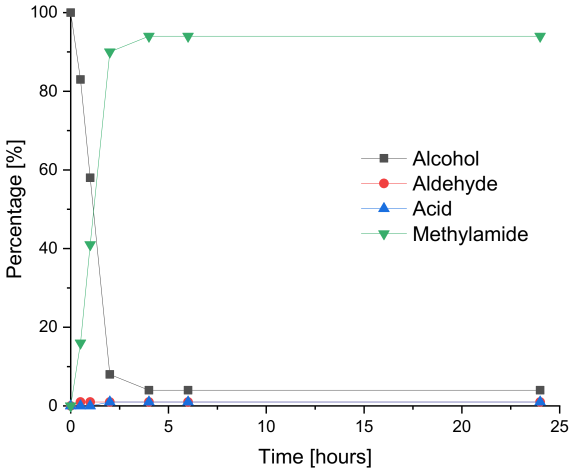

Scheme S22: Time study performed on substrate 1a in methylammonium formate buffer with Pf-ADH.

7.7.3. Pp-ADH and NH_3

Table S22: Time study on substrate 1a with Pp-ADH and ammonium formate buffer under optimized conditions.

Time [h]	Alcohol [%]	Aldehyde [%]	Acid [%]	Amide [%]
n.d.	100	n.d.	n.d.	n.d.
0.5	86	11	n.d.	3
1	64	18	3	15
2	36	20	6	38
4	14	13	11	62
6	n.d.	9	13	78
24	n.d.	n.d.	19	81

n.d.: not detected as it was below the detection limit.

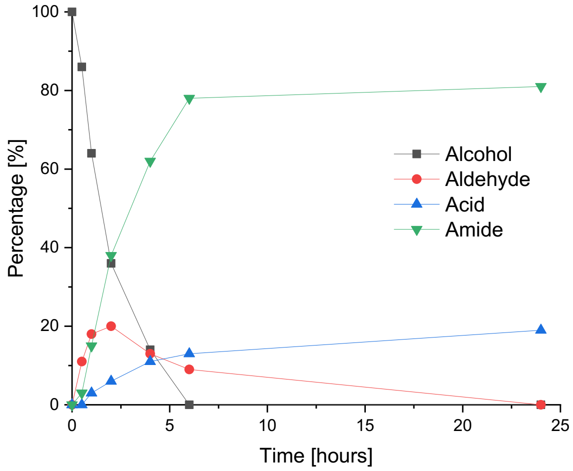

Scheme S23: Time study performed on substrate 1a in ammonium formate buffer with Pp-ADH.

7.7.4. Pp-ADH and $MeNH_2$

Table S23: Time study on substrate 1a with Pp-ADH and methylammonium formate buffer under optimized conditions.

Time [h]	Alcohol [%]	Aldehyde [%]	Acid [%]	Methylamide [%]
n.d.	100	n.d.	n.d.	n.d.
0.5	84	4	n.d.	12
1	65	4	n.d.	31
2	16	2	n.d.	84
4	8	2	1	89
6	8	2	2	89
24	8	2	1	89

n.d.: not detected as it was below the detection limit.

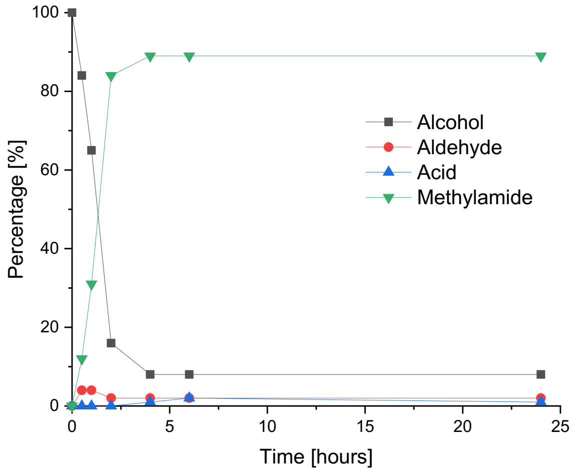

Scheme S24: Time study performed on substrate 1a in methylammonium formate buffer with Pp-ADH.

7.7.5. AA-ADH and NH_3

Table S24: Time study on substrate 1a with Aa-ADH and ammonium formate buffer under optimized conditions.

Time [h]	Alcohol [%]	Aldehyde [%]	Acid [%]	Amide [%]
n.d.	100	n.d.	n.d.	n.d.
0.5	98	1	n.d.	1
1	97	2	n.d.	1
2	93	2	n.d.	5
4	84	2	1	14
6	80	1	1	18
24	51	n.d.	3	46

n.d.: not detected as it was below the detection limit.

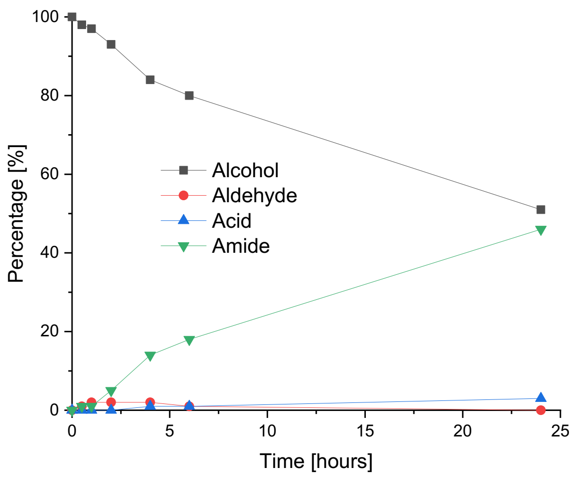

Scheme S25: Time study performed on substrate 1a in ammonium formate buffer with Aa-ADH.

7.7.6. AA-ADH and $MeNH_2$

Table S25: Time study on substrate 1a with Aa-ADH and methylammonium formate buffer under optimized conditions.

Time [h]	Alcohol [%]	Aldehyde [%]	Acid [%]	Methylamide [%]
n.d.	100	n.d.	n.d.	n.d.
0.5	99	1	n.d.	n.d.
1	98	2	n.d.	n.d.
2	93	4	n.d.	3
4	90	4	n.d.	6
6	90	2	n.d.	8
24	89	1	n.d.	11

n.d.: not detected as it was below the detection limit.

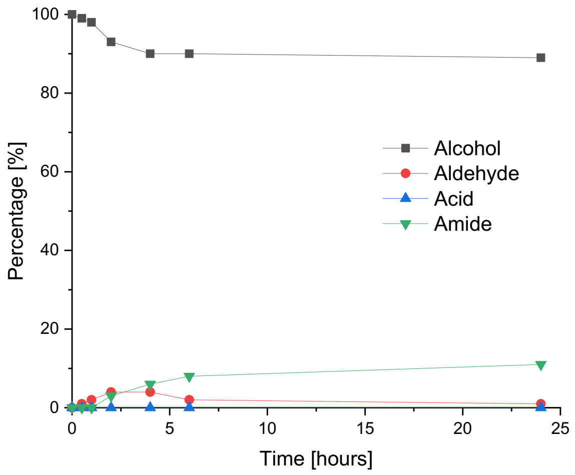

Scheme S26: Time study performed on substrate 1a in methylammonium formate buffer with Aa-ADH.

7.7.7. AA-ADH and NH_3 with substrate 14a

Table S26: Time study on substrate 14a with Aa-ADH and ammonium formate buffer under optimized conditions.

Time [h]	Alcohol [%]	Aldehyde [%]	Acid [%]	Amide [%]
n.d.	100	n.d.	n.d.	n.d.
0.5	96	4	n.d.	n.d.
1	89	4	n.d.	7
2	83	4	n.d.	13
4	51	4	n.d.	45
6	31	4	n.d.	65
24	1	n.d.	n.d.	99

n.d.: not detected as it was below the detection limit.

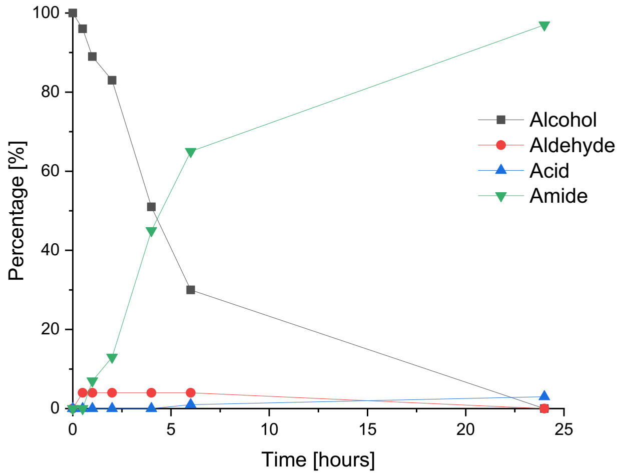

Scheme S27: Time study performed on substrate 14a in ammonium formate buffer with Aa-ADH.

7.7.8. AA-ADH and $MeNH_2$ with substrate 20a

Table S27: Time study on substrate 20a with Aa-ADH and methylammonium formate buffer under optimized conditions.

Time [h]	Alcohol [%]	Aldehyde [%]	Acid [%]	Amide [%]
n.d.	100	n.d.	n.d.	n.d.
0.5	96	4	n.d.	n.d.
1	89	4	n.d.	7
2	83	4	n.d.	13
4	51	4	n.d.	45
6	31	4	n.d.	65
24	n.d.	n.d.	n.d.	100

n.d.: not detected as it was below the detection limit.

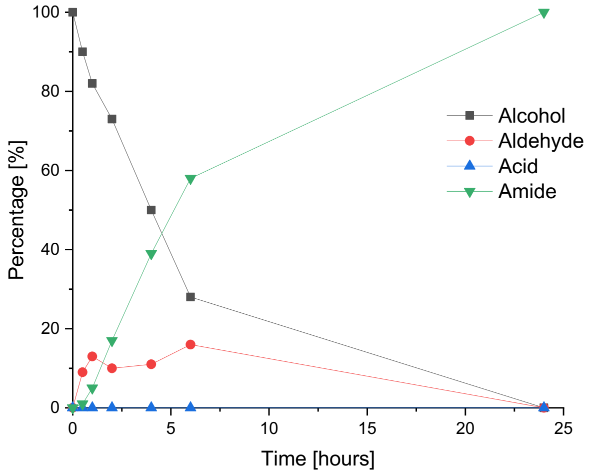

Scheme S28: Time study performed on substrate 20a in methylammonium formate buffer with Aa-ADH.

7.8. Control experiments to verify catalytic promiscuity and mechanism

The reaction was tested under optimized reaction condition for both amides and thioesters formation in the following cases:

1) Without any ADH.
2) Without any ADH and using the aldehyde as starting material.
3) Without NOx but with NAD^+ in catalytic amount (5 mol%).
4) Without NOx but with 1.1 eq of NAD^+.
5) Without NOx but with 2.2 eq of NAD^+.
6) Without NOx, but with 1.1 eq of NAD^+ and using the aldehyde as starting material.
7) Without external addition of NAD^+ (Note: some NAD^+/NADH is bound to the enzyme during expression and purification).
8) Without NOx and ADHs.
9) Without NOX and NAD^+.
10) Without any ADH and NAD^+.
11) Without any ADH, NOx and NAD^+ but using the aldehyde as starting material.
12) In amine free buffer (HCO_3^-/CO_3^{2-} 1 M pH 10) with all the other enzymes and cofactors in the reaction mixture.
13) In amine free buffer (HCO_3^-/CO_3^{2-} 1 M pH 10) using the aldehyde as starting material but without ADH.
14) In amine free buffer (HCO_3^-/CO_3^{2-} 1 M pH 10) using the aldehyde as starting material, but without ADH and NAD^+.
15-16) No ADH, alcohol and aldehyde as substrates in the reaction conditions in H_2S formate buffer
17-18) No ADH, alcohol and aldehyde as substrates in the reaction conditions in MeSH formate buffer
19-20) No ADH, alcohol and aldehyde as substrates in the reaction conditions in bphasic system with EtSH
21-22-23) No ADH, Carboxylic acid in reaction conditions
24) No NOx, stoichiometric amount of NAD

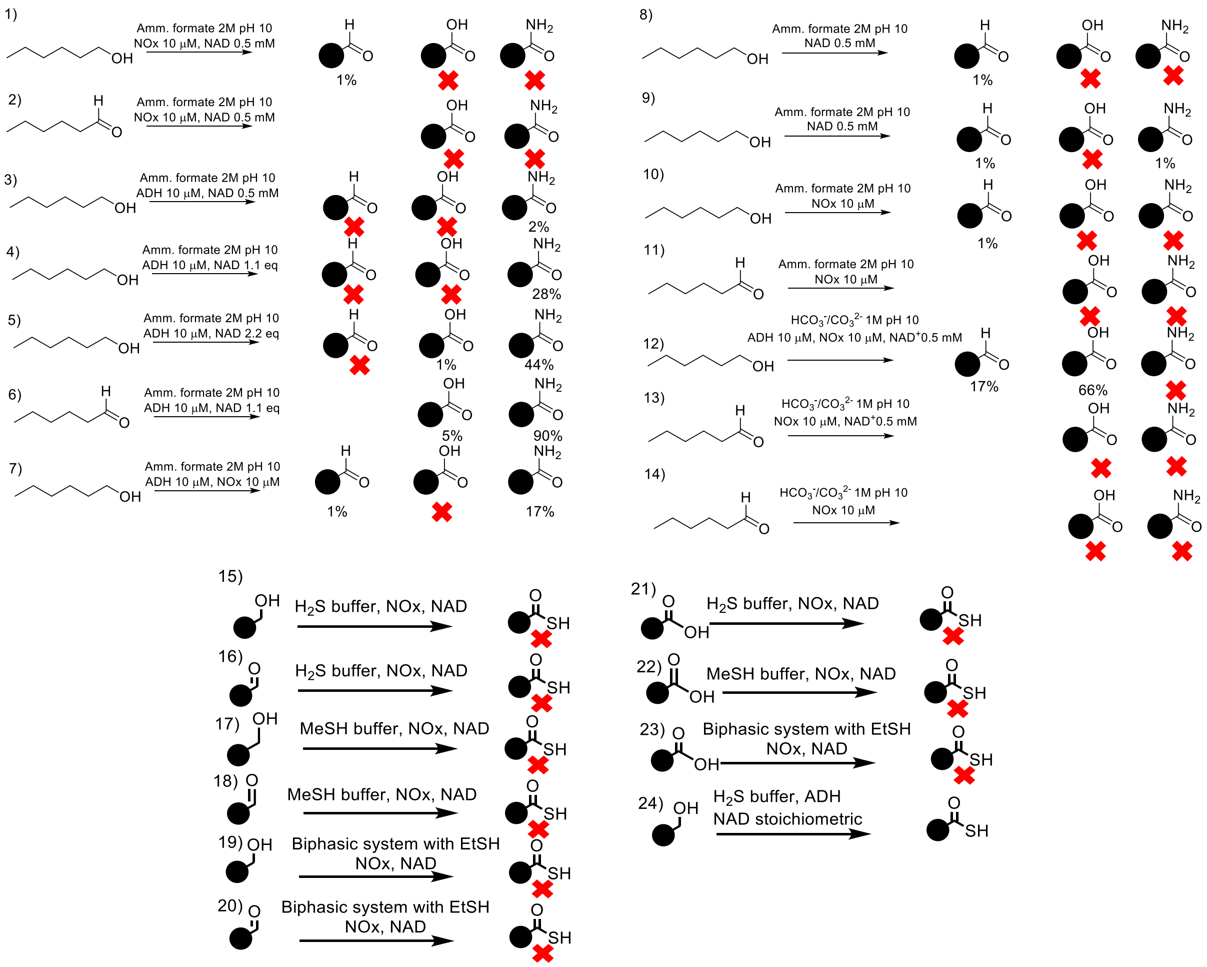

Scheme S29: Control experiments on substrate 1a to validate the catalytic promiscuous activity and reaction pathway.

7.9. Preparative scale synthesis of N-methylhexanamide

Test at increased substrate loading

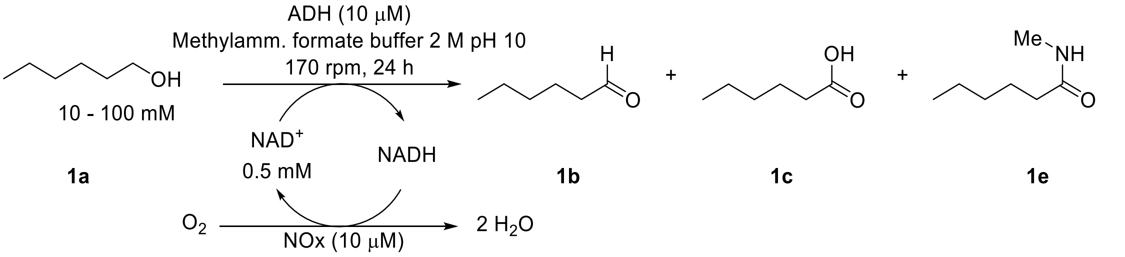

Scheme S30: Test on substrate 1a at increased substrate loading.

The reaction was tested under the optimized reaction conditions for the reaction catalyzed by Pf-ADH with substrate **1a** (ammonium formate 2 M pH 10, NOx 10 µM, NAD^+ 0.5 mM) at different substrate loadings: 10, 30, 50, 70, 100 mM, using the purified form of the enzyme.

The results are reported in Table S28.

Table S28: Increased substrate loading for the preparative scale synthesis of substrate 1a.

Substrate loading	Aldehyde	Alcohol	Acid	Amide
10 mM	1	4	1	94
20 mM	4	71	n.d.	25
30 mM	2	90	n.d.	8
50 mM	1	98	n.d.	1
70 mM	1	99	n.d.	n.d.
100 mM	1	99	n.d.	n.d.

n.d.: not detected as it was below the detection limit.

Afterwards, we decided to test the reaction on substrate **1a** (20 mM) by increasing the enzymes concentrations and using the optimized conditions for the reaction catalyzed by Pf-ADH.

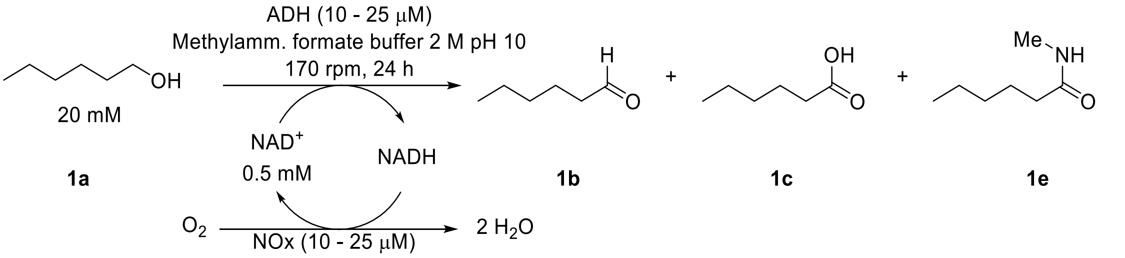

Scheme S31: Increased enzyme concentration for the reaction at 20 mM substrate 1a concentration to improve the conversion.

Table S29: Test at increased enzyme concentration to improve the conversion.

Enzyme concentration	Aldehyde	Alcohol	Acid	Amide
10 µM ADH<br>10 µM NOx	4	71	n.d.	25
20 µM ADH<br>10 µM NOx	4	67	n.d.	31
20 µM ADH<br>15 µM NOx	7	43	n.d.	50
20 µM ADH<br>20 µM NOx	6	23	n.d.	71
25 µM ADH<br>25 µM NOx	1	1	n.d.	98

n.d.: not detected as it was below the detection limit.

Scale-up experiment

0.668 g of lyophilized crude lysate deriving from cells overexpressing Pf-ADH (i.e., corresponding to ca. 20 µM of the pure enzyme in the final mixture) were suspended in a 1 L Erlenmeyer flask with 245 mL of methylammonium formate buffer (2 M, pH 10). The resulting mixture was incubated for 15 minutes, 170 rpm and 25 °C in an orbital shaker. Then, NAD^+ (0.5 mM), NOx (20 µM) and the substrate **1a** (20 mM) were added. The reaction mixture was incubated for 5 h, 170 rpm at 30 °C in an orbital shaker.
The reaction was quenched with HCl 2 M to pH 3-4, extracted with ethyl acetate (50 mL x 3), dried over $MgSO_4$ and analyzed via GC-FID, as previously reported in analytical scale experiments. The conversion was >99%. Afterwards, the solvent was removed, yielding 531 mg of the desired product (4.27 mmol, isolated yield 87%, considering the EtOAc present in the sample).

Productivity metrics:
Product: 4.27 mmol, 531 mg
Conversion: 99%
Isolated yield: 87%
Enzyme: 20 µM x 0.245 L = 4.90 µmol

TON = (moles product)/(moles enzyme) = 4270 µmol / 4.90 µmol = **871**
TOF = TON/time = 871 / 5h = 174.3 h^{-1} = **0.0484 s^{-1}**

Space time yield (STY, mmol L^{-1} h^{-1}) = 4.27 mmol / (0.245 L × 5 h) = **3.48 mmol·L^{-1}·h^{-1}**
Space time yield (mass) = 531 mg / (0.245 L x 5 h) = **433 mg·L^{-1}·h^{-1}**

^{1}HNMR (300 Hz, $CDCl_3$): 5.52 (s, 1H), 2.82 (d, J = 4.7 Hz, 3H), 2.18 (t, J = 7.4 Hz, 2H), 1.65 (p, J = 7.5 Hz, 2H), 1.33 (m, 4H), 0.90 (t, J = 6.9 Hz, 3H).

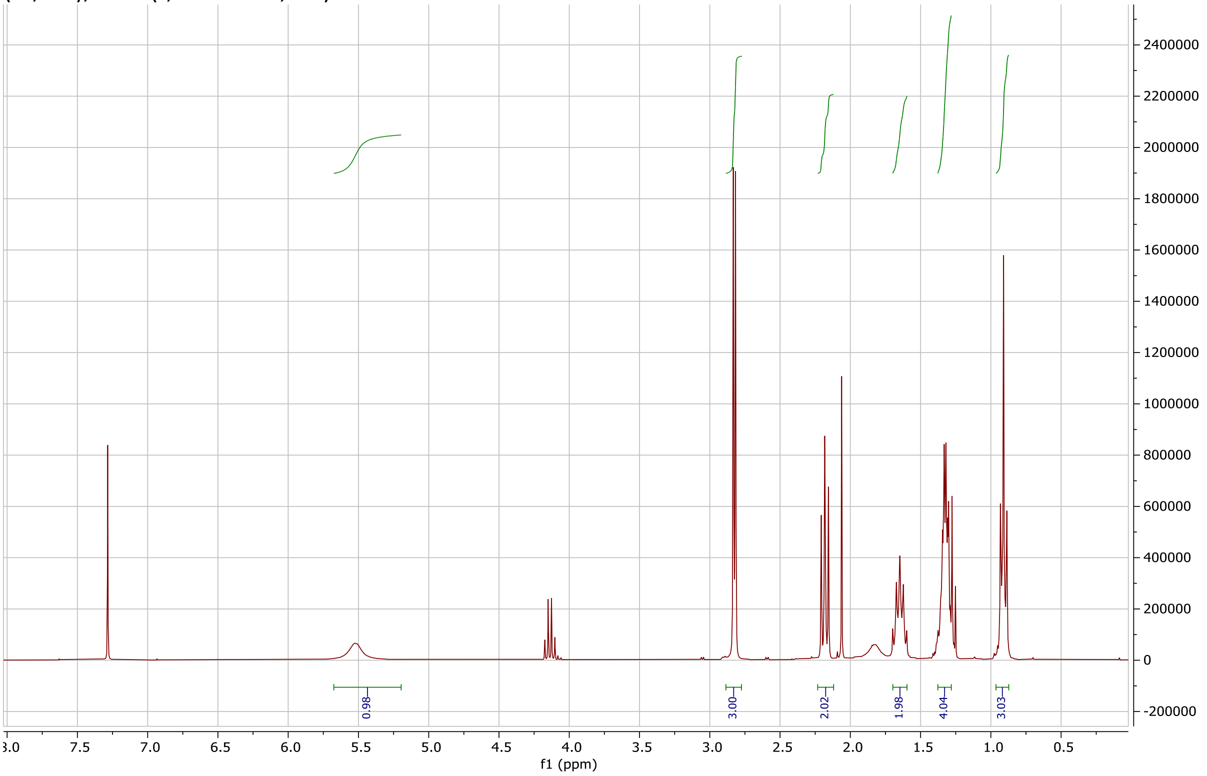

Figure S5: ^{1}H-NMR of product 1e obtained by the preparative scale reaction.

7.10. Reaction with substrate 26-28a

The substrates **26-28a** were not compatible with the acid work-up needed to extract all the components of the reaction (alcohol, aldehyde, carboxylic acid and amide) because in an acid environment the nitrogen of the pyridine ring is protonated, and the compounds are hard to extract quantitatively.

Therefore, for an accurate determination as reported in the main manuscript Figures 3 and 4, we selected the enzyme which led to the best results and performed the biotransformation on 100 mg of each substrate. For these substrates, the main manuscript reports the isolated yield of these compounds, using the crude lysate enzyme instead of the purified form. The procedure is the same as applied for the scale-up experiment on the synthesis of the *N*-methylhexanamide.

7.11. Quantitative determination of carboxylic acid

Since high amount of carboxylic acids can generate broad peaks in GC-FID, we decided to quantify them both as carboxylic acids and as methyl esters after derivatization. The derivatization was obtained with the following procedure.

The extracts were diluted to obtain a final concentration of 5 mM in 1 mL reaction. To the organic phase (250 µL), EtOAc (540 µL), MeOH (200 µL) and (trimethylsilyl)diazomethane (10 µL) were added and the reaction was shaken at 30 °C, 160 rpm for 60 min. The excess of the derivatization reagent was destroyed by the addition of acetic acid (2 µL) and incubation for further 30 min at 30 °C. Analysis was performed by GC-FID.

Both the analysis of the carboxylic acid and the methyl ester led to the same conversion of the alcohol into the corresponding acid.

7.12. Longer chain amines screening

With the optimized conditions found for WT Aa-ADH (amine formate buffer 1 M, pH 10.5), we screened the three new variants (Y93A, Y151A and L186A) with three model substrates (1a, 10a and 14a).

**Substrate 1a**	**$EtNH_2$**	**n-$PrNH_2$**	**i-$PrNH_2$**
Y93A	traces	traces	traces
Y151A	37	42	3
L186A	traces	traces	traces

**Substrate 10a**	**$EtNH_2$**	**n-$PrNH_2$**	**i-$PrNH_2$**
Y93A	-	-	-
Y151A	6	3	-
L186A	-	-	-

**Substrate 14a**	**$EtNH_2$**	**n-$PrNH_2$**	**i-$PrNH_2$**
Y93A	-	-	-
Y151A	-	-	-
L186A	-	-	-

8. Thioacids synthesis

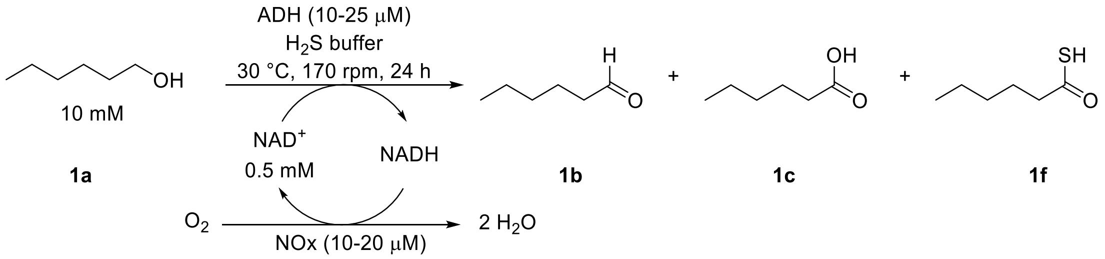

Scheme S32: First tests for the conversion of substrate 1a into thioacid 1f.

General procedure

In an Eppendorf tube (2 mL), NAD+ (0.5 mM), NOx (10–20 µM) and Pf-ADH (10–25 µM) were added in sulfuric acid buffer (varied concentration and pH, final volume of 1 mL). The substrate was added from a DMSO stock solution 1 M as last having, generally, a final concentration in the solution of 10 mM. The reaction was incubated at 30 °C, 170 for 24 h on an orbital shaker. Then, the aqueous phase was acidified to pH 2 with HCl 2 M and was extracted with ethyl acetate (500 µL x 2). The organic layer was dried over $MgSO_4$ and analyzed by GC-FID on DB-1701 30 m column. During the study, each point of any graph was at least the average value obtained from three independent tests.

8.1. Reaction optimization with *Pichia finlandica* ADH

8.1.1. Buffer concentration pH 10

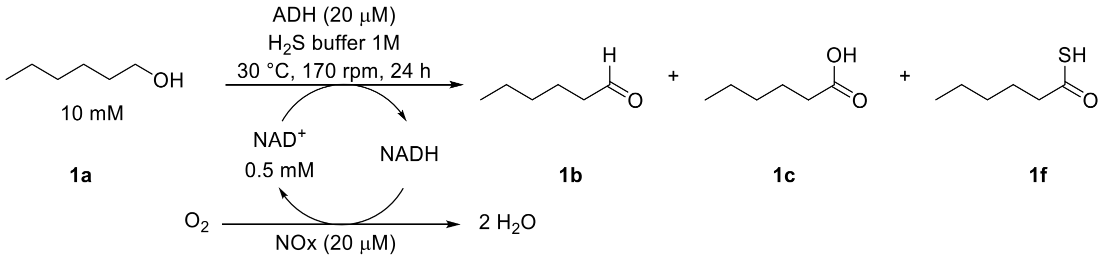

Scheme S33: First tests for the conversion of substrate 1a into thioacid 1f.

General procedure

In an Eppendorf tube (2 mL), NAD+ (0.5 mM), NOx (20 µM) and Pf-ADH (20 µM) were added in sulfuric acid buffer (varied concentration, pH 10, final volume of 1 mL). The substrate was added from a DMSO stock solution 1 M as last having, generally, a final concentration in the solution of 10 mM. The reaction was incubated at 30 °C, 800 rpm for 24 h on a thermomixer. Then, the aqueous phase was acidified to pH 2 with HCl 2 M and was extracted with ethyl acetate (500 µL x 2). The organic layer was dried over $MgSO_4$ and analyzed by GC-FID on HP-5 30 m column. During the study, each point of any graph was at least the average value obtained from three independent tests.

Table S30: Different buffer concentration for the formation of the thioacid.

Concentration [M]	Aldehyde [%]	Alcohol [%]	Carboxylic acid [%]	Thioacid [%]
0.5	8	25	53	14
1	0	0	73	27
2	0	0	70	30
3	0	0	66	34
4	0	0	60	40
5	0	89	10	1

8.1.2. pH study 1 M buffer

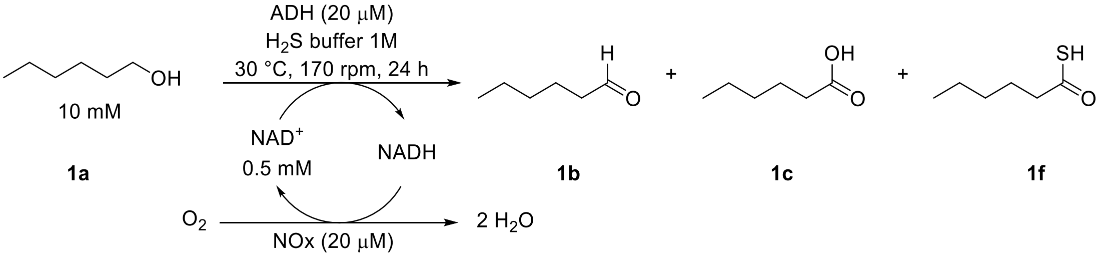

Scheme S34: First tests for the conversion of substrate 1a into thioacid 1f.

General procedure

In an Eppendorf tube (2 mL), NAD^+ (0.5 mM), NOx (20 µM) and Pf-ADH (20 µM) were added in sulfuric acid buffer (varied pH, 1 M, final volume of 1 mL). The substrate was added from a DMSO stock solution 1 M as last having, generally, a final concentration in the solution of 10 mM. The reaction was incubated at 30 °C, 800 rpm for 24 h on a thermomixer. Then, the aqueous phase was acidified to pH 2 with HCl 2 M and was extracted with ethyl acetate (500 µL x 2). The organic layer was dried over $MgSO_4$ and analyzed by GC-FID on HP-5 30 m column. During the study, each point of on any graph was at least the average value obtained from three independent tests.

Table S31: pH study with H_2S buffer concentration 1 M.

pH	Aldehyde [%]	Alcohol [%]	Carboxylic acid [%]	Thioacid [%]
6.5	5	83	2	10
7	0	0	12	88
7.5	0	0	22	78
8	0	0	31	69
9	0	0	60	40
9.5	0	0	61	39
10	0	0	65	35
10.5	0	0	68	32
11	0	0	67	33

8.1.3. Concentration buffer pH 7

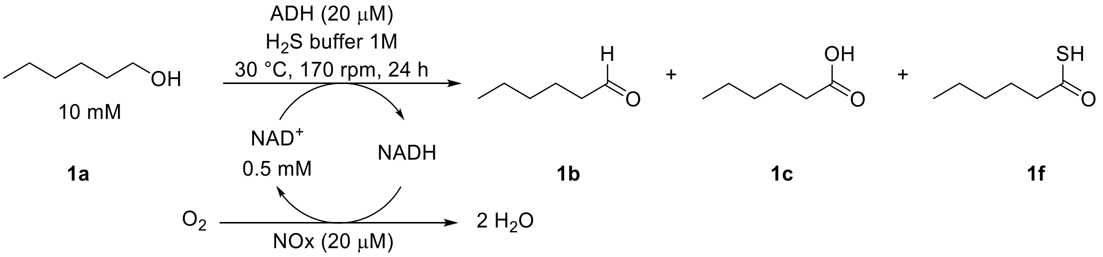

Scheme S35: First tests for the conversion of substrate 1a into thioacid 1f.

General procedure

In an Eppendorf tube (2 mL), NAD^+ (0.5 mM), NOx (20 µM) and Pf-ADH (20 µM) were added in sulfuric acid buffer (varied concentration, pH 7, final volume of 1 mL). The substrate was added from a DMSO stock solution 1 M as last having, generally, a final concentration in the solution of 10 mM. The reaction was incubated at 30 °C, 800 rpm for 24 h on a thermomixer. Then, the aqueous phase was acidified to pH 2 with HCl 2 M and was extracted with ethyl acetate (500 µL x 2). The organic layer was dried over $MgSO_4$ and analyzed by GC-FID on HP-5 30 m column. During the study, each point of on any graph was at least the average value obtained from three independent tests.

Table S32: Buffer concentration screening at pH 7.

Concentration [M]	Aldehyde [%]	Alcohol [%]	Carboxylic acid [%]	Thioacid [%]
0.1	1	23	20	56
0.3	0	0	7	93
0.5	0	0	8	92
1	0	0	12	88
1.5	1	43	1	55

8.1.4. pH study 0.3 M buffer

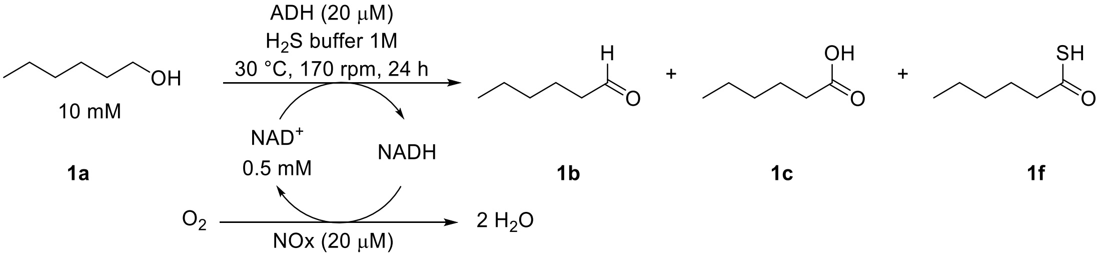

Scheme S36: First tests for the conversion of substrate 1a into thioacid 1f.

General procedure

In an Eppendorf tube (2 mL), NAD^+ (0.5 mM), NOx (20 µM) and Pf-ADH (20 µM) were added in sulfuric acid buffer (varied pH, 0.3 M, final volume of 1 mL). The substrate was added from a DMSO stock solution 1 M as last having, generally, a final concentration in the solution of 10 mM. The reaction was incubated at 30 °C, 800 rpm for 24 h on a thermomixer. Then, the aqueous phase was acidified to pH 2 with HCl 2 M and was extracted with ethyl acetate (500 µL x 2). The organic layer was dried over $MgSO_4$ and analyzed by GC-FID on HP-5 30 m column. During the study, each point of any graph was at least the average value obtained from three independent tests.

Table S33: pH study with the best buffer concentration (0.3 M).

pH	Aldehyde [%]	Alcohol [%]	Carboxylic acid [%]	Thioacid [%]
6.5	1	36	3	60
7	0	0	7	93
8	0	0	31	69
9	0	0	60	40
10	0	0	62	38
11	0	0	68	32

8.2. Reaction optimization with *Paracoccus pantotrophus ADH*

8.2.1. Buffer concentration pH 10

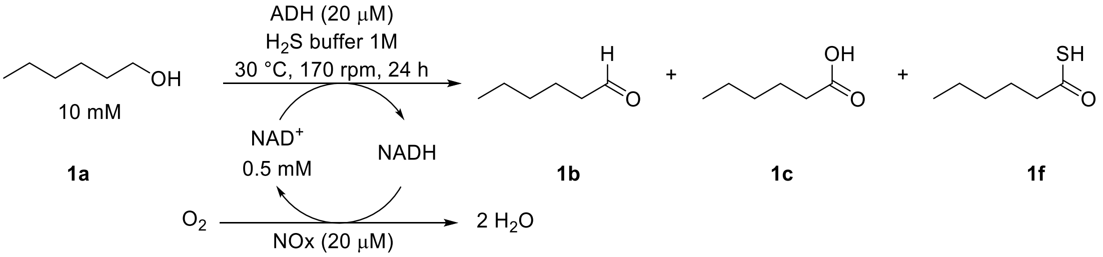

Scheme S37: First tests for the conversion of substrate 1a into thioacid 1f.

General procedure

In an Eppendorf tube (2 mL), NAD^+ (0.5 mM), NOx (20 µM) and Pf-ADH (20 µM) were added in sulfuric acid buffer (varied concentration, pH 10, final volume of 1 mL). The substrate was added from a DMSO stock solution 1 M as last having, generally, a final concentration in the solution of 10 mM. The reaction was incubated at 30 °C, 800 rpm for 24 h on a thermomixer. Then, the aqueous phase was acidified to pH 2 with HCl 2 M and was extracted

with ethyl acetate (500 µL x 2). The organic layer was dried over $MgSO_4$ and analyzed by GC-FID on HP-5 30 m column. During the study, each point of any graph was at least the average value obtained from three independent tests.

Table S34: Buffer molarity test.

Concentration [M]	Aldehyde [%]	Alcohol [%]	Carboxylic acid [%]	Thioacid [%]
0.5	4	36	41	19
1	0	0	75	25
2	0	0	69	31
3	0	0	65	35
4	0	0	54	46
5	1	9	50	40

8.2.2. pH study 1 M buffer

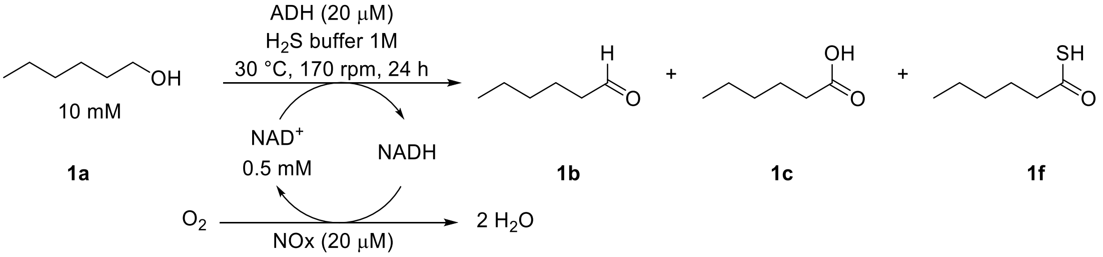

Scheme S38: First tests for the conversion of substrate 1a into thioacid 1f.

General procedure

In an Eppendorf tube (2 mL), NAD^+ (0.5 mM), NOx (20 µM) and Pf-ADH (20 µM) were added in sulfuric acid buffer (varied pH, 1 M, final volume of 1 mL). The substrate was added from a DMSO stock solution 1 M as last having, generally, a final concentration in the solution of 10 mM. The reaction was incubated at 30 °C, 800 rpm for 24 h on a thermomixer. Then, the aqueous phase was acidified to pH 2 with HCl 2 M and was extracted with ethyl acetate (500 µL x 2). The organic layer was dried over $MgSO_4$ and analyzed by GC-FID on HP-5 30 m column. During the study, each point of any graph was at least the average value obtained from three independent tests.

Table S35: pH screening with 1 M buffer.

pH	Aldehyde [%]	Alcohol [%]	Carboxylic acid [%]	Thioacid [%]
6.5	2	95	2	1
7	2	43	1	54
7.5	4	58	22	16
8	5	74	15	6
9	2	27	40	31
9.5	4	53	27	16
10	5	47	33	15
10.5	2	32	37	29
11	2	22	51	25

8.2.3. Concentration buffer pH 7

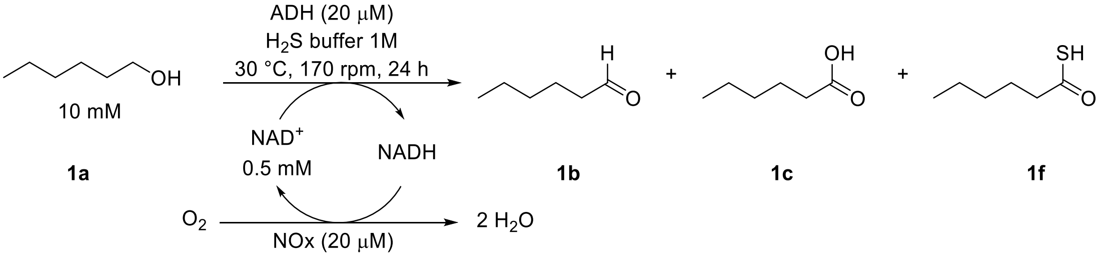

Scheme S39: First tests for the conversion of substrate 1a into thioacid 1f.

General procedure

In an Eppendorf tube (2 mL), NAD+ (0.5 mM), NOx (20 µM) and Pf-ADH (20 µM) were added in sulfuric acid buffer (varied concentration, pH 7, final volume of 1 mL). The substrate was added from a DMSO stock solution 1 M as last having, generally, a final concentration in the solution of 10 mM. The reaction was incubated at 30 °C, 800 rpm for 24 h on a thermomixer. Then, the aqueous phase was acidified to pH 2 with HCl 2 M and was extracted with ethyl acetate (500 µL x 2). The organic layer was dried over $MgSO_4$ and analyzed by GC-FID on HP-5 30 m column. During the study, each point of on any graph was at least the average value obtained from three independent tests.

Table S36: Buffer molarity screening with optimized pH.

Concentration [M]	Aldehyde [%]	Alcohol [%]	Carboxylic acid [%]	Thioacid [%]
0.1	0	0	16	84
0.3	0	1	6	93
0.5	1	14	4	81
1	2	43	1	54
1.5	2	56	1	41

8.2.4. pH study 0.3 M buffer

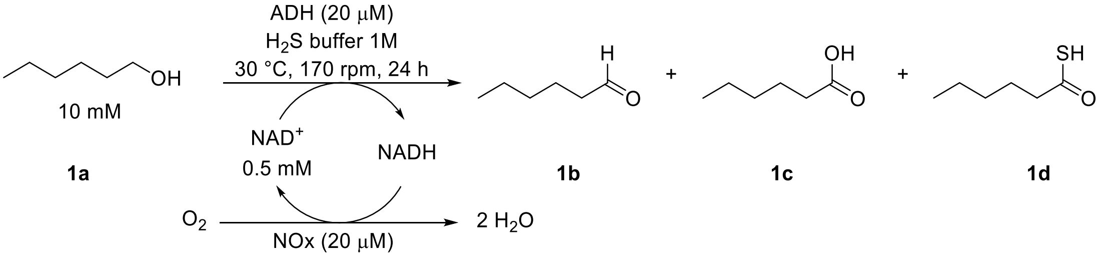

Scheme S40: First tests for the conversion of substrate 1a into thioacid 1f.

General procedure

In an Eppendorf tube (2 mL), NAD+ (0.5 mM), NOx (20 µM) and Pf-ADH (20 µM) were added in sulfuric acid buffer (varied pH, 0.3 M, final volume of 1 mL). The substrate was added from a DMSO stock solution 1 M as last having, generally, a final concentration in the solution of 10 mM. The reaction was incubated at 30 °C, 800 rpm for 24 h on a thermomixer. Then, the aqueous phase was acidified to pH 2 with HCl 2 M and was extracted with ethyl acetate (500 µL x 2). The organic layer was dried over $MgSO_4$ and analyzed by GC-FID on HP-5 30 m column. During the study, each point of any graph was at least the average value obtained from three independent tests.

Table S37: pH screening at the optimal buffer concentration.

pH	Aldehyde [%]	Alcohol [%]	Carboxylic acid [%]	Thioacid [%]
6.5	0	97	0	3
7	0	1	6	93
8	0	0	31	69
9	0	0	46	54
10	0	0	55	45
11	0	0	63	37

8.3. Reaction optimization with *Aromatoleum aromaticum* ADH

8.3.1. Buffer concentration pH 10

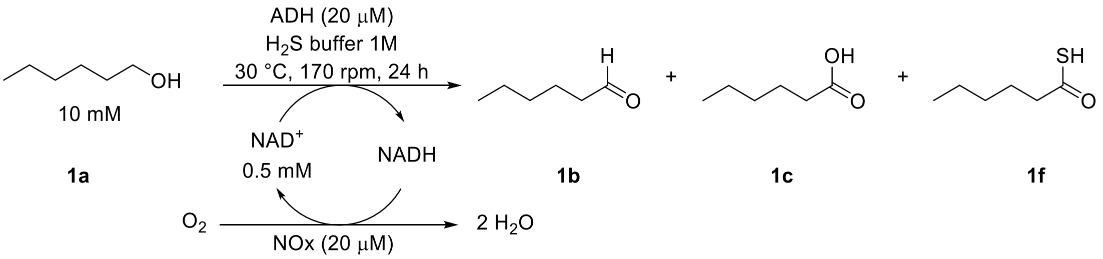

Scheme S41: First tests for the conversion of substrate 1a into thioacid 1f.

General procedure

In an Eppendorf tube (2 mL), NAD^+ (0.5 mM), NOx (20 µM) and Pf-ADH (20 µM) were added in sulfuric acid buffer (varied concentration, pH 10, final volume of 1 mL). The substrate was added from a DMSO stock solution 1 M as last having, generally, a final concentration in the solution of 10 mM. The reaction was incubated at 30 °C, 800 rpm for 24 h on a thermomixer. Then, the aqueous phase was acidified to pH 2 with HCl 2 M and was extracted with ethyl acetate (500 µL x 2). The organic layer was dried over $MgSO_4$ and analyzed by GC-FID on HP-5 30 m column. During the study, each point of any graph was at least the average value obtained from three independent tests.

Table S38: Concentration buffer screen at pH 10.

Concentration [M]	Aldehyde [%]	Alcohol [%]	Carboxylic acid [%]	Thioacid [%]
0.5	2	59	39	0
1	0	46	20	34
2	1	55	27	17
3	2	72	19	7
4	2	76	17	5
5	1	89	9	1

8.3.2. pH study 1 M buffer

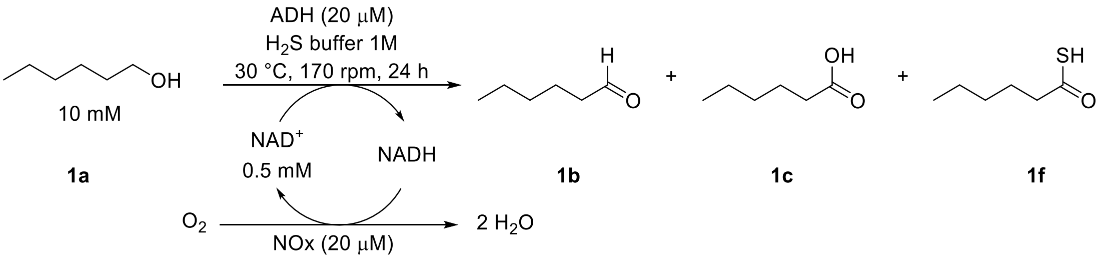

Scheme S42: First tests for the conversion of substrate 1a into thioacid 1f.

General procedure

In an Eppendorf tube (2 mL), NAD^+ (0.5 mM), NOx (20 µM) and Pf-ADH (20 µM) were added in sulfuric acid buffer (varied pH, 1 M, final volume of 1 mL). The substrate was added from a DMSO stock solution 1 M as last having, generally, a final concentration in the solution of 10 mM. The reaction was incubated at 30 °C, 800 rpm for 24 h on a thermomixer. Then, the aqueous phase was acidified to pH 2 with HCl 2 M and was extracted with ethyl acetate (500 µL x 2). The organic layer was dried over $MgSO_4$ and analyzed by GC-FID on HP-5 30 m column. During the study, each point of on any graph was at least the average value obtained from three independent tests.

Table S39: pH screening with 1 M buffer.

pH	Aldehyde [%]	Alcohol [%]	Carboxylic acid [%]	Thioacid [%]
6.5	5	83	2	0
7	0	26	4	70
7,5	1	30	24	45
8	1	51	9	39
9	2	37	29	32
9.5	4	54	34	8
10	4	55	30	11
10.5	2	43	28	27
11	2	41	37	20

8.3.3. Concentration buffer pH 7

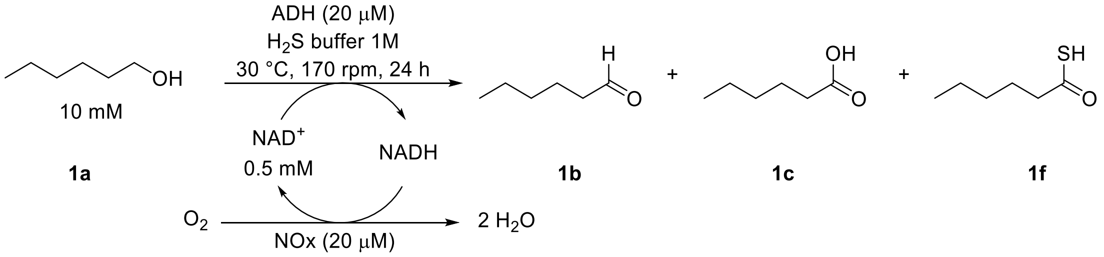

Scheme S43: First tests for the conversion of substrate 1a into thioacid 1f.

General procedure

In an Eppendorf tube (2 mL), NAD^+ (0.5 mM), NOx (20 µM) and Pf-ADH (20 µM) were added in sulfuric acid buffer (varied concentration, pH 7, final volume of 1 mL). The substrate was added from a DMSO stock solution 1 M as last having, generally, a final concentration in the solution of 10 mM. The reaction was incubated at 30 °C, 800 rpm for 24 h on a thermomixer. Then, the aqueous phase was acidified to pH 2 with HCl 2 M and was extracted with ethyl acetate (500 µL x 2). The organic layer was dried over $MgSO_4$ and analyzed by GC-FID on HP-5 30 m column. During the study, each point of on any graph was at least the average value obtained from three independent tests.

Table S40: Molarity buffer screening pH 7.

Concentration [M]	Aldehyde [%]	Alcohol [%]	Carboxylic acid [%]	Thioacid [%]
0.1	0	8	20	72
0.3	0	0	9	91
0.5	0	0	8	92
1	0	26	4	70
1.5	1	34	13	52

8.3.4. pH study 0.3 M buffer

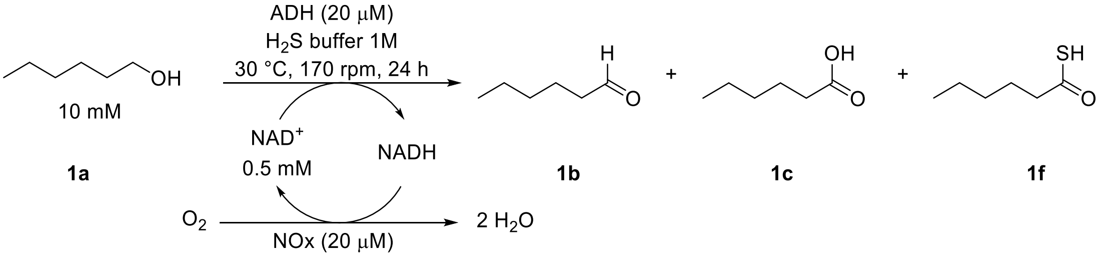

Scheme S44: First tests for the conversion of substrate 1a into amide 1d.

General procedure

In an Eppendorf tube (2 mL), NAD+ (0.5 mM), NOx (20 µM) and Pf-ADH (20 µM) were added in sulfuric acid buffer (varied pH, 0.3 M, final volume of 1 mL). The substrate was added from a DMSO stock solution 1 M as last having, generally, a final concentration in the solution of 10 mM. The reaction was incubated at 30 °C, 800 rpm for 24 h on a thermomixer. Then, the aqueous phase was acidified to pH 2 with HCl 2 M and was extracted with ethyl acetate (500 µL x 2). The organic layer was dried over $MgSO_4$ and analyzed by GC-FID on HP-5 30 m column. During the study, each point of on any graph was at least the average value obtained from three independent tests.

Table S41: pH screening with 0.3 M buffer.

pH	Aldehyde [%]	Alcohol [%]	Carboxylic acid [%]	Thioacid [%]
6.5	0	51	1	48
7	0	0	9	91
8	1	50	9	40
9	1	51	30	18
10	2	56	26	16
11	1	71	20	8

9. Methylthioester synthesis

9.1. Pf-ADH

9.1.1. Buffer concentration pH 7

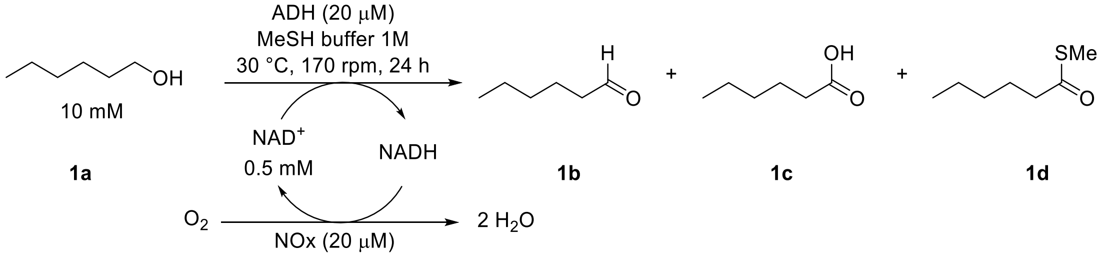

Scheme S45: First tests for the conversion of substrate 1a into thioester 1g.

General procedure

In an Eppendorf tube (2 mL), NAD^+ (0.5 mM), NOx (20 µM) and Pf-ADH (20 µM) were added in methyl mercaptan buffer (varied concentration, pH 7, final volume of 1 mL). The substrate was added from a DMSO stock solution 1 M as last having, generally, a final concentration in the solution of 10 mM. The reaction was incubated at 30 °C, 800 rpm for 24 h on a thermomixer. Then, the aqueous phase was acidified to pH 2 with HCl 2 M and was extracted with ethyl acetate (500 µL x 2). The organic layer was dried over $MgSO_4$ and analyzed by GC-FID on HP-5 30 m column. During the study, each point of on any graph was at least the average value obtained from three independent tests.

Table S42: Concentration buffer screening at pH 7.

Conc. [M]	Aldehyde [%]	Alcohol [%]	Carboxylic acid [%]	Thioacid [%]	Thioester [%]
1	0	0	32	41	27
0.5	5	32	36	17	9
0.3	0	0	45	46	19
0.1	10	64	21	3	2

9.2. Pp-ADH

9.2.1. Buffer concentration pH 7

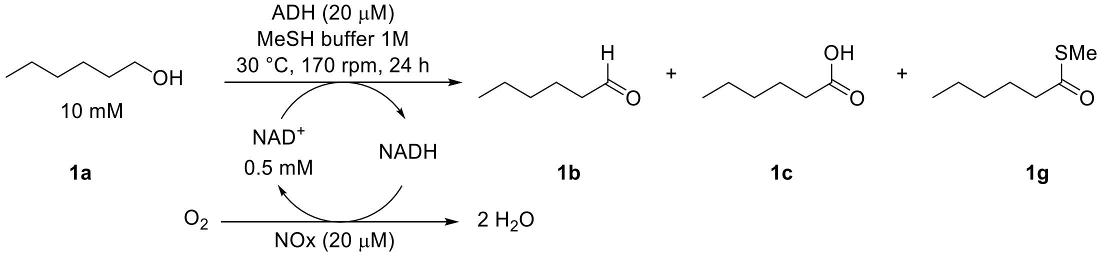

Scheme S46: First tests for the conversion of substrate 1a into thioester 1g.

General procedure

In an Eppendorf tube (2 mL), NAD^+ (0.5 mM), NOx (20 µM) and Pf-ADH (20 µM) were added in methyl mercaptan buffer (varied concentration, pH 7, final volume of 1 mL). The substrate was added from a DMSO stock solution 1 M as last having, generally, a final concentration in the solution of 10 mM. The reaction was incubated at 30 °C, 800 rpm for 24 h on a thermomixer. Then, the aqueous phase was acidified to pH 2 with HCl 2 M and was extracted with ethyl acetate (500 µL x 2). The organic layer was dried over $MgSO_4$ and analyzed by GC-FID on HP-5 30 m column. During the study, each point of on any graph was at least the average value obtained from three independent tests.

Table S43: Concentration buffer screening at pH 7.

Conc. [M]	Aldehyde [%]	Alcohol [%]	Carboxylic acid [%]	Thioacid [%]	Thioester [%]
1	0	0	0	5	95
0.5	0	49	2	3	46
0.3	0	68	1	2	28
0.1	1	86	2	1	10

9.3. Aa-ADH

9.3.1. Buffer concentration

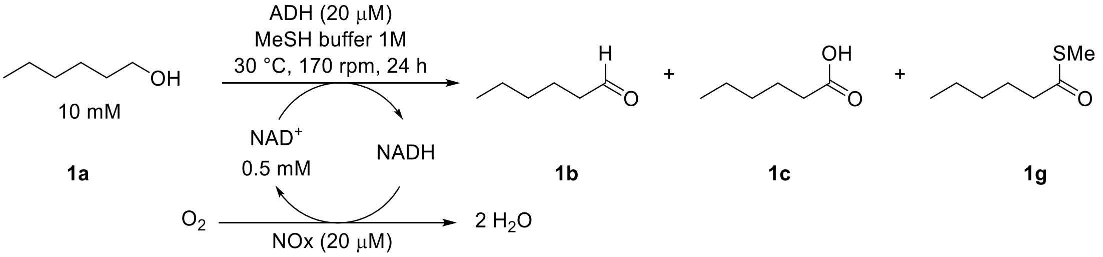

Scheme S47: First tests for the conversion of substrate 1a into thioester 1g.

General procedure

In an Eppendorf tube (2 mL), NAD^+ (0.5 mM), NOx (20 µM) and Pf-ADH (20 µM) were added in methyl mercaptan buffer (varied concentration, pH 7, final volume of 1 mL). The substrate was added from a DMSO stock solution 1 M as last having, generally, a final concentration in the solution of 10 mM. The reaction was incubated at 30 °C, 800 rpm for 24 h on a thermomixer. Then, the aqueous phase was acidified to pH 2 with HCl 2 M and was extracted with ethyl acetate (500 µL x 2). The organic layer was dried over $MgSO_4$ and analyzed by GC-FID on HP-5 30 m column. During the study, each point of any graph was at least the average value obtained from three independent tests.

Table S44: Concentration buffer screening at pH 7.

Conc. [M]	Aldehyde [%]	Alcohol [%]	Carboxylic acid [%]	Thioacid [%]	Thioester [%]
1		83		1	16
0.5		76		1	23
0.3		64	1	1	34
0.1	3	60	11		26

10. Thioesters (EtSH)

10.1. Pf-ADH

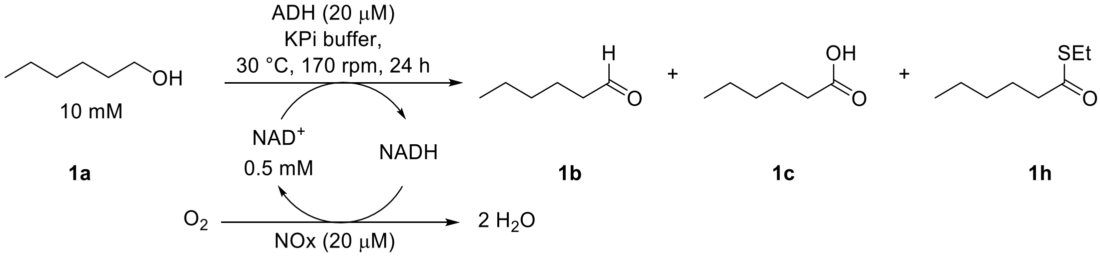

Scheme S48: First tests for the conversion of substrate 1a into thioester 1h.

General procedure
In an Eppendorf tube (2 mL), NAD^+ (0.5 mM), NOx (20 µM) and Pf-ADH (20 µM) were added in potassium phosphate buffer (varied concentration, pH 7, final volume 1 mL) or a mixture 1:1 of potassium phosphate buffer and heptane, final volume of 1 mL. The substrate was added from a DMSO stock solution 1 M, a final concentration in the solution of 10 mM. Finally, the thiol was added (0.5 M concentration). The reaction was incubated at 30 °C, 800 rpm for 24 h on a thermomixer. Then, the aqueous phase was acidified to pH 2 with HCl 2 M, the heptane phase was separated, the aqueous buffer was extracted with ethyl acetate (350 µL x 2) and the organic phases were combined. The organic layer was dried over $MgSO_4$ and analyzed by GC-FID on HP-5 30 m column. During the study, each point of any graph was at least the average value obtained from three independent tests.

Table S45: Optimization in monophasic and biphasic system.

Pf-ADH	Aldehyde [%]	Alcohol [%]	Carb. Acid [%]	Thioester [%]
KPi 500 mM monophasic	28	55	17	1
KPi 200 mM monophasic	7	93	-	-
KPi 100 mM monophasic	7	93	-	-
KPi 500 mM biphasic	20	16	62	1
KPi 200 mM biphasic	32	-	62	1
KPi 100 mM biphasic	35	64	-	1
KPi 500 mM 1 M EtSH	6	94	-	-
KPi 500 mM 0.1 M EtSH	15	80	5	-

10.2. Pp-ADH

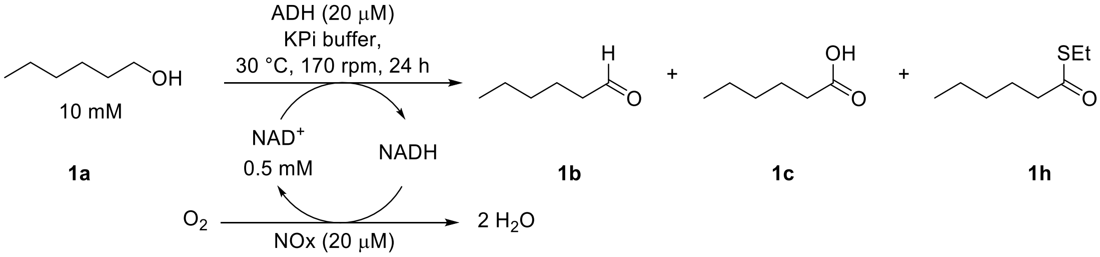

Scheme S49: First tests for the conversion of substrate 1a into thioester 1h.

General procedure
In an Eppendorf tube (2 mL), NAD^+ (0.5 mM), NOx (20 µM) and Pf-ADH (20 µM) were added in potassium phosphate buffer (varied concentration, pH 7, final volume 1 mL) or a mixture 1:1 of potassium phosphate buffer and heptane, final volume of 1 mL. The substrate was added from a DMSO stock solution 1 M, a final concentration in the solution of 10 mM. Finally, the thiol was added (0.5 M concentration). The reaction was incubated at 30 °C, 800 rpm for 24 h on a thermomixer. Then, the aqueous phase was acidified to pH 2 with HCl 2 M, the heptane phase was separated, the aqueous buffer was extracted with ethyl acetate (350 µL x 2) and the organic phases were combined. The organic layer was dried over $MgSO_4$ and analyzed by GC-FID on HP-5 30 m

column. During the study, each point of any graph was at least the average value obtained from three independent tests.

Table S46: Optimization in monophasic and biphasic system.

Pp-ADH	Aldehyde [%]	Alcohol [%]	Carb. Acid [%]	Thioester [%]
KPi 500 mM monophasic	1	90	-	9
KPi 200 mM monophasic	1	96	-	3
KPi 100 mM monophasic	1	97	-	2
KPi 500 mM biphasic	7	32	3	57
KPi 200 mM biphasic	8	42	3	47
KPi 100 mM biphasic	8	51	2	39
KPi 500 mM 1 M EtSH	-	100	-	-
KPi 500 mM 0.1 M EtSH	1	95	1	3

Afterwards, the concentration of the thiol was screened (0.1, 0.2, 0.3, and 0.5 M) in the optimal conditions (biphasic system, KPi buffer 500 mM pH 7 and heptane)

Table S47: increasing thiol concentration.

Pp-ADH	Aldehyde [%]	Alcohol [%]	Carb. Acid [%]	Thioester [%]
0.1 M EtSH	12	28	17	43
0.2 M EtSH	10	26	12	52
0.3 M EtSH	10	27	11	53
0.5 M EtSH	7	32	3	57

10.3. Aa-ADH

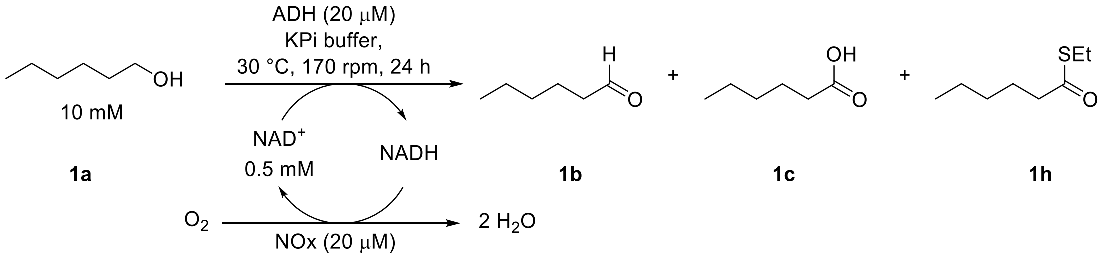

Scheme S50: First tests for the conversion of substrate 1a into thioester 1h.

General procedure

In an Eppendorf tube (2 mL), NAD^+ (0.5 mM), NOx (20 µM) and Pf-ADH (20 µM) were added in potassium phosphate buffer (varied concentration, pH 7, final volume 1 mL) or a mixture 1:1 of potassium phosphate buffer and heptane, final volume of 1 mL. The substrate was added from a DMSO stock solution 1 M, a final concentration in the solution of 10 mM. Finally, the thiol was added (0.5 M concentration). The reaction was incubated at 30 °C, 800 rpm for 24 h on a thermomixer. Then, the aqueous phase was acidified to pH 2 with HCl 2 M, the heptane phase was separated, the aqueous buffer was extracted with ethyl acetate (350 µL x 2) and the organic phases were combined. The organic layer was dried over $MgSO_4$ and analyzed by GC-FID on HP-5 30 m column. During the study, each point of any graph was at least the average value obtained from three independent tests.

Table S48: Optimization in monophasic and biphasic system.

Aa-ADH	Aldehyde [%]	Alcohol [%]	Carb. Acid [%]	Thioester [%]
KPi 500 mM monophasic	2	90	-	8
KPi 200 mM monophasic	2	92	-	6
KPi 100 mM monophasic	2	93	-	5
KPi 500 mM biphasic	15	61	3	21
KPi 200 mM biphasic	16	64	3	17
KPi 100 mM biphasic	15	65	4	16
KPi 500 mM 1 M EtSH	1	98	-	1
KPi 100 mM 0.1 M EtSH	2	90	2	6

Afterwards, the concentration of the thiol was screened (0.1, 0.2, 0.3, and 0.5 M) in the optimal conditions (biphasic system, KPi buffer 500 mM pH 7 and heptane)

Table S49: Increasing thiol concentration.

Aa-ADH	Aldehyde [%]	Alcohol [%]	Carb. Acid [%]	Thioester [%]
0.1 M EtSH	22	48	18	12
0.2 M EtSH	18	53	14	15
0.3 M EtSH	17	56	9	18
0.5 M EtSH	15	61	3	21

11. Longer chain thiols screening

Using the optimized condition found in the previous section (KPi 500 mM buffer, pH 7, RSH 0.5 M), we tested longer thiols with the mutants from Aa-ADH WT, (Y93A, Y151A and L186A) using three model substrates (1a, 10a and 14a).

Table S50: Longer thiols tested with substrate 1a, 10a and 14a.

Substrate 1a	EtSH	n-PrSH	n-BuSH	S-iPrSH
Y93A	traces	-	-	-
Y151A	53	41	7	7
L186A	51	9	5	-

Substrate 14a	EtSH	n-PrSH	n-BuSH	S-iPrSH
Y93A	traces	-	-	-
Y151A	44	46	68	12
L186A	43	44	12	11

Substrate 10a	EtSH	n-PrSH	n-BuSH	S-iPrSH
Y93A	-	-	-	-
Y151A		-	-	-
L186A	43	13	-	1

12. Substrate scope

12.1. Amides

Table S51: Substrate 1a in ammonium formate buffer.

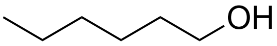

1a

1a NH3	Ald	Alc	Acid	Amide
Pf-ADH	0	0	11	89
Pp-ADH	0	0	19	81
AA-ADH	0	52	3	45
Te-ADH	1	36	48	15
Ht-ADH	13	0	73	12

Table S52: Substrate 2a in ammonium formate buffer.

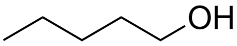

2a

2a NH3	Ald	Alc	Acid	Amide
Pf-ADH	0	0	14	86
Pp-ADH	0	0	30	70
AA-ADH	0	65	3	32
Te-ADH	1	90	7	2
Ht-ADH	72	1	14	13

Table S53: Substrate 3a in ammonium formate buffer.

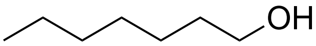

3a

3a NH3	Ald	Alc	Acid	Amide
Pf-ADH	1	0	9	90
Pp-ADH	2	0	16	82
AA-ADH	2	90	1	7
Te-ADH	5	73	12	10
Ht-ADH	75	1	11	13

Table S54: Substrate 4a in ammonium formate buffer.

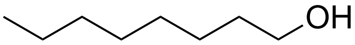

4a

4a NH3	Ald	Alc	Acid	Amide
Pf-ADH	1	0	11	88
Pp-ADH	1	5	14	80
AA-ADH	1	82	1	16
Te-ADH	1	72	17	10
Ht-ADH	89	0	6	5

Table S55: Substrate 5a in ammonium formate buffer.

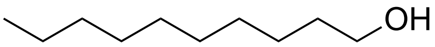

5a

5a NH3	Ald	Alc	Acid	Amide
Pf-ADH	38	48	1	13
Pp-ADH	1	5	21	73
AA-ADH	1	32	1	66
Te-ADH	7	72	13	8
Ht-ADH	94	0	4	2

Table S56: Substrate 6a in ammonium formate buffer.

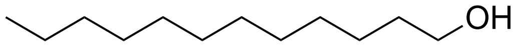

6a

6a NH3	Ald	Alc	Acid	Amide
Pf-ADH	9	90	0	1
Pp-ADH	13	44	9	34
AA-ADH	1	47	1	51
Te-ADH	6	72	13	9
Ht-ADH	100	0	0	0

Table S57: Substrate 7a in ammonium formate buffer.

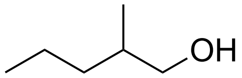

7a

7a NH3	Ald	Alc	Acid	Amide
Pf-ADH	4	91	0	5
Pp-ADH	0	1	44	55
AA-ADH	0	10	10	80
Te-ADH	2	90	7	1
Ht-ADH	27	71	1	1

Table S58: Substrate 8a in ammonium formate buffer.

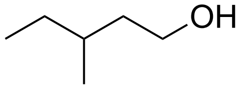

8a

8a NH3	Ald	Alc	Acid	Amide
Pf-ADH	7	90	0	3
Pp-ADH	1	10	27	62
AA-ADH	2	81	2	15
Te-ADH	3	86	9	2
Ht-ADH	32	66	1	1

Table S59: Substrate 9a in ammonium formate buffer.

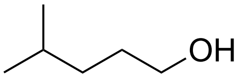

9a

9a NH3	Ald	Alc	Acid	Amide
Pf-ADH	4	11	15	70
Pp-ADH	0	0	21	79
AA-ADH	0	80	1	19
Te-ADH	4	84	8	4
Ht-ADH	82	0	2	16

Table S60: Substrate 10a in ammonium formate buffer.

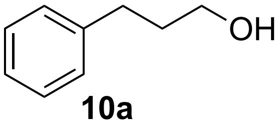

10a

10a NH3	Ald	Alc	Acid	Amide
Pf-ADH	0	0	5	95
Pp-ADH	0	0	20	80
AA-ADH	0	93	0	7
Te-ADH	1	81	11	7
Ht-ADH	0	94	3	3

Table S61: Substrate 11a in ammonium formate buffer.

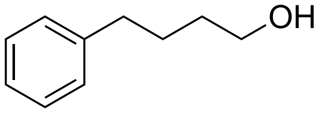

11a

11a NH3	Ald	Alc	Acid	Amide
Pf-ADH	5	94	0	1
Pp-ADH	2	92	0	6
AA-ADH	1	98	0	1
Te-ADH	1	93	3	3
Ht-ADH	94	0	3	3

Table S62: Substrate 12a in ammonium formate buffer

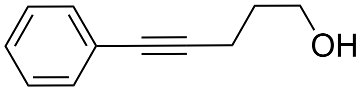

12a

12a NH3	Ald	Alc	Acid	Amide
Pf-ADH	2	70	0	28
Pp-ADH	2	88	0	10
AA-ADH	0	97	0	3
Te-ADH	0	100	0	0
Ht-ADH	0	100	0	0

Table S63: Substrate 13a in ammonium formate buffer.

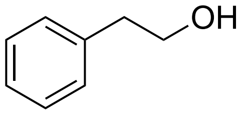

13a

13a NH3	Ald	Alc	Acid	Amide
Pf-ADH	1	99	0	0
Pp-ADH	1	87	1	11
AA-ADH	1	98	0	1
Te-ADH	1	95	2	2
Ht-ADH	1	99	0	0

Table S64: Substrate 14a in ammonium formate buffer.

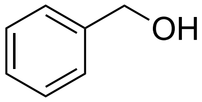

14a

14a NH3	Ald	Alc	Acid	Amide
Pf-ADH	8	91	0	1
Pp-ADH	8	52	5	35
AA-ADH	0	0	3	97
Te-ADH	1	99	0	0
Ht-ADH	1	99	0	0

Table S65: Substrate 15a in ammonium formate buffer.

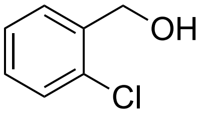

15a

15a NH3	Ald	Alc	Acid	Amide
Pf-ADH	0	95	0	5
Pp-ADH	6	91	0	3
AA-ADH	1	59	0	40
Te-ADH	0	100	0	0
Ht-ADH	0	100	0	0

Table S66: Substrate 16a in ammonium formate buffer.

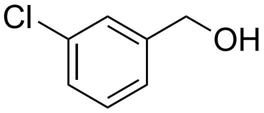

16a

16a NH3	Ald	Alc	Acid	Amide
Pf-ADH	11	85	0	4
Pp-ADH	7	89	0	4
AA-ADH	1	87	0	12
Te-ADH	1	99	0	0
Ht-ADH	39	61	0	0

Table S67: Substrate 17a in ammonium formate buffer.

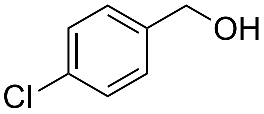

17a

17a NH3	Ald	Alc	Acid	Amide
Pf-ADH	8	88	0	4
Pp-ADH	10	82	0	8
AA-ADH	1	87	0	12
Te-ADH	1	99	0	0
Ht-ADH	82	18	0	0

Table S68: Substrate 18a in ammonium formate buffer.

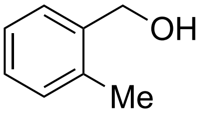

18a

18a NH3	Ald	Alc	Acid	Amide
Pf-ADH	0	100	0	0
Pp-ADH	21	73	0	6
AA-ADH	1	72	0	27
Te-ADH	0	100	0	0
Ht-ADH	0	100	0	0

Table S69: Substrate 19a in ammonium formate buffer.

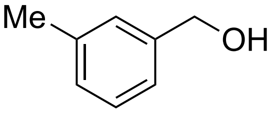

19a

19a NH3	Ald	Alc	Acid	Amide
Pf-ADH	15	84	0	1
Pp-ADH	16	79	0	5
AA-ADH	1	75	0	24
Te-ADH	0	100	0	0
Ht-ADH	69	31	0	0

Table S70: Substrate 20a in ammonium formate buffer.

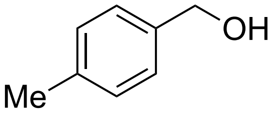

20a

20a NH3	Ald	Alc	Acid	Amide
Pf-ADH	15	83	0	2
Pp-ADH	31	56	0	13
AA-ADH	1	66	0	33
Te-ADH	2	98	0	0
Ht-ADH	100	0	0	0

Table S71: substrate 21a in ammonium formate buffer.

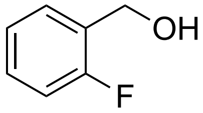

21a

21a NH3	Ald	Alc	Acid	Amide
Pf-ADH	0	100	0	0
Pp-ADH	2	80	0	18
AA-ADH	0	3	0	97
Te-ADH	0	100	0	0
Ht-ADH	29	71	0	0

Table S72: Substrate 22a in ammonium formate buffer.

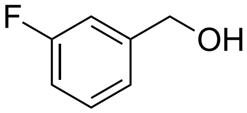

22a

22a NH3	Ald	Alc	Acid	Amide
Pf-ADH	8	87	0	5
Pp-ADH	4	40	4	52
AA-ADH	0	25	0	75
Te-ADH	0	100	0	0
Ht-ADH	22	78	0	0

Table S73: substrate 23a in ammonium formate buffer.

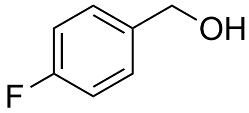

23a

23a NH3	Ald	Alc	Acid	Amide
Pf-ADH	11	86	0	3
Pp-ADH	17	67	0	16
AA-ADH	0	51	0	49
Te-ADH	0	100	0	0
Ht-ADH	100	0	0	0

Table S74: Substrate 24a in ammonium formate buffer.

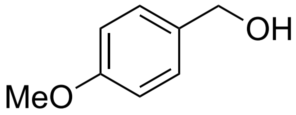

24a

24a NH3	Ald	Alc	Acid	Amide
Pf-ADH	19	81	0	0
Pp-ADH	17	67	0	16
AA-ADH	4	54	0	42
Te-ADH	1	99	0	0
Ht-ADH	0	100	0	0

Table S75: Substrate 25a in ammonium formate buffer.

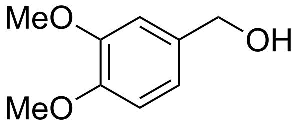

25a

25a NH3	Ald	Alc	Acid	Amide
Pf-ADH	3	42	0	55
Pp-ADH	24	76	0	0
AA-ADH	52	37	0	11
Te-ADH	2	98	0	0
Ht-ADH	45	55	0	0

Table S76: Substrate 26a in ammonium formate buffer.

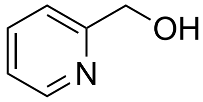

26a

26a NH3	Ald	Alc	Acid	Amide
Pf-ADH	0	100	0	0
Pp-ADH	0	97	0	3
AA-ADH	0	33	0	67
Te-ADH	8	92	0	0
Ht-ADH	54	46	0	0

Table S77: Substrate 27a in ammonium formate buffer.

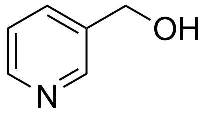

27a

27a NH3	Ald	Alc	Acid	Amide
Pf-ADH	0	100	0	0
Pp-ADH	0	100	0	0
AA-ADH	0	32	0	68
Te-ADH	0	100	0	0
Ht-ADH	100	0	0	0

Table S78: Substrate 28a in ammonium formate buffer.

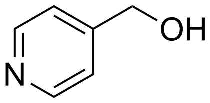

28a

28a NH3	Ald	Alc	Acid	Amide
Pf-ADH	0	100	0	0
Pp-ADH	0	100	0	0
AA-ADH	0	40	0	60
Te-ADH	2	98	0	0
Ht-ADH	42	58	0	0

MeNH$_2$

Table S79: Substrate 1a in methylammonium formate buffer.

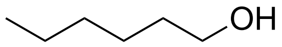

1a

1a MeNH2	Ald	Alc	Acid	Amide
Pf-ADH	1	4	1	94
Pp-ADH	2	8	1	89
AA-ADH	1	89	0	11
Te-ADH	2	56	18	24
Ht-ADH	100	0	0	0

Table S80: Substrate 2a in methylammonium formate buffer.

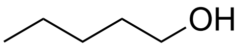

2a

2a MeNH2	Ald	Alc	Acid	Amide
Pf-ADH	4	33	0	63
Pp-ADH	2	46	7	45
AA-ADH	3	82	0	15
Te-ADH	2	77	11	10
Ht-ADH	100	0	0	0

Table S81: Substrate 3a in methylammonium formate buffer.

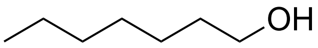

3a

3a MeNH2	Ald	Alc	Acid	Amide
Pf-ADH	5	53	0	42
Pp-ADH	7	0	11	82
AA-ADH	1	93	0	6
Te-ADH	4	71	10	15
Ht-ADH	100	0	0	0

Table S82: Substrate 4a in methylammonium formate buffer.

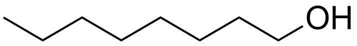

4a

4a MeNH2	Ald	Alc	Acid	Amide
Pf-ADH	10	30	0	60
Pp-ADH	18	0	11	71
AA-ADH	1	95	0	4
Te-ADH	9	56	16	19
Ht-ADH	100	0	0	0

Table S83: Substrate 5a in methylammonium formate buffer.

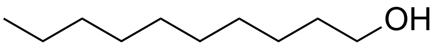

5a

5a MeNH2	Ald	Alc	Acid	Amide
Pf-ADH	1	1	1	97
Pp-ADH	9	27	7	57
AA-ADH	2	92	0	6
Te-ADH	9	62	12	17
Ht-ADH	100	0	0	0

Table S84: Substrate 6a in methylammonium formate buffer.

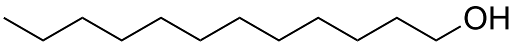

6a

6a MeNH2	Ald	Alc	Acid	Amide
Pf-ADH	5	87	0	8
Pp-ADH	6	86	1	7
AA-ADH	4	81	0	15
Te-ADH	8	81	3	8
Ht-ADH	100	0	0	0

Table S85: Substrate 7a in methylammonium formate buffer.

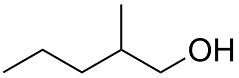

7a

7a MeNH2	Ald	Alc	Acid	Amide
Pf-ADH	4	66	0	30
Pp-ADH	2	0	43	55
AA-ADH	2	84	1	13
Te-ADH	10	72	17	1
Ht-ADH	21	79	0	0

Table S86: Substrate 8a in methylammonium formate buffer.

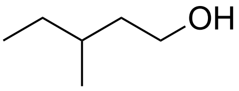

8a

8a MeNH2	Ald	Alc	Acid	Amide
Pf-ADH	8	76	0	16
Pp-ADH	39	34	9	18
AA-ADH	8	90	0	2
Te-ADH	6	71	18	5
Ht-ADH	48	52	0	0

Table S87: Substrate 9a in methylammonium formate buffer.

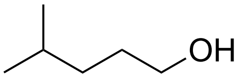

9a

9a MeNH2	Ald	Alc	Acid	Amide
Pf-ADH	1	0	4	95
Pp-ADH	10	15	11	64
AA-ADH	2	93	0	5
Te-ADH	0	71	10	19
Ht-ADH	100	0	0	0

Table S88: Substrate 10a in methylammonium formate buffer.

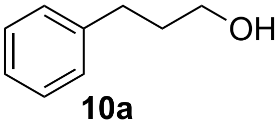

10a

10a MeNH2	Ald	Alc	Acid	Amide
Pf-ADH	2	54	0	44
Pp-ADH	4	74	2	20
AA-ADH	0	97	0	3
Te-ADH	1	83	5	11
Ht-ADH	100	0	0	0

Table S89: Substrate 11a in methylammonium formate buffer.

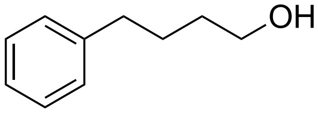

11a

11a MeNH2	Ald	Alc	Acid	Amide
Pf-ADH	3	90	0	7
Pp-ADH	3	94	0	3
AA-ADH	1	98	0	1
Te-ADH	1	84	4	11
Ht-ADH	61	39	0	0

Table S90: Substrate 12a in methylammonium formate buffer.

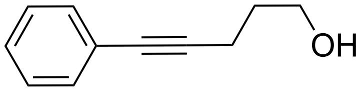

12a

12a MeNH2	Ald	Alc	Acid	Amide
Pf-ADH	2	82	0	16
Pp-ADH	3	87	0	10
AA-ADH	1	97	0	2
Te-ADH	3	91	2	4
Ht-ADH	30	70	0	0

Table S91: Substrate 13a in methylammonium formate buffer.

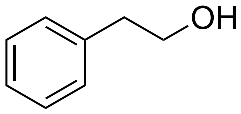

13a

13a MeNH2	Ald	Alc	Acid	Amide
Pf-ADH	0	100	0	0
Pp-ADH	3	96		1
AA-ADH	1	99	0	0
Te-ADH	1	93	3	3
Ht-ADH	100	0	0	0

Table S92: Substrate 14a in methylammonium formate buffer.

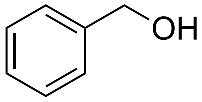

14a

14a MeNH2	Ald	Alc	Acid	Amide
Pf-ADH	13	84	0	3
Pp-ADH	41	50	0	9
AA-ADH	1	88	0	11
Te-ADH	1	99	0	0
Ht-ADH	100	0	0	0

Table S93: Substrate 15a in methylammonium formate buffer.

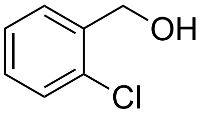

15a

15a MeNH2	Ald	Alc	Acid	Amide
Pf-ADH	0	100	0	0
Pp-ADH	1	99	0	0
AA-ADH	11	76	0	13
Te-ADH	0	100	0	0
Ht-ADH	100	0	0	0

Table S94: Substrate 16a in methylammonium formate buffer.

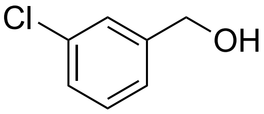

16a

16a MeNH2	Ald	Alc	Acid	Amide
Pf-ADH	0	100	0	0
Pp-ADH	1	99	0	0
AA-ADH	11	76	0	13
Te-ADH	0	100	0	0
Ht-ADH	100	0	0	0

Table S95: Substrate 17a in methylammonium formate buffer.

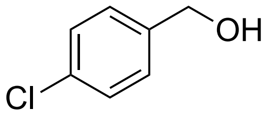

17a

17a MeNH2	Ald	Alc	Acid	Amide
Pf-ADH	5	93	0	2
Pp-ADH	14	85	0	1
AA-ADH	24	20	0	56
Te-ADH	1	99	0	0
Ht-ADH	100	0	0	0

Table S96: Substrate 18a in methylammonium formate buffer.

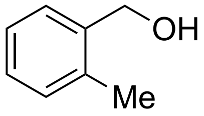

18a

18a MeNH2	Ald	Alc	Acid	Amide
Pf-ADH	0	100	0	0
Pp-ADH	8	92	0	0
AA-ADH	47	34	0	19
Te-ADH	0	100	0	0
Ht-ADH	100	0	0	0

Table S97: Substrate 19a in methylammonium formate buffer.

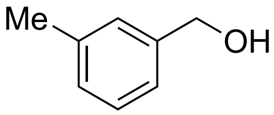

19a

19a MeNH2	Ald	Alc	Acid	Amide
Pf-ADH	41	9	0	50
Pp-ADH	22	77	0	1
AA-ADH	10	0	0	90
Te-ADH	1	99	0	0
Ht-ADH	26	74	0	0

Table S98: Substrate 20a in methylammonium formate buffer.

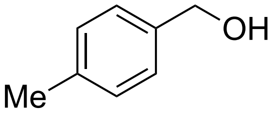

20a

20a MeNH3	Ald	Alc	Acid	a
Pf-ADH	28	60	0	12
Pp-ADH	36	64	0	2
AA-ADH	0	0	0	100
Te-ADH	1	99	0	0
Ht-ADH	100	0	0	0

Table S99: Substrate 21a in methylammonium formate buffer.

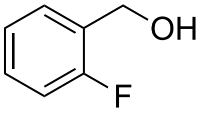

21a

21a MeNH2	Ald	Alc	Acid	Amide
Pf-ADH	0	100	0	0
Pp-ADH	5	94	0	1
AA-ADH	1	80	0	19
Te-ADH	0	100	0	0
Ht-ADH	100	0	0	0

Table S100: Substrate 22a in methylammonium formate buffer.

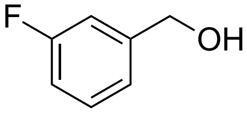

22a

22a MeNH2	Ald	Alc	Acid	Amide
Pf-ADH	13	82	0	5
Pp-ADH	22	69	0	9
AA-ADH	2	79	0	19
Te-ADH	0	100	0	0
Ht-ADH	81	19	0	0

Table S101: Substrate 23a in methylammonium formate buffer.

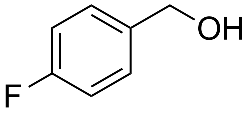

23a

23a MeNH2	Ald	Alc	Acid	Amide
Pf-ADH	5	94	0	1
Pp-ADH	37	58	0	5
AA-ADH	2	82	0	16
Te-ADH	1	99	0	0
Ht-ADH	100	0	0	0

Table S102: Substrate 24a in methylammonium formate buffer.

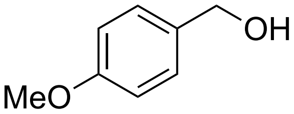

24a

24a MeNH2	Ald	Alc	Acid	Amide
Pf-ADH	31	67	0	2
Pp-ADH	64	33	1	2
AA-ADH	28	54	0	18
Te-ADH	1	99	0	0
Ht-ADH	100	0	0	0

Table S103: Substrate 25a in methylammonium formate buffer.

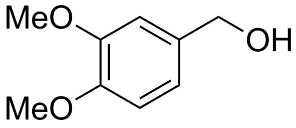

25a

25a MeNH2	Ald	Alc	Acid	Amide
Pf-ADH	5	95	0	0
Pp-ADH	9	91	0	0
AA-ADH	39	57	0	4
Te-ADH	2	98	0	0
Ht-ADH	5	95	0	0

Table S104: Substrate 26a in methylammonium formate buffer.

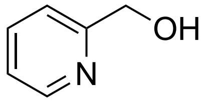

26a

26a MeNH2	Ald	Alc	Acid	Amide
Pf-ADH	0	100	0	0
Pp-ADH	2	95	0	3
AA-ADH	4	61	0	35
Te-ADH	0	100	0	0
Ht-ADH	77	23	0	0

Table S105: Substrate 27a in methylammonium formate buffer.

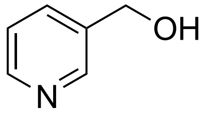

27a

27a MeNH2	Ald	Alc	Acid	Amide
Pf-ADH	4	94	0	2
Pp-ADH	4	93	0	3
AA-ADH	11	70	0	19
Te-ADH	0	100	0	0
Ht-ADH	92	8	0	0

Table S106: Substrate 28a in methylammonium formate buffer.

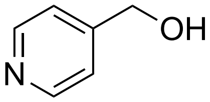

28a

28a MeNH2	Ald	Alc	Acid	Amide
Pf-ADH	0	97	0	3
Pp-ADH	0	100	0	0
AA-ADH	20	76	0	20
Te-ADH	0	100	0	0
Ht-ADH	54	46	0	0

$EtNH_2$

Table S107: Substrate 1a in ethylammonium formate buffer.

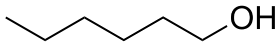

1a

1a EtNH2	Ald	Alc	Acid	Amide
Aa-ADH^{Y93A}	1	98	0	1
Aa-ADH^{Y151A}	5	58	1	37
AA-ADH^{L186A}	0	99	0	1

n-$PrNH_2$

Table S108: Substrate 1a in n-propylammonium formate buffer.

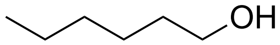

1a

1a nPrNH2	Ald	Alc	Acid	Amide
Aa-ADH^{Y93A}	1	98	0	1
Aa-ADH^{Y151A}	8	89	0	3
AA-ADH^{L186A}	1	98	0	1

iPr-NH_2

Table S109: Substrate 1a in isopropyl ammonium formate buffer.

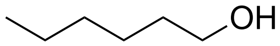

1a

1a iPrNH2	Ald	Alc	Acid	Amide
Aa-ADH^{Y93A}	1	98	0	1
Aa-ADH^{Y151A}	5	58	1	37
AA-ADH^{L186A}	0	99	0	1

12.2. Thioacids and thioesters

Table S110: Substrate 1a in hydrogen sulfide formate buffer.

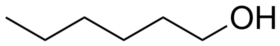

1a

1a H_2S	Ald	Alc	Acid	Thioacid
Pf-ADH	0	0	7	93
Pp-ADH	1	1	5	93
AA-ADH	3	3	3	91

Table S111: Substrate 3a in hydrogen sulfide formate buffer.

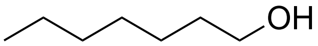

3a

3a H_2S	Ald	Alc	Acid	Thioacid
Pf-ADH	0	0	8	92
Pp-ADH	3	90	2	5
AA-ADH	2	5	4	89

Table S112: Substrate 4a in hydrogen sulfide formate buffer.

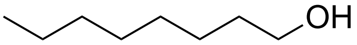

4a

4a H_2S	Ald	Alc	Acid	Thioacid
Pf-ADH	0	15	6	79
Pp-ADH	1	84	5	10
AA-ADH	3	17	2	78

Table S113: Substrate 5a in hydrogen sulfide formate buffer.

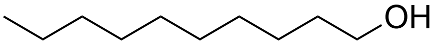

5a

5a H_2S	Ald	Alc	Acid	Thioacid
Pf-ADH	2	98	0	0
Pp-ADH	0	92	2	6
AA-ADH	1	76	2	21

Table S114: Substrate 6a in hydrogen sulfide formate buffer.

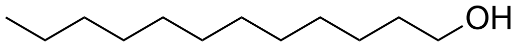

6a

6a H_2S	Ald	Alc	Acid	Thioacid
Pf-ADH	1	99	0	0
Pp-ADH	2	88	3	7
AA-ADH	2	77	1	20

Table S115: Substrate 7a in hydrogen sulfide formate buffer.

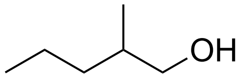

7a

7a H_2S	Ald	Alc	Acid	Thioacid
Pf-ADH	9	91	0	0
Pp-ADH	2	38	4	56
AA-ADH	0	0	0	100

Table S116: Substrate 8a in hydrogen sulfide formate buffer.

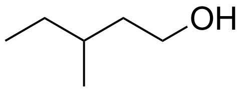

8a

8a H_2S	Ald	Alc	Acid	Thioacid
Pf-ADH	4	96	0	0
Pp-ADH	23	3	0	74
AA-ADH	16	46	1	37

Table S117: Substrate 9a in hydrogen sulfide formate buffer.

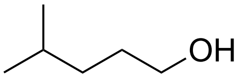

9a

9a H_2S	Ald	Alc	Acid	Thioacid
Pf-ADH	0	4	4	92
Pp-ADH	5	10	3	82
AA-ADH	3	34	1	62

Table S118: Substrate 10a in hydrogen sulfide formate buffer.

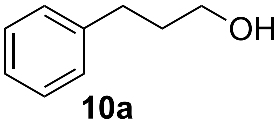

10a

10a H_2S	Ald	Alc	Acid	Thioacid
Pf-ADH	1	1	10	88
Pp-ADH	4	92	1	3
AA-ADH	2	90	0	8

Table S119: Substrate 11a in hydrogen sulfide formate buffer.

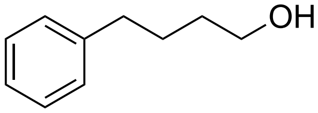

11a

11a H_2S	Ald	Alc	Acid	Thioacid
Pf-ADH	1	99	0	0
Pp-ADH	4	94	1	1
AA-ADH	2	90	1	7

Table S120: Substrate 13a in hydrogen sulfide formate buffer.

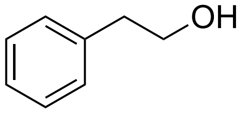

13a

13a H_2S	Ald	Alc	Acid	Thioacid
Pf-ADH	0	100	0	0
Pp-ADH	1	90	2	7
AA-ADH	1	98	0	1

Table S121: Substrate 14a in hydrogen sulfide formate buffer.

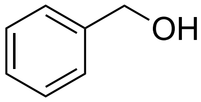

14a

14a H_2S	Ald	Alc	Acid	Thioacid
Pf-ADH	0	100	0	0
Pp-ADH	41	50	0	9
AA-ADH	0	16	1	83

Table S122: Substrate 15a in hydrogen sulfide formate buffer.

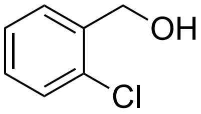

15a

15a H_2S	Ald	Alc	Acid	Thioacid
Pf-ADH	0	100	0	0
Pp-ADH	0	100	0	0
AA-ADH	64	10	0	24

Table S123: Substrate 16a in hydrogen sulfide formate buffer.

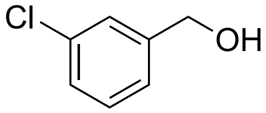

16a

16a H_2S	Ald	Alc	Acid	Thioacid
Pf-ADH	0	100	0	0
Pp-ADH	0	100	0	0
AA-ADH	25	4	1	70

Table S124: Substrate 17a in hydrogen sulfide formate buffer.

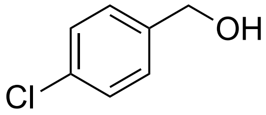

17a

17a H_2S	Ald	Alc	Acid	Thioacid
Pf-ADH	0	100	0	0
Pp-ADH	0	100	0	0
AA-ADH	74	10	0	16

Table S125: Substrate 18a in hydrogen sulfide formate buffer.

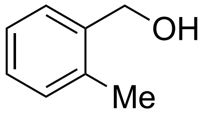

18a

18a H_2S	Ald	Alc	Acid	Thioacid
Pf-ADH	0	100	0	0
Pp-ADH	0	100	0	0
AA-ADH	0	8	8	84

Table S126: Substrate 19a in hydrogen sulfide formate buffer.

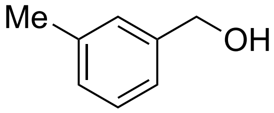

19a

19a H_2S	Ald	Alc	Acid	Thioacid
Pf-ADH	0	100	0	0
Pp-ADH	0	100	0	0
AA-ADH	13	2	3	82

Table S127: Substrate 20a in hydrogen sulfide formate buffer.

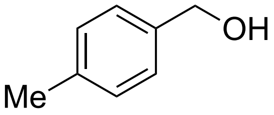

20a

20a H_2S	Ald	Alc	Acid	Thioacid
Pf-ADH	0	100	0	0
Pp-ADH	0	100	0	0
AA-ADH	0	3	4	93

Table S128: Substrate 21a in hydrogen sulfide formate buffer.

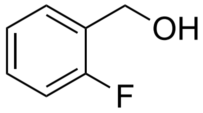

21a

21a H_2S	Ald	Alc	Acid	Thioacid
Pf-ADH	0	100	0	0
Pp-ADH	0	100	0	0
AA-ADH	0	36	0	64

Table S129: Substrate 22a in hydrogen sulfide formate buffer.

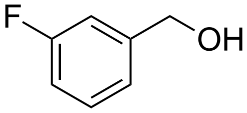

22a

22a H_2S	Ald	Alc	Acid	Thioacid
Pf-ADH	0	100	0	0
Pp-ADH	0	100	0	0
AA-ADH	0	40	3	57

Table S130: Substrate 23a in hydrogen sulfide formate buffer.

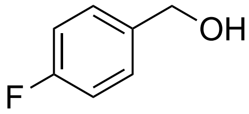

23a

23a H_2S	Ald	Alc	Acid	Thioacid
Pf-ADH	0	100	0	0
Pp-ADH	0	100	0	0
AA-ADH	3	53	4	40

Table S131: Substrate 24a in hydrogen sulfide formate buffer.

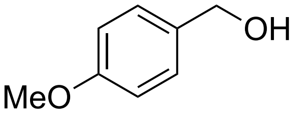

24a

24a H_2S	Ald	Alc	Acid	Thioacid
Pf-ADH	0	100	0	0
Pp-ADH	0	100	0	0
AA-ADH	0	0	10	90

MeSH

Table S132: Substrate 1a in methyl sulfide formate buffer.

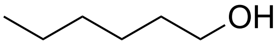

1a

1a MeSH	Ald	Alc	Acid	Methyl Thioester
Pf-ADH	6	43	7	44
Pp-ADH	0	5	0	95
AA-ADH	5	28	2	65

Table S133: Substrate 3a in methyl sulfide formate buffer.

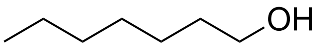

3a

3a MeSH	Ald	Alc	Acid	Methyl Thioester
Pf-ADH	16	49	5	30
Pp-ADH	4	46	3	47
AA-ADH	0	51	1	48

Table S134: Substrate 4a in methyl sulfide formate buffer.

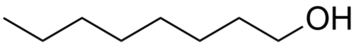

4a

4a MeSH	Ald	Alc	Acid	Methyl Thioester
Pf-ADH	3	63	5	29
Pp-ADH	0	4	1	95
AA-ADH	0	2	1	97

Table S135: Substrate 5a in methyl sulfide formate buffer.

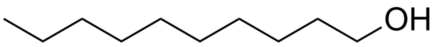

5a

5a MeSH	Ald	Alc	Acid	Methyl Thioester
Pf-ADH	2	98	0	0
Pp-ADH	8	55	3	34
AA-ADH		74	2	24

Table S136: Substrate 6a in methyl sulfide formate buffer.

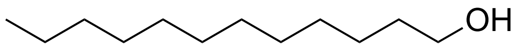

6a

6a MeSH	Ald	Alc	Acid	Methyl Thioester
Pf-ADH	1	99	0	0
Pp-ADH	5	95	0	0
AA-ADH	5	94	1	0

Table S137: Substrate 7a in methyl sulfide formate buffer.

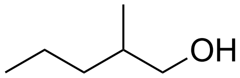

7a

7a MeSH	Ald	Alc	Acid	Methyl Thioester
Pf-ADH	0	100	0	0
Pp-ADH	2	98	0	0
AA-ADH		92	1	7

Table S138: Substrate 8a in methyl sulfide formate buffer.

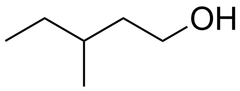

8a

8a MeSH	Ald	Alc	Acid	Methyl Thioester
Pf-ADH	2	83	3	12
Pp-ADH	0	72	0	28
AA-ADH	1	88	1	10

Table S139: Substrate 9a in methyl sulfide formate buffer.

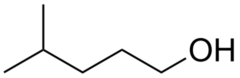

9a

9a MeSH	Ald	Alc	Acid	Methyl Thioester
Pf-ADH	0	32	0	68
Pp-ADH	1	47	2	50
AA-ADH	1	63	1	35

Table S140: Substrate 10a in methyl sulfide formate buffer.

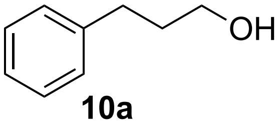

10a

10a MeSH	Ald	Alc	Acid	Methyl Thioester
Pf-ADH	52	12	10	26
Pp-ADH	54	32	4	10
AA-ADH	38	40	0	12

Table S141: Substrate 11a in methyl sulfide formate buffer.

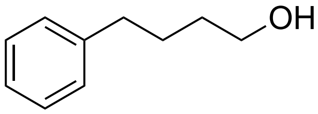

11a

11a MeSH	Ald	Alc	Acid	Methyl Thioester
Pf-ADH	1	93	1	5
Pp-ADH	2	80		18
AA-ADH	4	84	1	11

Table S142: Substrate 13a in methyl sulfide formate buffer.

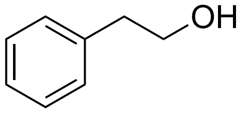

13a

13a MeSH	Ald	Alc	Acid	Methyl Thioester
Pf-ADH	0	100	0	0
Pp-ADH	2	93	0	5
AA-ADH	1	98	0	1

Table S143: Substrate 14a in methyl sulfide formate buffer.

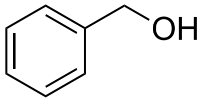

14a

14a MeSH	Ald	Alc	Acid	Methyl Thioester
Pf-ADH	0	100	0	0
Pp-ADH	1	85	0	14
AA-ADH	0	11	6	83

Table S144: Substrate 15a in methyl sulfide formate buffer.

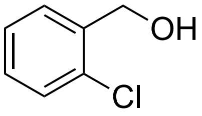

15a

15a MeSH	Ald	Alc	Acid	Methyl Thioester
Pf-ADH	0	100	0	0
Pp-ADH	0	100	0	0
AA-ADH	2	57	0	41

Table S145: Substrate 16a in methyl sulfide formate buffer.

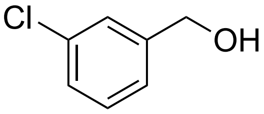

16a

16a MeSH	Ald	Alc	Acid	Methyl Thioester
Pf-ADH	0	100	0	0
Pp-ADH	0	100	0	0
AA-ADH	25	5		70

Table S146: Substrate 17a in methyl sulfide formate buffer.

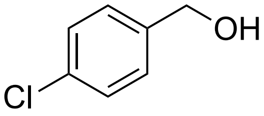

17a

17a MeSH	Ald	Alc	Acid	Methyl Thioester
Pf-ADH	0	100	0	0
Pp-ADH	0	100	0	0
AA-ADH	0	58	0	42

Table S147: Substrate 18a in methyl sulfide formate buffer.

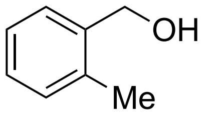

18a

18a MeSH	Ald	Alc	Acid	Methyl Thioester
Pf-ADH	0	100	0	0
Pp-ADH	0	100	0	0
AA-ADH	0	7	3	90

Table S148: Substrate 19a in methyl sulfide formate buffer.

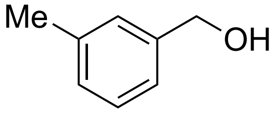

19a

19a MeSH	Ald	Alc	Acid	Methyl Thioester
Pf-ADH	0	100	0	0
Pp-ADH	0	100	0	0
AA-ADH	0	56	0	44

Table S149: Substrate 20a in methyl sulfide formate buffer.

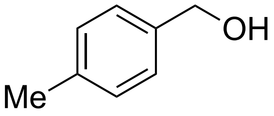

20a

20a MeSH	Ald	Alc	Acid	Methyl Thioester
Pf-ADH	0	100	0	0
Pp-ADH	0	100	0	0
AA-ADH	0	1	1	98

Table S150: Substrate 21a in methyl sulfide formate buffer.

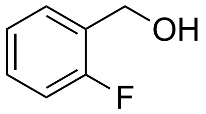

21a

21a MeSH	Ald	Alc	Acid	Methyl Thioester
Pf-ADH	0	100	0	0
Pp-ADH	0	100	0	0
AA-ADH	0	23	3	64

Table S151: Substrate 22a in methyl sulfide formate buffer..

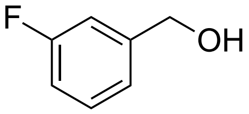

22a

22a MeSH	Ald	Alc	Acid	Methyl Thioester
Pf-ADH	0	100	0	0
Pp-ADH	0	100	0	0
AA-ADH	0	53	0	47

Table S152: Substrate 23a in methyl sulfide formate buffer..

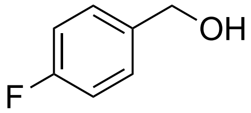

23a

23a MeSH	Ald	Alc	Acid	Methyl Thioester
Pf-ADH	0	100	0	0
Pp-ADH	0	100	0	0
AA-ADH	0	53	1	46

Table S153: Substrate 24a in methyl sulfide formate buffer..

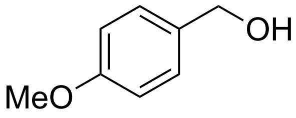

24a

24a MeSH	Ald	Alc	Acid	Methyl Thioester
Pf-ADH	0	100	0	0
Pp-ADH	0	100	0	0
AA-ADH	0	10	0	90

EtSH

Table S154: Substrate 1a in biphasic system (KPi 500 mM pH 7 and MTBE containing 0.5 M EtSH).

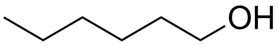

1a

1a EtSH	Ald	Alc	Acid	Thioester
Pf-ADH	2	96	1	1
Pp-ADH	0	43	4	53
AA-ADH	0	78	1	21

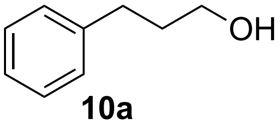

10a

Table S155: Substrate 10a in biphasic system (KPi 500 mM pH 7 and MTBE containing 0.5 M EtSH).

10a EtSH	Ald	Alc	Acid	Thioester
Pf-ADH	0	100	0	0
Pp-ADH	8	55	0	37
AA-ADH	8	76	1	15

Table S156: Substrate 14a in biphasic system (KPi 500 mM pH 7 and MTBE containing 0.5 M EtSH).

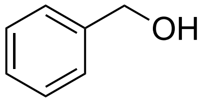

14a

14a EtSH	Ald	Alc	Acid	Thioester
Pf-ADH	0	100	0	0
Pp-ADH	35	45	3	17
AA-ADH	33	51	1	15

n-PrSH

Table S157: Substrate 1a in biphasic system (KPi 500 mM pH 7 and MTBE containing 0.5 M n-PrSH).

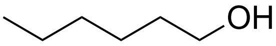

1a

1a n-PrSH	Ald	Alc	Acid	Thioester
Aa-ADH^{Y93A}	1	98	0	1
Aa-ADH^{Y151A}	0	59	0	41
AA-ADH^{L186A}	1	90	0	9

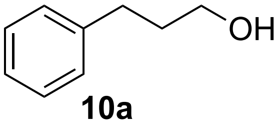

10a

Table S158: Substrate 10a in biphasic system (KPi 500 mM pH 7 and MTBE containing 0.5 M n-PrSH).

10a n-PrSH	Ald	Alc	Acid	Thioester
Aa-ADH^{Y93A}	0	100	0	0
Aa-ADH^{Y151A}	0	100	0	0
AA-ADH^{L186A}	16	71	0	13

Table S159: Substrate 14a in biphasic system (KPi 500 mM pH 7 and MTBE containing 0.5 M n-PrSH).

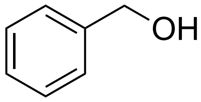

14a

14a n-PrSH	Ald	Alc	Acid	Thioester
Aa-ADH^{Y93A}	0	100	0	0
Aa-ADH^{Y151A}	39	12	3	46
AA-ADH^{L186A}	40	14	2	44

i-PrSH

Table S160: Substrate 1a in biphasic system (KPi 500 mM pH 7 and MTBE containing 0.5 M i-PrSH).

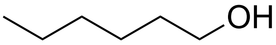

1a

1a i-PrSH	Ald	Alc	Acid	Thioester
Aa-ADH^{Y93A}	40	58	1	1
Aa-ADH^{Y151A}	37	56	0	7
AA-ADH^{L186A}	1	90	0	1

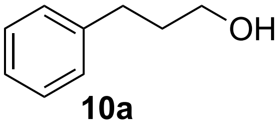

10a

Table S161: Substrate 10a in biphasic system (KPi 500 mM pH 7 and MTBE containing 0.5 M i-PrSH).

10a i-PrSH	Ald	Alc	Acid	Thioester
Aa-ADH^{Y93A}	0	100	0	0
Aa-ADH^{Y151A}	0	100	0	0
AA-ADH^{L186A}	1	98	0	1

Table S162: Substrate 14a in biphasic system (KPi 500 mM pH 7 and MTBE containing 0.5 M i-PrSH).

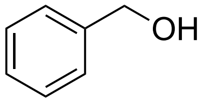

14a

14a i-PrSH	Ald	Alc	Acid	Thioester
Aa-ADH^{Y93A}	0	100	0	0
Aa-ADH^{Y151A}	81	7	0	12
AA-ADH^{L186A}	88	1	0	11

n-BuSH

Table S163: Substrate 1a in biphasic system (KPi 500 mM pH 7 and MTBE containing 0.5 M BuSH).

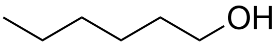

1a

1a n-BuSH	Ald	Alc	Acid	Thioester
Aa-ADH^{Y93A}	1	98	0	1
Aa-ADH^{Y151A}	0	59	0	7
AA-ADH^{L186A}	1	90	0	5

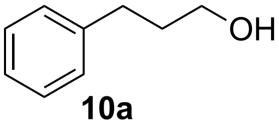

10a

Table S164: Substrate 10a in biphasic system (KPi 500 mM pH 7 and MTBE containing 0.5 M BuSH).

10a n-BuSH	Ald	Alc	Acid	Thioester
Aa-ADH^{Y93A}	0	100	0	0
Aa-ADH^{Y151A}	0	100	0	0
AA-ADH^{L186A}	0	100	0	0

Table S165: Substrate 14a in biphasic system (KPi 500 mM pH 7 and MTBE containing 0.5 M BuSH).

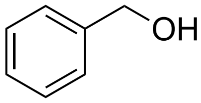

14a

14a n-BuSH	Ald	Alc	Acid	Thioester
Aa-ADH^{Y93A}	0	100	0	0
Aa-ADH^{Y151A}	27	3	2	68
AA-ADH^{L186A}	40	47	1	12

13. Computational studies

13.1. General procedure of docking

The molecular dockings were performed using Autodock Vina as tool incorporated into YASARA structure using the crystal structure of Aa-ADH in complex with NAD (PDB: 2EWB). In instances where variants of Aa-ADH were used, all mutations to the parental enzyme were simulated in silico using the YASARA structure software with the AMBER 03 force field.[9] After the introduction of a mutation or modification, the energy of the system was minimized following a three-step protocol deformation. In step one, only the atoms constituting the mutated amino acid residue were subjected to energy minimization. In step two, the process for energy minimization was repeated by including all the atoms of the amino acid residues that are located within 6 Å distance from the mutated residue. In step three, the energy of the overall structure was minimized.
The 3D structures of the substrates (**1d'**, **1i'**, and **1j'**) were built using YASARA structures, saved as pdb files, and subjected to energy minimization using the AMBER03 force field before docking.
In all dockings (**Table S166**), the simulation box was placed 10 Å around the active site of the enzyme. For each simulation, 25 VINA docking runs of each ligand to the receptor were run. After clustering the 25 runs, the following 3 distinct complex conformations were found for each docking simulation. Results were sorted by binding energy and in all cases the binding pose with the best binding energy was selected. More positive energies indicate stronger binding and negative energies mean no binding. All binding poses were inspected visually.

Table S166: Docking studies performed in this work

Entry	Enzyme	Substrate	Docking clusters	*Binding energy (Kcal/mol)*
1	Aa-ADH	**1d'**	**001**	**000004.4100**
			002	000004.0430
			003	000003.0970
2	AA-ADH^{Y151A}	**1i'**	**001**	**000004.8070**
			002	000004.1080
			003	000003.5880
3	AA-ADH^{Y151A}	**1j'**	**001**	**000004.4870**
			002	000004.0060
			003	000003.8720

13.2. Docking experiment to rationalize the lack of reactivity

In a separate molecular docking simulation, we examined the binding of intermediate **14d'**, formed from **14a** and ethylamine (Figure S6a), within the Aa-ADH^{Y151A} variant. Although this reaction yielded only 2% conversion, it provides valuable insight into the structural basis of the limited reactivity observed. The docking results suggest that while the aromatic ring of **14a** is still accommodated within the enlarged cavity created by the Y151A mutation, the intermediate adopts a non-productive orientation. In particular, the distance between the hydrogen at the oxidation site of the hemiaminal and the C4 carbon of the NAD^+ nicotinamide ring (dashed blue line in Figure S6c-d) was found to be 5.6 Å, which is too long for efficient hydride transfer. Notably, this pose corresponded to the most representative one from the only cluster identified in this docking run, with a calculated binding energy of 4.23 kcal/mol, indicating stable yet catalytically unproductive binding.

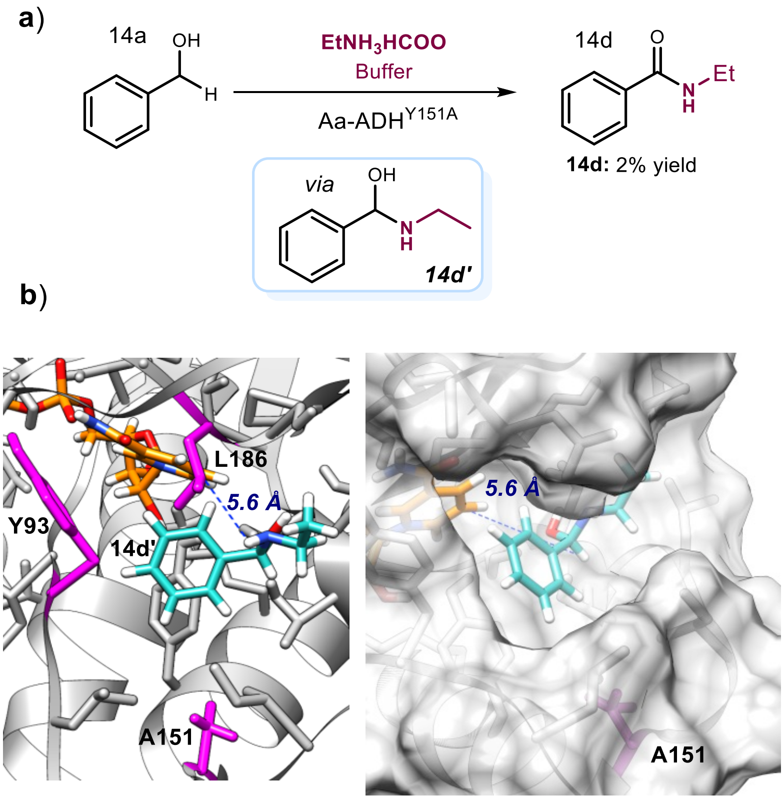

Figure S6: a) Reaction investigated with docking simulations using 14d' as ligand. b-c) Docking outcome of the best binding energy.

14. Site-directed mutagenesis procedure

The AA-ADH variants were obtained by site-directed mutagenesis using the Q5 site-directed mutagenesis kit (NEB), according to following procedure. Mutagenic primers (**Table S167**, 0.5 µM) were used in PCR reactions (25 µL) using 20 ng of the template DNA, and Q5 Hot Start High-Fidelity 2X Master Mix provided by the kit. PCR parameters were: 1 cycle for 30 seconds at 98 ˚C, following by 25 cycles, each one consisted of 10 seconds at 98 °C (denaturation), 30 seconds at the appropriate Ta (**Table S167**, annealing) and 3 minutes at 72˚C (extension). After the PCR the reactions were treated with Kinase, Ligase and DpnI (KLD) enzyme mix for circularization and template removal. Next, 5 µL of the purified product were used to transform competent BL21 (DE3) *E.coli* cells. In total, 100 µL of the transformation were plated intro LB-agar petri dishes containing kanamycin (50 µg mL^{-1}) and the next day single colonies were picked. These colonies were used to inoculate LB medium overnight. The resulted overnight cultures were used for plasmid extraction and for glycerol stock preparation. The mutations introduced were verified with sequencing and the glycerol stocks were used for enzyme expression following the procedure reported in section 6.

Table S167: Primers used for construction of AA-ADH variants.

Parental DNA	Mutations		Primer Sequence (5'→ 3')	Ta
AA-ADH	Y93A	F	CGCGGGCATCgcgCCGCTGATTC	57
		R	TTGTTAACCAGGATGTC	
	Y151A	F	GATCGAAGCGgcgACCCACTATATTAGC	58
		R	TTCAGCCAATAGGTGG	
	L186A	F	TGCGCCGAGCgcgGTTCGTACCG	66
		R	ATCGCGTTCACGGTGATG	

15. Analytical methods and GC chromatograms

Method A: constant pressure 6.9 psi, split ratio 20:1, T injector 250 °C. Temperature program: T initial 60 °C, hold 6.5 min, gradient 20 °C/min up to 100 °C; hold 1 min, gradient 20 °C/min up to 280 °C; hold 1 min.

Method B: constant pressure 6.9 psi, split ratio 20:1, T injector 250 °C. Temperature program: T initial 80 °C, hold 6.5 min, gradient 10 °C/min up to 160 °C; hold 1 min, gradient 20 °C/min up to 200 °C; hold 2 min, gradient 20 °C/min up to 280 °C; hold 1 min.

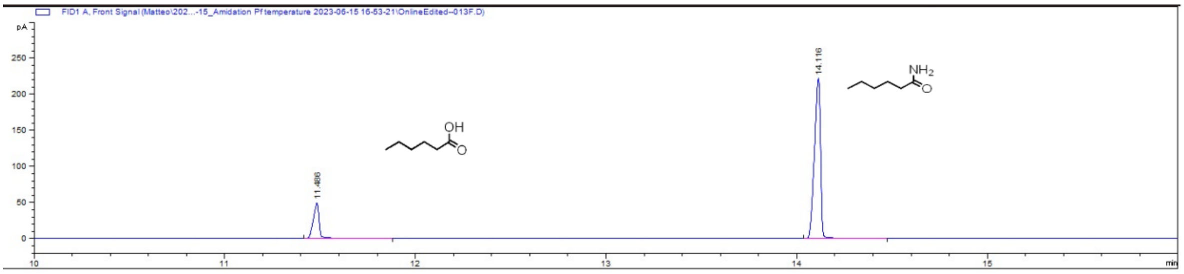

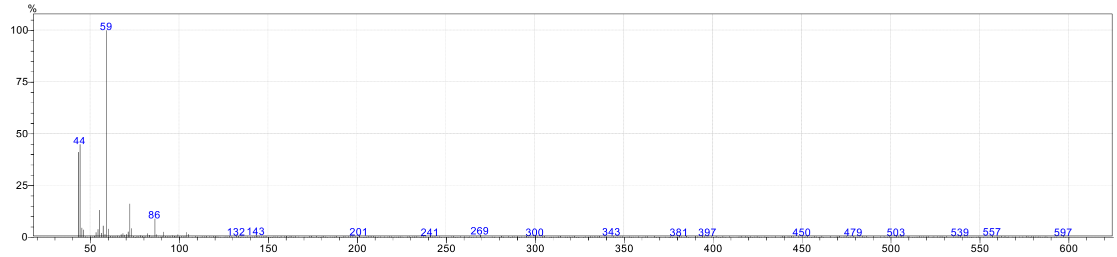

Figure S7: Up) GC-FID chromatogram for the determination of the conversion of 1a with Pf-ADH. Bottom) GC-MS of 1d.

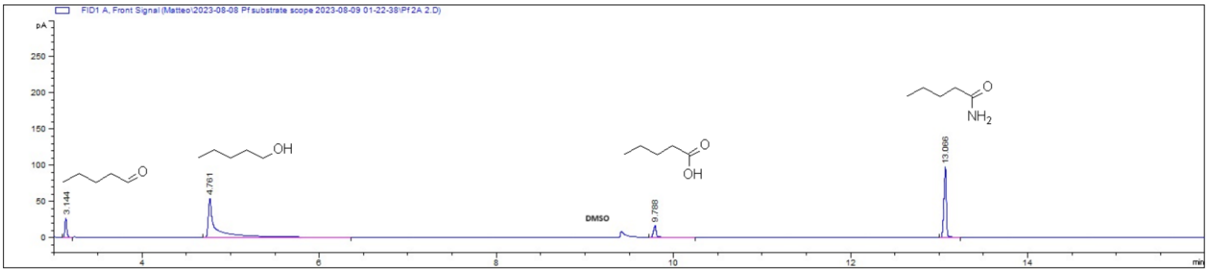

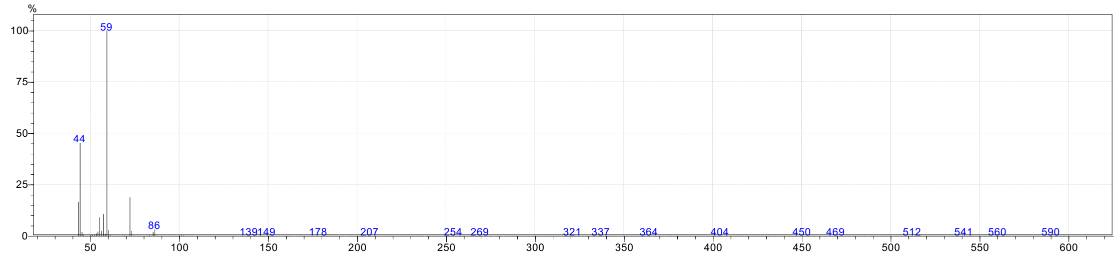

Figure S8: Up) GC-FID chromatogram for the determination of the conversion of 2a with Pf-ADH. Bottom) GC-MS of 2d.

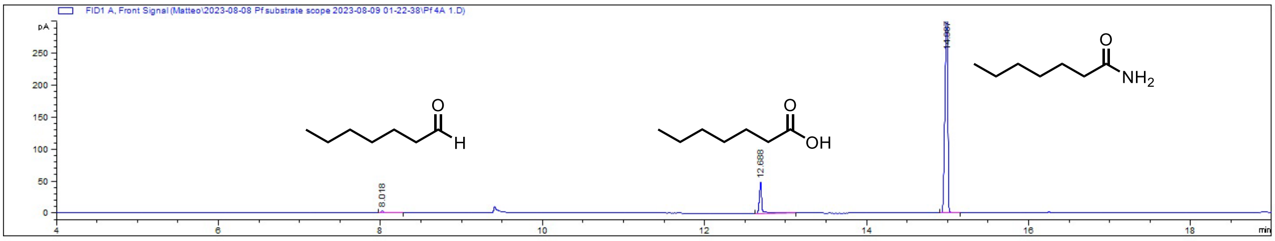

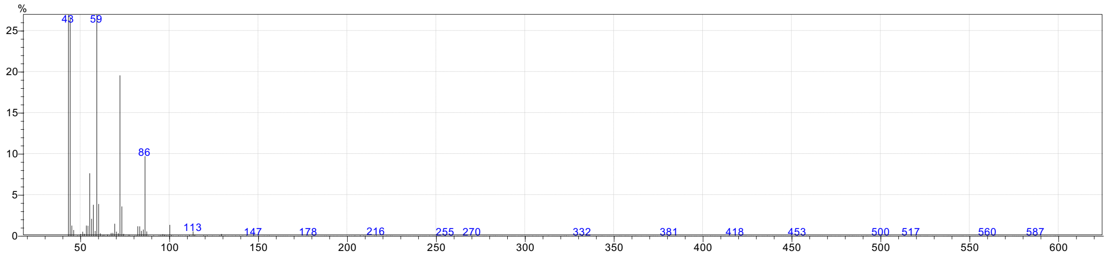

Figure S9: Up) GC-FID chromatogram for the determination of the conversion of 3a with Pf-ADH. Bottom) GC-MS of 3d.

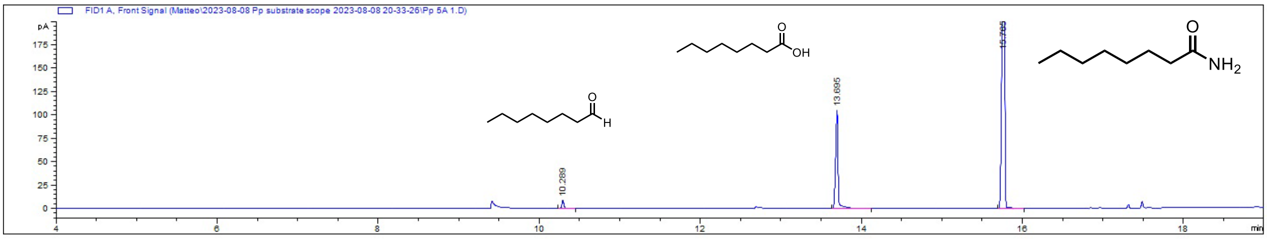

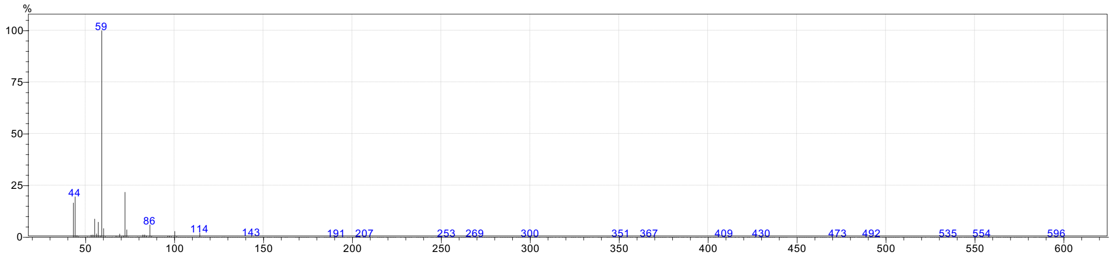

Figure S10: Up) GC-FID chromatogram for the determination of the conversion of 4a with Pp-ADH. Bottom) GC-MS of 4d.

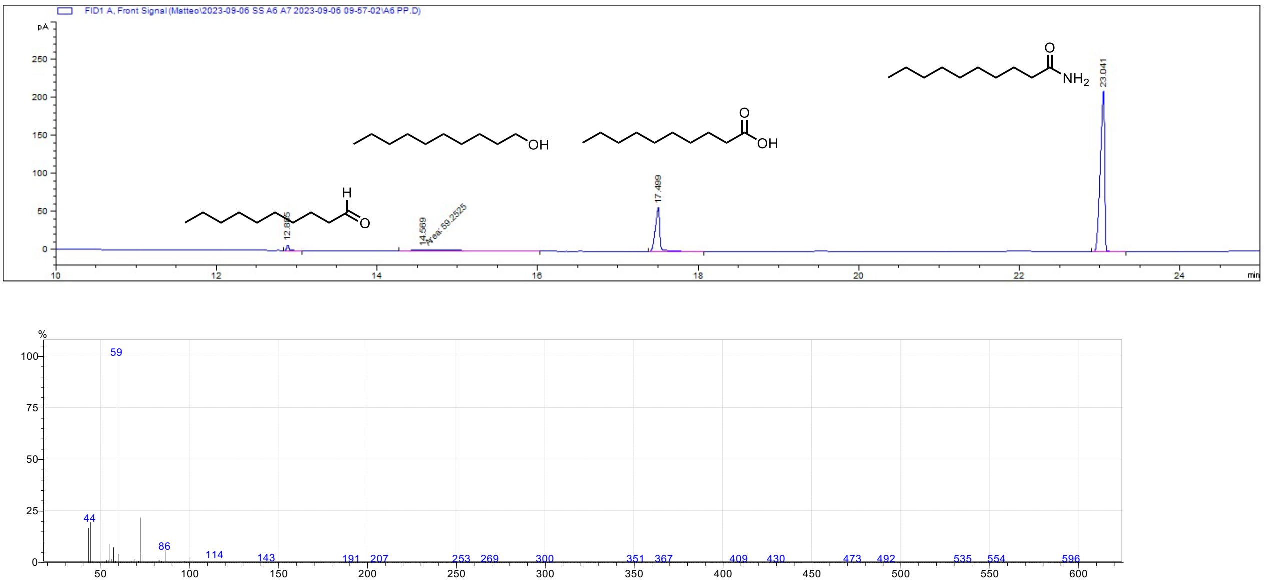

Figure S11: Up) GC-FID chromatogram for the determination of the conversion of 5a with Pp-ADH. Bottom) GC-MS of 5d.

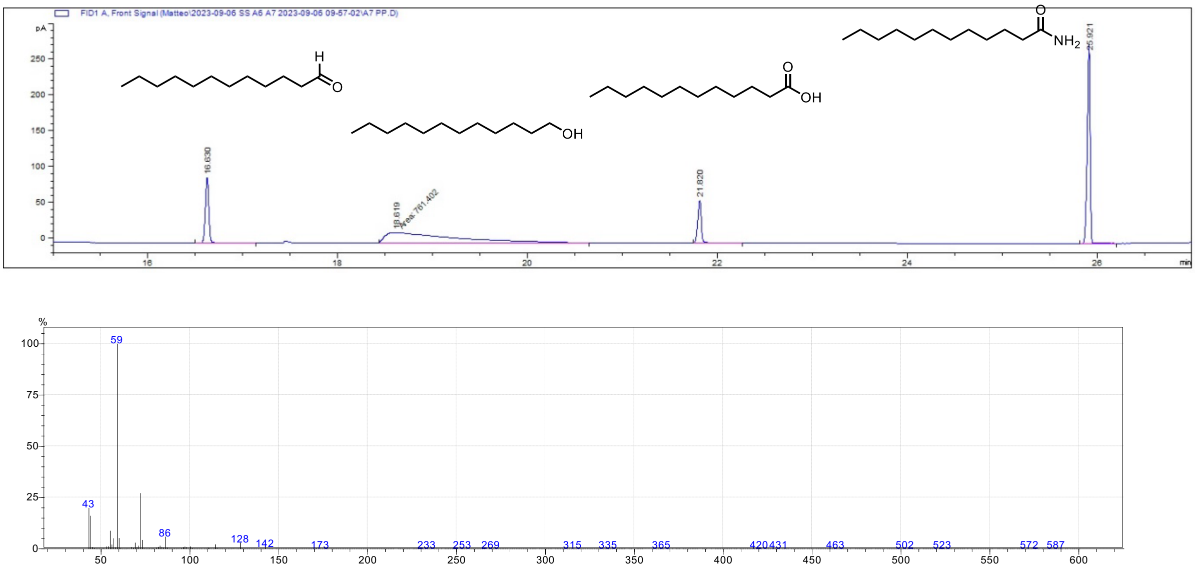

Figure S12: Up) GC-FID chromatogram for the determination of the conversion of 6a with Aa-ADH. Bottom) GC-MS of 6d.

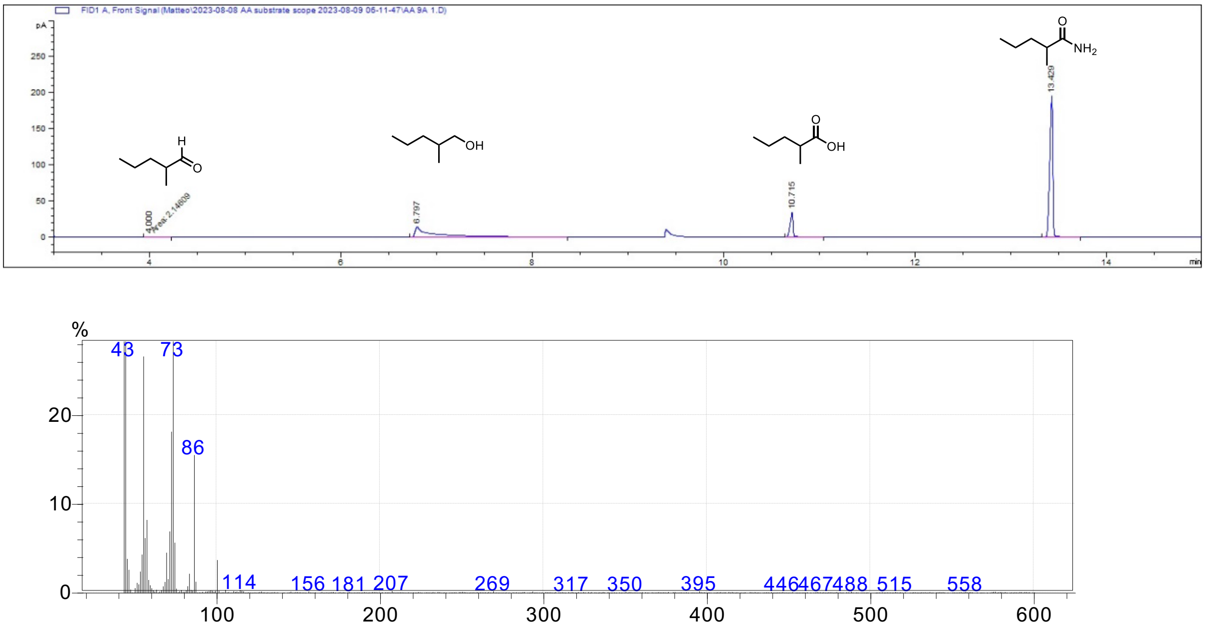

Figure S13: Up) GC-FID chromatogram for the determination of the conversion of 7a with Aa-ADH. Bottom) GC-MS of 7d.

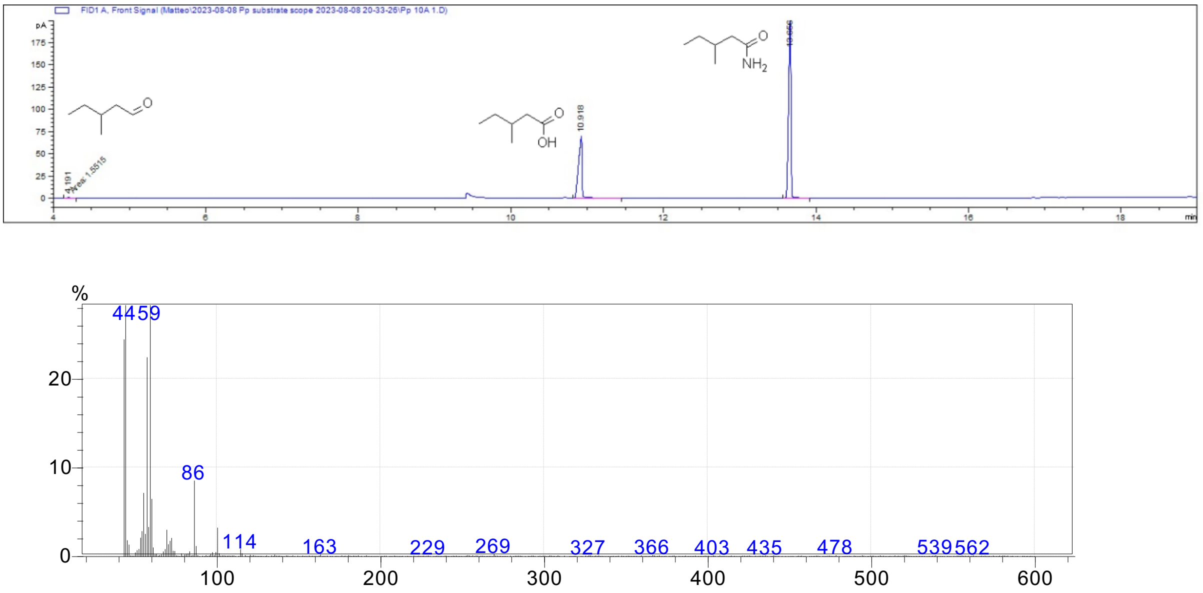

Figure S14: Up) GC-FID chromatogram for the determination of the conversion of 8a with Pp-ADH. Bottom) GC-MS of 8d.

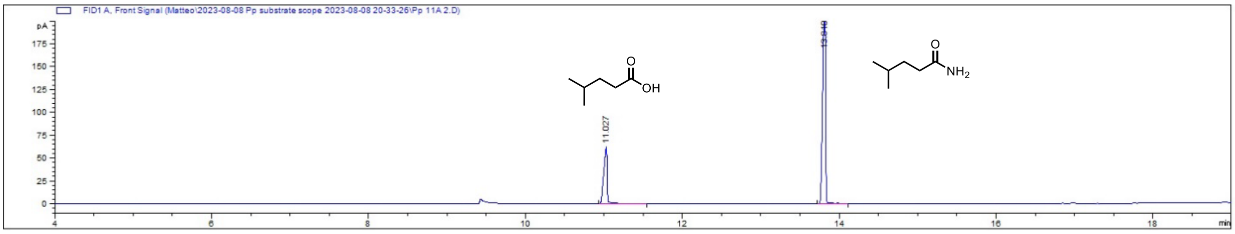

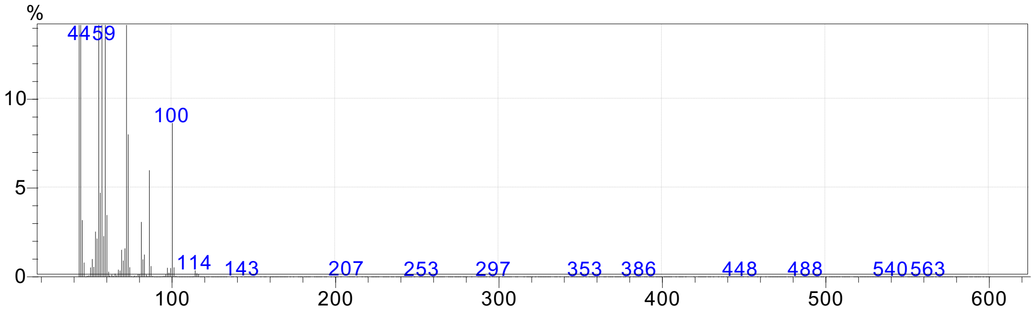

Figure S15: Up) GC-FID chromatogram for the determination of the conversion of 9a with Pp-ADH. Bottom) GC-MS of 9d.

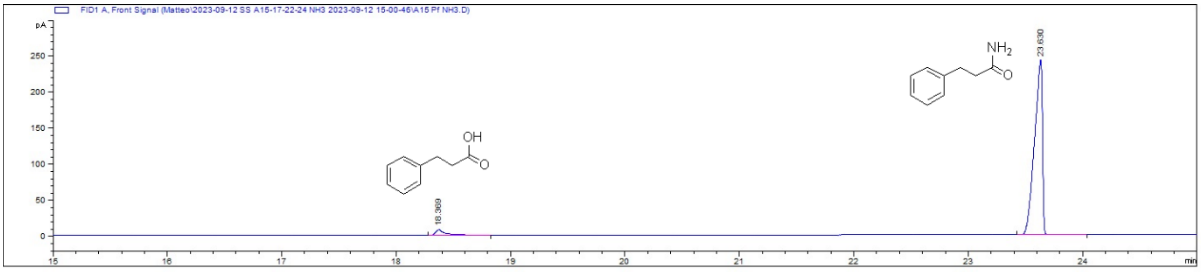

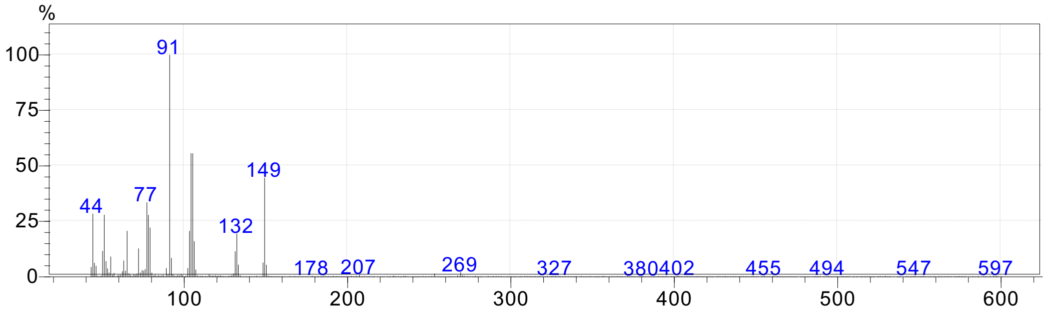

Figure S16: Up) GC-FID chromatogram for the determination of the conversion of 10a with Pf-ADH. Bottom) GC-MS of 10d.

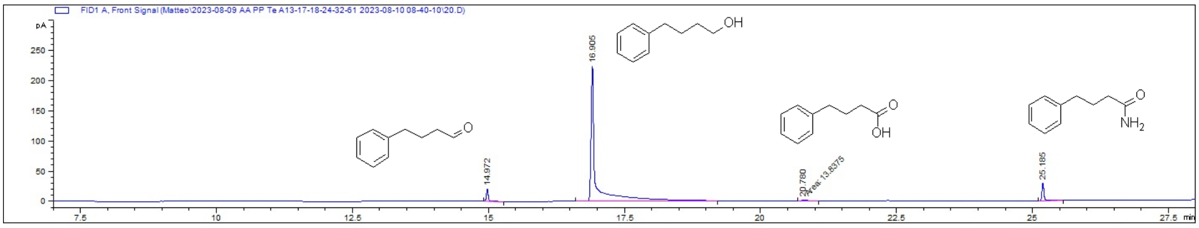

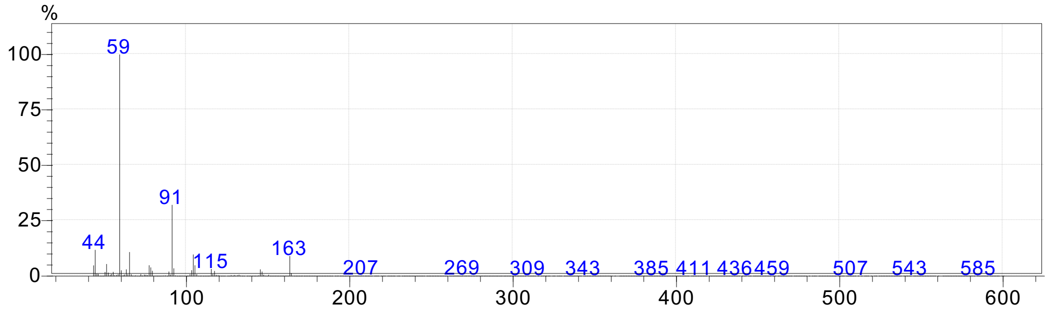

Figure S17: Up) GC-FID chromatogram for the determination of the conversion of 11a with Pp-ADH. Bottom) GC-MS of 11d.

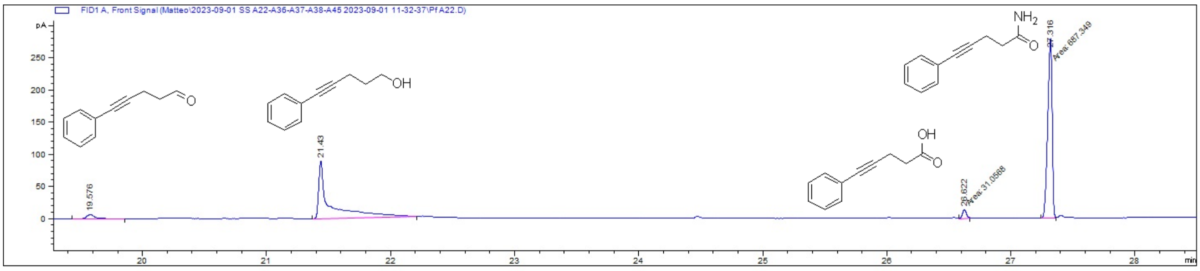

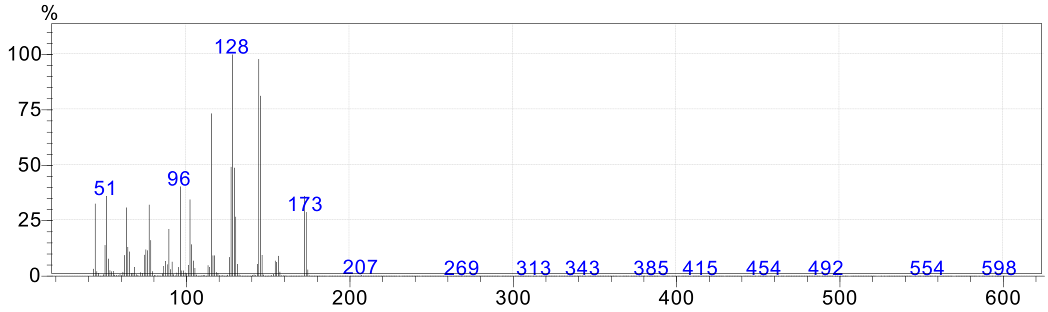

Figure S18: Up) GC-FID chromatogram for the determination of the conversion of 12a with Pf-ADH. Bottom) GC-MS of 12d.

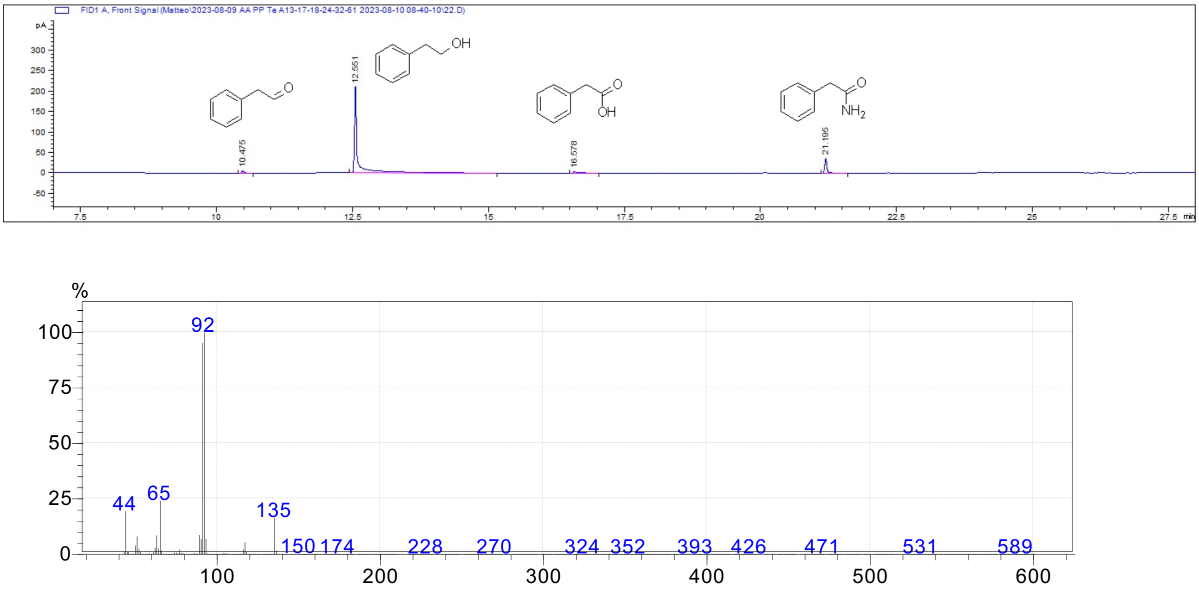

Figure S19: Up) GC-FID chromatogram for the determination of the conversion of 13a with Pp-ADH. Bottom) GC-MS of 13d.

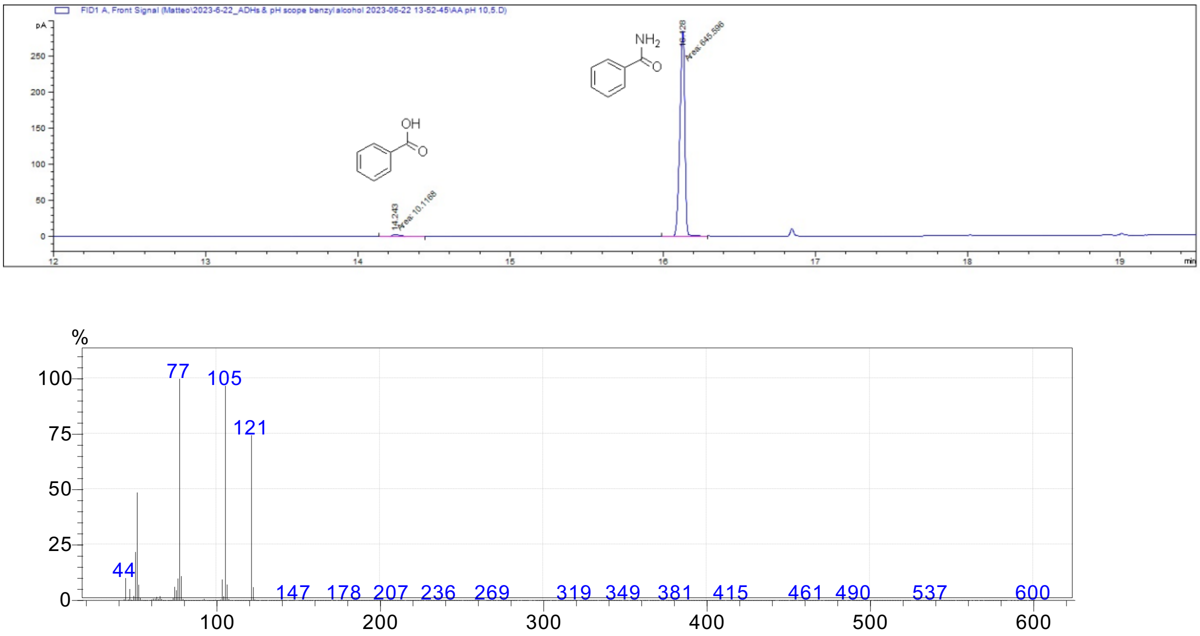

Figure S20: Up) GC-FID chromatogram for the determination of the conversion of 14a with Aa-ADH. Bottom) GC-MS of 14d.

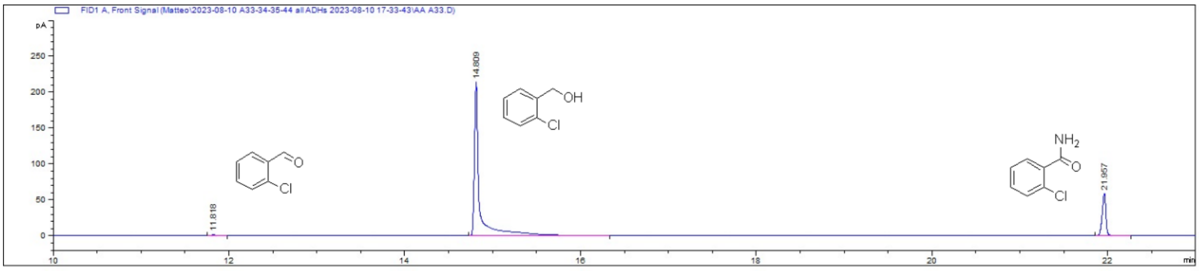

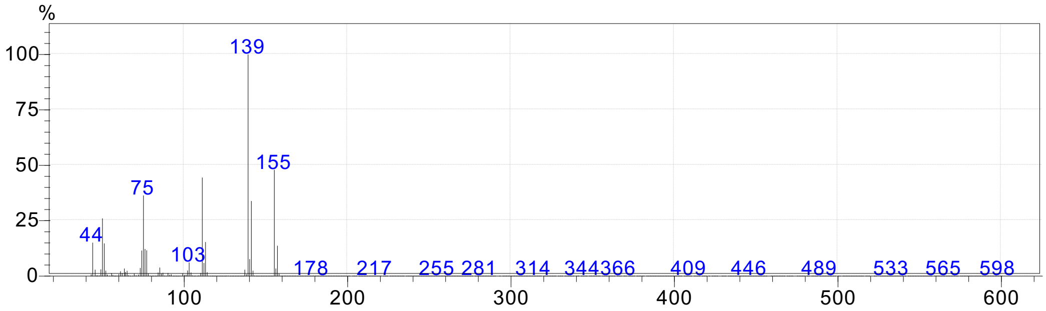

Figure S21: Up) GC-FID chromatogram for the determination of the conversion of 15a with Aa-ADH. Bottom) GC-MS of 15d.

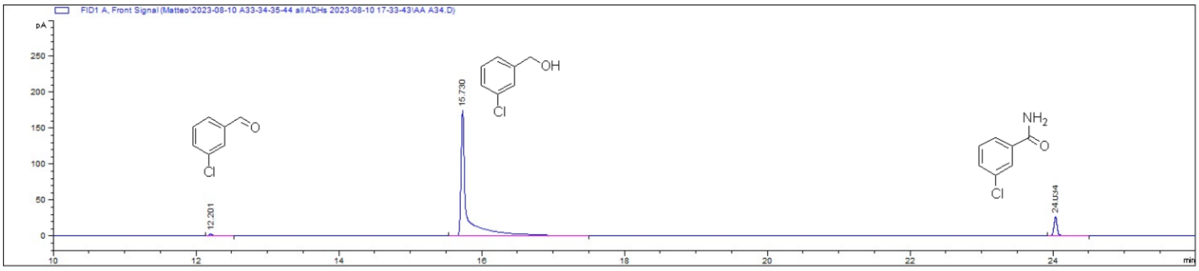

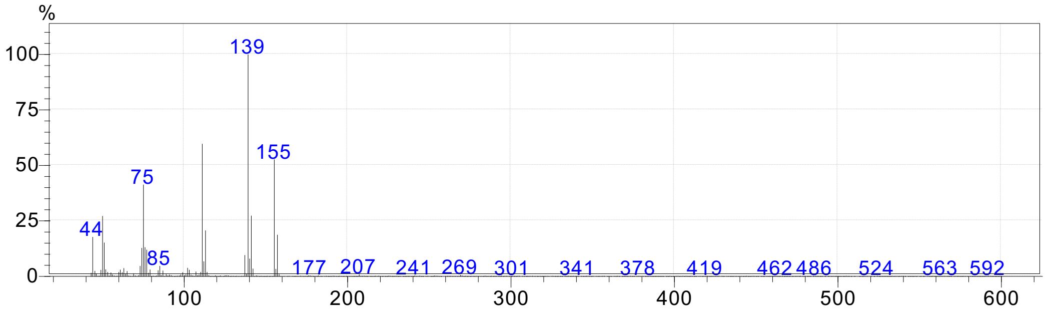

Figure S22: Up) GC-FID chromatogram for the determination of the conversion of 16a with Aa-ADH. Bottom) GC-MS of 16d.

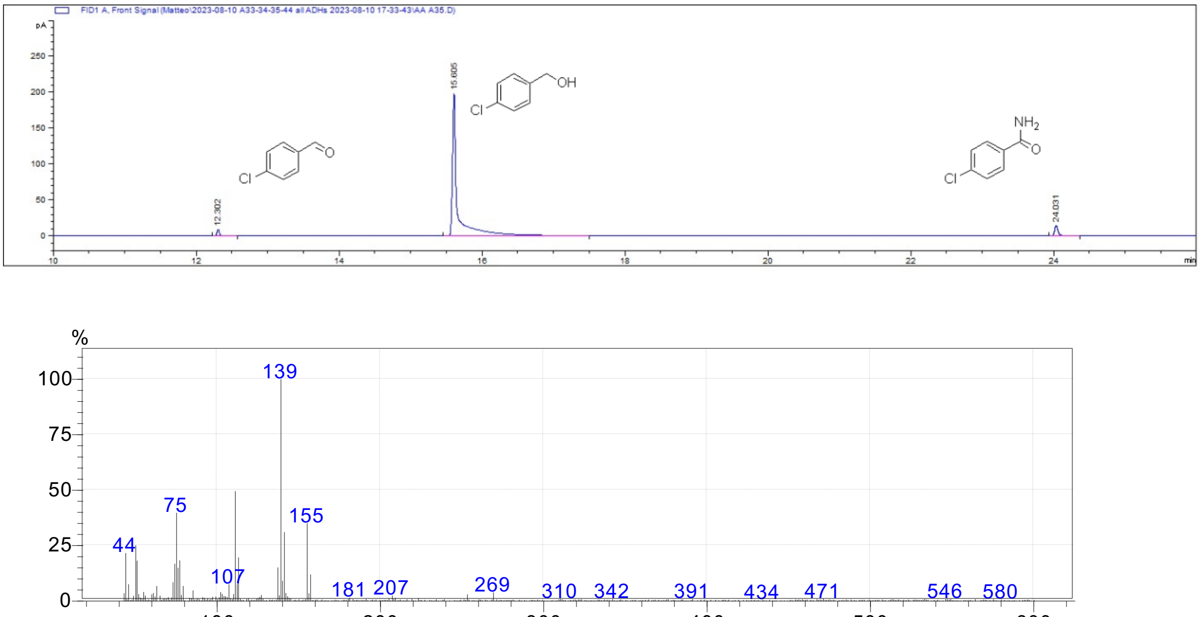

Figure S23: Up) GC-FID chromatogram for the determination of the conversion of 17a with Aa-ADH. Bottom) GC-MS of 17d.

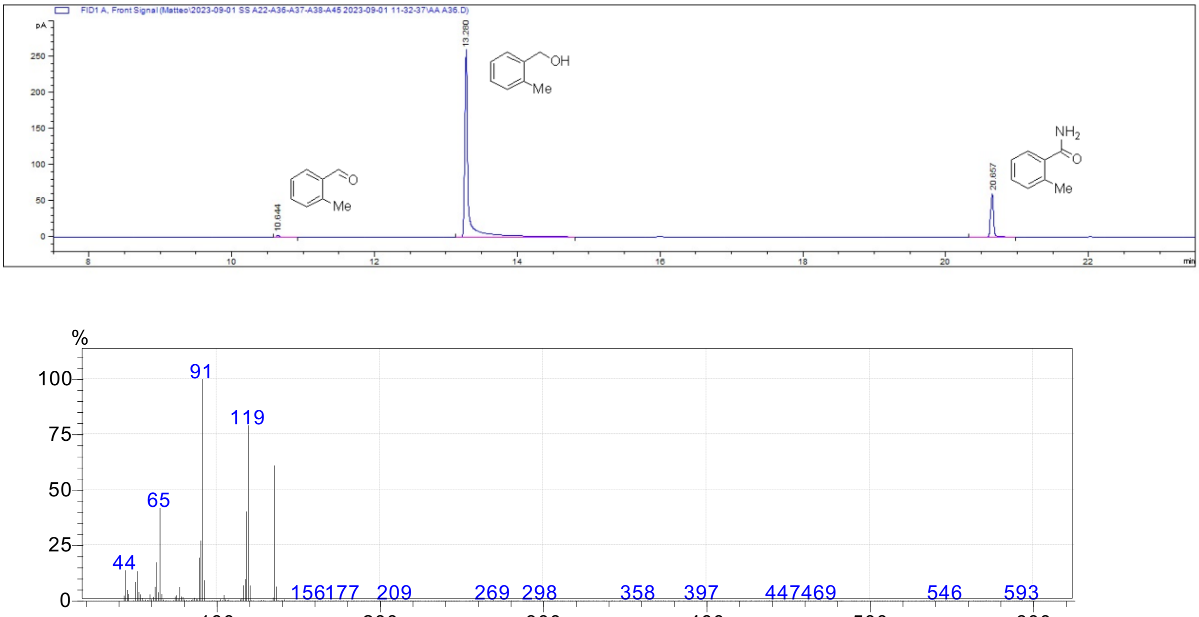

Figure S24: Up) GC-FID chromatogram for the determination of the conversion of 18a with Aa-ADH. Bottom) GC-MS of 18d.

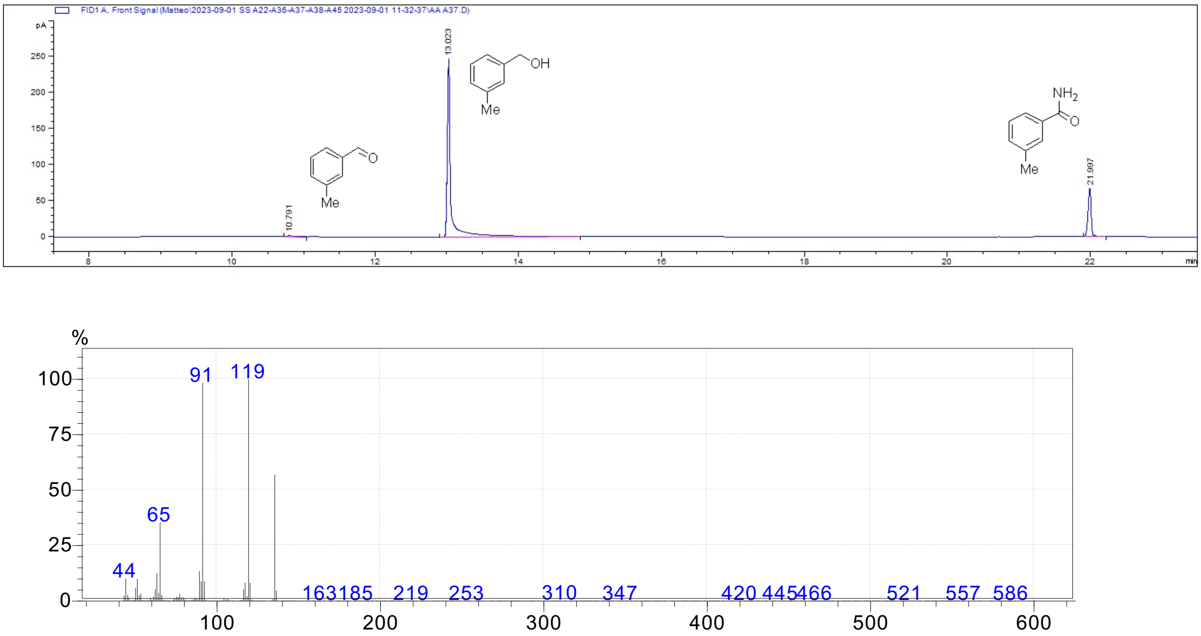

Figure S25: Up) GC-FID chromatogram for the determination of the conversion of 19a with Aa-ADH. Bottom) GC-MS of 19d.

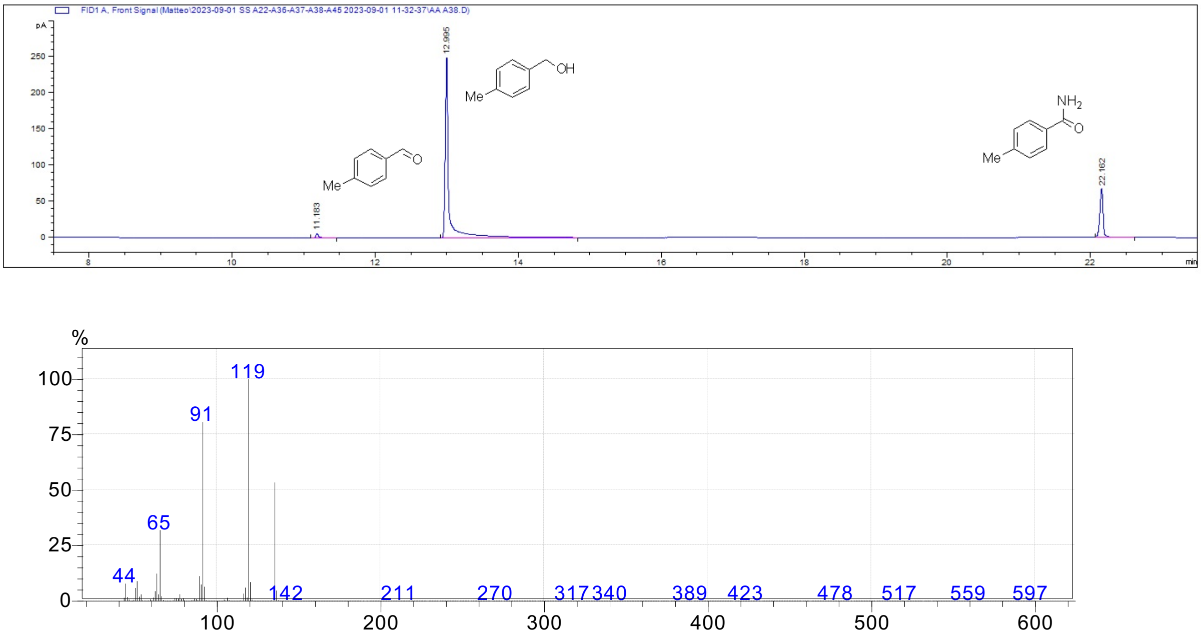

Figure S26: Up) GC-FID chromatogram for the determination of the conversion of 20a with Aa-ADH. Bottom) GC-MS of 20d.

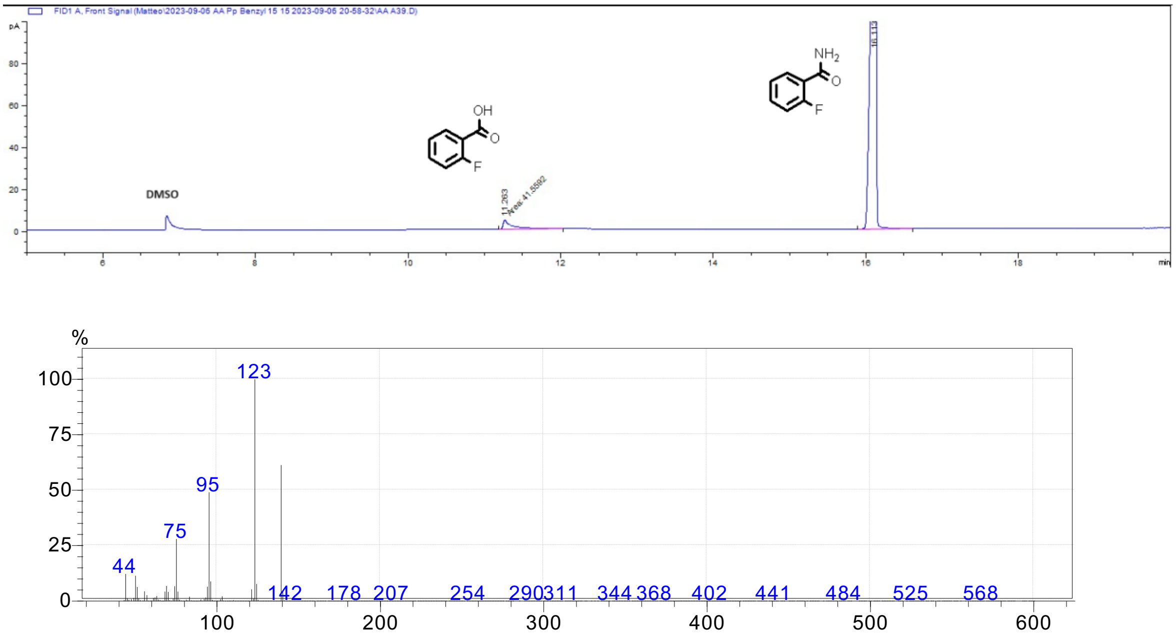

Figure S27: Up) GC-FID chromatogram for the determination of the conversion of 21a with Aa-ADH. Bottom) GC-MS of 21d.

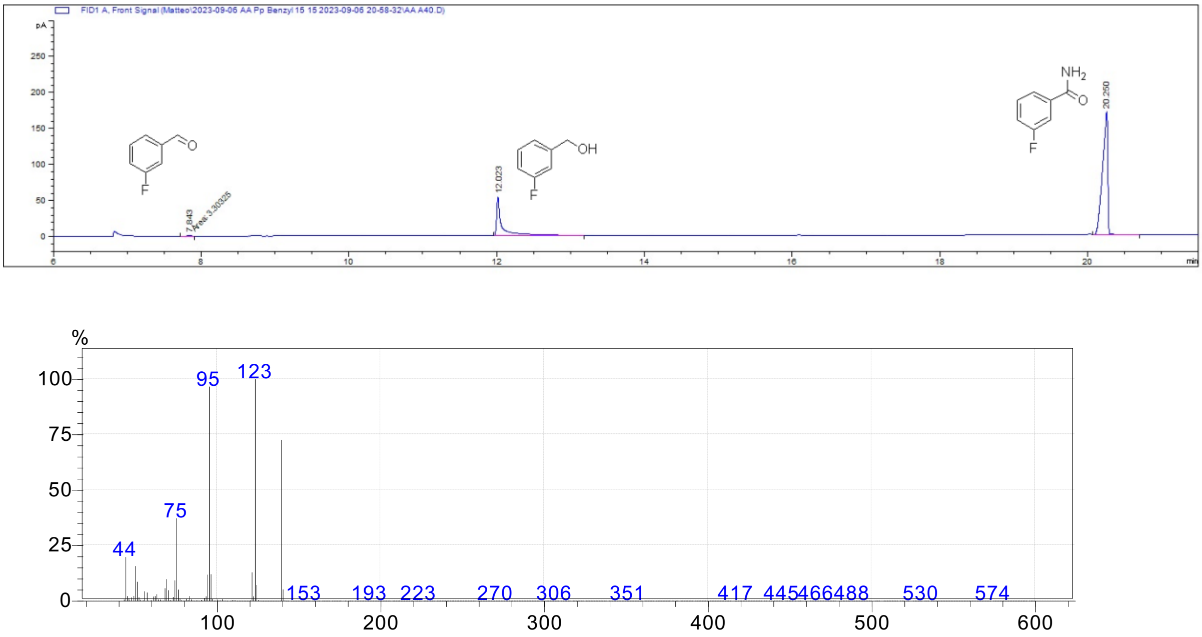

Figure S28: Up) GC-FID chromatogram for the determination of the conversion of 22a with Aa-ADH. Bottom) GC-MS of 22d.

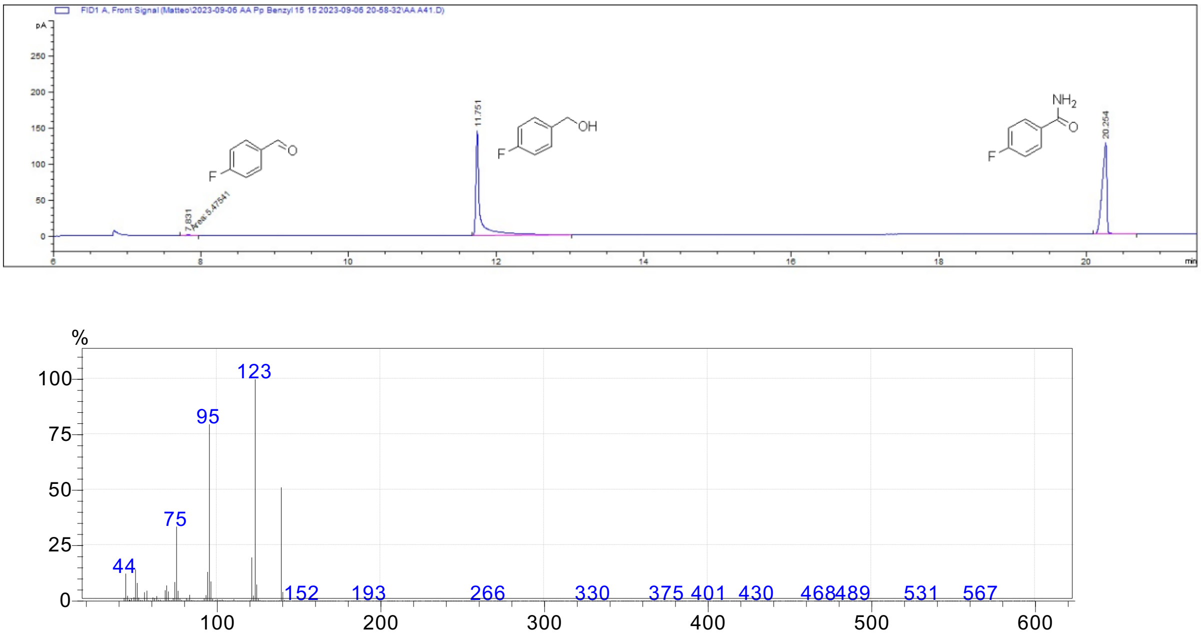

Figure S29: Up) GC-FID chromatogram for the determination of the conversion of 23a with Aa-ADH. Bottom) GC-MS of 23d.

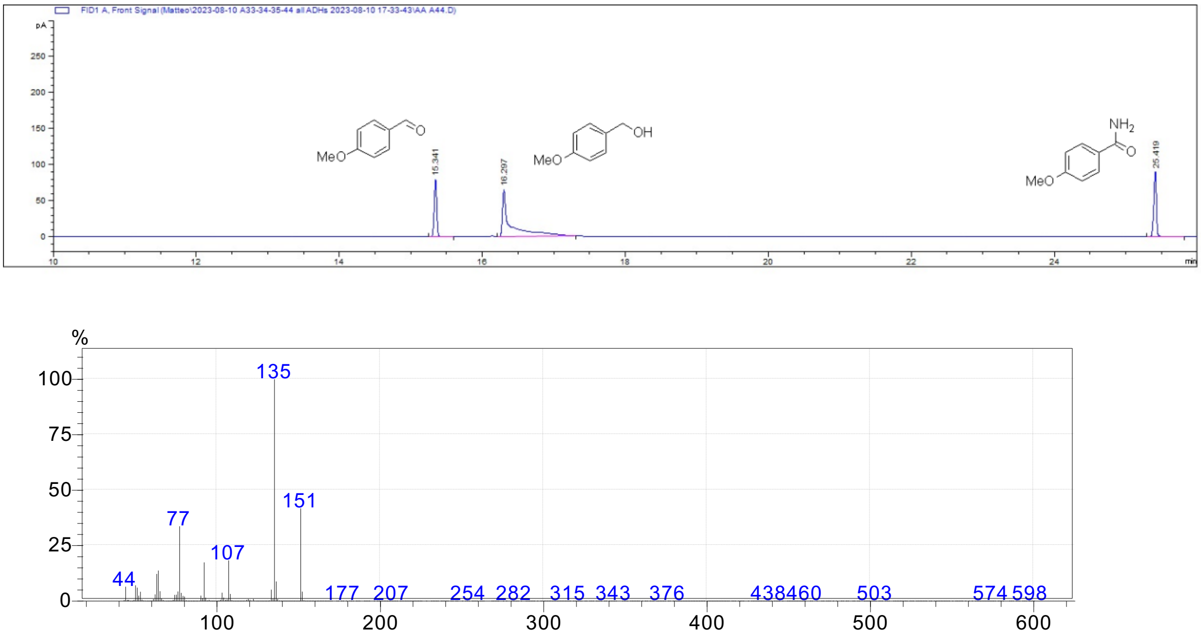

Figure S30: Up) GC-FID chromatogram for the determination of the conversion of 24a with Aa-ADH. Bottom) GC-MS of 24d.

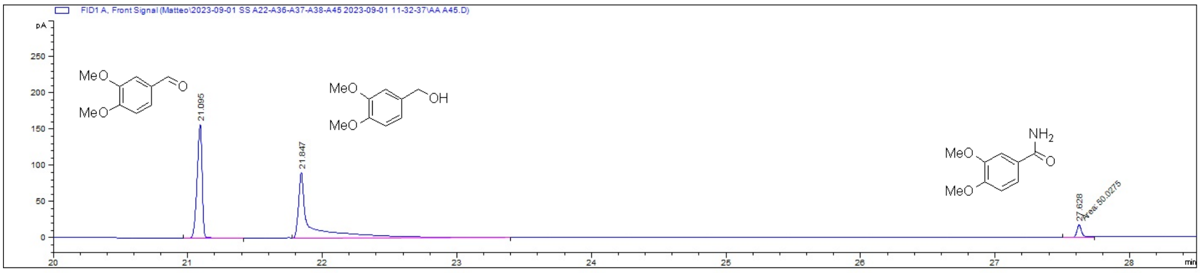

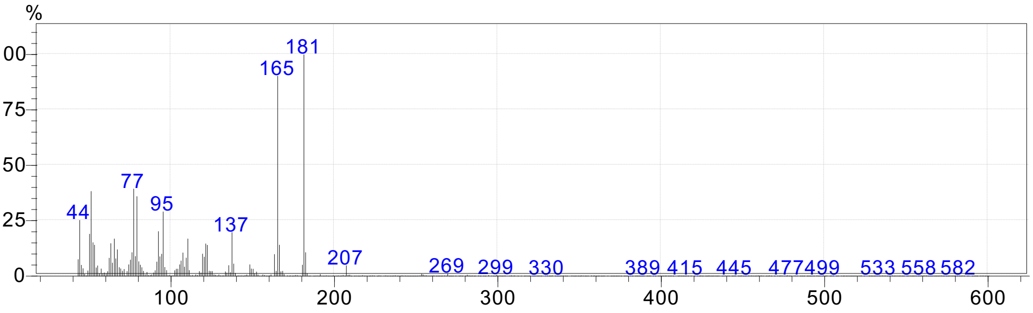

Figure S31: Up) GC-FID chromatogram for the determination of the conversion of 25a with Aa-ADH. Bottom) GC-MS of 25d.

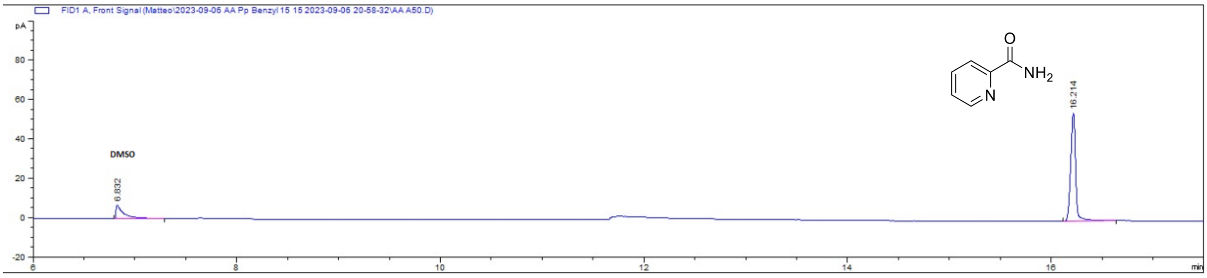

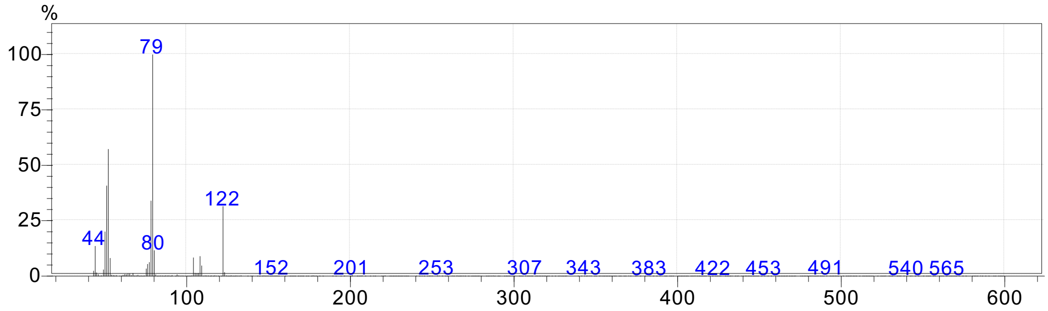

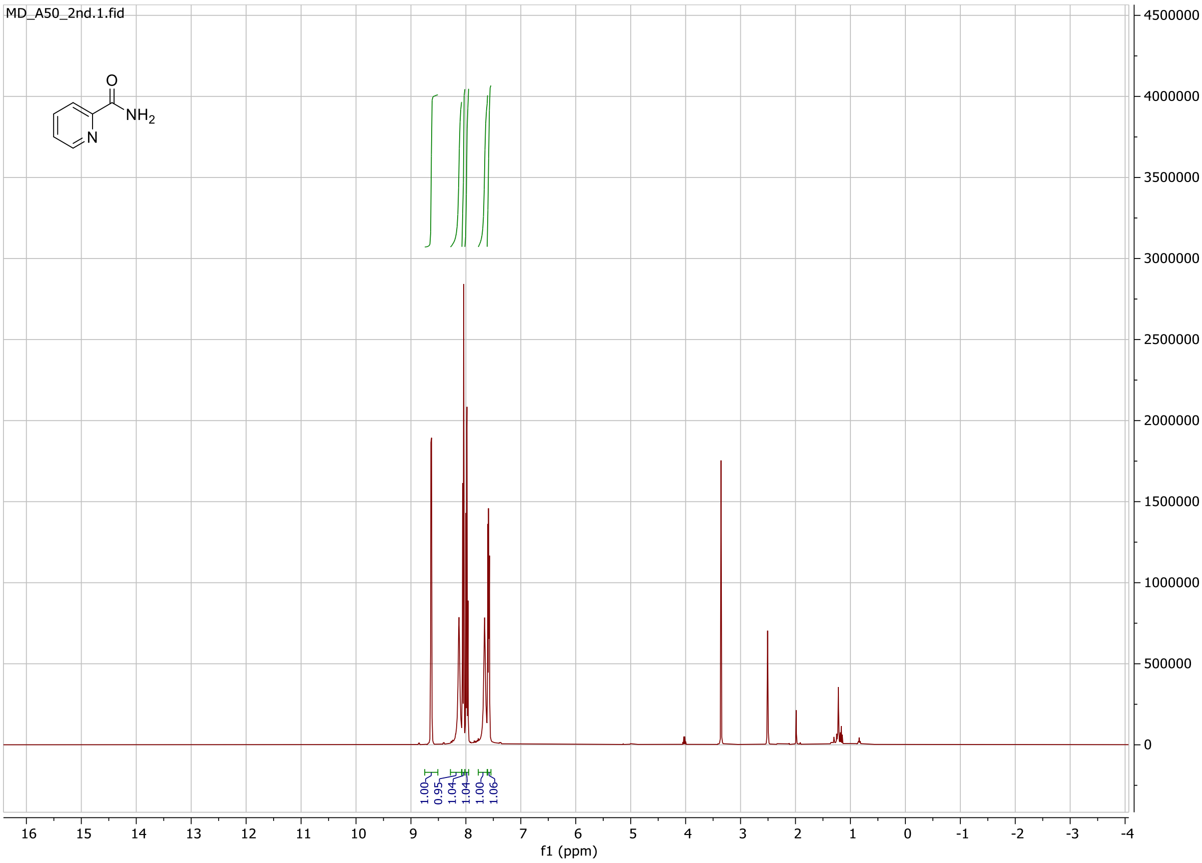

Figure S32: Up) GC-FID chromatogram for the determination of the conversion of 26a with Aa-ADH. Middle) GC-MS of 26d. Bottom) ^{1}H-NMR of 26d.

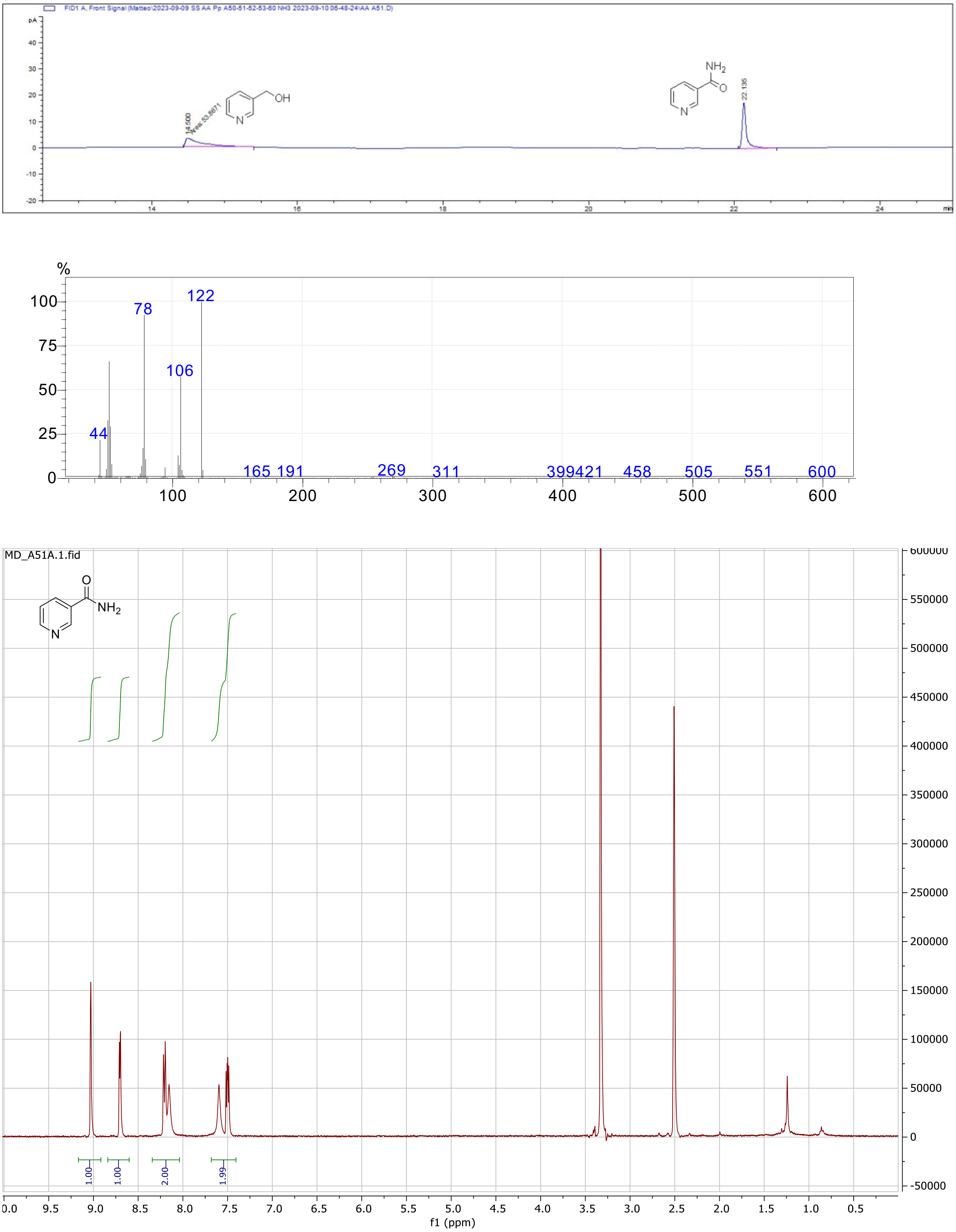

Figure S33: Up) GC-FID chromatogram for the determination of the conversion of 27a with Aa-ADH. Middle) GC-MS of 27d. Bottom) ^{1}H-NMR of 27d.

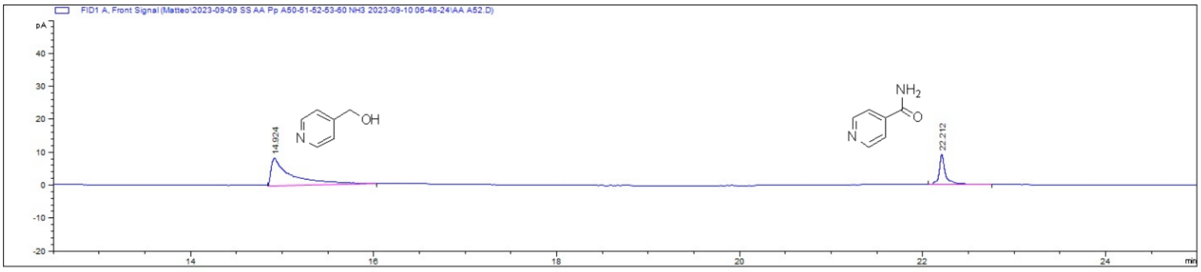

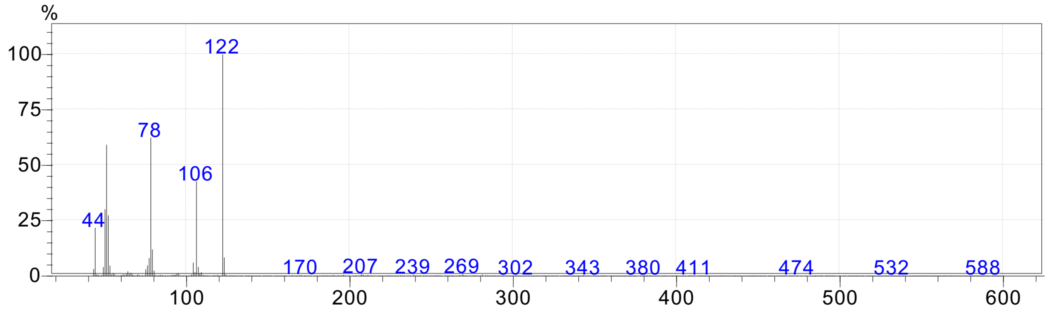

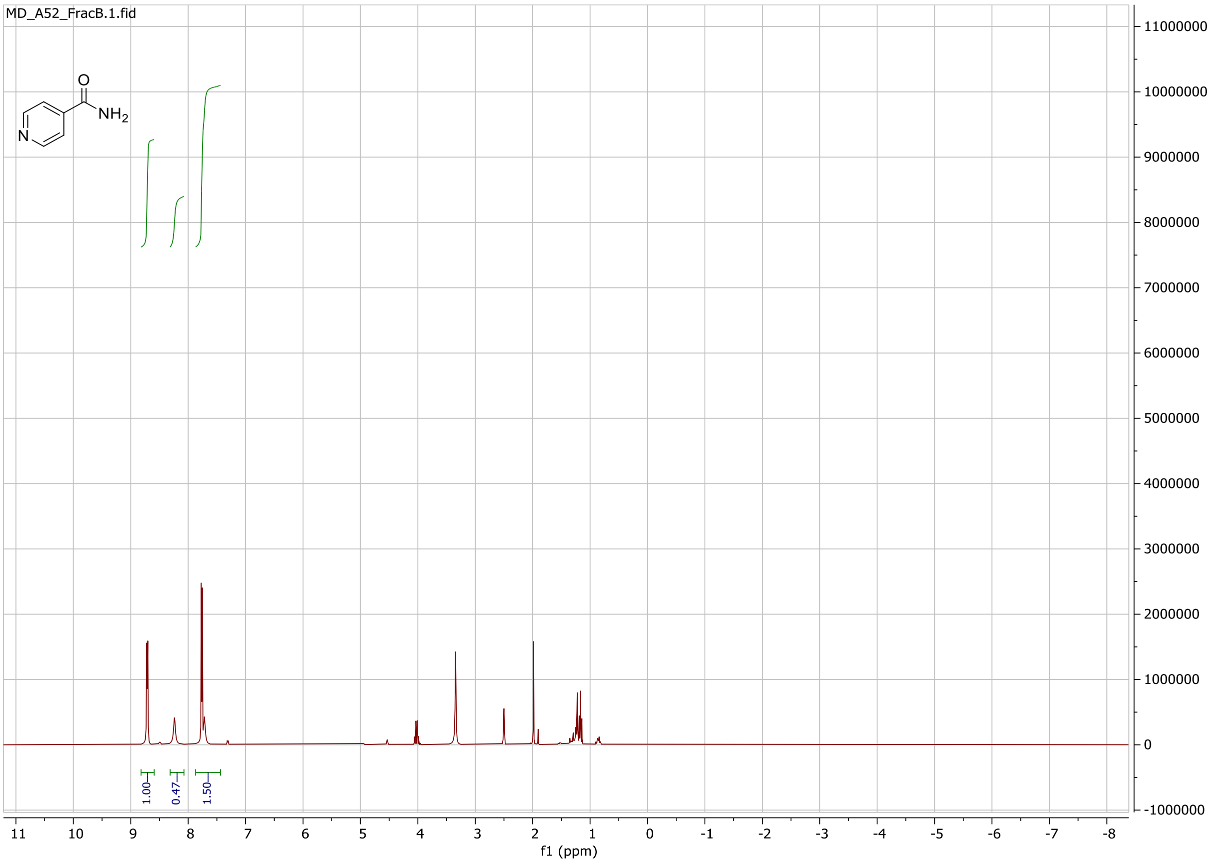

Figure S34: Up) GC-FID chromatogram for the determination of the conversion of 28a with Aa-ADH. Middle) GC-MS of 28d. Bottom) ^{1}H-NMR of 28d.

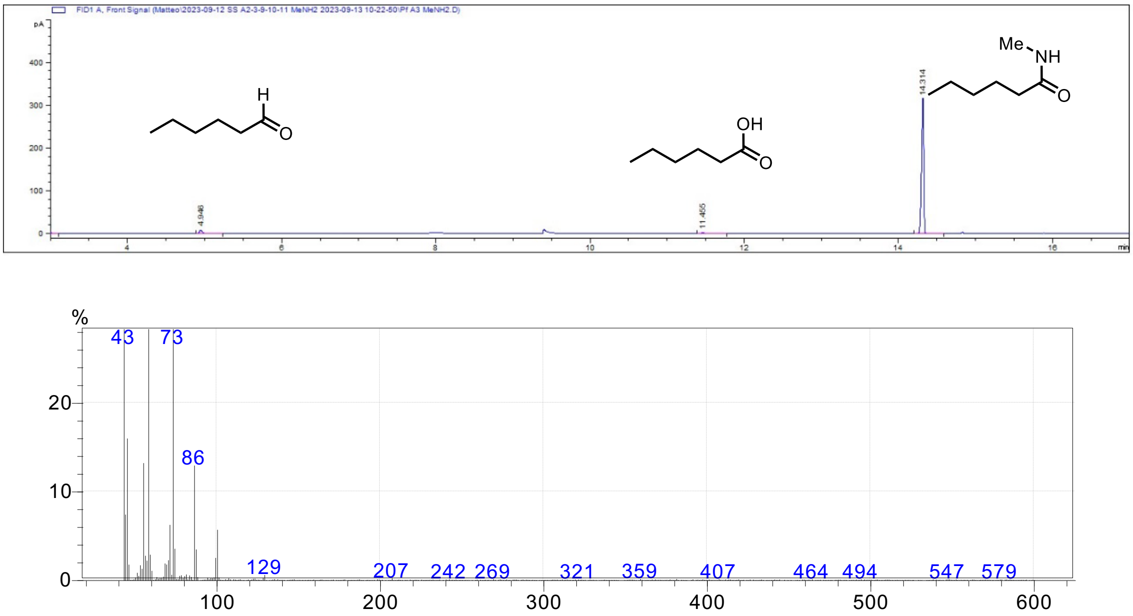

Figure S35: Up) GC-FID chromatogram for the determination of the conversion of 1a in methylammonium formate with Pf-ADH. Bottom) GC-MS of 1e.

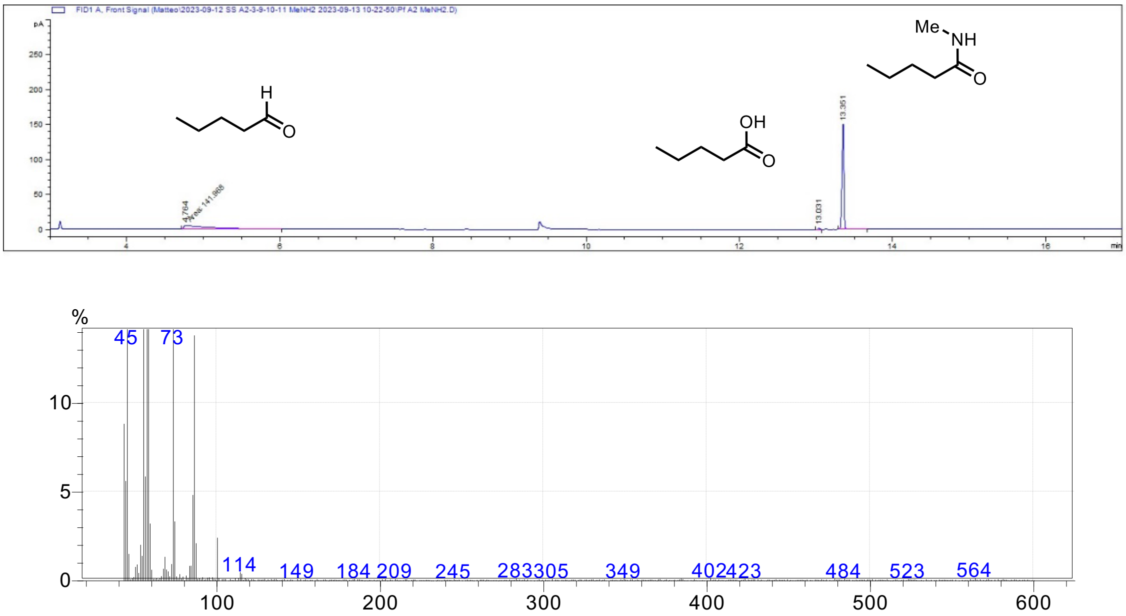

Figure S36: Up) GC-FID chromatogram for the determination of the conversion of 2a in methylammonium formate with Pf-ADH. Bottom) GC-MS of 2e.

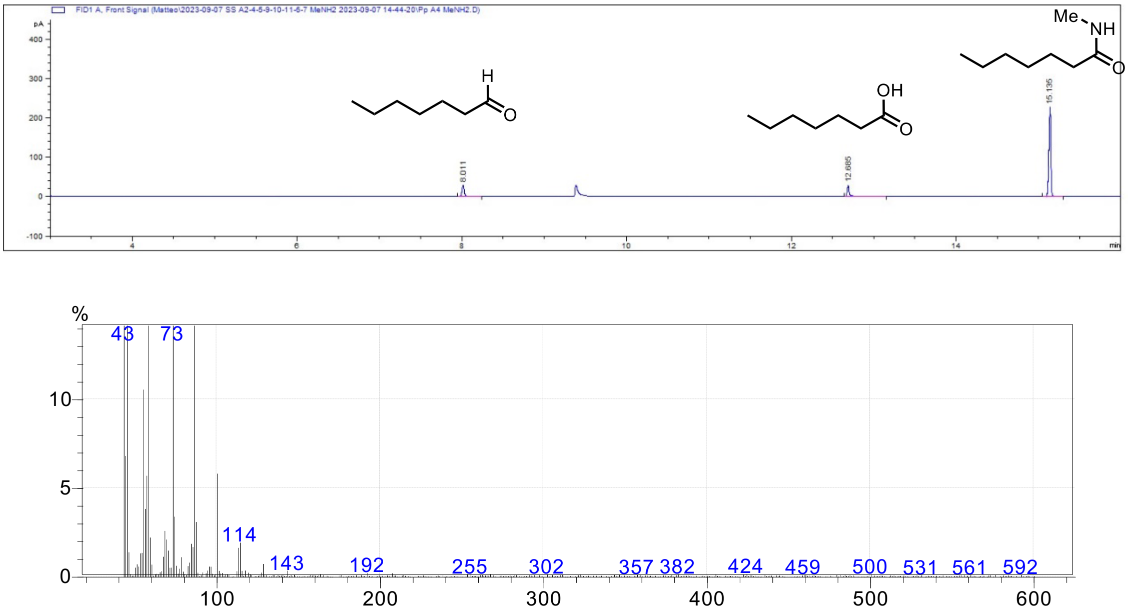

Figure S37: Up) GC-FID chromatogram for the determination of the conversion of 3a in methylammonium formate with Pp-ADH. Bottom) GC-MS of 3e.

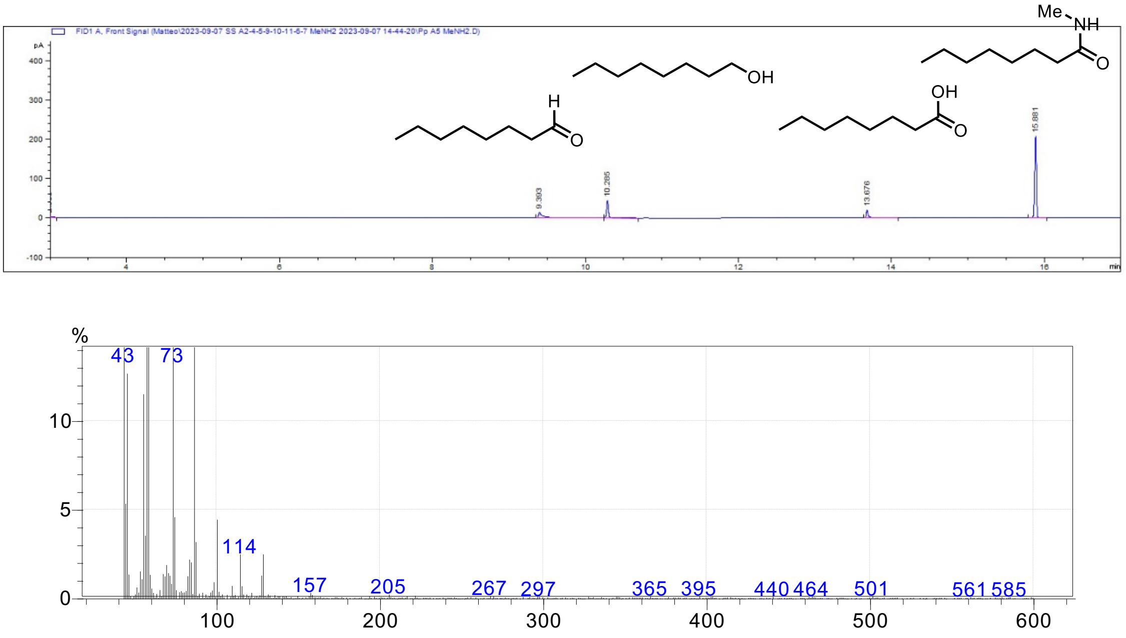

Figure S38: Up) GC-FID chromatogram for the determination of the conversion of 4a in methylammonium formate with Pp-ADH. Bottom) GC-MS of 4e.

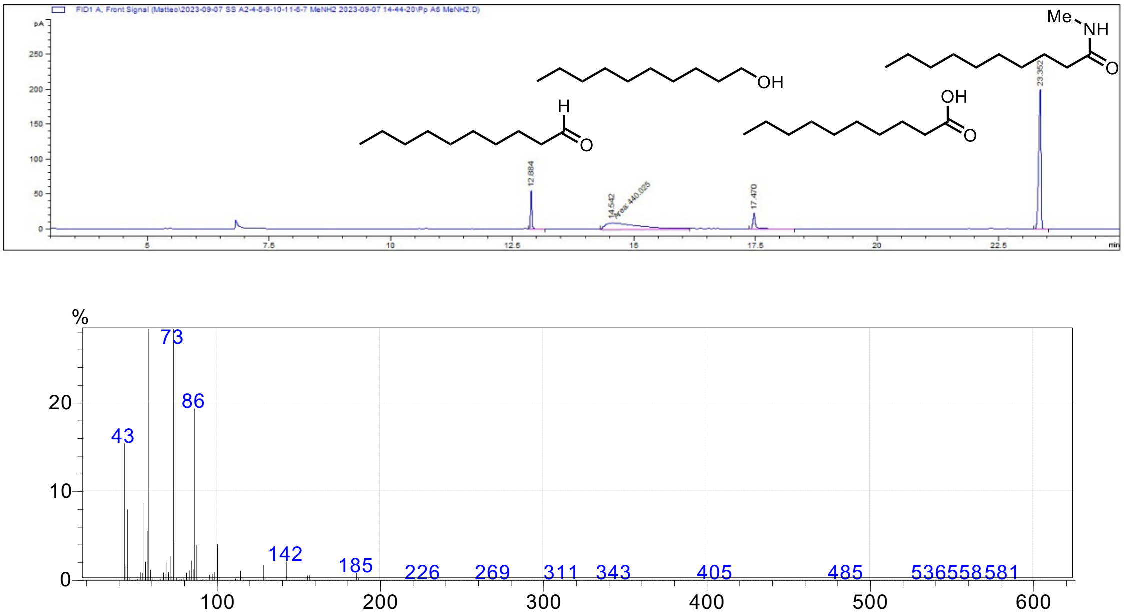

Figure S39: Up) GC-FID chromatogram for the determination of the conversion of 5a in methylammonium formate with Pp-ADH. Bottom) GC-MS of 5e.

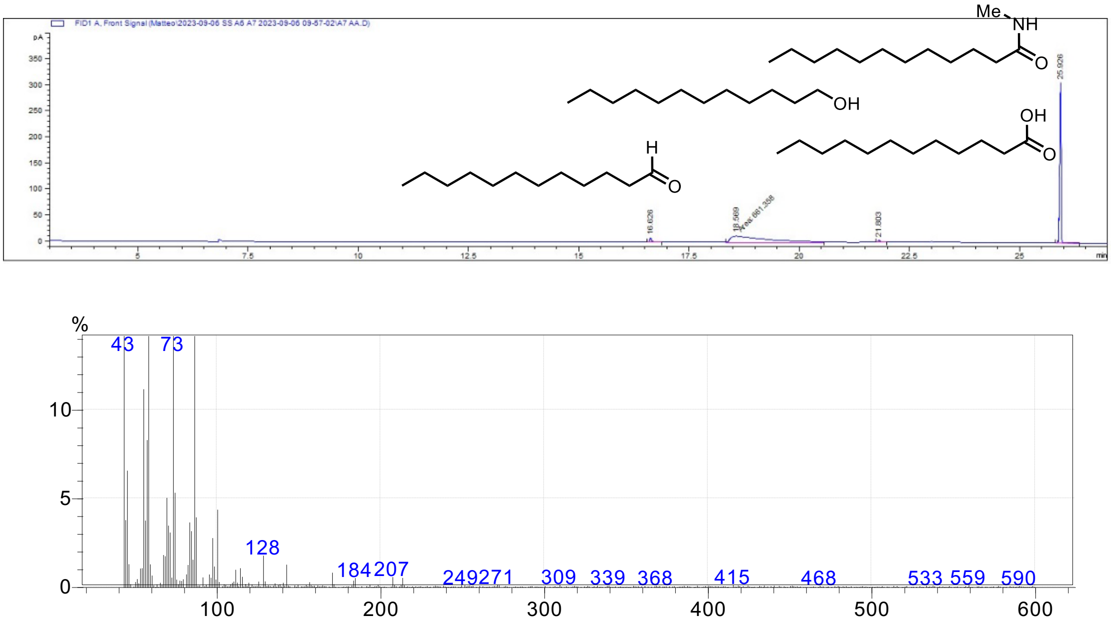

Figure S40: Up) GC-FID chromatogram for the determination of the conversion of 6a in methylammonium formate with Aa-ADH. Bottom) GC-MS of 6e.

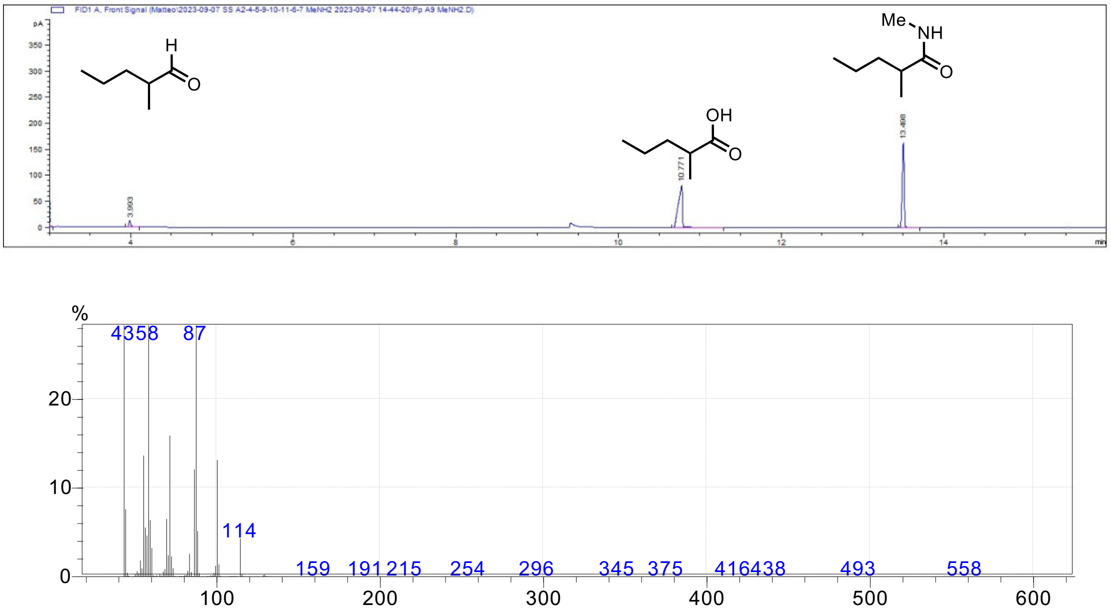

Figure S41: Up) GC-FID chromatogram for the determination of the conversion of 7a in methylammonium formate with Pp-ADH. Bottom) GC-MS of 7e.

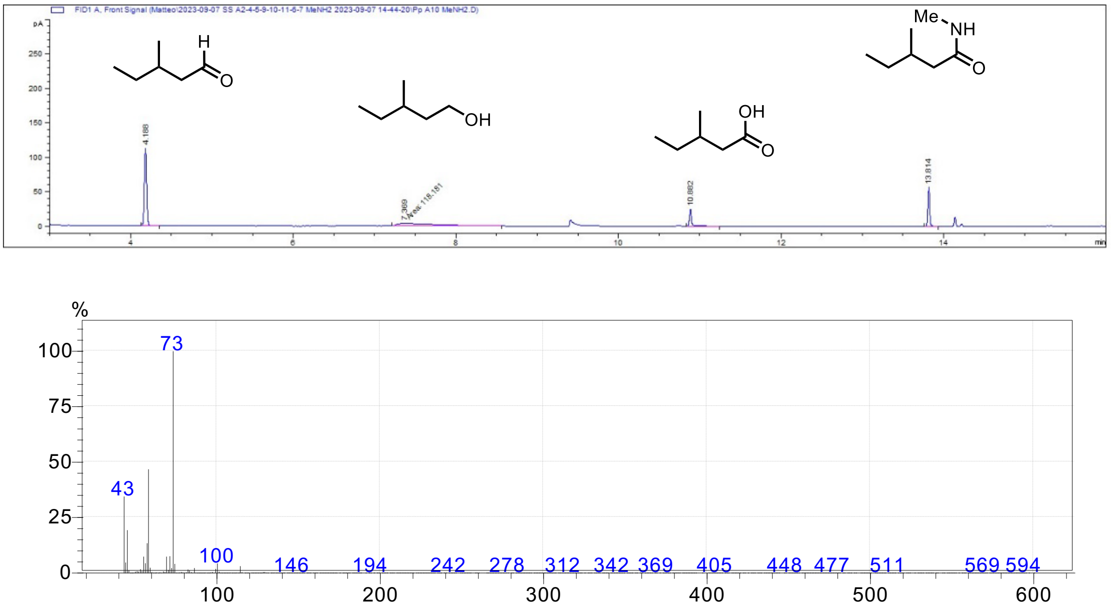

Figure S42: Up) GC-FID chromatogram for the determination of the conversion of 8a in methylammonium formate with Pp-ADH. Bottom) GC-MS of 8e.

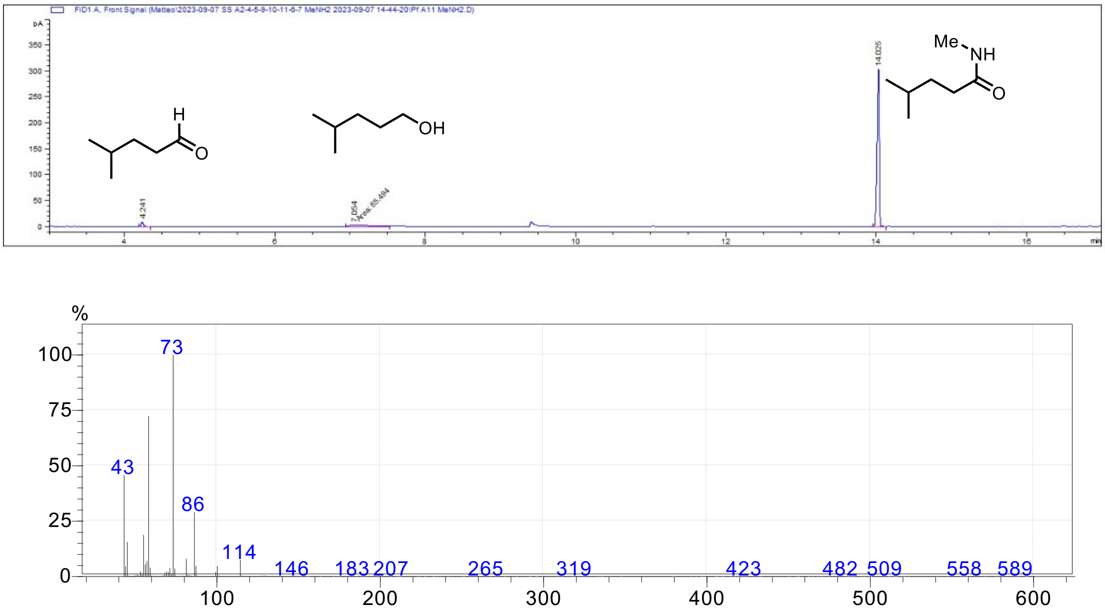

Figure S43: Up) GC-FID chromatogram for the determination of the conversion of 9a in methylammonium formate with Pf-ADH. Bottom) GC-MS of 9e.

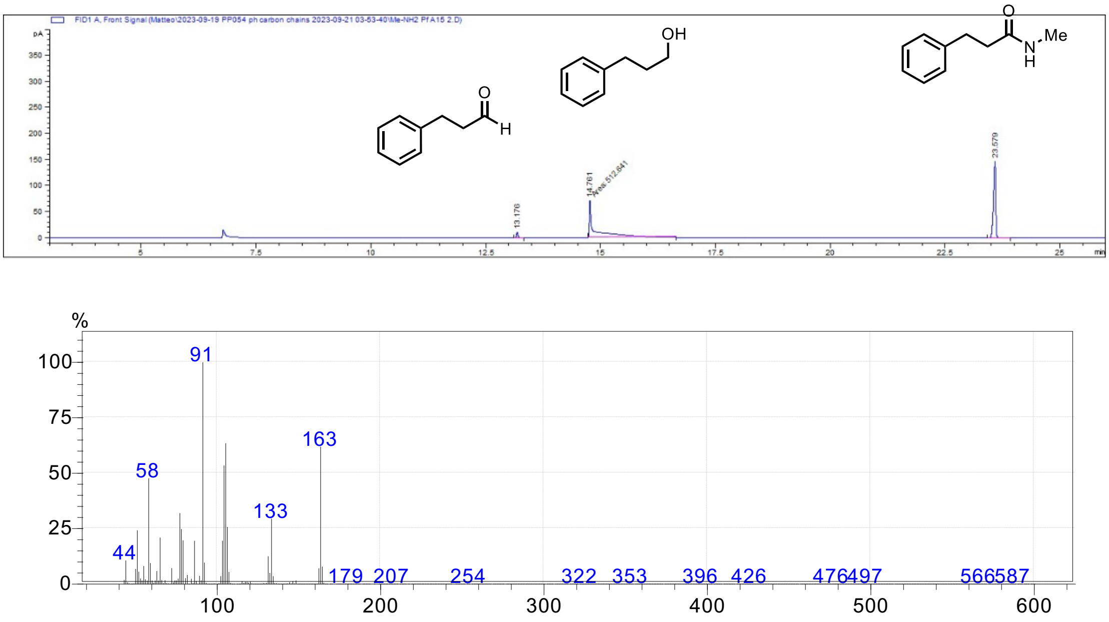

Figure S44: Up) GC-FID chromatogram for the determination of the conversion of 10a in methylammonium formate with Pf-ADH. Bottom) GC-MS of 10e.

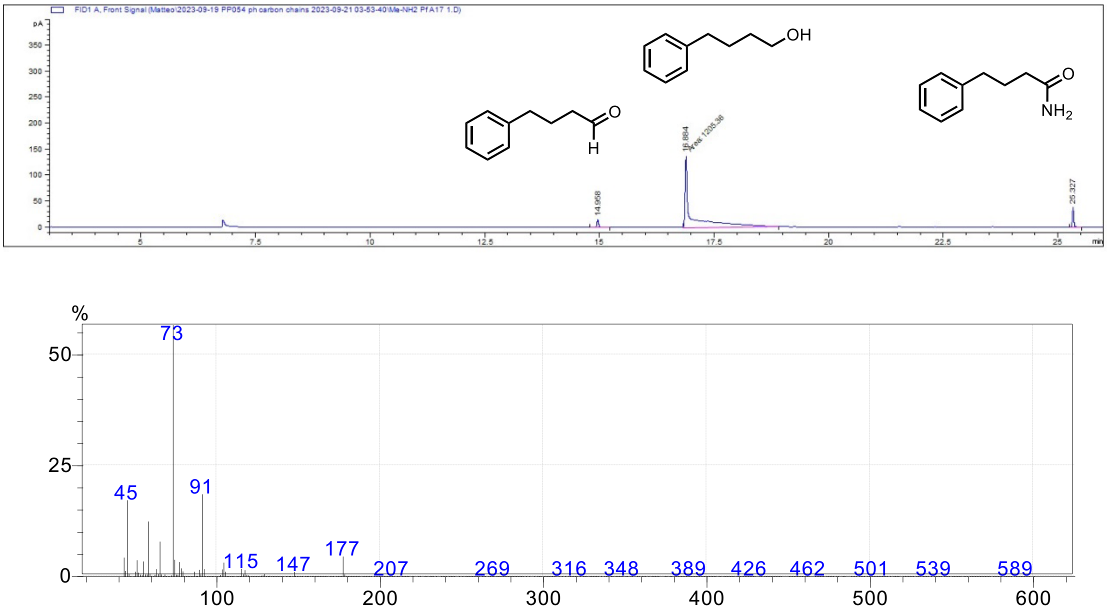

Figure S45: Up) GC-FID chromatogram for the determination of the conversion of 11a in methylammonium formate with Pf-ADH. Bottom) GC-MS of 11e.

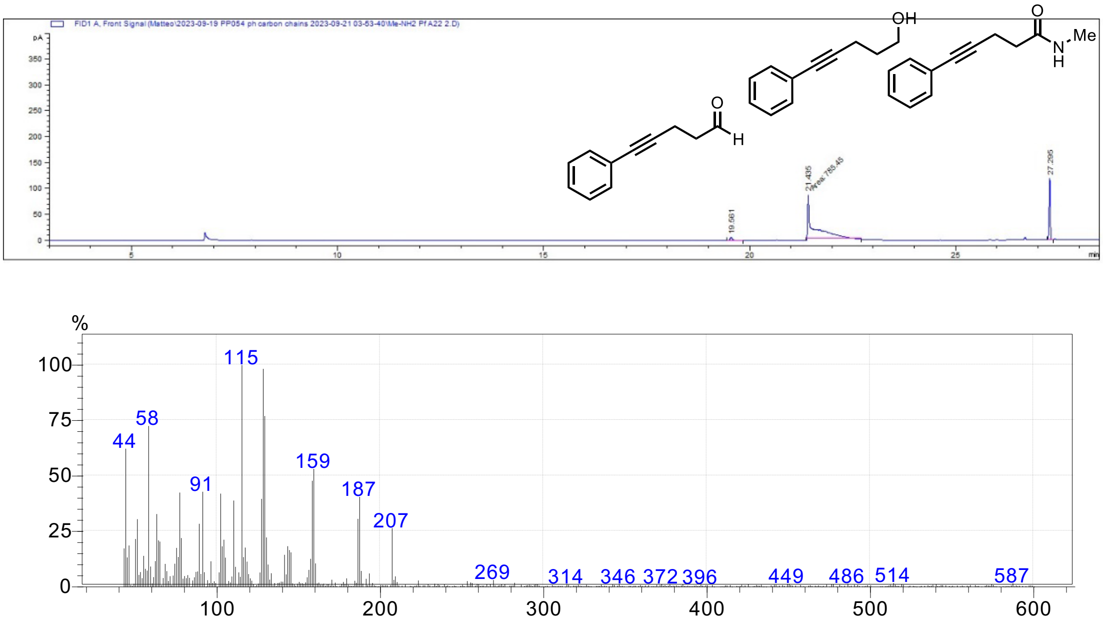

Figure S46: Up) GC-FID chromatogram for the determination of the conversion of 12a in methylammonium formate with Pf-ADH. Bottom) GC-MS of 12e.

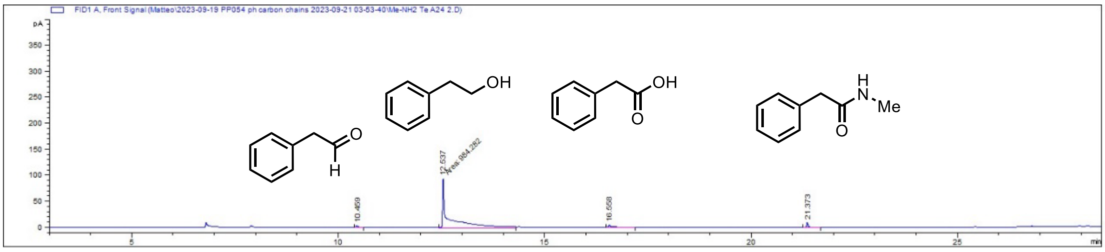

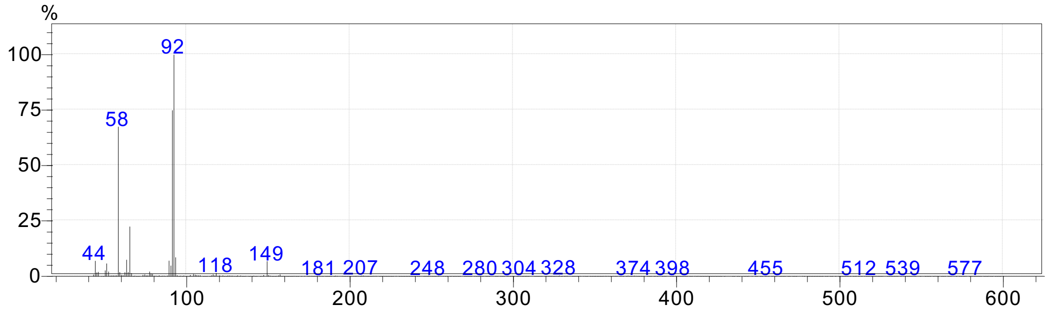

Figure S47: Up) GC-FID chromatogram for the determination of the conversion of 13a in methylammonium formate with Te-ADH W110A. Bottom) GC-MS of 13e.

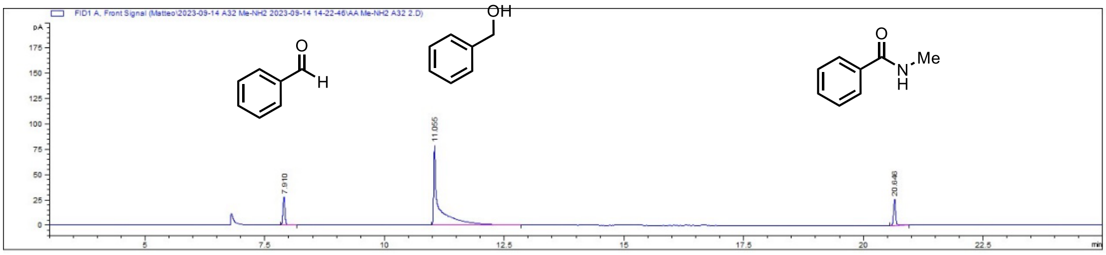

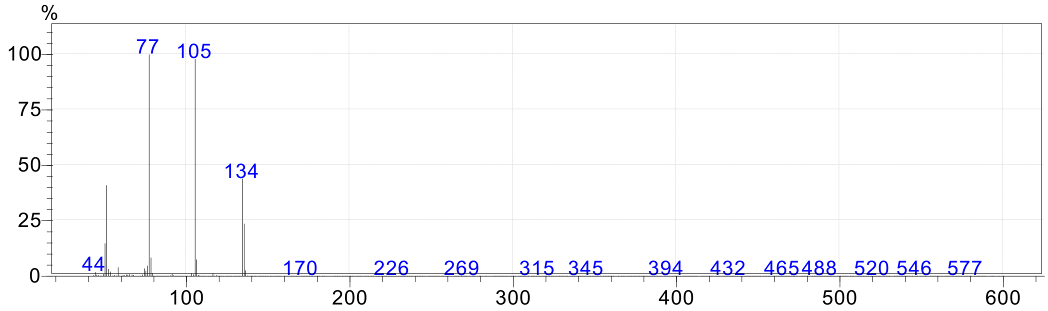

Figure S48: Up) GC-FID chromatogram for the determination of the conversion of 14a in methylammonium formate with Aa-ADH. Bottom) GC-MS of 14e.

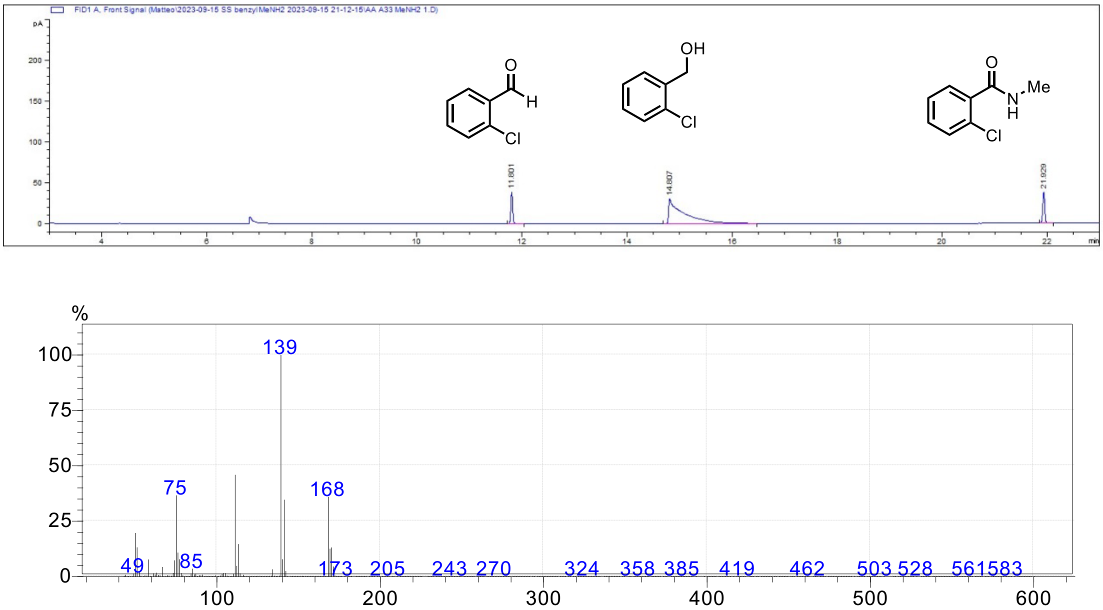

Figure S49: Up) GC-FID chromatogram for the determination of the conversion of 15a in methylammonium formate with Aa-ADH. Bottom) GC-MS of 15e.

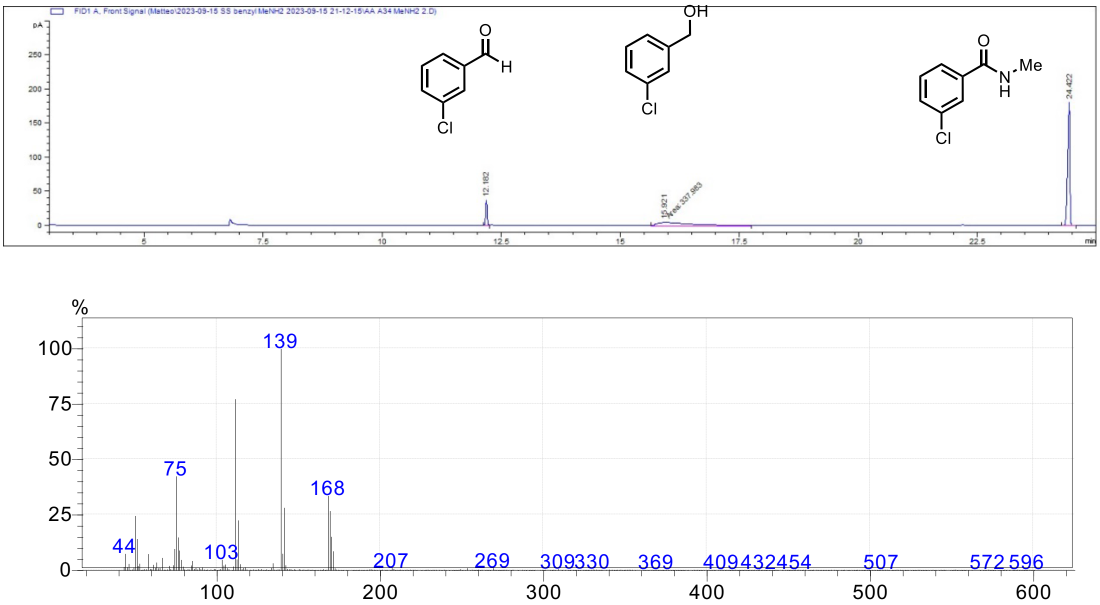

Figure S50: Up) GC-FID chromatogram for the determination of the conversion of 16a in methylammonium formate with Aa-ADH. Bottom) GC-MS of 16e.

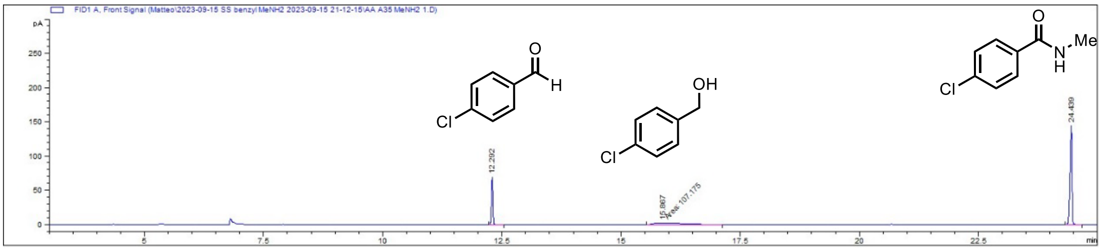

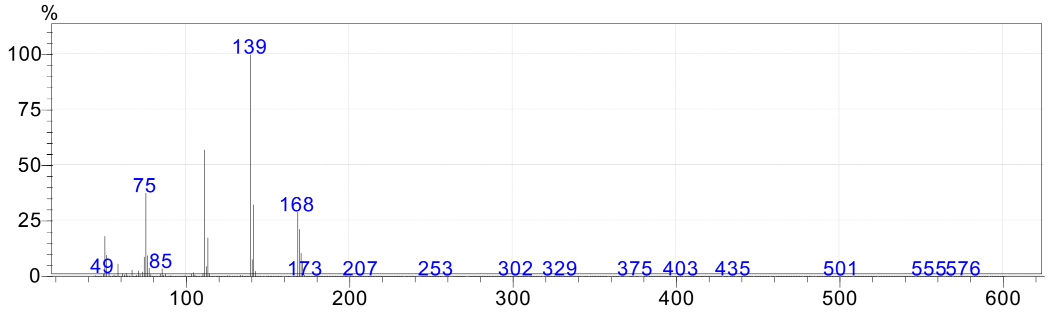

Figure S51: Up) GC-FID chromatogram for the determination of the conversion of 17a in methylammonium formate with Aa-ADH. Bottom) GC-MS of 17e.

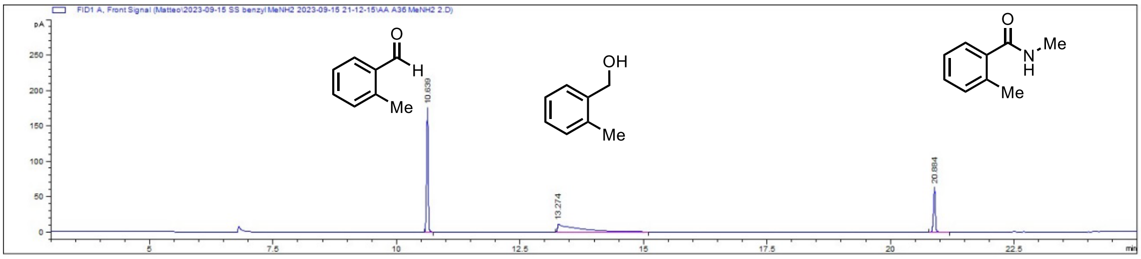

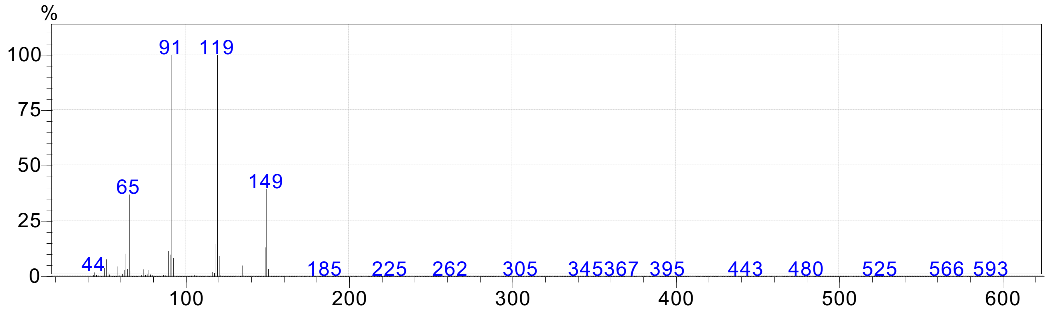

Figure S52: Up) GC-FID chromatogram for the determination of the conversion of 18a in methylammonium formate with Aa-ADH. Bottom) GC-MS of 18e.

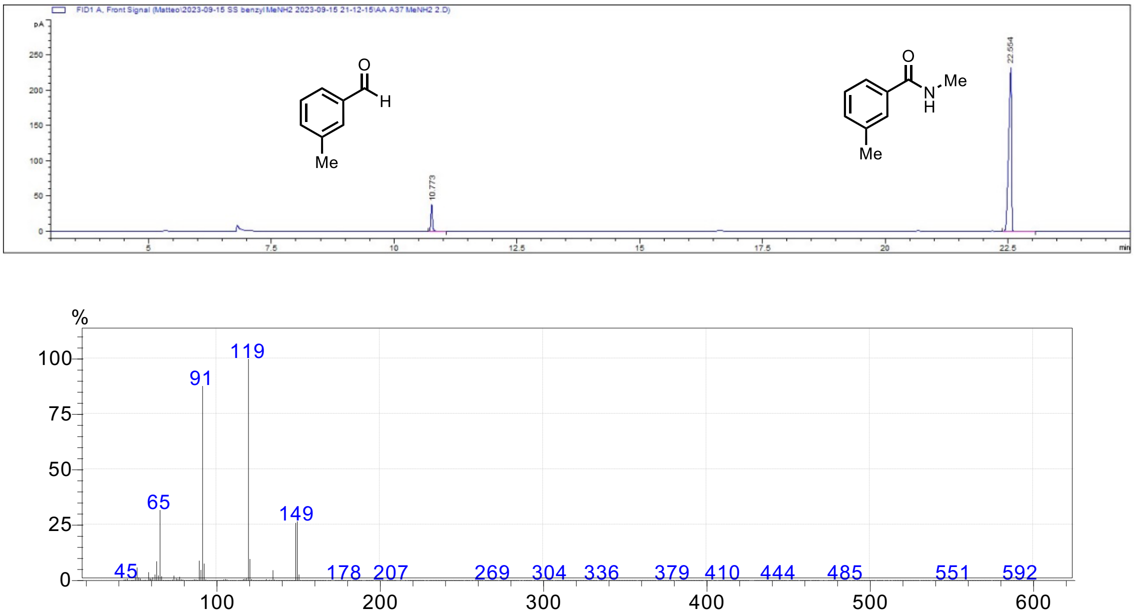

Figure S53: Up) GC-FID chromatogram for the determination of the conversion of 19a in methylammonium formate with Aa-ADH. Bottom) GC-MS of 19e.

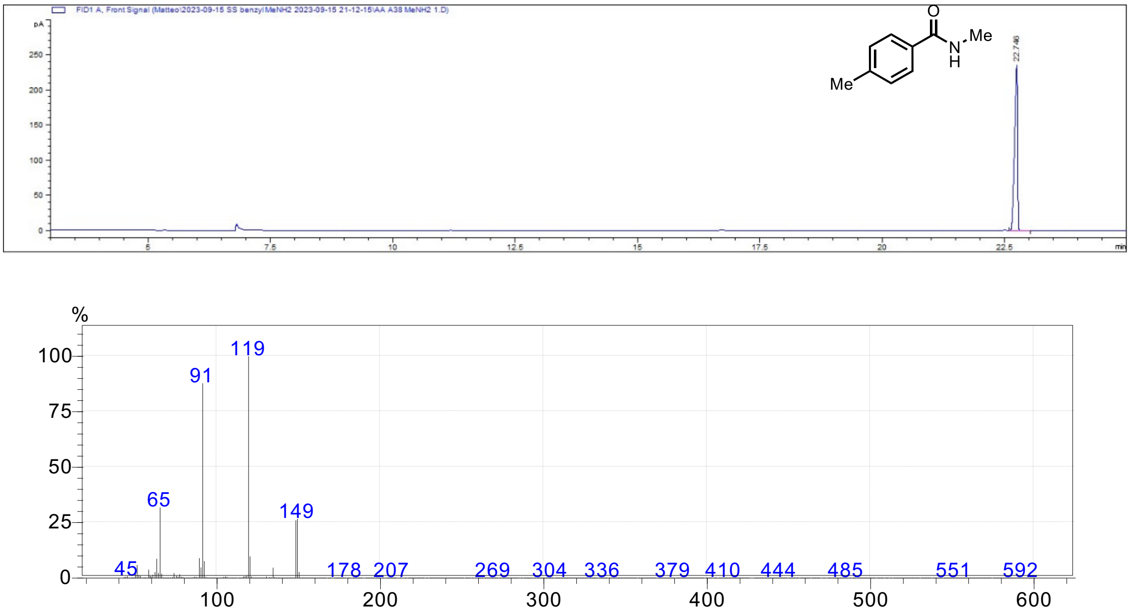

Figure S54: Up) GC-FID chromatogram for the determination of the conversion of 20a in methylammonium formate with Aa-ADH. Bottom) GC-MS of 20e.

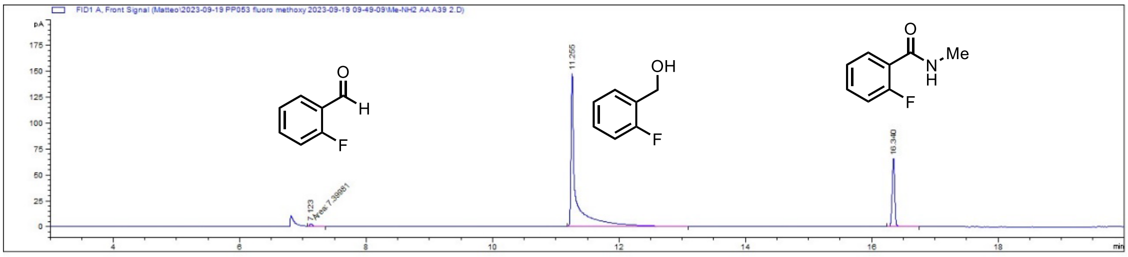

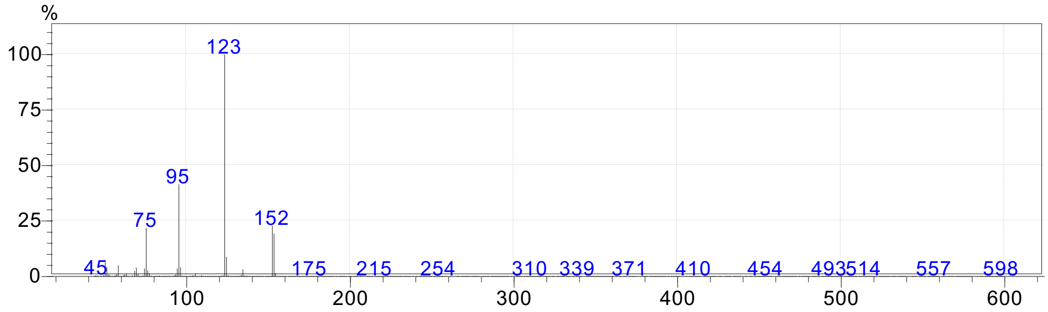

Figure S55: Up) GC-FID chromatogram for the determination of the conversion of 21a in methylammonium formate with Aa-ADH. Bottom) GC-MS of 21e.

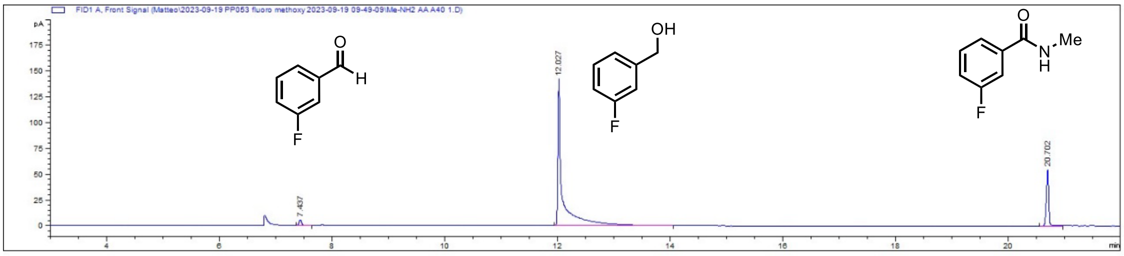

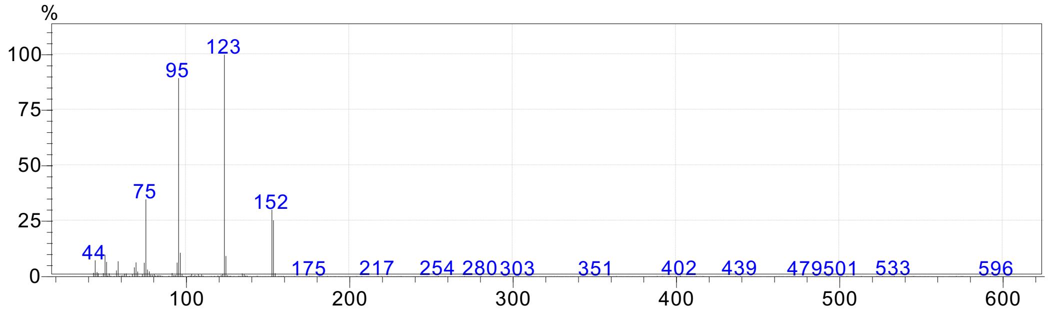

Figure S56: Up) GC-FID chromatogram for the determination of the conversion of 22a in methylammonium formate with Aa-ADH. Bottom) GC-MS of 22e.

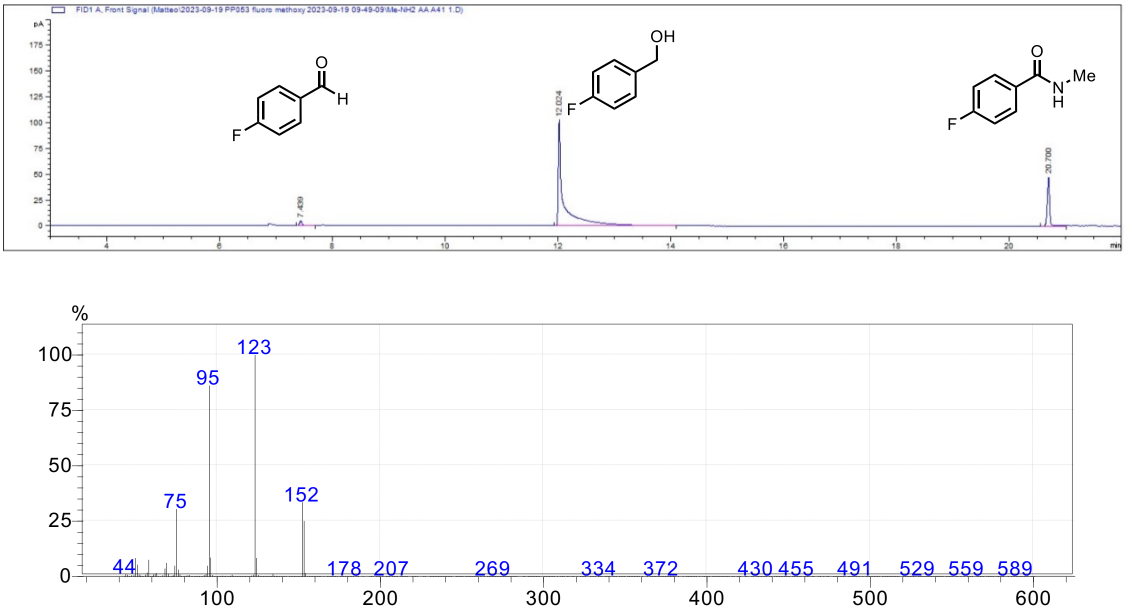

Figure S57: Up) GC-FID chromatogram for the determination of the conversion of 23a in methylammonium formate with Aa-ADH. Bottom) GC-MS of 23e.

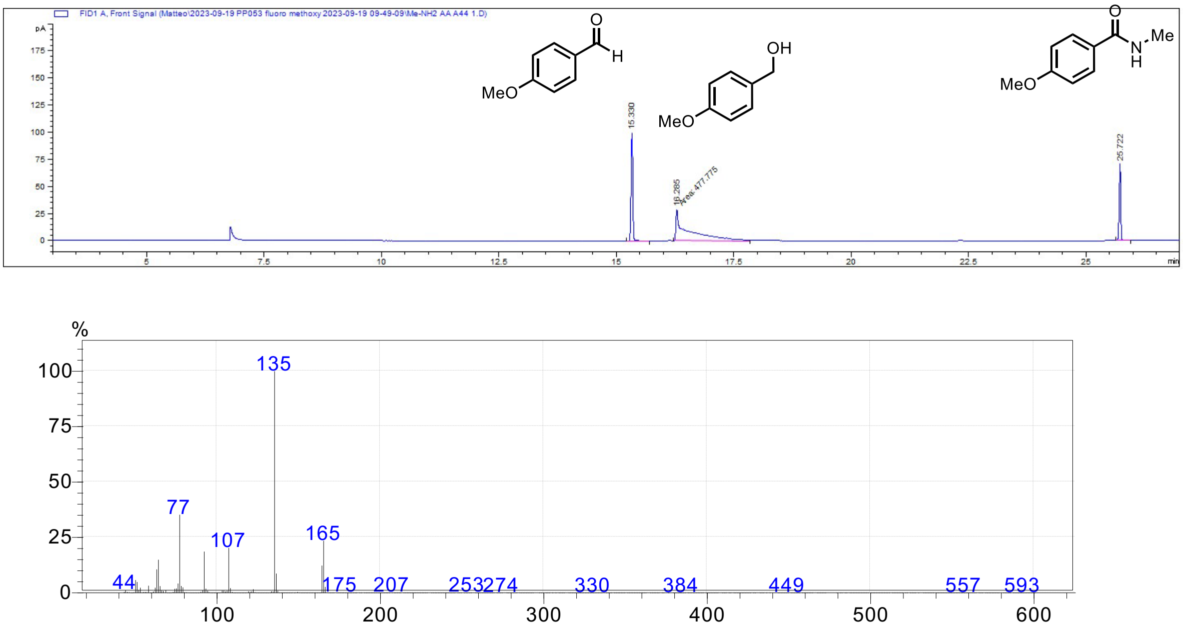

Figure S58: Up) GC-FID chromatogram for the determination of the conversion of 24a in methylammonium formate with Aa-ADH. Bottom) GC-MS of 24e.

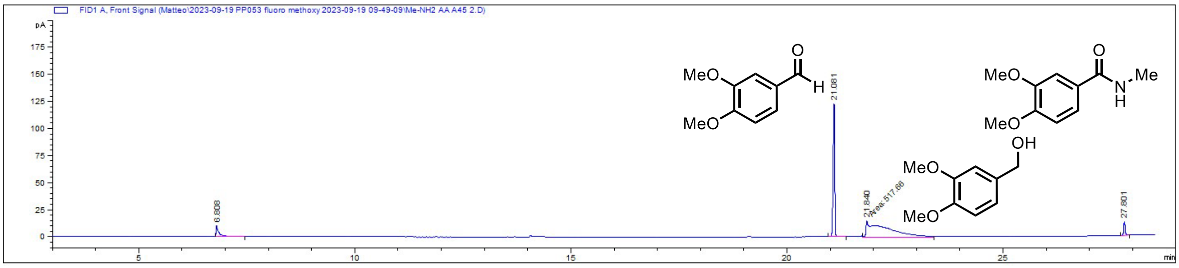

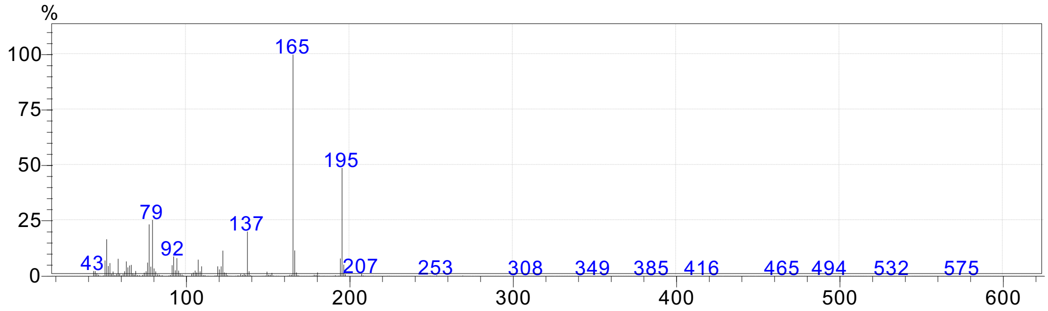

Figure S59: Up) GC-FID chromatogram for the determination of the conversion of 25a in methylammonium formate with Aa-ADH. Bottom) GC-MS of 25e.

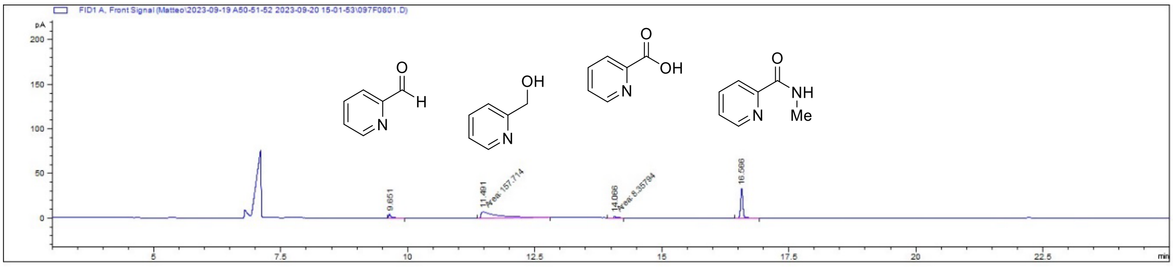

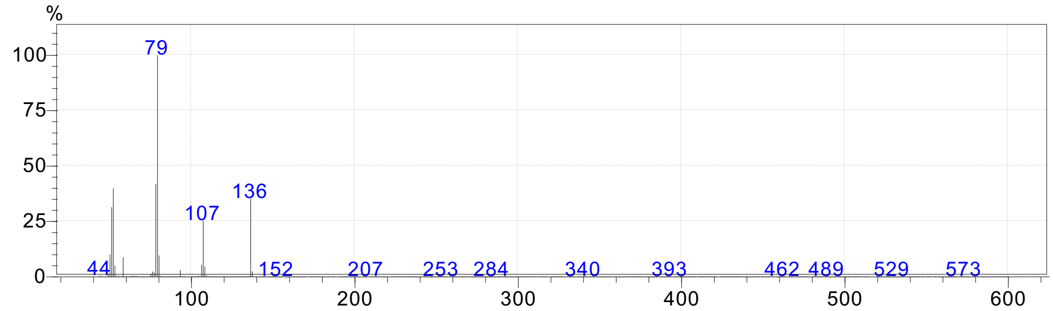

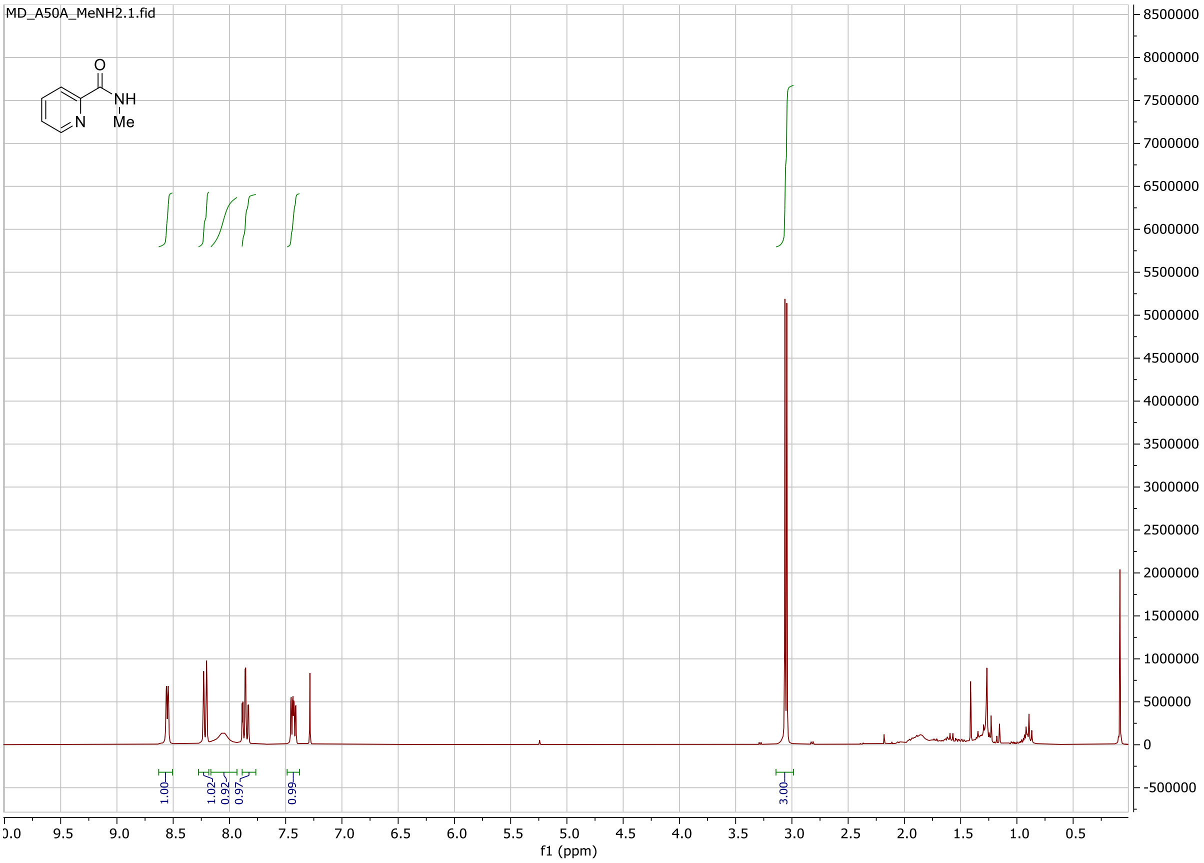

Figure S60: Up) GC-FID chromatogram for the determination of the conversion of 26a in methylammonium formate with Aa-ADH. Middle) GC-MS of 26e. Bottom) ^{1}H-NMR of 26e.

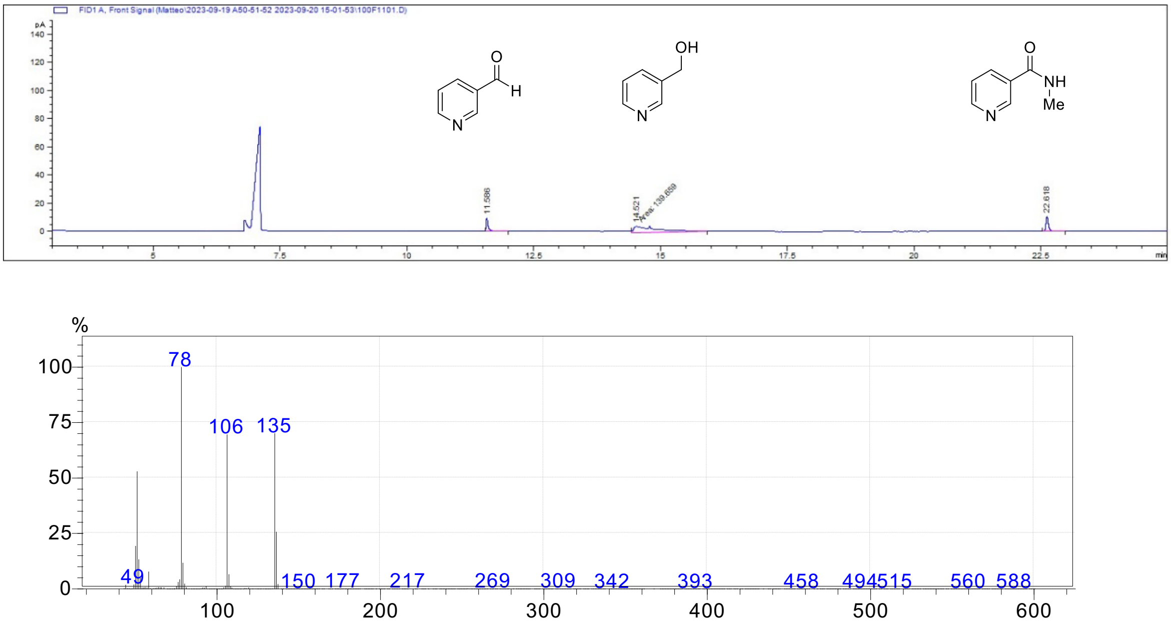

Figure S61: Up) GC-FID chromatogram for the determination of the conversion of 27a in methylammonium formate with Aa-ADH. Bottom) GC-MS of 27e.

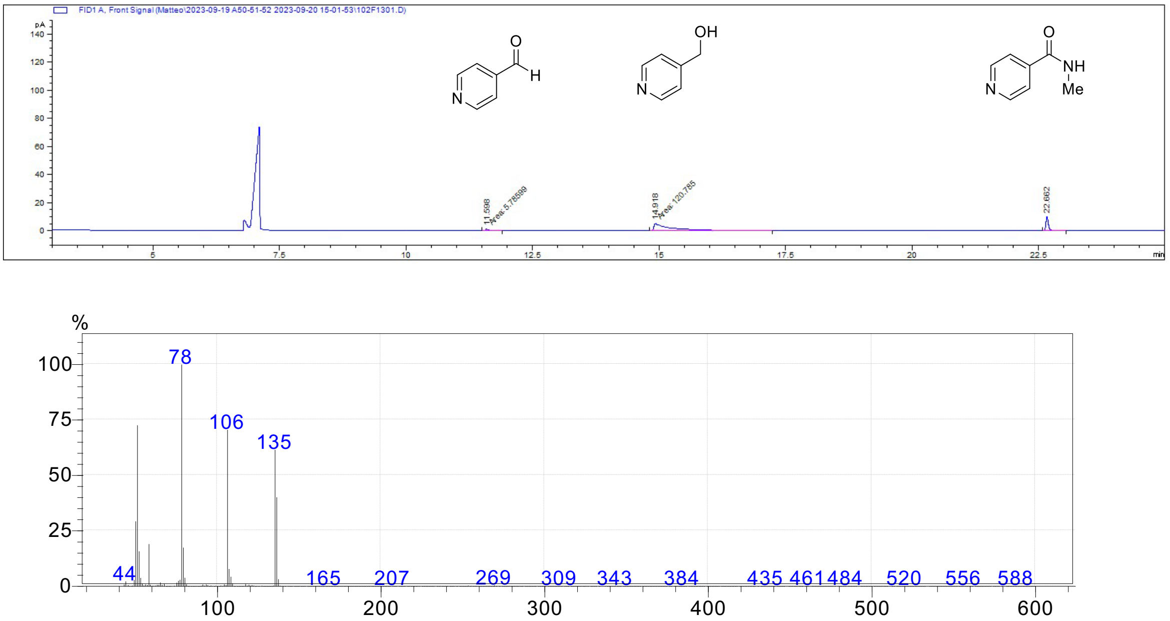

Figure S62: Up) GC-FID chromatogram for the determination of the conversion of 28a in methylammonium formate with Aa-ADH. Bottom) GC-MS of 27e.

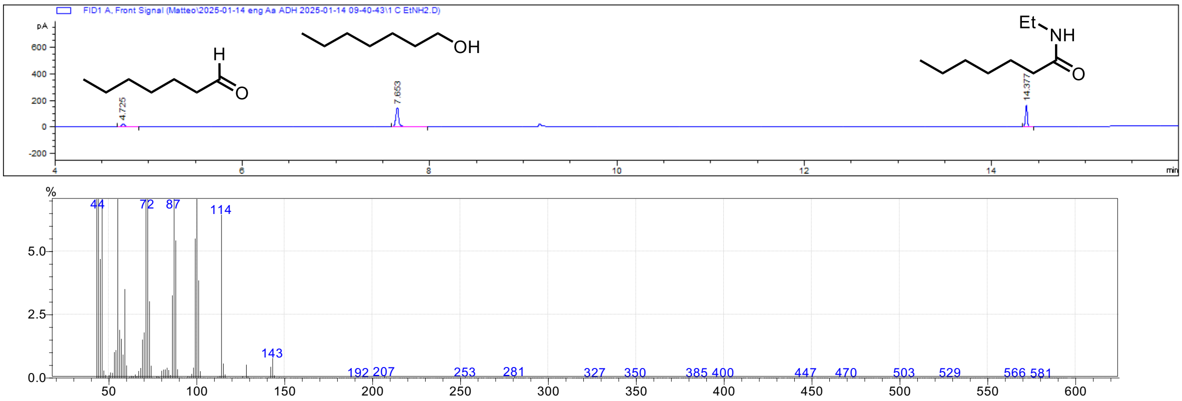

Figure S63: Up) GC-FID chromatogram for the determination of the conversion of 1i in ethylammonium formate with Aa-ADH. Bottom) GC-MS of 1i.

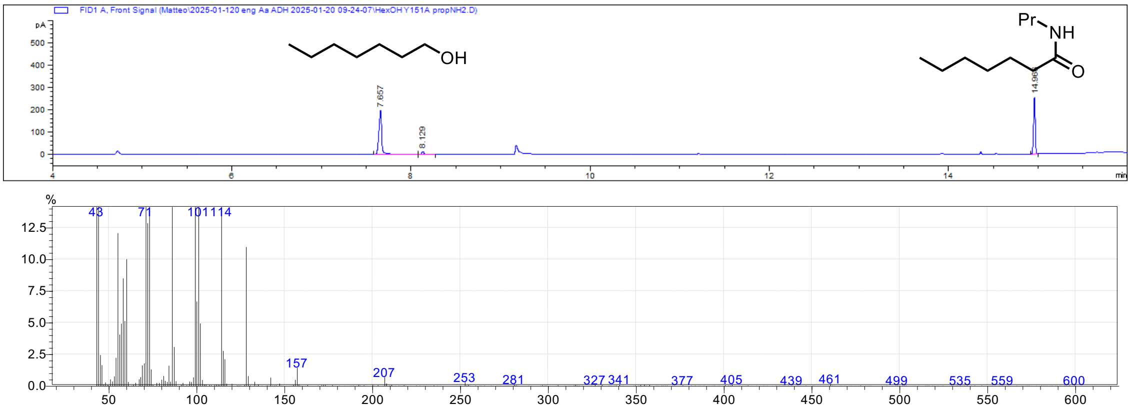

Figure S64: Up) GC-FID chromatogram for the determination of the conversion of 1j in propylammonium formate with Aa-ADH. Bottom) GC-MS of 1j.

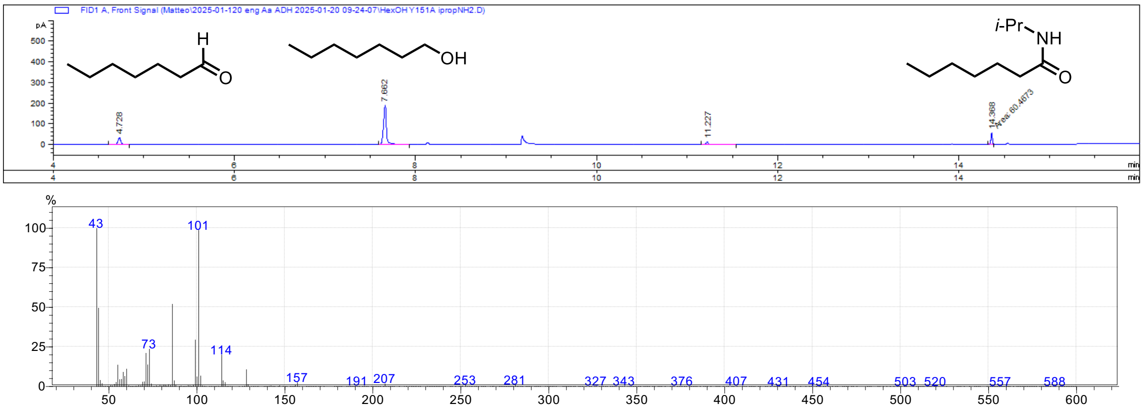

Figure S65: Up) GC-FID chromatogram for the determination of the conversion of 1k in isopropylammonium formate with Aa-ADH. Bottom) GC-MS of 1k.

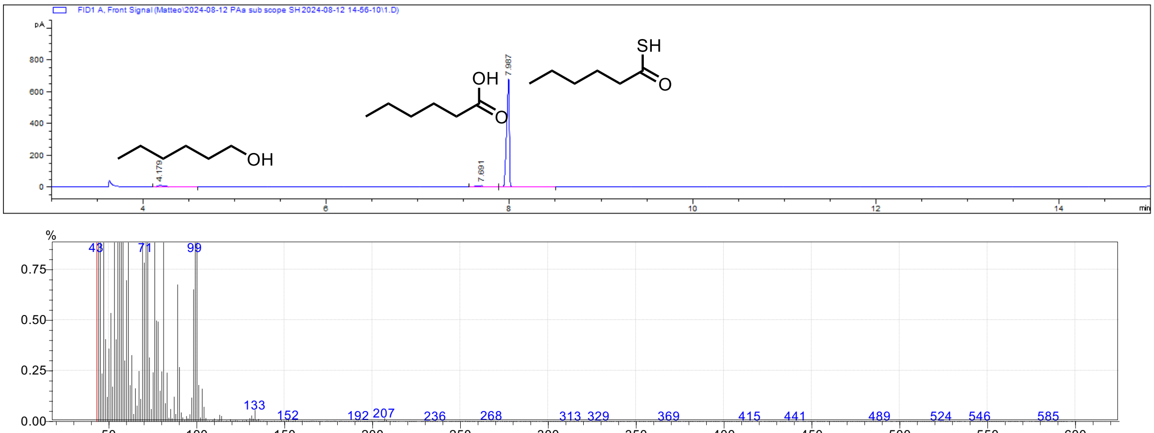

Figure S66: Up) GC-FID chromatogram for the determination of the conversion of 1f in hydrogen sulfide formate with Aa-ADH. Bottom) GC-MS of 1f.

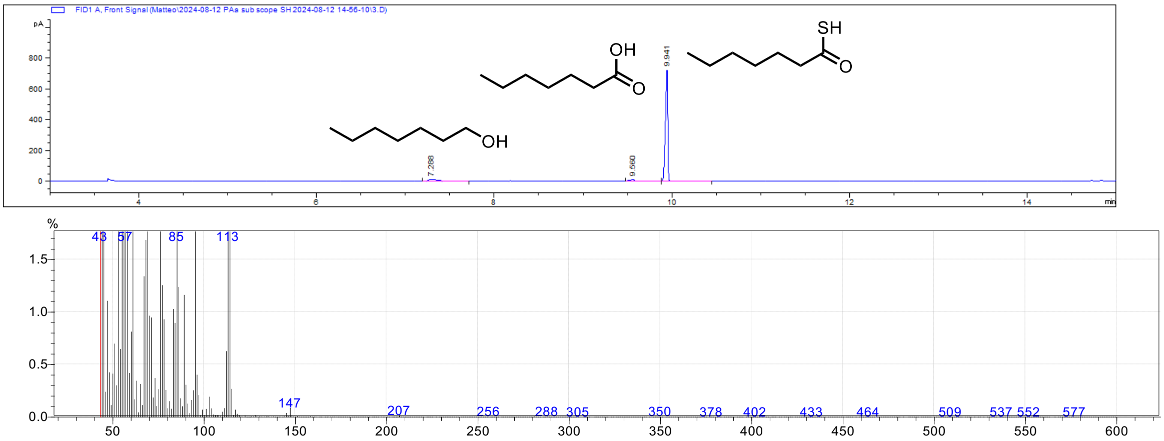

Figure S67: Up) GC-FID chromatogram for the determination of the conversion of 3f in hydrogen sulfide formate with Aa-ADH. Bottom) GC-MS of 3f.

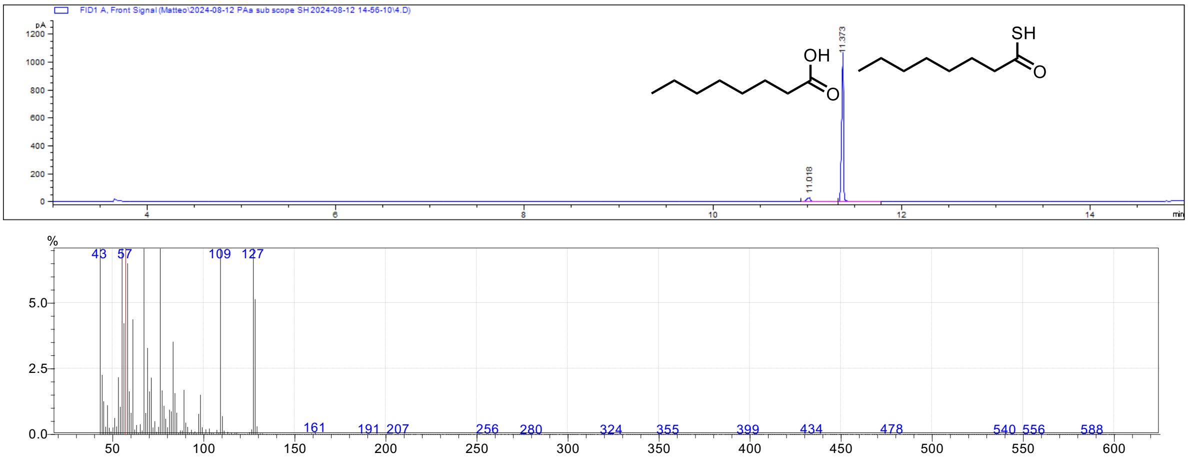

Figure S68: Up) GC-FID chromatogram for the determination of the conversion of 4f in hydrogen sulfide formate with Aa-ADH. Bottom) GC-MS of 4f.

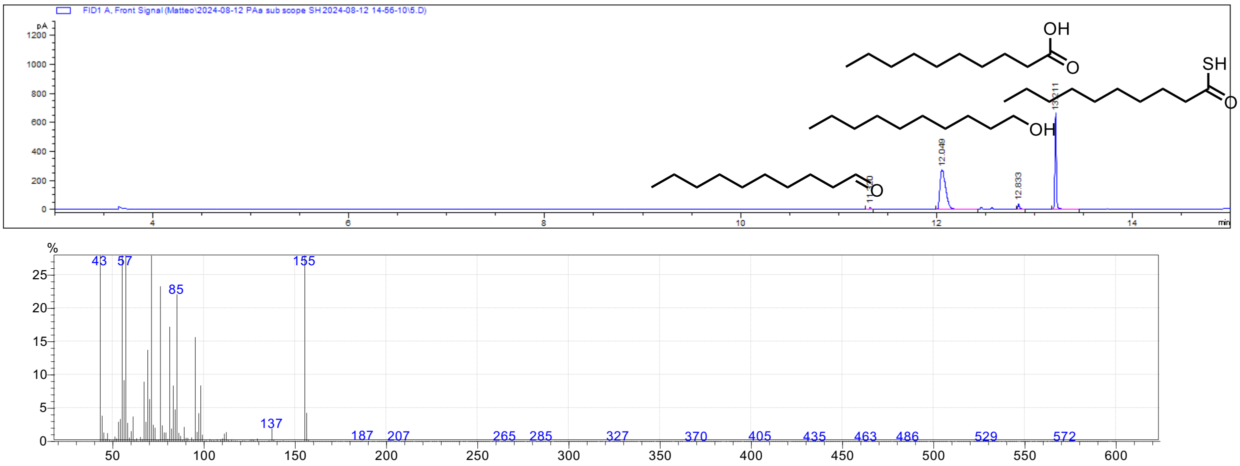

Figure S69: Up) GC-FID chromatogram for the determination of the conversion of 5f in hydrogen sulfide formate with Aa-ADH. Bottom) GC-MS of 5f.

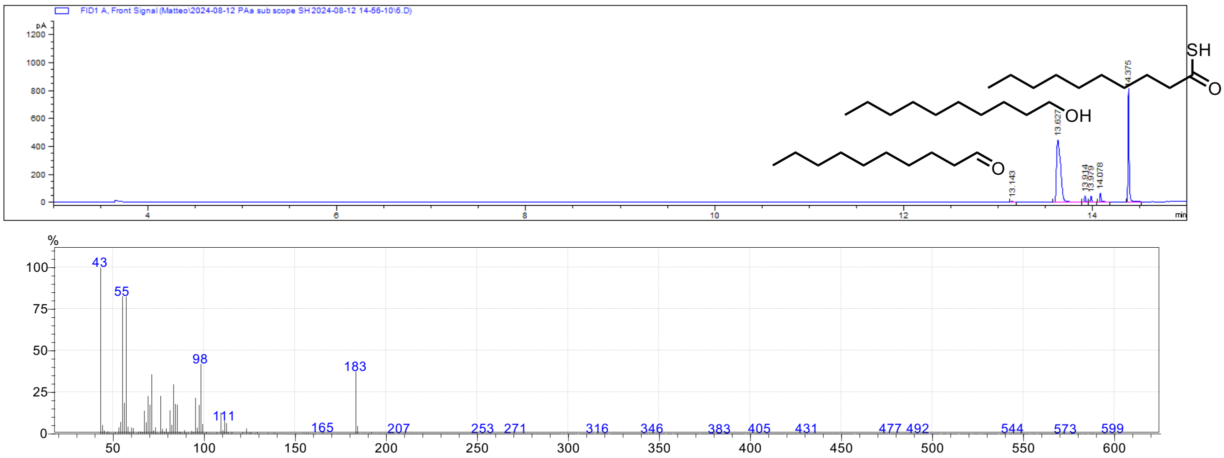

Figure S70: Up) GC-FID chromatogram for the determination of the conversion of 6f in hydrogen sulfide formate with Aa-ADH. Bottom) GC-MS of 6f.

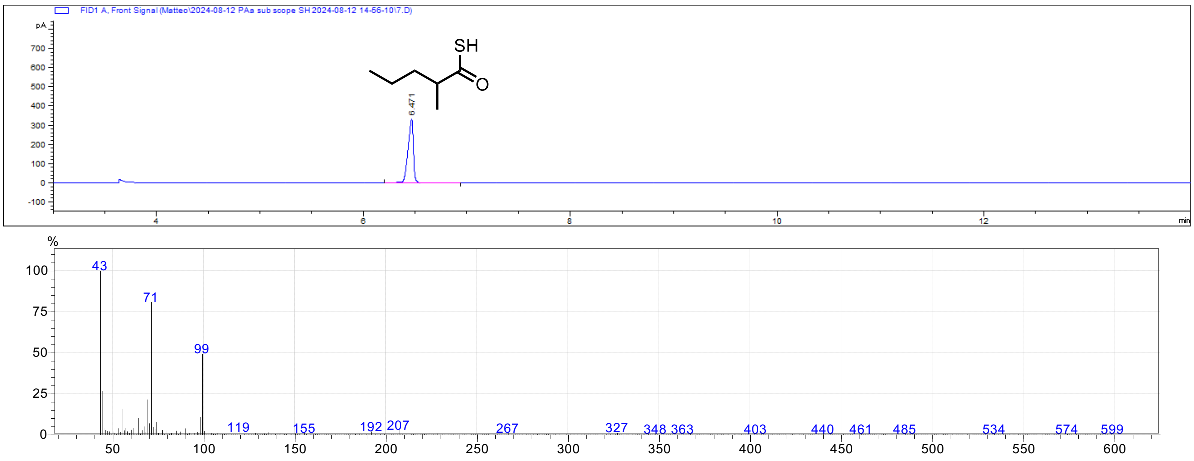

Figure S71: Up) GC-FID chromatogram for the determination of the conversion of 7f in hydrogen sulfide formate with Aa-ADH. Bottom) GC-MS of 7f.

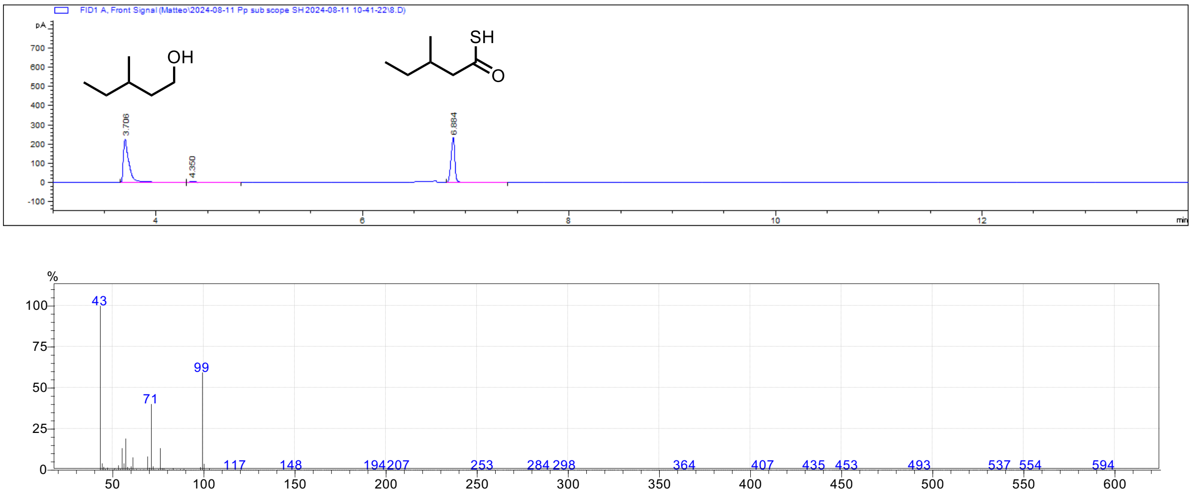

Figure S72: Up) GC-FID chromatogram for the determination of the conversion of 8f in hydrogen sulfide formate with Aa-ADH. Bottom) GC-MS of 8f.

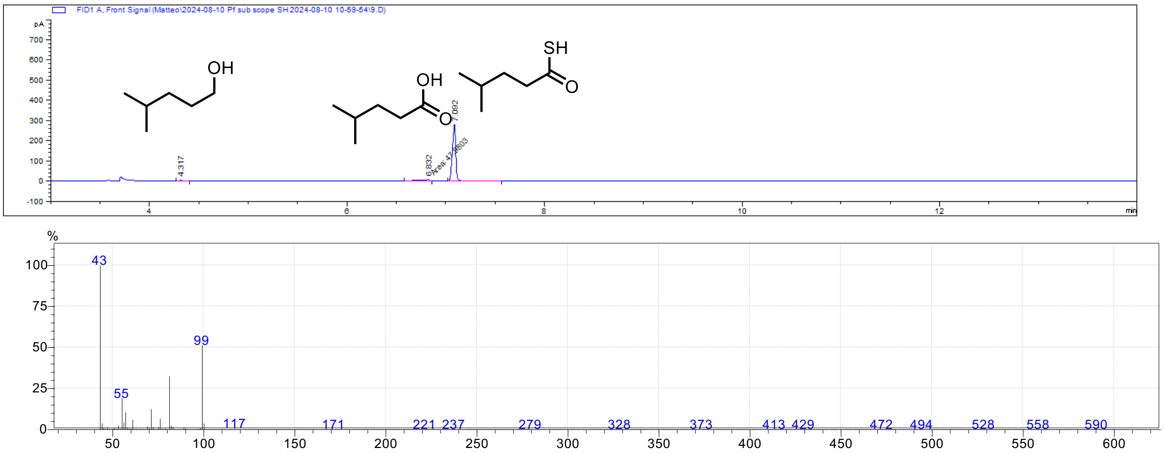

Figure S73: Up) GC-FID chromatogram for the determination of the conversion of 9f in hydrogen sulfide formate with Aa-ADH. Bottom) GC-MS of 9f.

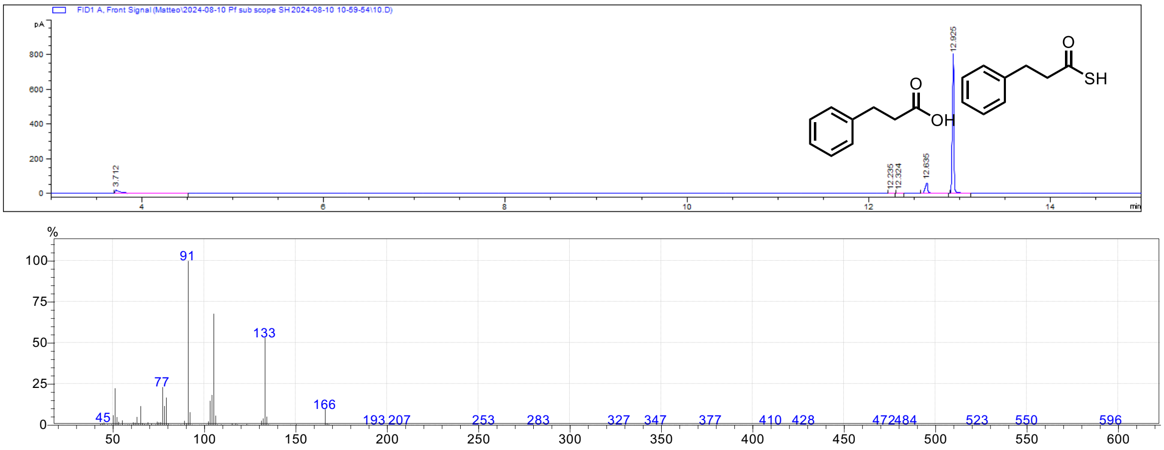

Figure S74: Up) GC-FID chromatogram for the determination of the conversion of 10f in hydrogen sulfide formate with Aa-ADH. Bottom) GC-MS of 10f.

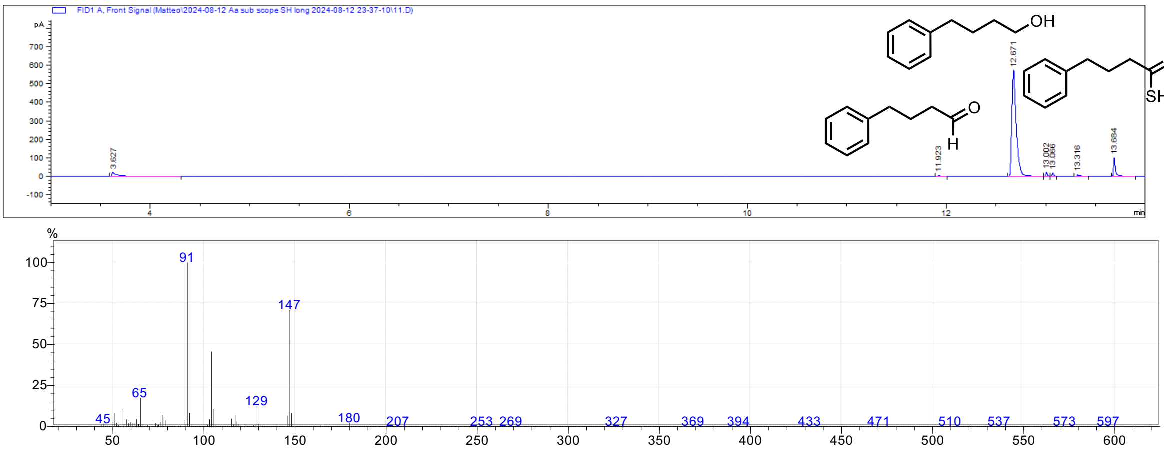

Figure S75: Up) GC-FID chromatogram for the determination of the conversion of 11f in hydrogen sulfide formate with Aa-ADH. Bottom) GC-MS of 11f.

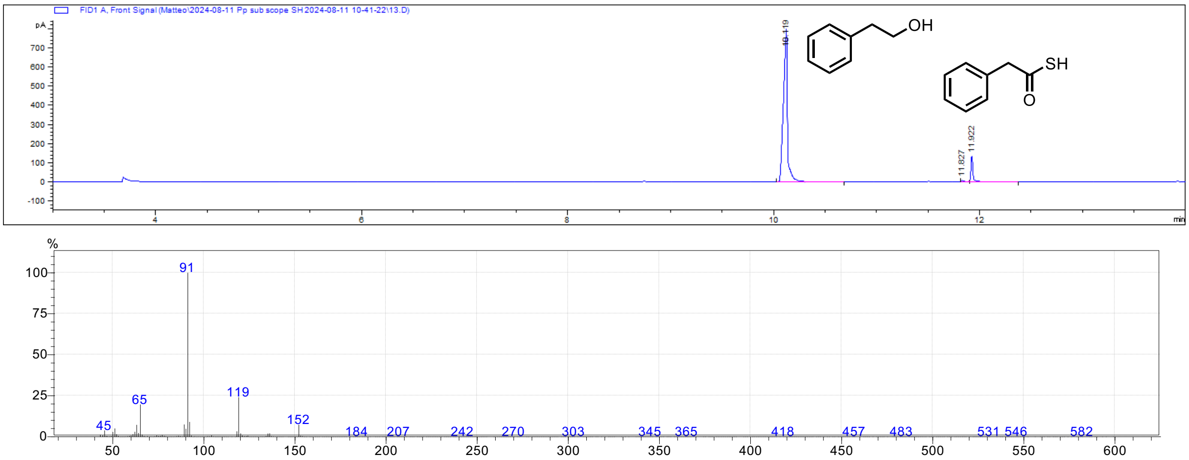

Figure S76: Up) GC-FID chromatogram for the determination of the conversion of 13f in hydrogen sulfide formate with Aa-ADH. Bottom) GC-MS of 13f.

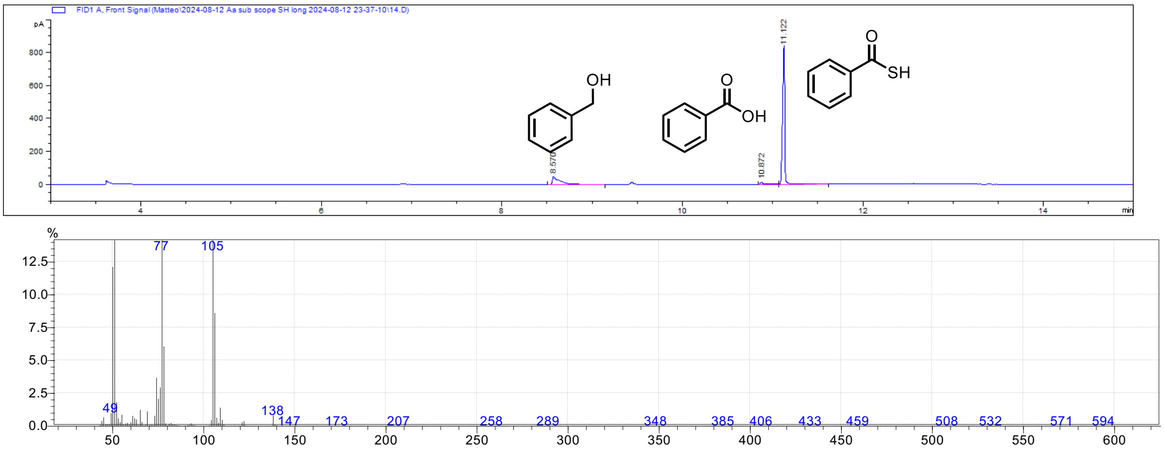

Figure S77: Up) GC-FID chromatogram for the determination of the conversion of 14f in hydrogen sulfide formate with Aa-ADH. Bottom) GC-MS of 14f.

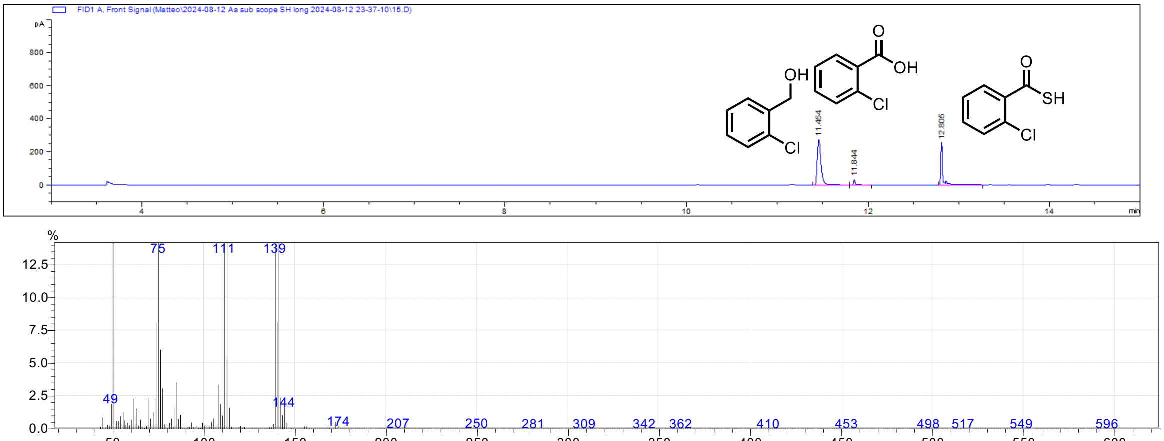

Figure S78: Up) GC-FID chromatogram for the determination of the conversion of 15f in hydrogen sulfide formate with Aa-ADH. Bottom) GC-MS of 15f.

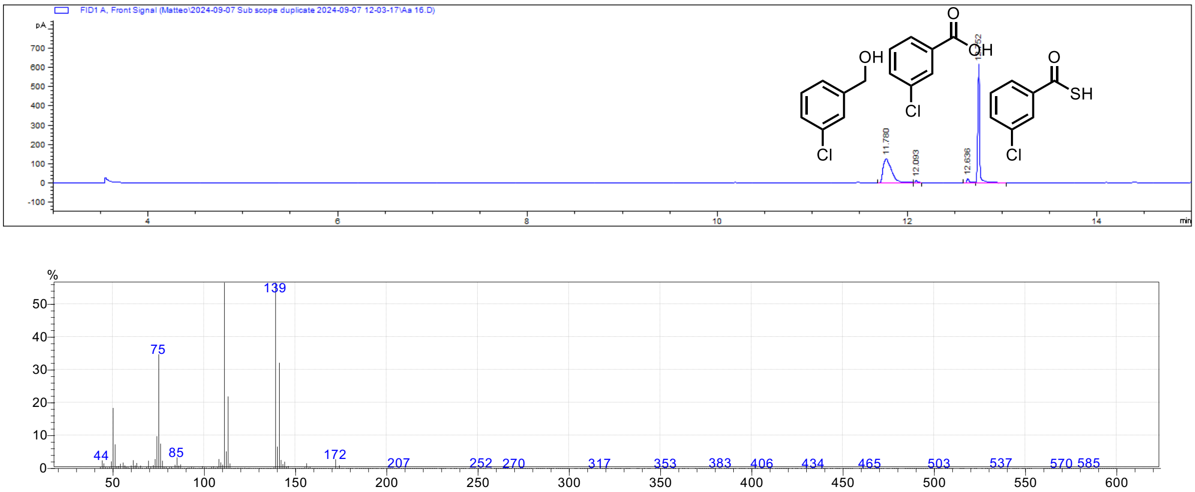

Figure S79: Up) GC-FID chromatogram for the determination of the conversion of 16f in hydrogen sulfide formate with Aa-ADH. Bottom) GC-MS of 16f.

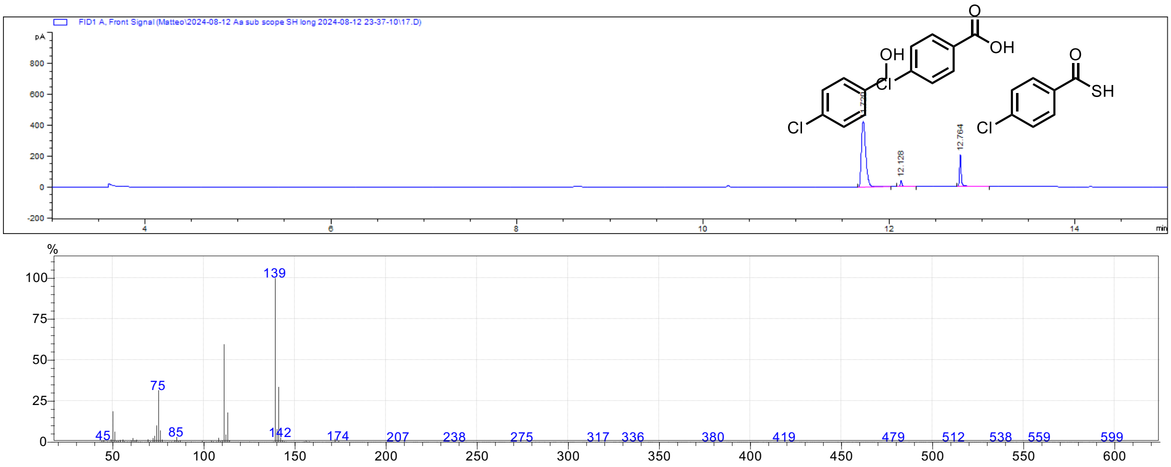

Figure S80: Up) GC-FID chromatogram for the determination of the conversion of 17f in hydrogen sulfide formate with Aa-ADH. Bottom) GC-MS of 17f.

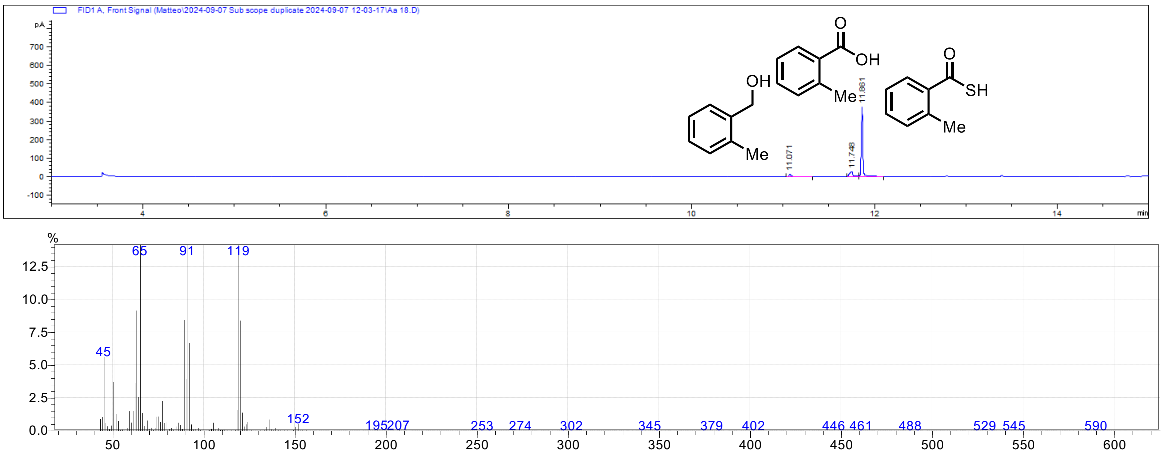

Figure S81: Up) GC-FID chromatogram for the determination of the conversion of 18f in hydrogen sulfide formate with Aa-ADH. Bottom) GC-MS of 18f.

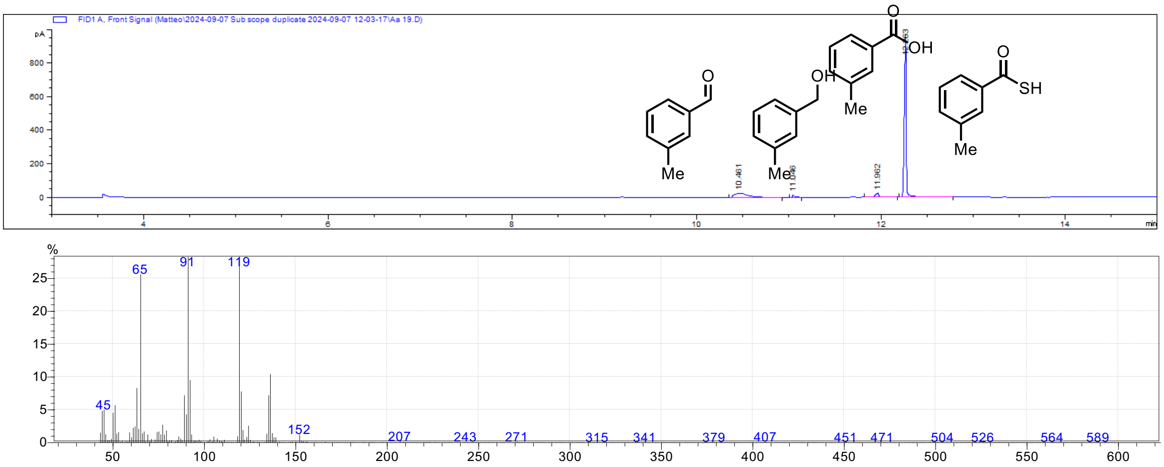

Figure S82: Up) GC-FID chromatogram for the determination of the conversion of 19f in hydrogen sulfide formate with Aa-ADH. Bottom) GC-MS of 19f.

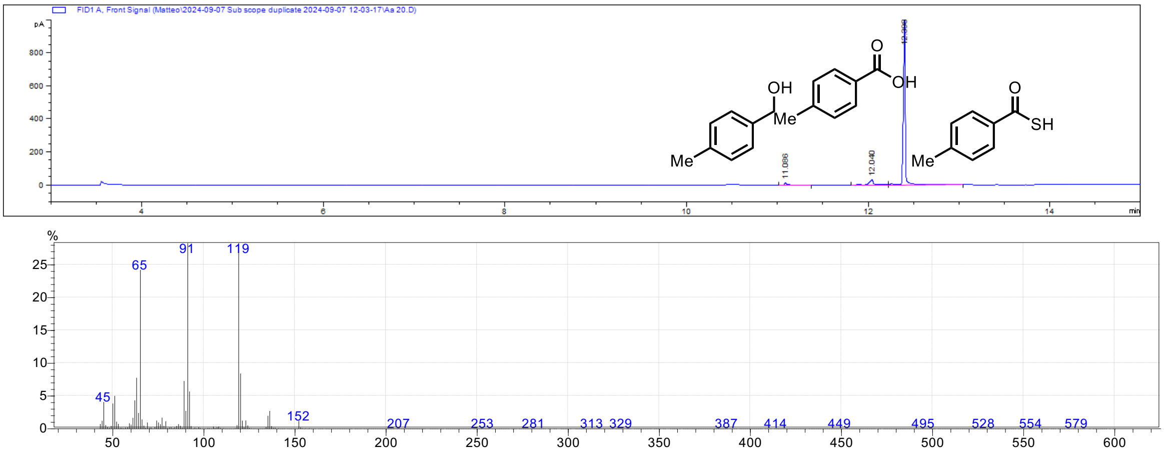

Figure S83: Up) GC-FID chromatogram for the determination of the conversion of 20f in hydrogen sulfide formate with Aa-ADH. Bottom) GC-MS of 20f.

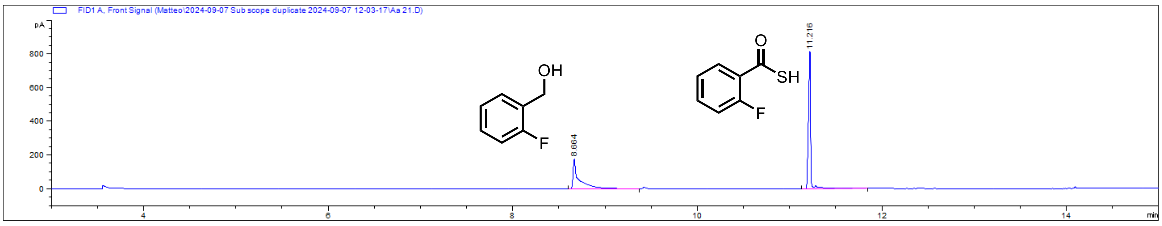

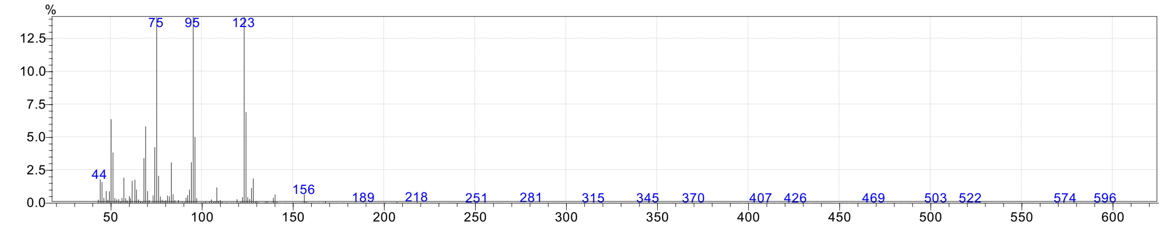

Figure S84: Up) GC-FID chromatogram for the determination of the conversion of 21f in hydrogen sulfide formate with Aa-ADH. Bottom) GC-MS of 21f.

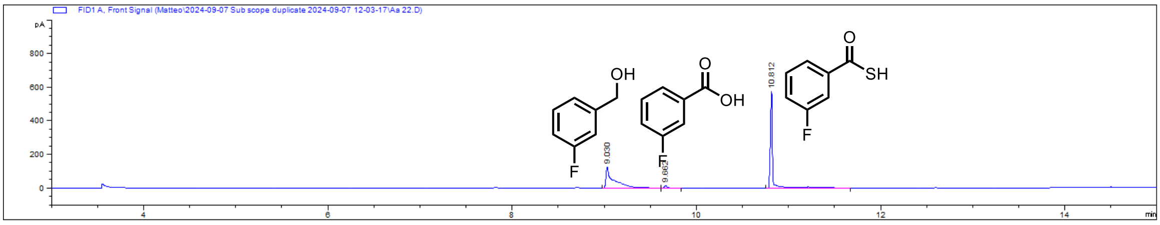

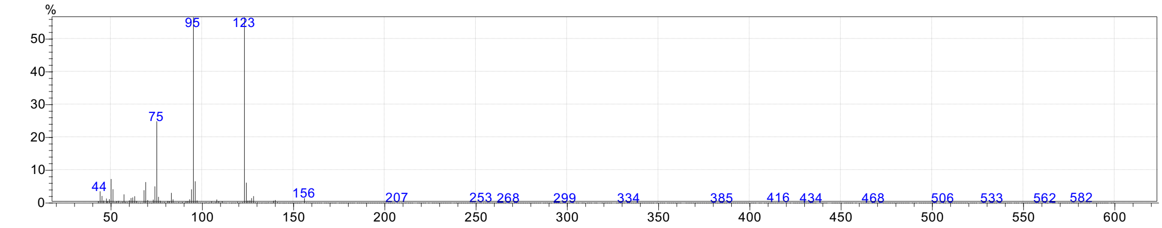

Figure S85: Up) GC-FID chromatogram for the determination of the conversion of 22f in hydrogen sulfide formate with Aa-ADH. Bottom) GC-MS of 22f.

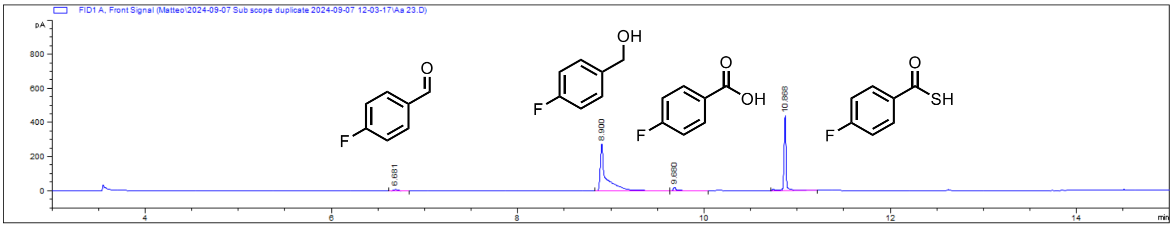

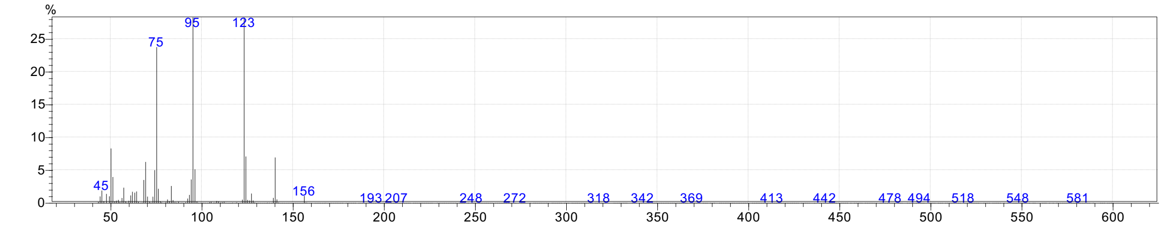

Figure S86: Up) GC-FID chromatogram for the determination of the conversion of 23f in hydrogen sulfide formate with Aa-ADH. Bottom) GC-MS of 23f.

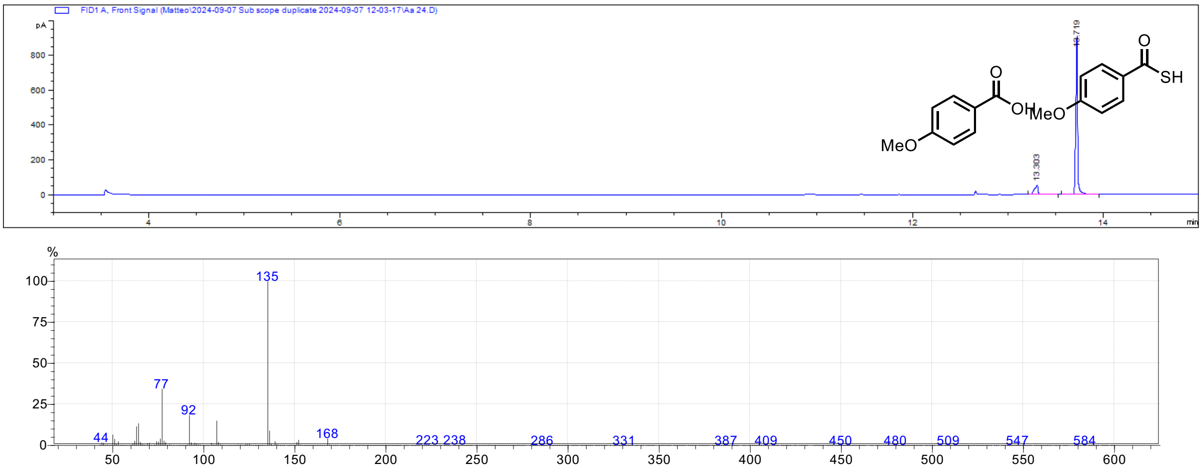

Figure S87: Up) GC-FID chromatogram for the determination of the conversion of 24f in hydrogen sulfide formate with Aa-ADH. Bottom) GC-MS of 24f.

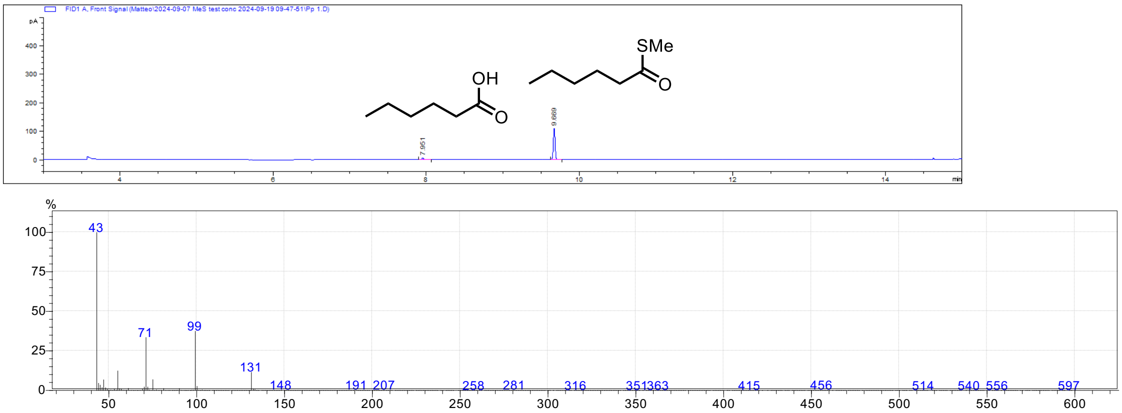

Figure S88: Up) GC-FID chromatogram for the determination of the conversion of 1g in methyl sulfide formate with Aa-ADH. Bottom) GC-MS of 1g.

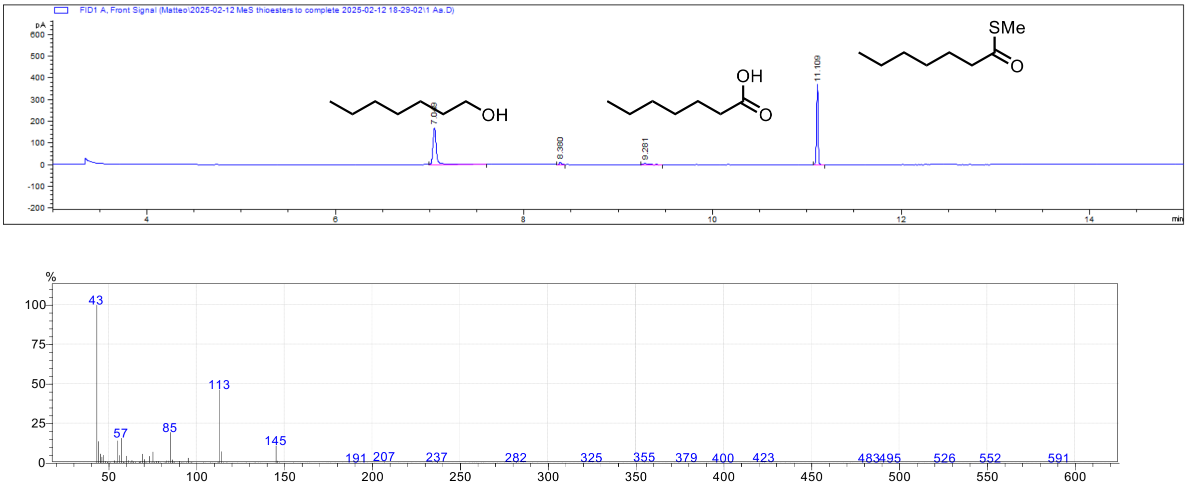

Figure S89: Up) GC-FID chromatogram for the determination of the conversion of 3g in methyl sulfide formate with Aa-ADH. Bottom) GC-MS of 3g.

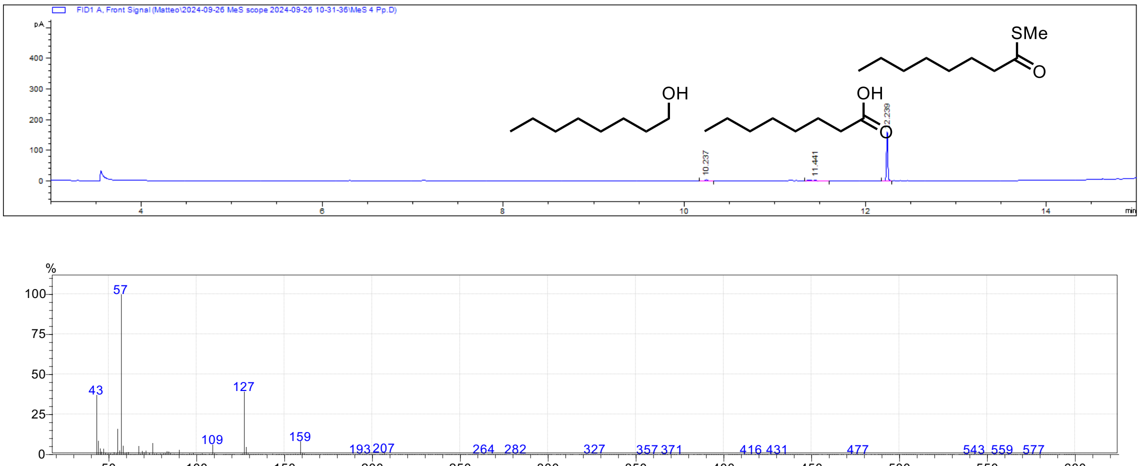

Figure S90: Up) GC-FID chromatogram for the determination of the conversion of 4g in methyl sulfide formate with Aa-ADH. Bottom) GC-MS of 4g.

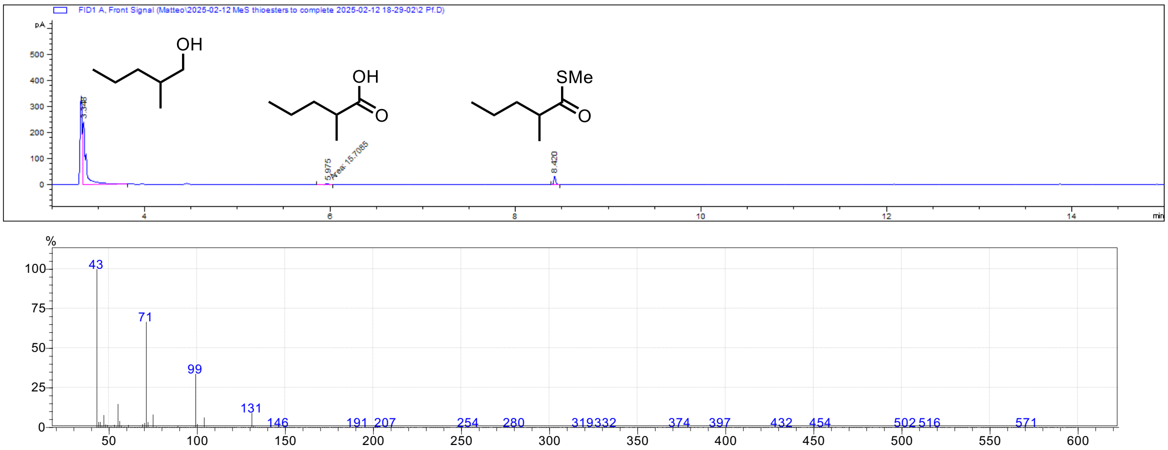

Figure S91: Up) GC-FID chromatogram for the determination of the conversion of 7g in methyl sulfide formate with Aa-ADH. Bottom) GC-MS of 7g.

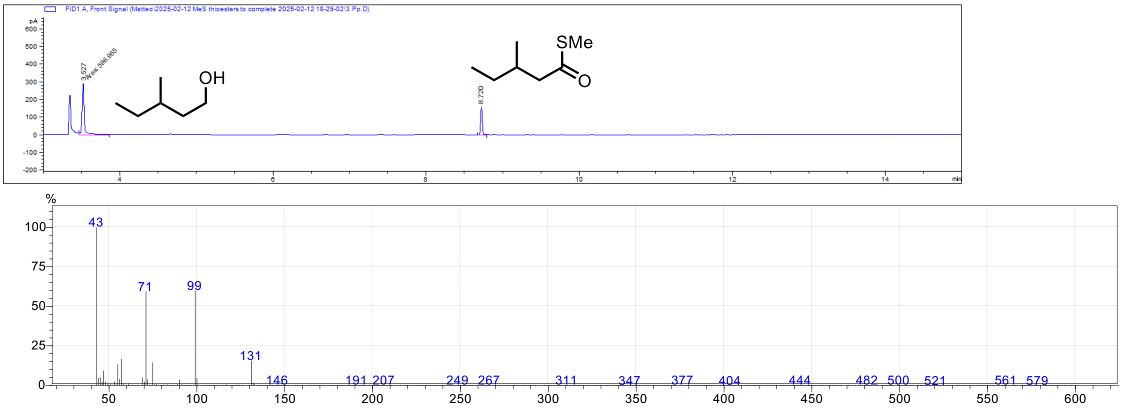

Figure S92: Up) GC-FID chromatogram for the determination of the conversion of 8g in methyl sulfide formate with Aa-ADH. Bottom) GC-MS of 8g.

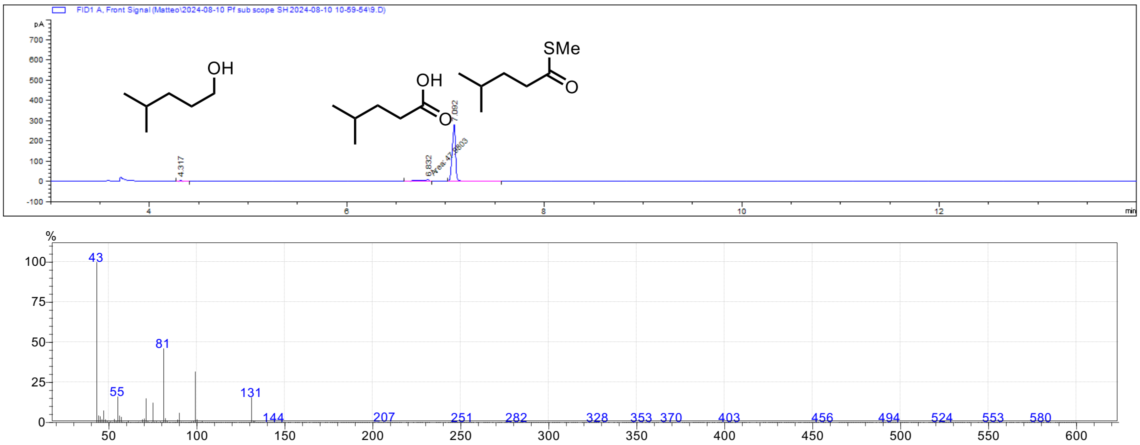

Figure S93: Up) GC-FID chromatogram for the determination of the conversion of 9g in methyl sulfide formate with Aa-ADH. Bottom) GC-MS of 9g.

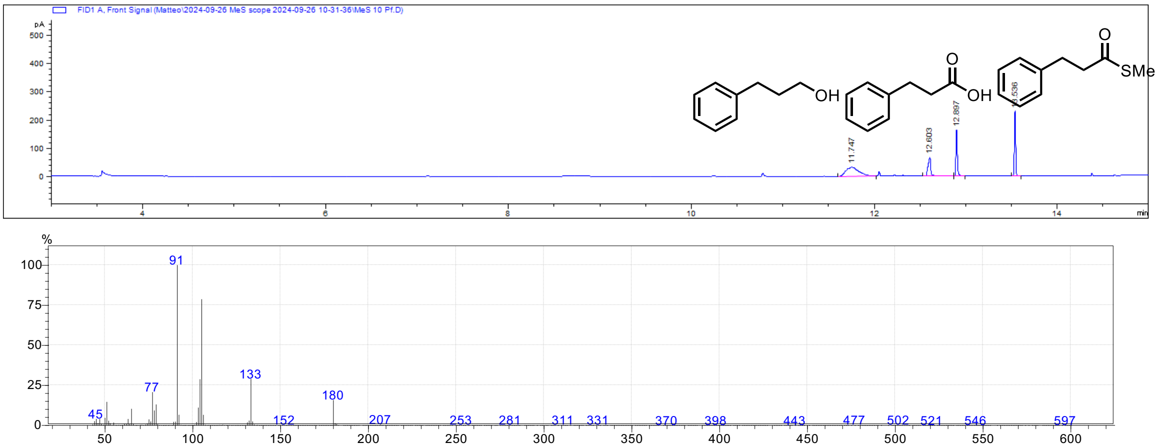

Figure S94: Up) GC-FID chromatogram for the determination of the conversion of 10g in methyl sulfide formate with Aa-ADH. Bottom) GC-MS of 10g.

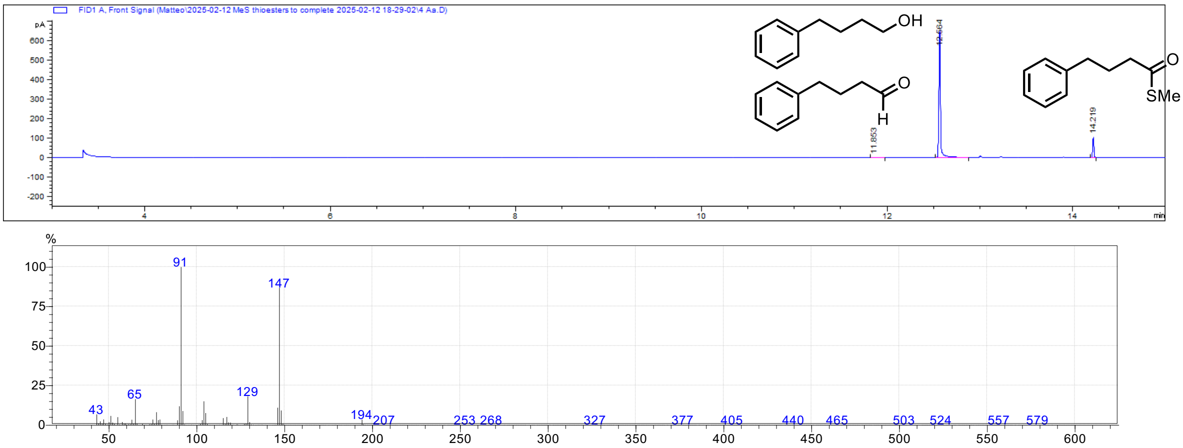

Figure S95: Up) GC-FID chromatogram for the determination of the conversion of 11g in methyl sulfide formate with Aa-ADH. Bottom) GC-MS of 11g.

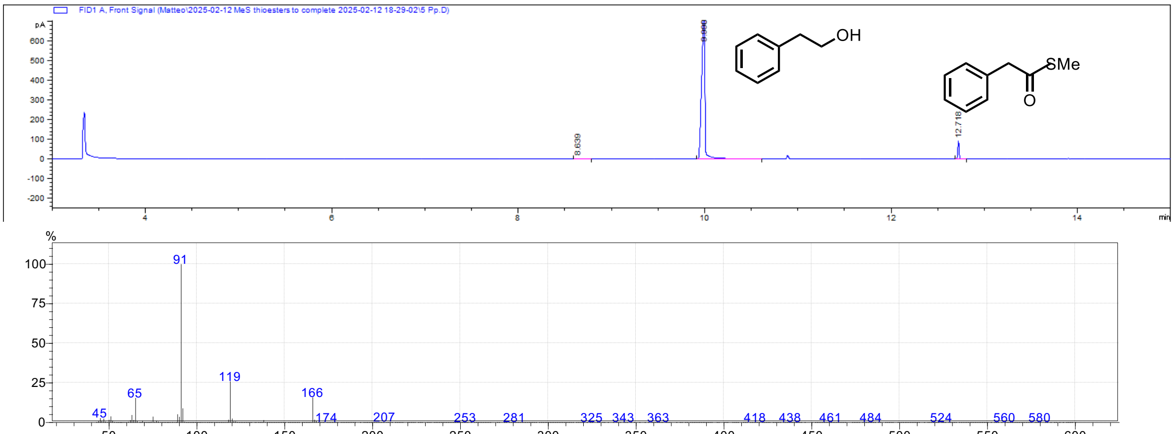

Figure S96: Up) GC-FID chromatogram for the determination of the conversion of 13g in methyl sulfide formate with Aa-ADH. Bottom) GC-MS of 13g.

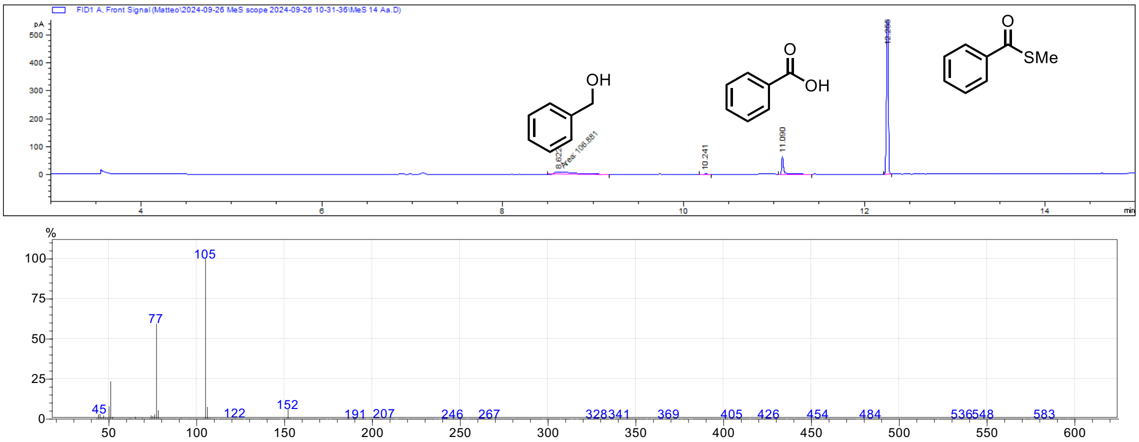

Figure S97: Up) GC-FID chromatogram for the determination of the conversion of 14g in methyl sulfide formate with Aa-ADH. Bottom) GC-MS of 14g.

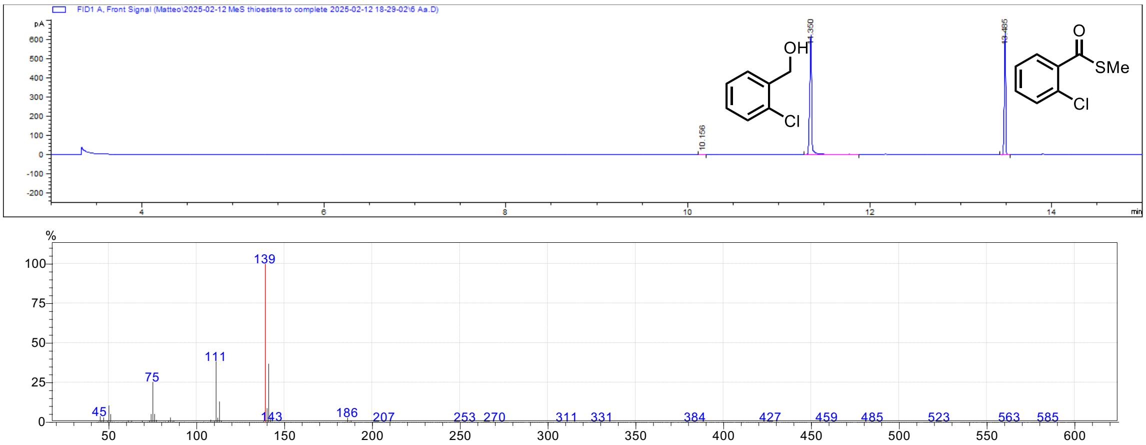

Figure S98: Up) GC-FID chromatogram for the determination of the conversion of 15g in methyl sulfide formate with Aa-ADH. Bottom) GC-MS of 15g.

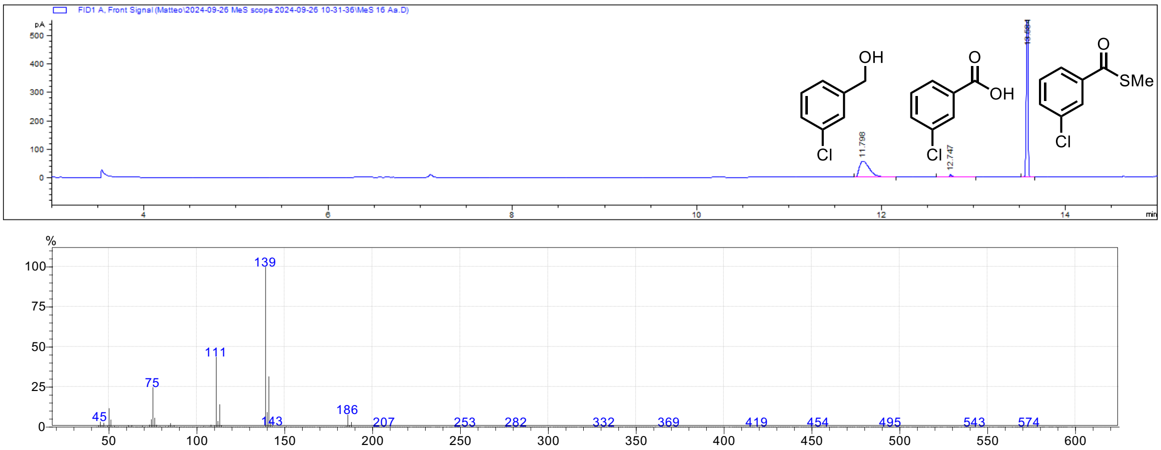

Figure S99: Up) GC-FID chromatogram for the determination of the conversion of 16g in methyl sulfide formate with Aa-ADH. Bottom) GC-MS of 16g.

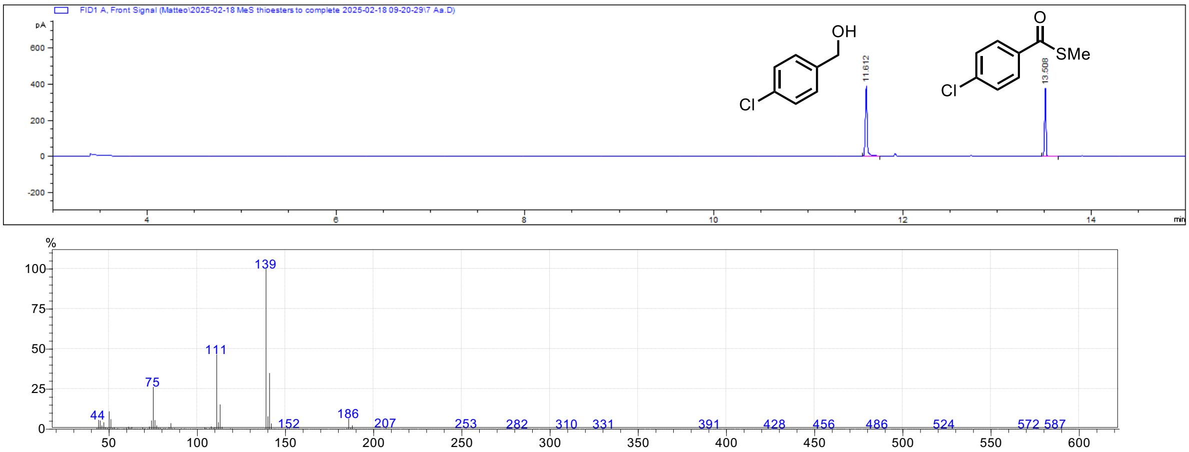

Figure S100: Up) GC-FID chromatogram for the determination of the conversion of 17g in methyl sulfide formate with Aa-ADH. Bottom) GC-MS of 17g.

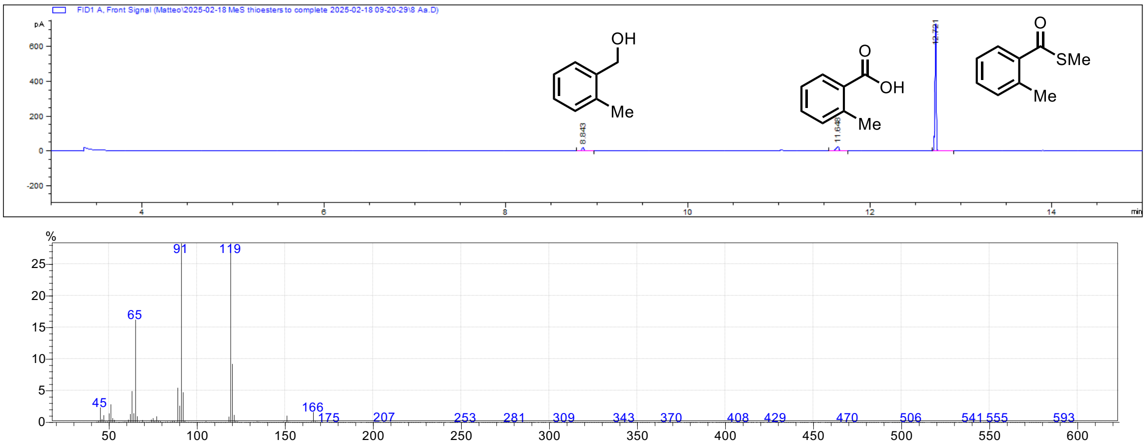

Figure S101: Up) GC-FID chromatogram for the determination of the conversion of 18g in methyl sulfide formate with Aa-ADH. Bottom) GC-MS of 18g.

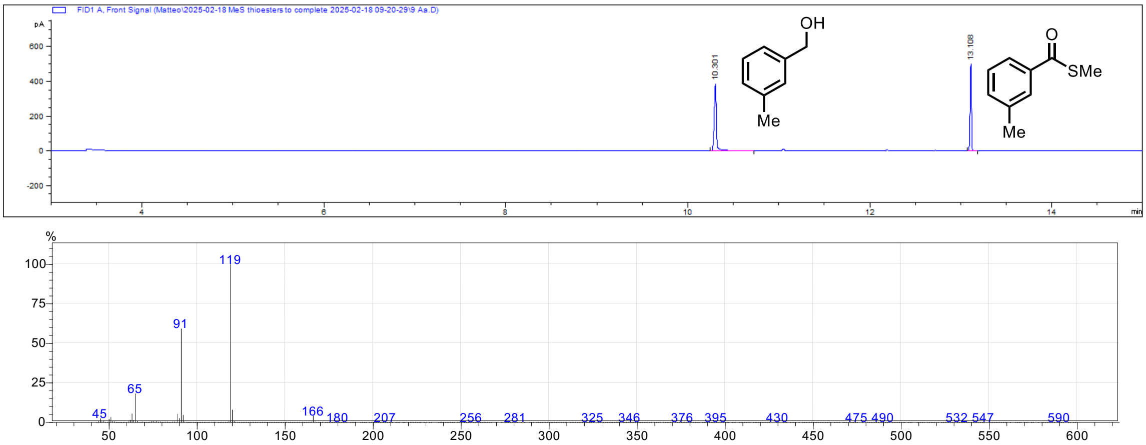

Figure S102: Up) GC-FID chromatogram for the determination of the conversion of 19g in methyl sulfide formate with Aa-ADH. Bottom) GC-MS of 19g.

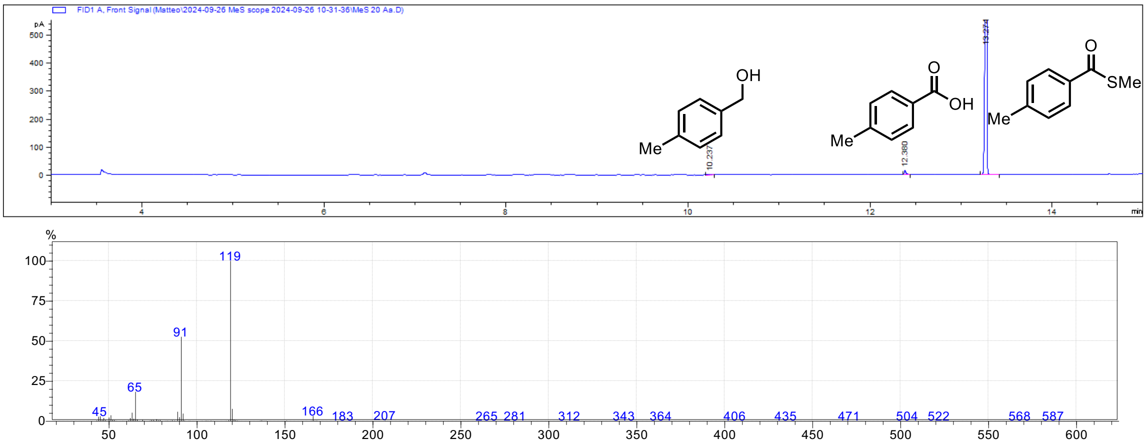

Figure S103: Up) GC-FID chromatogram for the determination of the conversion of 20g in methyl sulfide formate with Aa-ADH. Bottom) GC-MS of 20g.

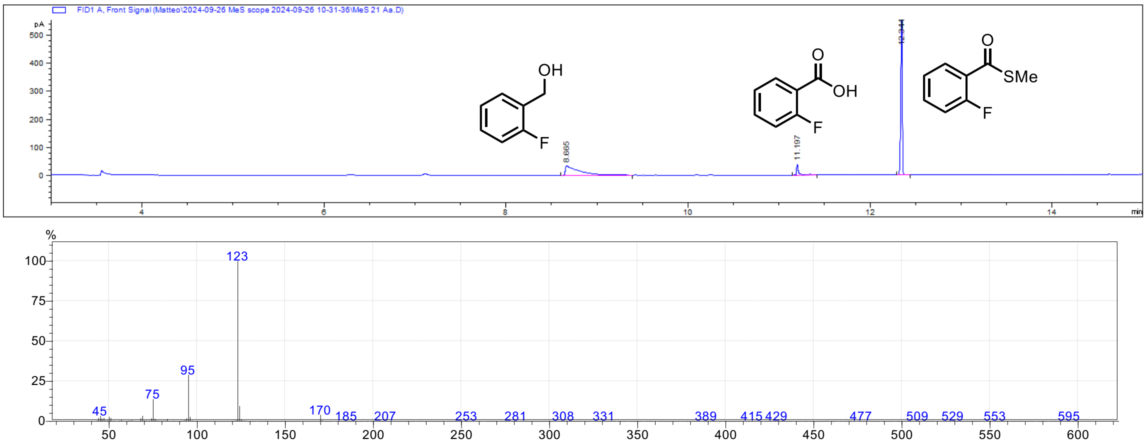

Figure S104: Up) GC-FID chromatogram for the determination of the conversion of 21g in methyl sulfide formate with Aa-ADH. Bottom) GC-MS of 21g.

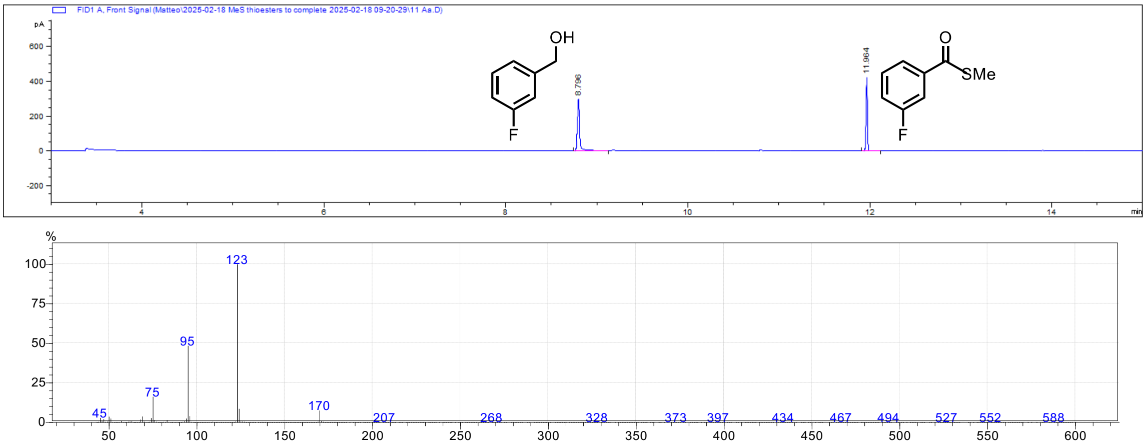

Figure S105: Up) GC-FID chromatogram for the determination of the conversion of 22g in methyl sulfide formate with Aa-ADH. Bottom) GC-MS of 22g.

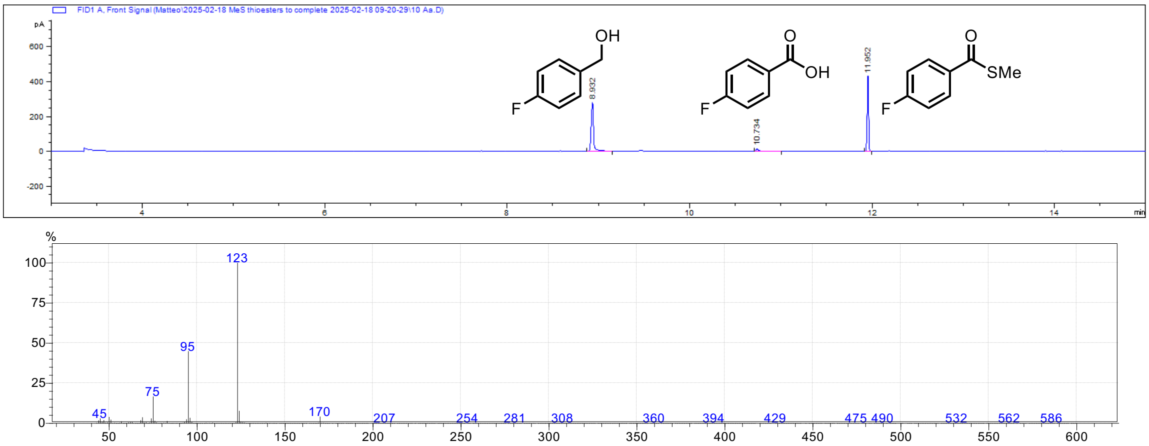

Figure S106: Up) GC-FID chromatogram for the determination of the conversion of 23g in methyl sulfide formate with Aa-ADH. Bottom) GC-MS of 23g.

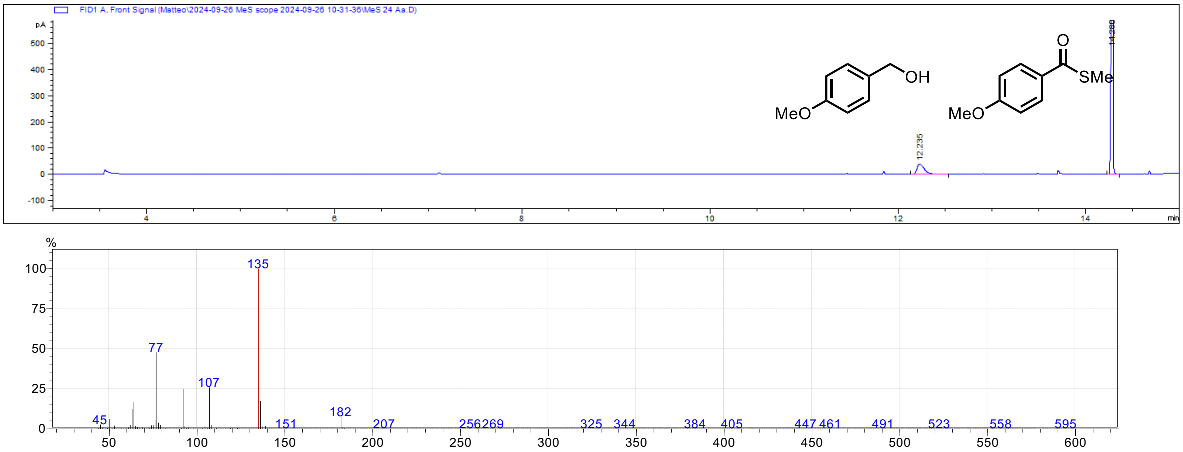

Figure S107: Up) GC-FID chromatogram for the determination of the conversion of 24g in methyl sulfide formate with Aa-ADH. Bottom) GC-MS of 24g.

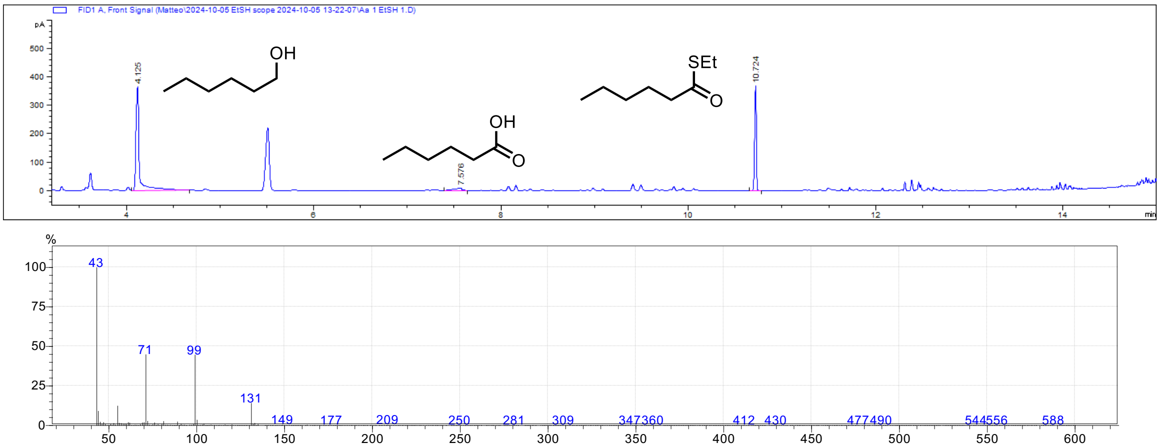

Figure S108: Up) GC-FID chromatogram for the determination of the conversion of 1h in biphasic system with Aa-ADH. Bottom) GC-MS of 1h.

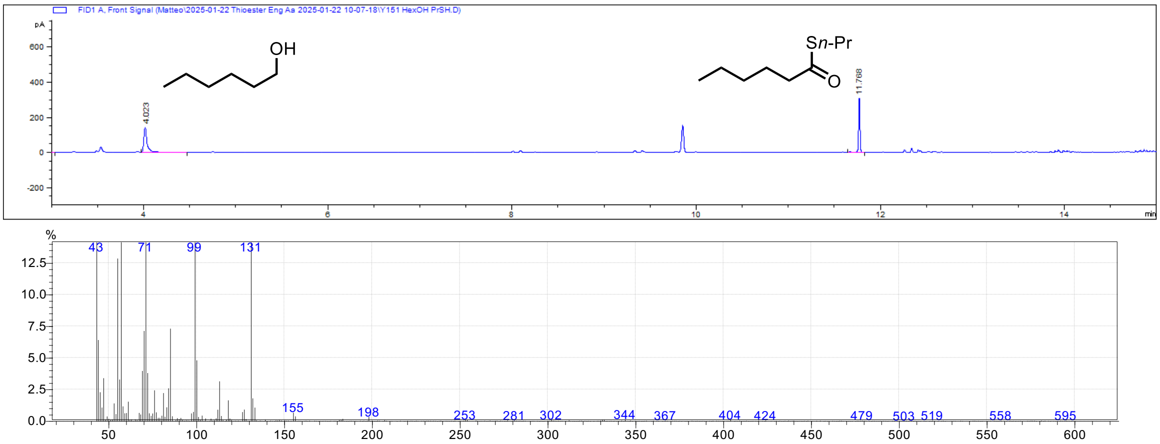

Figure S109: Up) GC-FID chromatogram for the determination of the conversion of 1l in biphasic system with Y151A Aa-ADH. Bottom) GC-MS of 1l.

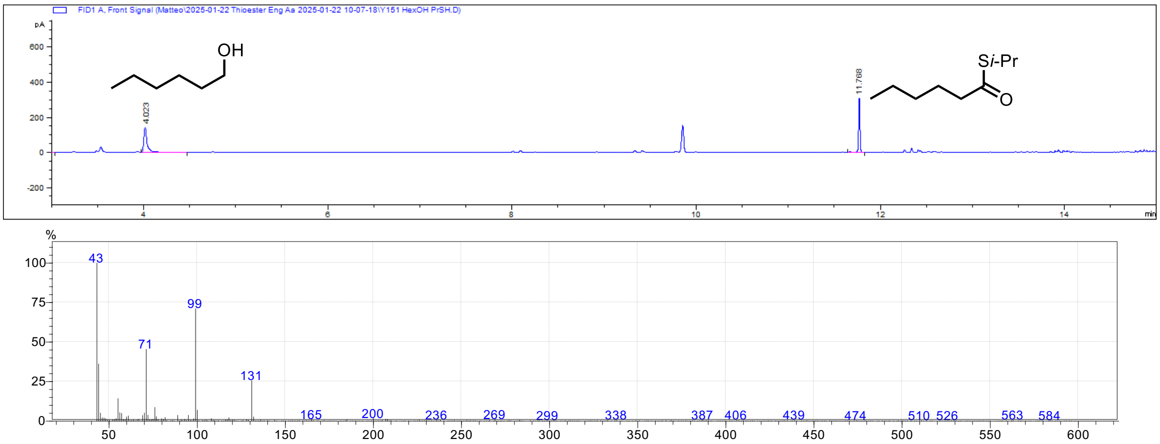

Figure S110: Up) GC-FID chromatogram for the determination of the conversion of 1m in biphasic system with Y151A Aa-ADH. Bottom) GC-MS of 1m.

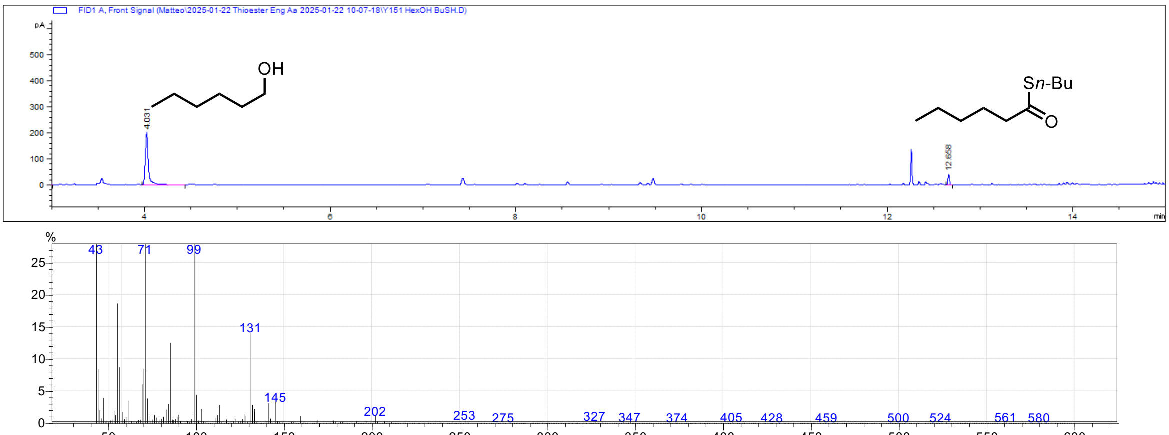

Figure S111: Up) GC-FID chromatogram for the determination of the conversion of 1n in biphasic system with Y151A Aa-ADH. Bottom) GC-MS of 1n.

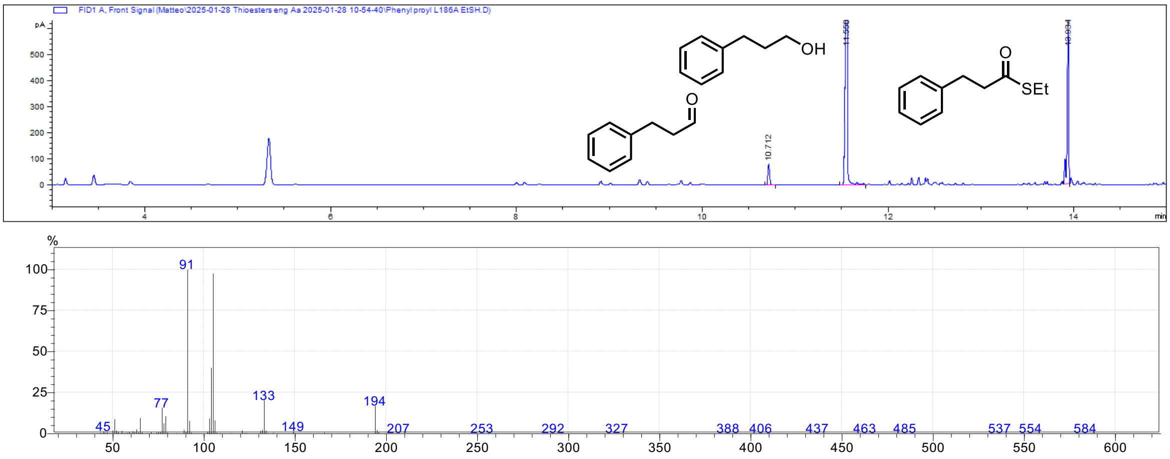

Figure S112: Up) GC-FID chromatogram for the determination of the conversion of 10h in biphasic system with Y151A Aa-ADH. Bottom) GC-MS of 10h.

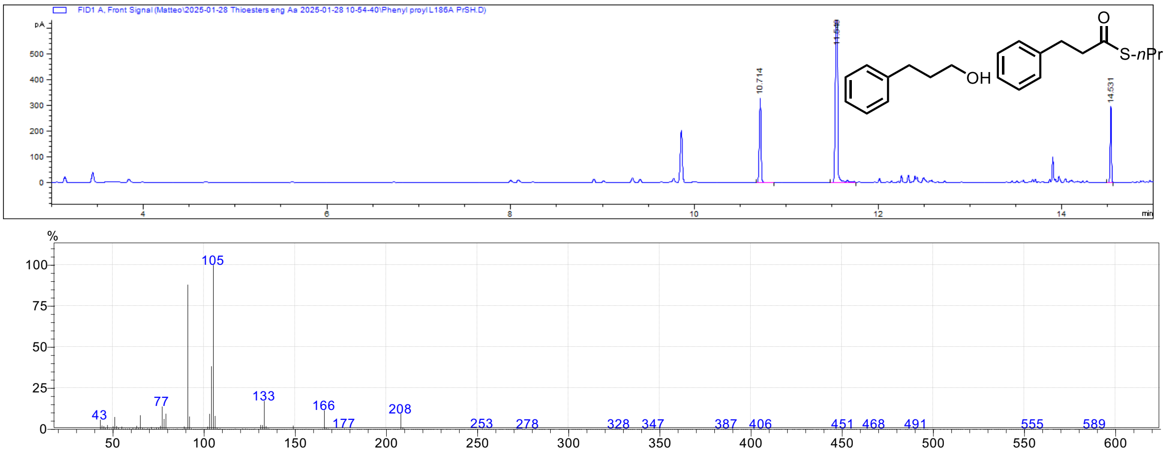

Figure S113: Up) GC-FID chromatogram for the determination of the conversion of 10l in biphasic system with Y151A Aa-ADH. Bottom) GC-MS of 10l.

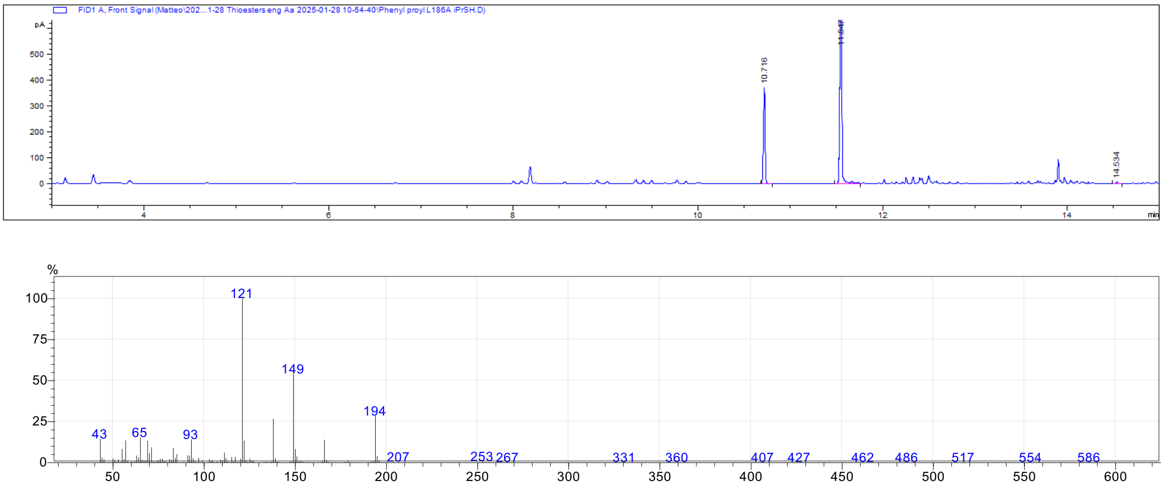

Figure S114: Up) GC-FID chromatogram for the determination of the conversion of 10m in biphasic system with Y151A Aa-ADH. Bottom) GC-MS of 10m.

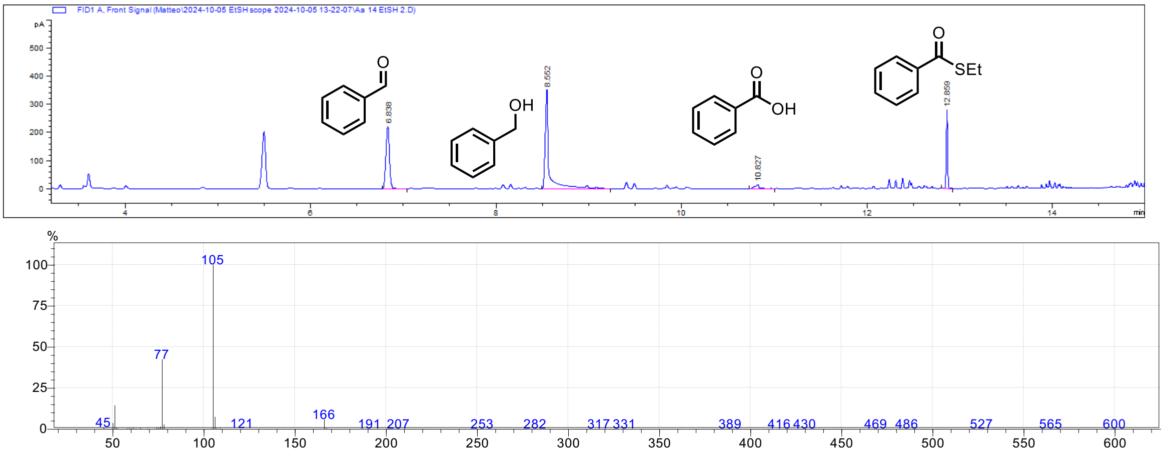

Figure S115: Up) GC-FID chromatogram for the determination of the conversion of 14h in biphasic system with Y151A Aa-ADH. Bottom) GC-MS of 14h.

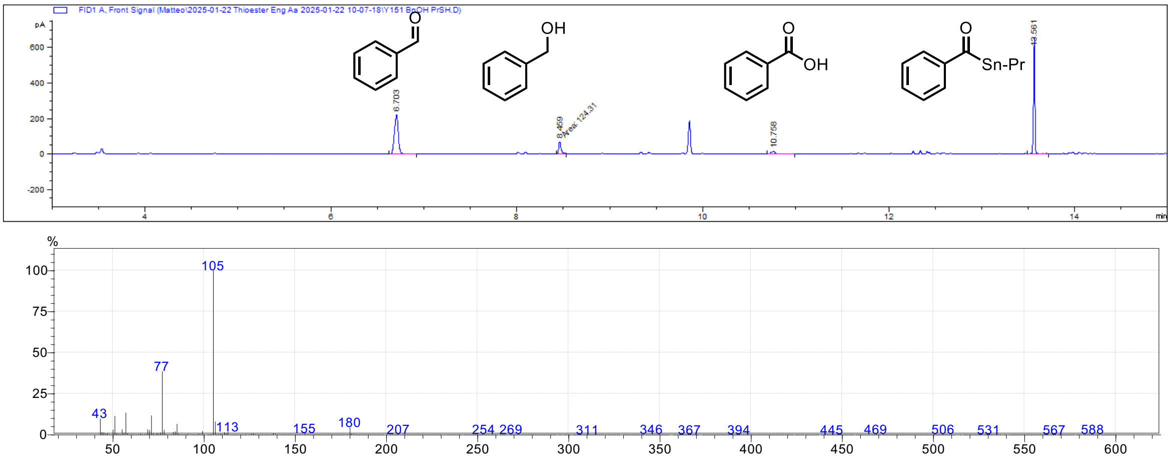

Figure S116: Up) GC-FID chromatogram for the determination of the conversion of 14l in biphasic system with Y151A Aa-ADH. Bottom) GC-MS of 14l.

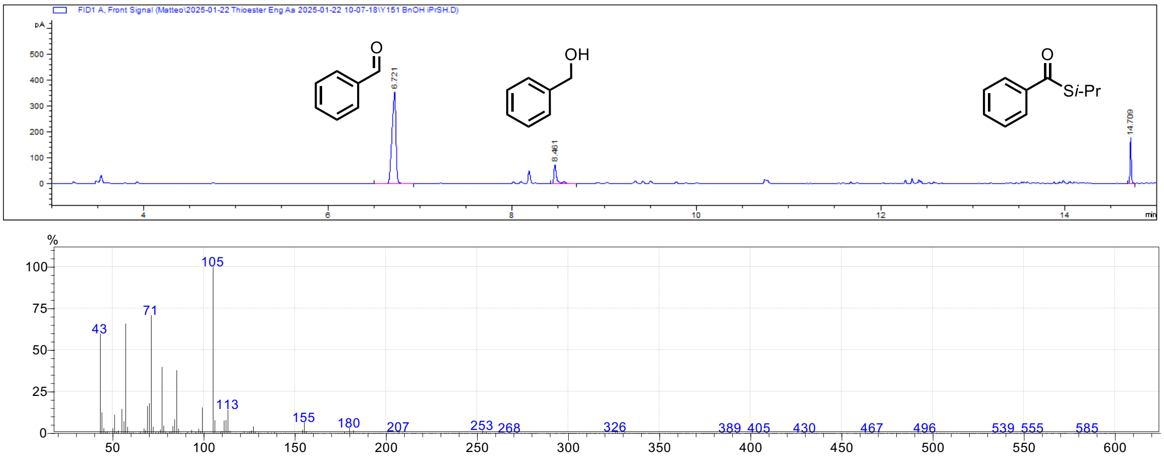

Figure S117: Up) GC-FID chromatogram for the determination of the conversion of 14m in biphasic system with Y151A Aa-ADH. Bottom) GC-MS of 14m.

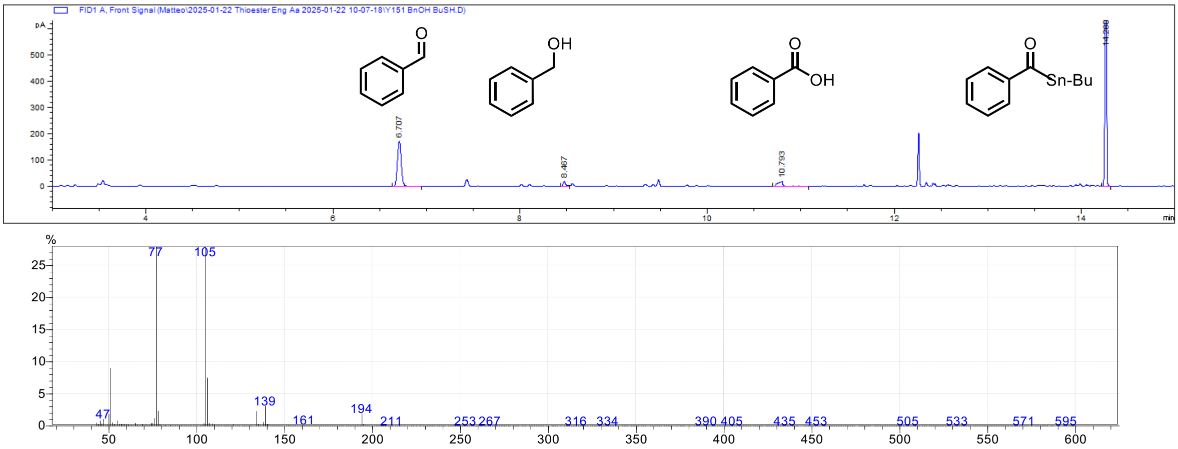

Figure S118: Up) GC-FID chromatogram for the determination of the conversion of 14n in biphasic system with Y151A Aa-ADH. Bottom) GC-MS of 14n.

16. References

(1) Damian, M.; Mutti, F. G. Two anti-Prelog NAD-Dependent Alcohol Dehydrogenases with Broad Substrate Scope and Excellent Enantioselectivity. *Eur. J. Org. Chem.* **2023**, *26* (47), e202300734.

(2) Höffken, H. W.; Duong, M.; Friedrich, T.; Breuer, M.; Hauer, B.; Reinhardt, R.; Rabus, R.; Heider, J. Crystal structure and enzyme kinetics of the (S)-specific 1-phenylethanol dehydrogenase of the denitrifying bacterium strain EbN1. *Biochemistry* **2006**, *45* (1), 82-93.

(3) Mutti, F. G.; Knaus, T.; Scrutton, N. S.; Breuer, M.; Turner, N. J. Conversion of alcohols to enantiopure amines through dual-enzyme hydrogen-borrowing cascades. *Science* **2015**, *349* (6255), 1525-1529.

(4) Lavandera, I.; Hoeller, B.; Kern, A.; Ellmer, U.; Glieder, A.; de Wildeman, S.; Kroutil, W. Asymmetric anti-Prelog reduction of ketones catalysed by Paracoccus pantotrophus and Comamonas sp. cells via hydrogen transfer. *Tetrahedron: Asymmetry* **2008**, *19* (16), 1954-1958.

(5) Cannio, R.; Rossi, M.; Bartolucci, S. A few amino acid substitutions are responsible for the higher thermostability of a novel NAD+-dependent bacillar alcohol dehydrogenase. *Eur. J. Biochem.* **1994**, *222* (2), 345-352.

(6) Musa; Ziegelmann-Fjeld, K. I.; Vieille, C.; Zeikus, J. G.; Phillips, R. S. Asymmetric reduction and oxidation of aromatic ketones and alcohols using W110A secondary alcohol dehydrogenase from Thermoanaerobacter ethanolicus. *J. Org. Chem.* **2007**, *72* (1), 30-34. Knaus, T.; Cariati, L.; Masman, M. F.; Mutti, F. G. In vitro biocatalytic pathway design: orthogonal network for the quantitative and stereospecific amination of alcohols. *Org. Biomol. Chem.* **2017**, *15* (39), 8313-8325.

(7) Corrado, M. L.; Knaus, T.; Mutti, F. G. Regio-and stereoselective multi-enzymatic aminohydroxylation of β-methylstyrene using dioxygen, ammonia and formate. *Green Chem.* **2019**, *21* (23), 6246-6251. Zhang, J.; Xu, T.; Li, Z. Enantioselective Biooxidation of Racemic trans-Cyclic Vicinal Diols: One-Pot Synthesis of Both Enantiopure (S, S)-Cyclic Vicinal Diols and (R)-α-Hydroxy Ketones. *Adv. Synth. Catal.* **2013**, *355* (16), 3147-3153.

(8) Matsumoto, J.; Higuchi, M.; Shimada, M.; Yamamoto, Y.; Kamio, Y. Molecular cloning and sequence analysis of the gene encoding the H2O-forming NADH oxidase from Streptococcus mutans. *Biosci. Biotechnol. Biochem.* **1996**, *60* (1), 39-43.

(9) Duan, Y.; Wu, C.; Chowdhury, S.; Lee, M. C.; Xiong, G.; Zhang, W.; Yang, R.; Cieplak, P.; Luo, R.; Lee, T.; et al. A point-charge force field for molecular mechanics simulations of proteins based on condensed-phase quantum mechanical calculations. *J. Comput. Chem.* **2003**, *24* (16), 1999-2012.
